# Supplementary material for: A general deoxygenation approach for synthesis of ketones from aromatic carboxylic acids and alkenes
Source: Nat Commun. 2018 Aug 29;9:3517. doi: 10.1038/s41467-018-06019-1 (PMC6115474; doi:10.1038/s41467-018-06019-1)
Supplement: Supplementary file 1 — Supplementary Information [file 41467_2018_6019_MOESM1_ESM.pdf]

Supplementary Information

A General Deoxygenation Approach for Synthesis of Ketones from Aromatic Carboxylic Acids and Alkenes

Zhang et al.

Supplementary Methods

General Information

All reactions were carried out under Ar atmosphere unless otherwise noted. All reagents and solvents were obtained from commercial suppliers and used without further purification. Reactions were monitored by TLC on silica gel plates (GF_{254}), and the analytical thin-layer chromatography (TLC) was performed on precoated, glass-backed silica gel plates. ^{1}H NMR, ^{13}C NMR and ^{31}P NMR spectra were recorded on a Bruker AVANCE Ⅲ-400 spectrometer at room temperature. Chemical shifts (δ) are reported in ppm downfield from tetramethylsilane. Abbreviations for signal couplings are: s, singlet; d, doublet; t, triplet; m, multiplet; broad (br). Gas chromatographic (GC) analyses were performed on a GC equipped with a flameionization detector and an Rtx@-65 (30 m × 0.32 mm ID × 0.25 μm df) column. GC-MS analyses were performed on a GC-MS with an EI mode. High resolution mass spectra were obtained using an Agilent 6210 Series TOF LC-MS equipped with electrospray ionization (ESI) probe operating in positive ion mode. HPLC analysis was performed on Shimadzu SPD-20A using Daicel Chiralpak AD Column. The 5 W blue LED lamps (λ_{max} = 455 nm) and 36W CFL were directly got from the supermarket.

Supplementary Table 1. Optimization of the Reaction Conditions[a]

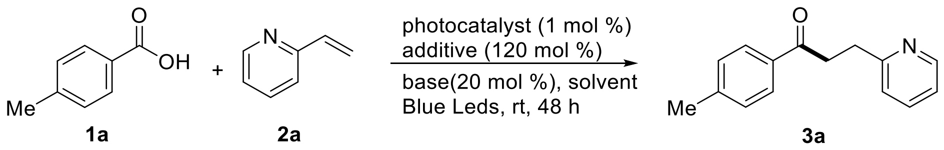

entry	cat	additive	base	solvent	yield[b]
1	**I**	Ph_3P	K_2HPO_4	CH_3CN	25
2	**I**	Ph_3P	K_2HPO_4	DMSO	20
3	**I**	Ph_3P	K_2HPO_4	DMF	18
4	**I**	Ph_3P	K_2HPO_4	DCM	40
5	**I**	Ph_3P	K_2HPO_4	MeOH-H_2O (4:1)	trace
6	**I**	Ph_3P	K_2HPO_4	DMF-H_2O (4:1)	trace

7	**I**	Ph_3P	K_2HPO_4	DMSO-H_2O (4:1)	trace
8	**I**	Ph_3P	K_2HPO_4	DCM/H_2O (4:1)	72
9	**I**	$(EtO)_3P$	K_2HPO_4	DCM/H_2O (4:1)	0
10	**I**	*n*-Bu_3P	K_2HPO_4	DCM/H_2O (4:1)	0
11	**I**	Ph_3P	Cs_2CO_3	DCM/H_2O (4:1)	37
12	**I**	Ph_3P	K_2CO_3	DCM/H_2O (4:1)	51
13	**I**	Ph_3P	DBU	DCM/H_2O (4:1)	14
14[c]	**I**	Ph_3P	K_2HPO_4	DCM/H_2O (4:1)	70
15	**II**	Ph_3P	K_2HPO_4	DCM/H_2O (4:1)	ND
16	**III**	Ph_3P	K_2HPO_4	DCM/H_2O (4:1)	ND
17	**IV**	Ph_3P	K_2HPO_4	DCM/H_2O (4:1)	ND
18	**V**	Ph_3P	K_2HPO_4	DCM/H_2O (4:1)	ND

[a] The reactions were carried out with **1a** (0.2 mmol), **2a** (0.3 mmol, 1.5 equiv.), Ph_3P (0.24 mmol, 1.2 equiv.), photocatalyst (0.002 mmol, 1 mol%), base (0.04 mmol, 20 mol%), solvent (2.0 mL), at room temperature, 5W Blue LEDs, 48 h. [b] isolated yields. [c] 36 W CFL.

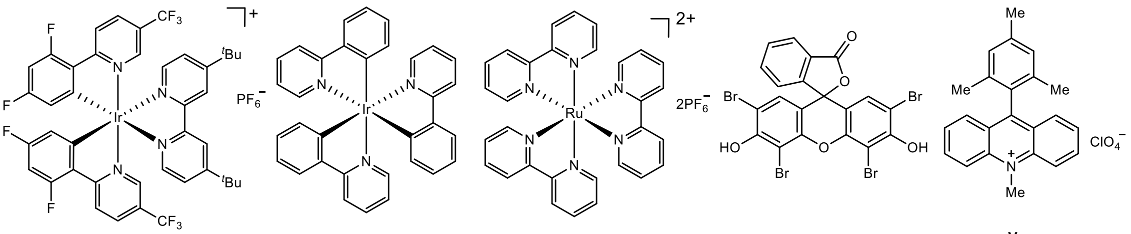

I II III IV V

General Procedure for Deoxygenative Cross-Coupling Reaction

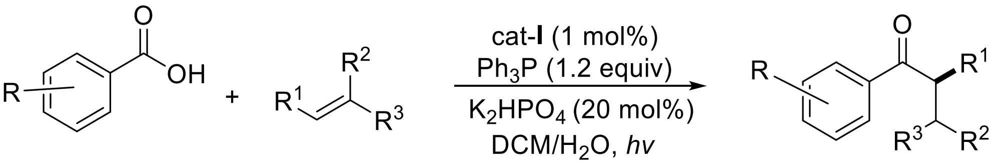

1 **2** **3**

To a 10 mL Schlenk tube equipped with a magnetic stir bar was added aromatic carboxylic acid (0.2 mmol, 1.0 equiv.), photocatalyst Ir[dF(CF_3)ppy]$_2$(dtbbpy)PF_6 (2.3 mg, 1 mol%), K_2HPO_4 (7.0 mg, 20 mol%), and Ph_3P (62.9 mg, 0.24 mmol, 1.2 equiv.) and the tube was evacuated and backfilled with Ar (three times). The alkenes **2** (0.3 mmol, 1.5 equiv.) in DCM/H_2O (2.0 mL, 4:1 v/v) were added by syringe under Ar. The tube was then sealed and was placed at a distance (app. 5 cm) from 5 W blue LEDs lamp, and the mixture was stirred for 36 - 60 h at room temperature (Supplementary Figure 1.). After completion, the mixture was quenched with water and extracted with DCM (3 x 10 mL). The combined organic layer was dried over anhydrous Na_2SO_4, then the solvent was removed under vacuo. The residue was purified with chromatography column on silica gel (gradient eluent of petroleum ether/EtOAc) to give the corresponding ketone products **3**.

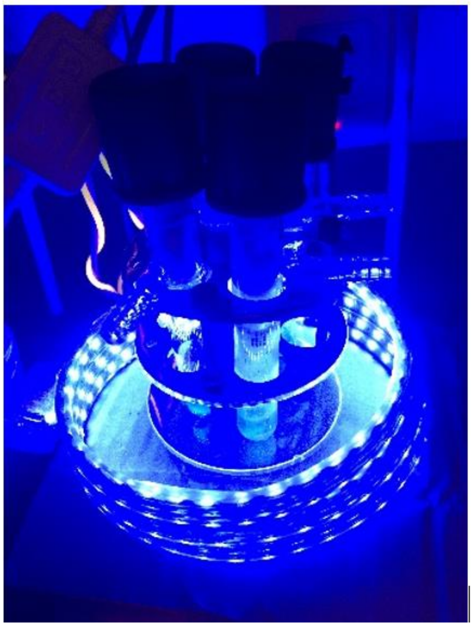

Supplementary Figure 1. Photoreactor setup

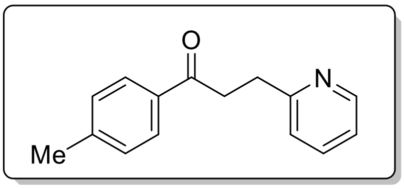

3-(pyridin-2-yl)-1-(*p*-tolyl)propan-1-one **3a**

The reaction was carried out according to the general procedure on 0.2 mmol scale (48 h). The residue was purified by flash column chromatography petroleum ether: ethyl

acetate, 10/1-4/1) to afford **3a**, (72%, 32.4 mg), yellowish oil. ^{1}H NMR (400 MHz, Chloroform-d) δ 8.51 (d, *J* = 4.8 Hz, 1H), 7.89 (d, *J* = 8.2 Hz, 2H), 7.59 (td, *J* = 7.7, 1.8 Hz, 1H), 7.30 - 7.20 (m, 3H), 7.15 – 6.76 (m, 1H), 3.48 (t, *J* = 7.3 Hz, 2H), 3.23 (t, *J* = 7.3 Hz, 2H), 2.40 (s, 3H). ^{13}C NMR (100 MHz, Chloroform-*d*) δ 198.9, 160.8, 149.2, 143.7, 136.4, 134.4, 129.2, 128.2, 123.4, 121.2, 37.8, 32.2, 21.6. HRMS (ESI) Calculated for $C_{15}H_{16}NO^{+}$ ($[M+H]^{+}$): 226.1226, found: 226.1228.

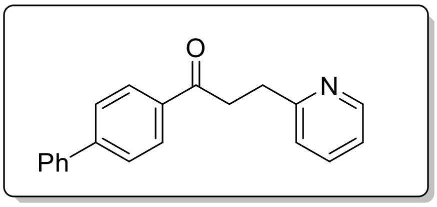

1-([1,1'-biphenyl]-4-yl)-3-(pyridin-2-yl)propan-1-one **3b**

The reaction was carried out according to the general procedure on 0.2 mmol scale (48 h). The residue was purified by flash column chromatography (petroleum ether: ethyl acetate, 10/1-4/1) to afford **3b**, (57%, 32.7 mg), yellowish solid, mp: 80 – 82 °C. ^{1}H NMR (400 MHz, Chloroform-*d*) δ 8.52 (d, *J* = 5.6 Hz, 1H), 8.07 (d, *J* = 10.4 Hz, 2H), 7.74 - 7.52 (m, 6H), 7.46 (t, *J* = 7.4 Hz, 2H), 7.39 (t, *J* = 6.7 Hz, 1H), 7.27 (d, *J* = 9.5 Hz, 1H), 7.16 - 7.05 (m, 1H), 3.54 (t, *J* = 7.3 Hz, 2H), 3.26 (t, *J* = 7.3 Hz, 2H). ^{13}C NMR (100 MHz, Chloroform-*d*) δ 198.9, 160.7, 149.2, 145.7, 139.9, 136.4, 135.6, 128.9, 128.7, 128.2, 127.3, 127.2, 123.4, 121.3, 37.9, 32.1. HRMS (ESI) Calculated for $C_{20}H_{18}NO^{+}$ ($[M+H]^{+}$): 288.1383, found: 288.1382.

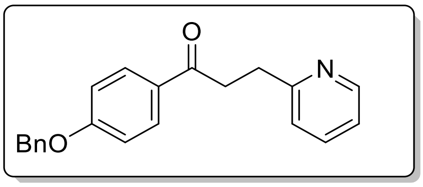

1-(4-(benzyloxy)phenyl)-3-(pyridin-2-yl)propan-1-one **3c**

The reaction was carried out according to the general procedure on 0.2 mmol scale (48 h). The residue was purified by flash column chromatography (petroleum ether: ethyl acetate, 10/1-4/1) to afford **3c**, (48%, 30.4 mg), yellowish solid, mp: 97 − 99 °C. ^{1}H NMR (400 MHz, Chloroform-*d*) δ 8.51 (d, *J* = 4.9 Hz, 1H), 7.97 (d, *J* = 6.9 Hz, 2H), 7.70 - 7.52 (m, 1H), 7.48 - 7.30 (m, 5H), 7.25 (d, *J* = 7.8 Hz, 1H), 7.10 (dd, *J* = 8.5, 4.9 Hz, 1H), 6.99 (d, *J* = 8.9 Hz, 2H), 5.11 (s, 2H), 3.45 (t, *J* = 7.3 Hz, 2H), 3.22 (t, *J* = 7.3

Hz, 2H). ^{13}C NMR (100 MHz, Chloroform-*d*) δ 197.9, 162.6, 160.9, 149.2, 136.4, 136.2, 130.4, 130.2, 128.7, 128.2, 127.5, 123.4, 121.2, 114.5, 70.1, 37.6, 32.2. HRMS (ESI) Calculated for $C_{21}H_{20}NO_2^+$ ($[M+H]^+$): 318.1489, found: 318.1488.

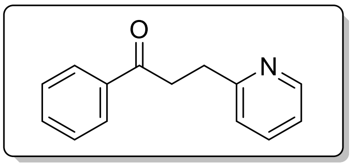

1-phenyl-3-(pyridin-2-yl)propan-1-one **3d**

The reaction was carried out according to the general procedure on 0.2 mmol scale (48 h). The residue was purified by flash column chromatography (petroleum ether: ethyl acetate, 10/1-4/1) to afford **3d**, (78 %, 32.9 mg), yellowish oil. ^{1}H NMR (400 MHz, Chloroform-*d*) δ 8.51 (d, *J* = 5.6 Hz, 1H), 7.98 (d, *J* = 1.4 Hz, 2H), 7.61-7.52 (m, 2H), 7.45 (t, *J* = 7.6 Hz, 2H), 7.26 (d, *J* = 7.6 Hz, 1H), 7.14 – 7.06 (m, 1H), 3.51 (t, *J* = 7.3 Hz, 2H), 3.24 (t, *J* = 7.3 Hz, 2H). ^{13}C NMR (100 MHz, Chloroform-*d*) δ 199.3, 160.7, 149.2, 136.9, 136.4, 133.0, 128.6, 128.1, 123.4, 121.3, 37.8, 32.1. HRMS (ESI) Calculated for $C_{14}H_{14}NO^+$ ($[M+H]^+$): 212.1070, found: 212.1073.

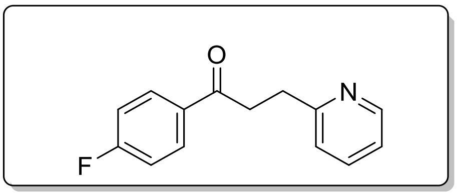

1-(4-fluorophenyl)-3-(pyridin-2-yl)propan-1-one **3e**

The reaction was carried out according to the general procedure on 0.2 mmol scale (48 h). The residue was purified by flash column chromatography (petroleum ether: ethyl acetate, 10/1-4/1) to afford **3e**, (75%, 34.4 mg), yellowish oil. ^{1}H NMR (400 MHz, Chloroform-*d*) δ 8.51 (d, *J* = 4.8 Hz, 1H), 8.12 - 7.87 (m, 2H), 7.60 (t, *J* = 7.7 Hz, 1H), 7.26 (d, *J* = 9.9 Hz, 1H), 7.17 - 7.05 (m, 3H), 3.49 (t, *J* = 7.2 Hz, 2H), 3.23 (t, *J* = 7.2 Hz, 2H). ^{13}C NMR (100 MHz, Chloroform-*d*) δ 197.8, 165.7 (d, *J* = 254.5 Hz), 160.5, 149.2, 136.4, 133.3 (d, *J* = 3.1 Hz), 130.7 (d, *J* = 9.2 Hz), 123.4, 121.3, 115.6 (d, *J* = 21.8 Hz), 37.7, 32.0. HRMS (ESI) Calculated for $C_{14}H_{13}FNO^+$ ($[M+H]^+$): 230.0976, found: 230.0976.

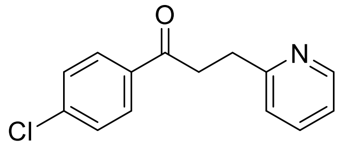

1-(4-chlorophenyl)-3-(pyridin-2-yl)propan-1-one **3f**

The reaction was carried out according to the general procedure on 0.2 mmol scale (48 h). The residue was purified by flash column chromatography (petroleum ether: ethyl acetate, 10/1-4/1) to afford **3f**, (70%, 34.3 mg), yellowish oil. ^{1}H NMR (400 MHz, Chloroform-*d*) δ 8.51 (d, J = 4.8 Hz, 1H), 7.94 (d, J = 9.1 Hz, 2H), 7.68 - 7.51 (m, 1H), 7.42 (d, J = 13.4 Hz, 2H), 7.31 - 7.20 (m, 1H), 7.15 - 7.06 (m, 1H), 3.48 (t, J = 7.2 Hz, 2H), 3.23 (t, J = 7.2 Hz, 2H). ^{13}C NMR (100 MHz, Chloroform-*d*) δ 198.1, 160.4, 149.2, 139.4, 136.4, 135.2, 129.5, 128.9, 123.4, 121.3, 37.7, 31.9. HRMS (ESI) Calculated for $C_{14}H_{13}ClNO^+$ ([M+H]$^+$): 246.0680, found: 246.0681.

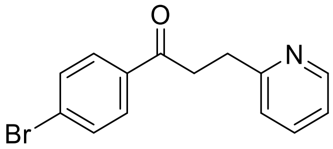

1-(4-bromophenyl)-3-(pyridin-2-yl)propan-1-one **3g**

The reaction was carried out according to the general procedure on 0.2 mmol scale (48 h). The residue was purified by flash column chromatography (petroleum ether: ethyl acetate, 10/1-4/1) to afford **3g**, (62%, 36.0 mg), yellowish oil. ^{1}H NMR (400 MHz, Chloroform-*d*) δ 8.50 (d, J = 5.6 Hz, 1H), 7.86 (d, J = 8.6 Hz, 2H), 7.66 - 7.52 (m, 3H), 7.25 (d, J = 7.8 Hz, 1H), 7.16 - 7.06 (m, 1H), 3.47 (t, J = 7.2 Hz, 2H), 3.23 (t, J = 7.2 Hz, 2H). ^{13}C NMR (100 MHz, Chloroform-*d*) δ 198.3, 160.4, 149.2, 136.4, 135.6, 131.9, 129.6, 128.2, 123.4, 121.3, 37.7, 31.9. HRMS (ESI) Calculated for $C_{14}H_{13}BrNO^+$ ([M+H]$^+$): 290.0175, found: 290.0176.

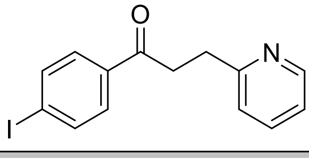

1-(4-iodophenyl)-3-(pyridin-2-yl)propan-1-one **3h**

The reaction was carried out according to the general procedure on 0.2 mmol scale (48

h). The residue was purified by flash column chromatography (petroleum ether: ethyl acetate, 10/1-4/1) to afford **3h**, (52%, 35.1 mg), yellowish oil. ^{1}H NMR (400 MHz, Chloroform-*d*) δ 8.50 (d, *J* = 5.5 Hz, 1H), 7.81 (d, *J* = 10.6 Hz, 2H), 7.70 (d, *J* = 8.6 Hz, 2H), 7.59 (d, *J* = 15.3 Hz, 1H), 7.33 - 7.21 (m, 1H), 7.16 - 7.07 (m, 1H), 3.46 (t, *J* = 7.2 Hz, 2H), 3.23 (t, *J* = 7.2 Hz, 2H). ^{13}C NMR (100 MHz, Chloroform-*d*) δ 198.6, 160.4, 149.2, 137.9, 136.4, 136.2, 129.5, 123.4, 121.3, 101.0, 37.6, 31.9. HRMS (ESI) Calculated for $C_{14}H_{13}INO^{+}$ ($[M+H]^{+}$): 338.0036, found: 338.0037.

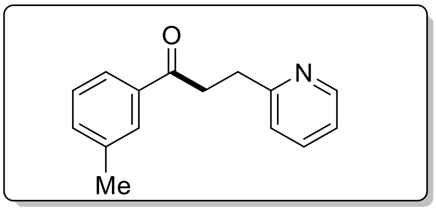

3-(pyridin-2-yl)-1-(*m*-tolyl)propan-1-one **3i**

The reaction was carried out according to the general procedure on 0.2 mmol scale (48 h). The residue was purified by flash column chromatography (petroleum ether: ethyl acetate, 10/1-4/1) to afford **3i**, (78%. 35.1 mg), yellowish oil. ^{1}H NMR (400 MHz, Chloroform-*d*) δ 8.52 (d, *J* = 4.3 Hz, 1H), 7.79 (d, *J* = 7.9 Hz, 2H), 7.58 (d, *J* = 5.9 Hz, 1H), 7.37 - 7.31 (m, 2H), 7.26 (d, *J* = 8.4 Hz, 1H), 7.16 - 7.05 (m, 1H), 3.50 (t, *J* = 7.3 Hz, 2H), 3.23 (t, *J* = 7.3 Hz, 2H), 2.39 (s, 3H). ^{13}C NMR (100 MHz, Chloroform-*d*) δ 199.5, 160.8, 149.2, 138.3, 136.9, 136.4, 133.8, 128.6, 128.4, 125.3, 123.4, 121.2, 37.9, 32.1, 21.4. HRMS (ESI) Calculated for $C_{15}H_{16}NO^{+}$ ($[M+H]^{+}$): 226.1226, found: 226.1228.

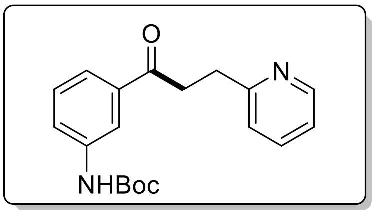

tert-butyl (3-(3-(pyridin-2-yl)propanoyl)phenyl)carbamate **3j**

The reaction was carried out according to the general procedure on 0.2 mmol scale (60 h). The residue was purified by flash column chromatography (petroleum ether: ethyl acetate, 10/1-4/1) to afford **3j**, (74%, 48.4 mg), yellowish solid, mp: 145 − 147 °C. ^{1}H NMR (400 MHz, Chloroform-*d*) δ 8.50 (d, *J* = 4.8 Hz, 1H), 7.91 (s, 1H), 7.72 - 7.56

(m, 3H), 7.35 (t, *J* = 7.9 Hz, 1H), 7.25 (d, *J* = 7.8 Hz, 1H), 7.12 - 7.08 (m, 1H), 6.97 (br s, 1H), 3.48 (t, *J* = 7.3 Hz, 2H), 3.22 (t, *J* = 7.3 Hz, 2H), 1.51 (s, 9H). ^{13}C NMR (100 MHz, Chloroform-*d*) δ 199.0, 160.6, 152.8, 149.1, 139.0, 137.5, 136.5, 129.2, 123.4, 123.0, 122.6, 121.3, 117.9, 80.8, 38.0, 32.0, 28.3. HRMS (ESI) Calculated for $C_{19}H_{23}N_2O_3^+$ ([M+H]$^+$): 327.1703, found: 327.1704.

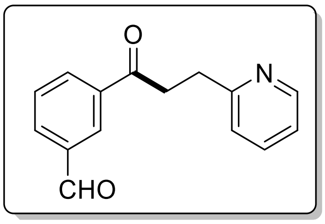

3-(3-(pyridin-2-yl)propanoyl)benzaldehyde **3k**

The reaction was carried out according to the general procedure on 0.2 mmol scale (60 h). The residue was purified by flash column chromatography (petroleum ether: ethyl acetate, 10/1-4/1) to afford **3k**, (61%, 29.2 mg), yellowish oil. ^{1}H NMR (400 MHz, Chloroform-*d*) δ 10.08 (s, 1H), 8.57 - 8.40 (m, 2H), 8.26 (d, *J* = 9.3 Hz, 1H), 8.08 (d, *J* = 7.6 Hz, 1H), 7.69 - 7.54 (m, 2H), 7.32 - 7.22 (m, 1H), 7.12 (dd, *J* = 7.2, 5.1 Hz, 1H), 3.57 (t, *J* = 7.2 Hz, 2H), 3.28 (t, *J* = 7.2 Hz, 2H). ^{13}C NMR (100 MHz, Chloroform-*d*) δ 198.3, 191.5, 160.2, 149.2, 137.7, 136.6, 136.4, 133.6, 133.3, 129.6, 129.5, 123.4, 121.4, 37.8, 31.8. HRMS (ESI) Calculated for $C_{15}H_{14}NO_2^+$ ([M+H]$^+$): 240.1019, found: 240.1018.

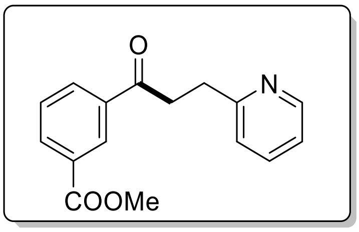

methyl 3-(3-(pyridin-2-yl)propanoyl)benzoate **3l**

The reaction was carried out according to the general procedure on 0.2 mmol scale (60 h). The residue was purified by flash column chromatography (petroleum ether: ethyl acetate, 10/1-4/1) to afford **3l**, (50%, 26.9 mg), yellowish oil. ^{1}H NMR (400 MHz, Chloroform-*d*) δ 8.64 (s, 1H), 8.51 (d, *J* = 5.7 Hz, 1H), 8.23 - 8.17 (m, 2H), 7.66- 7.49 (m, 2H), 7.27 (d, *J* = 8.6 Hz, 1H), 7.11 (dd, *J* = 7.5, 4.9 Hz,1H), 3.95 (s, 3H), 3.56 (t, *J* = 7.2 Hz, 2H), 3.26 (t, *J* = 7.2 Hz, 2H). ^{13}C NMR (100 MHz, Chloroform-*d*) δ 198.5,

166.3, 160.4, 149.2, 137.1, 136.4, 133.8, 132.2, 130.7, 129.2, 128.8, 123.4, 121.3, 52.4, 37.8, 31.9. HRMS (ESI) Calculated for $C_{16}H_{16}NO_3^+$ ($[M+H]^+$): 270.1125, found: 270.1126.

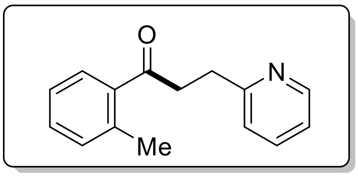

3-(pyridin-2-yl)-1-(*o*-tolyl)propan-1-one **3m**

The reaction was carried out according to the general procedure on 0.2 mmol scale (48 h). The residue was purified by flash column chromatography (petroleum ether: ethyl acetate, 10/1-4/1) to afford **3m**, (70%, 31.5 mg), yellowish oil. ^{1}H NMR (400 MHz, Chloroform-*d*) δ 8.50 (d, 1H, 4.1 Hz), 7.69 (d, *J* = 7.7 Hz, 1H), 7.59 (td, *J* = 7.7, 1.8 Hz, 1H), 7.34 (d, *J* = 8.7 Hz, 1H), 7.23 (t, *J* = 7.4 Hz, 3H), 7.10 (t, *J* = 7.6 Hz, 1H), 3.41 (t, *J* = 7.1 Hz, 2H), 3.22 (t, *J* = 7.2 Hz, 2H), 2.46 (s, 3H). ^{13}C NMR (100 MHz, Chloroform-*d*) δ 203.4, 160.6, 149.2, 138.0 (2C), 136.4, 131.8, 131.2, 128.5, 125.6, 123.3, 121.2, 40.6, 32.2, 21.2. HRMS (ESI) Calculated for $C_{15}H_{16}NO^+$ ($[M+H]^+$): 226.1226, found: 226.1227.

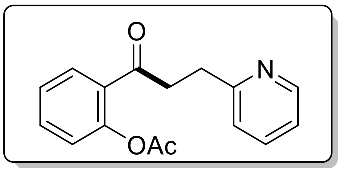

2-(3-(pyridin-2-yl)propanoyl)phenyl acetate **3n**

The reaction was carried out according to the general procedure on 0.2 mmol scale (48 h). The residue was purified by flash column chromatography (petroleum ether: ethyl acetate, 10/1-4/1) to afford **3n**, (64%, 34.4 mg), yellowish oil. ^{1}H NMR (400 MHz, Chloroform-*d*) δ 8.50 (d, *J* = 5.5 Hz, 1H), 7.84 (dd, *J* = 7.8, 1.6 Hz, 1H), 7.65 - 7.45 (m, 2H), 7.35 - 7.28 (m, 1H), 7.22 (d, *J* = 7.8 Hz, 1H), 7.12 - 7.08 (m, 1H), 3.41 (t, *J* = 7.3 Hz, 2H), 3.19 (t, *J* = 7.3 Hz, 2H), 2.32 (s, 3H). ^{13}C NMR (100 MHz, Chloroform-*d*) δ 199.3, 169.6, 160.4, 149.2, 148.6, 136.3, 133.1, 130.9, 129.9, 126.0, 123.8, 123.3, 121.3, 40.5, 32.0, 21.2. HRMS (ESI) Calculated for $C_{16}H_{16}NO_3^+$ ($[M+H]^+$): 270.1125, found: 270.1127.

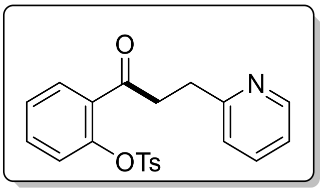

2-(3-(pyridin-2-yl)propanoyl)phenyl 4-methylbenzenesulfonate **3o**

The reaction was carried out according to the general procedure on 0.2 mmol scale (48 h). The residue was purified by flash column chromatography (petroleum ether: ethyl acetate, 10/1-4/1) to afford **3o**, (52%, 39.6 mg), yellowish oil. ^{1}H NMR (400 MHz, Chloroform-*d*) δ 8.48 (d, *J* = 4.2 Hz, 1H), 7.68 (d, *J* = 8.3 Hz, 2H), 7.63 - 7.53 (m, 2H), 7.41 (td, *J* = 7.8, 1.8 Hz, 1H), 7.30 (dd, *J* = 15.8, 8.2 Hz, 3H), 7.20 (d, *J* = 7.8 Hz, 1H), 7.15 - 7.04 (m, 2H), 3.32 (t, *J* = 7.3 Hz, 2H), 3.13 (t, *J* = 7.3 Hz, 2H), 2.43 (s, 3H). ^{13}C NMR (100 MHz, Chloroform-*d*) δ 199.8, 160.4, 149.1, 146.6, 145.8, 136.3, 134.0, 132.4, 132.0, 130.0, 129.9, 128.6, 127.2, 123.4, 123.2, 121.2, 41.6, 32.1, 21.8. HRMS (ESI) Calculated for $C_{21}H_{20}NO_4S^+$ ([M+H]$^+$): 382.1108, found: 382.1109.

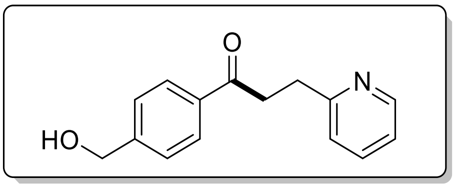

1-(4-(hydroxymethyl)phenyl)-3-(pyridin-2-yl)propan-1-one **3p**

The reaction was carried out according to the general procedure on 0.2 mmol scale (48 h). The residue was purified by flash column chromatography (petroleum ether: ethyl acetate, 10/1-4/1) to afford **3p**, (68%, 32.8 mg), yellowish solid, mp: 112−114 °C. ^{1}H NMR (400 MHz, Chloroform-*d*) δ 8.48 (d, *J* = 4.9 Hz, 1H), 7.90 (d, *J* = 8.3 Hz, 2H), 7.61 (td, *J* = 7.7, 1.8 Hz, 1H), 7.41 (d, *J* = 8.4 Hz, 2H), 7.26 (d, *J* = 8.3 Hz, 1H), 7.17 - 7.07 (m, 1H), 4.76 (s, 2H), 3.41 (t, *J* = 7.4 Hz, 2H), 3.20 (t, *J* = 7.3 Hz, 2H). ^{13}C NMR (100 MHz, Chloroform-*d*) δ 198.8, 160.7, 149.0, 146.6, 136.6, 135.8, 128.3, 126.5, 123.5, 121.4, 64.3, 38.0, 32.0. HRMS (ESI) Calculated for $C_{15}H_{16}NO_2^+$ ([M+H]$^+$): 242.1176, found: 242.1176.

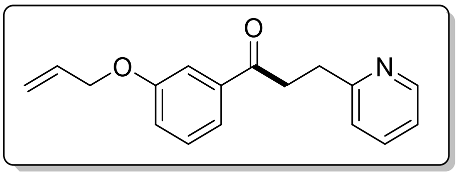

1-(3-(allyloxy)phenyl)-3-(pyridin-2-yl)propan-1-one **3q**

The reaction was carried out according to the general procedure on 0.2 mmol scale (48 h). The residue was purified by flash column chromatography (petroleum ether: ethyl acetate, 10/1-4/1) to afford **3q**, (65%, 34.7 mg), yellowish oil. ^{1}H NMR (400 MHz, Chloroform-*d*) δ 8.51 (d, *J* = 4.8 Hz, 1H), 7.65 - 7.56 (m, 2H), 7.54 - 7.51 (m, 1H), 7.35 (t, *J* = 7.9 Hz, 1H), 7.26 (d, *J* = 9.0 Hz, 1H), 7.11 (dd, *J* = 9.5, 3.2 Hz, 2H), 6.14 - 5.96 (m, 1H), 5.42 (dd, *J* = 17.3, 1.5 Hz, 1H), 5.30 (dd, *J* = 10.5, 1.3 Hz, 1H), 4.58 (dt, *J* = 5.3, 1.4 Hz, 2H), 3.49 (t, *J* = 7.2 Hz, 2H), 3.23 (t, *J* = 7.2 Hz, 2H). ^{13}C NMR (100 MHz, Chloroform-*d*) δ 199.1, 160.7, 158.8, 149.2, 138.2, 136.4, 132.9, 129.6, 123.4, 121.2, 120.9, 120.2, 117.9, 113.2, 68.9, 37.9, 32.1. HRMS (ESI) Calculated for $C_{17}H_{18}NO_2^+$ ($[M+H]^+$): 268.1332, found: 268.1334.

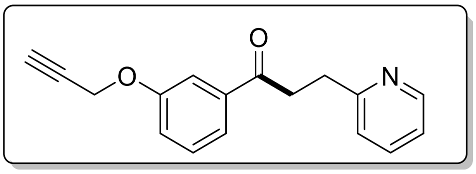

1-(3-(prop-2-yn-1-yloxy)phenyl)-3-(pyridin-2-yl)propan-1-one **3r**

The reaction was carried out according to the general procedure on 0.2 mmol scale (48 h). The residue was purified by flash column chromatography (petroleum ether: ethyl acetate, 10/1-4/1) to afford **3r**, (71%, 37.6 mg), yellowish oil. ^{1}H NMR (400 MHz, Chloroform-*d*) δ 8.51 (d, *J* = 5.7 Hz, 1H), 7.70 - 7.53 (m, 3H), 7.38 (t, *J* = 7.9 Hz, 1H), 7.26 (d, *J* = 9.0 Hz, 1H), 7.17 (dd, *J* = 9.1, 2.7 Hz, 1H), 7.11 (dd, *J* = 7.9, 5.4 Hz, 1H), 4.73 (d, *J* = 2.4 Hz, 2H), 3.50 (t, *J* = 7.2 Hz, 2H), 3.23 (t, *J* = 7.2 Hz, 2H), 2.54 (t, *J* = 2.4 Hz, 1H). ^{13}C NMR (100 MHz, Chloroform-*d*) δ 198.9, 160.6, 157.7, 149.2, 138.3, 136.4, 129.7, 123.4, 121.6, 121.3, 120.3, 113.5, 78.1, 75.9, 56.0, 37.9, 32.0. HRMS (ESI) Calculated for $C_{17}H_{16}NO_2^+$ ($[M+H]^+$): 266.1176, found: 266.1177.

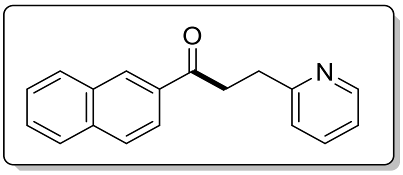

1-(naphthalen-2-yl)-3-(pyridin-2-yl)propan-1-one **3s**

The reaction was carried out according to the general procedure on 0.2 mmol scale (48 h). The residue was purified by flash column chromatography (petroleum ether: ethyl acetate, 10/1-4/1) to afford **3s**, (55%, 28.7 mg), yellowish oil. ^{1}H NMR (400 MHz,

Chloroform-*d*) δ 8.54 (d, *J* = 6.4 Hz, 2H), 8.06 (dd, *J* = 8.6, 1.7 Hz, 1H), 7.95 (d, *J* = 8.0 Hz, 1H), 7.90 - 7.82 (m, 2H), 7.66 - 7.49 (m, 3H), 7.29 (d, *J* = 7.8 Hz, 1H), 7.12 (dd, *J* = 7.8, 5.4 Hz, 1H), 3.65 (t, *J* = 7.3 Hz, 2H), 3.31 (t, *J* = 7.3 Hz, 2H). ^{13}C NMR (100 MHz, Chloroform-*d*) δ 199.3, 160.8, 149.2, 136.4, 135.6, 134.2, 132.5, 129.8, 129.6, 128.4, 127.8, 126.7, 123.9, 123.5, 121.3, 37.9, 32.2. HRMS (ESI) Calculated for $C_{18}H_{16}NO^+$ ([M+H]$^+$): 262.1226, found: 262.1228.

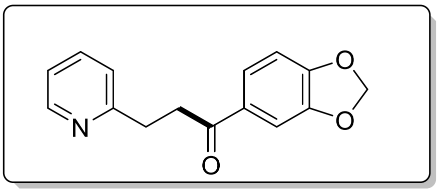

1-(benzo[*d*][1,3]dioxol-5-yl)-3-(pyridin-2-yl)propan-1-one **3t**

The reaction was carried out according to the general procedure on 0.2 mmol scale (48 h). The residue was purified by flash column chromatography (petroleum ether: ethyl acetate, 10/1-4/1) to afford **3t**, (62%, 31.6 mg), yellowish oil. ^{1}H NMR (400 MHz, Chloroform-*d*) δ 8.51 (d, *J* = 4.8 Hz, 1H), 7.74 - 7.50 (m, 2H), 7.46 (d, *J* = 1.7 Hz, 1H), 7.26 (t, *J* = 7.8 Hz, 1H), 7.11 (d, *J* = 5.1 Hz, 1H), 6.83 (d, *J* = 8.2 Hz, 1H), 6.02 (s, 2H), 3.42 (t, *J* = 7.3 Hz, 2H), 3.21 (t, *J* = 7.3 Hz, 2H). ^{13}C NMR (100 MHz, Chloroform-*d*) δ 197.4, 160.8, 151.7, 149.2, 148.1, 136.5, 131.8, 124.3, 123.4, 121.2, 107.9, 107.8, 101.8, 37.6, 32.4. HRMS (ESI) Calculated for $C_{15}H_{14}NO_3^+$ ([M+H]$^+$): 256.0968, found: 256.0969.

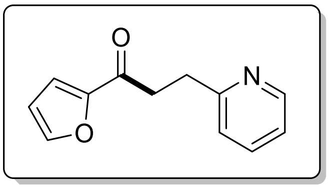

1-(furan-2-yl)-3-(pyridin-2-yl)propan-1-one **3u**

The reaction was carried out according to the general procedure on 0.2 mmol scale (36 h). The residue was purified by flash column chromatography (petroleum ether: ethyl acetate, 10/1-4/1) to afford **3u**, (64%, 25.7 mg), yellowish oil. ^{1}H NMR (400 MHz, Chloroform-*d*) δ 8.51 (d, *J* = 4.8 Hz, 1H), 7.59 (dd, *J* = 15.3, 1.8 Hz, 2H), 7.34 - 7.17 (m, 2H), 7.11 (dd, *J* = 6.9, 5.4 Hz, 1H), 6.51 (dd, *J* = 3.6, 1.7 Hz, 1H), 3.35 (t, *J* = 7.3 Hz, 2H), 3.22 (t, *J* = 7.3 Hz, 2H). ^{13}C NMR (100 MHz, Chloroform-*d*) δ 188.5, 160.4, 152.6, 149.2, 146.3, 136.4, 123.3, 121.3, 117.1, 112.1, 37.6, 31.8. HRMS (ESI)

Calculated for $C_{12}H_{12}NO_2^+$ ($[M+H]^+$): 202.0863, found: 202.0864.

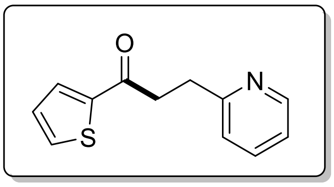

3-(pyridin-2-yl)-1-(thiophen-2-yl)propan-1-one **3v**

The reaction was carried out according to the general procedure on 0.2 mmol scale (36 h). The residue was purified by flash column chromatography (petroleum ether: ethyl acetate, 10/1-4/1) to afford **3v**, (68%, 29.5 mg), yellowish oil. ^1H NMR (400 MHz, Chloroform-*d*) δ 8.52 (d, J = 5.6 Hz, 1H), 7.75 (d, J = 4.9 Hz, 1H), 7.65 - 7.52 (m, 2H), 7.31 - 7.20 (m, 1H), 7.11 (dd, J = 5.0, 3.8 Hz, 2H), 3.45 (t, J = 7.3 Hz, 2H), 3.23 (t, J = 7.3 Hz, 2H). ^{13}C NMR (100 MHz, Chloroform-*d*) δ 192.3, 160.4, 149.2, 144.2, 136.4, 133.4, 132.0, 128.1, 123.4, 121.3, 38.5, 32.20. HRMS (ESI) Calculated for $C_{12}H_{12}NOS^+$ ($[M+H]^+$): 218.0634, found: 218.0635.

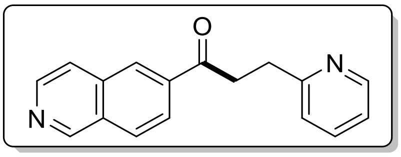

1-(isoquinolin-6-yl)-3-(pyridin-2-yl)propan-1-one **3w**

The reaction was carried out according to the general procedure on 0.2 mmol scale (48 h). The residue was purified by flash column chromatography (petroleum ether: ethyl acetate, 10/1-4/1) to afford **3w**, (38%, 19.9 mg), yellowish oil. ^1H NMR (400 MHz, Chloroform-*d*) δ 9.01 (dd, J = 4.2, 1.7 Hz, 1H), 8.58 - 8.48 (m, 2H), 8.36 - 8.23 (m, 2H), 8.15 (d, J = 8.9 Hz, 1H), 7.61 (td, J = 7.7, 1.8 Hz, 1H), 7.48 (dd, J = 8.3, 4.2 Hz, 1H), 7.29 (d, J = 7.8 Hz, 1H), 7.18 - 7.07 (m, 1H), 3.66 (t, J = 7.2 Hz, 2H), 3.31 (t, J = 7.2 Hz, 2H). ^{13}C NMR (100 MHz, Chloroform-*d*) δ 198.7, 160.5, 152.6, 150.1, 149.2, 137.6, 136.5, 134.7, 130.0, 129.6, 127.7, 127.5, 123.5, 121.9, 121.4, 37.9, 32.1. HRMS (ESI) Calculated for $C_{17}H_{15}N_2O^+$ ($[M+H]^+$): 263.1179, found: 263.1181.

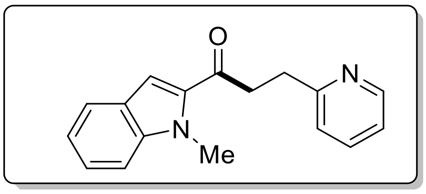

1-(1-methyl-1*H*-indol-2-yl)-3-(pyridin-2-yl)propan-1-one **3x**

The reaction was carried out according to the general procedure on 0.2 mmol scale (36 h). The residue was purified by flash column chromatography (petroleum ether: ethyl acetate, 10/1-4/1) to afford **3x**, (59%, 31.2 mg), yellowish oil. ^{1}H NMR (400 MHz, Chloroform-*d*) δ 8.53 (d, *J* = 4.9 Hz, 1H), 7.66 (d, *J* = 8.0 Hz, 1H), 7.58 (td, *J* = 7.7, 1.8 Hz, 1H), 7.35 (d, *J* = 5.2 Hz, 3H), 7.24 (d, *J* = 7.8 Hz, 1H), 7.18 - 6.96 (m, 2H), 4.05 (s, 3H), 3.50 (t, *J* = 7.4 Hz, 2H), 3.24 (t, *J* = 7.4 Hz, 2H). ^{13}C NMR (100 MHz, Chloroform-*d*) δ 193.3, 160.7, 149.3, 140.0, 136.4, 134.7, 125.8, 123.3, 122.9, 121.3, 120.7, 111.4, 110.3, 39.0, 32.6, 32.2. HRMS (ESI) Calculated for $C_{17}H_{17}N_2O^+$ ([M+H]$^+$): 265.1335, found: 265.1338.

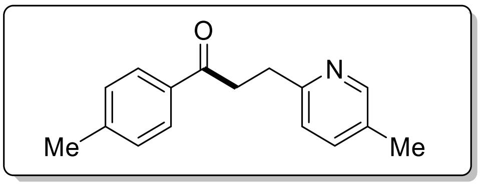

3-(5-methylpyridin-2-yl)-1-(*p*-tolyl)propan-1-one **3y**

The reaction was carried out according to the general procedure on 0.2 mmol scale (48 h). The residue was purified by flash column chromatography (petroleum ether: ethyl acetate, 10/1-4/1) to afford **3y**, (64%, 30.6 mg), yellowish solid, mp: 61 − 63 °C. ^{1}H NMR (400 MHz, Chloroform-*d*) δ 8.34 (s, 1H), 7.89 (d, *J* = 8.1 Hz, 2H), 7.40 (d, *J* = 6.3 Hz, 1H), 7.24 (d, *J* = 8.0 Hz, 2H), 7.14 (d, *J* = 7.9 Hz, 1H), 3.45 (t, *J* = 7.3 Hz, 2H), 3.18 (t, *J* = 7.3 Hz, 2H), 2.40 (s, 3H), 2.28 (s, 3H). ^{13}C NMR (100 MHz, Chloroform-*d*) δ 199.1, 157.8, 149.5, 143.7, 137.0, 134.4, 130.5, 129.2, 128.2, 122.8, 38.0, 31.7, 21.6, 18.0. HRMS (ESI) Calculated for $C_{16}H_{18}NO^+$ ([M+H]$^+$): 240.1383, found: 240.1384.

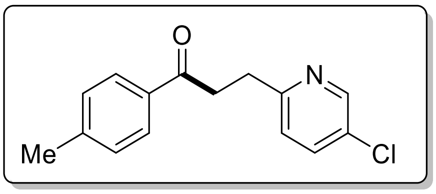

3-(5-chloropyridin-2-yl)-1-(*p*-tolyl)propan-1-one **3z**

The reaction was carried out according to the general procedure on 0.2 mmol scale (48 h). The residue was purified by flash column chromatography (petroleum ether: ethyl acetate, 10/1-4/1) to afford **3z**, (75%, 38.9 mg), yellowish solid, mp: 82 - 84 °C. ^{1}H NMR (400 MHz, Chloroform-*d*) δ 8.45 (s, 1H), 7.88 (d, *J* = 8.1 Hz, 2H), 7.56 (dd, *J* =

8.3, 2.3 Hz, 1H), 7.23 (t, J = 8.4 Hz, 3H), 3.46 (t, J = 7.1 Hz, 2H), 3.20 (t, J = 7.1 Hz, 2H), 2.40 (s, 3H). ^{13}C NMR (100 MHz, Chloroform-*d*) δ 198.7, 159.0, 148.0, 143.9, 136.0, 134.3, 129.5, 129.3, 128.2, 124.2, 37.4, 31.4, 21.6. HRMS (ESI) Calculated for $C_{15}H_{15}ClNO^+$ ($[M+H]^+$): 260.0837, found: 260.0838.

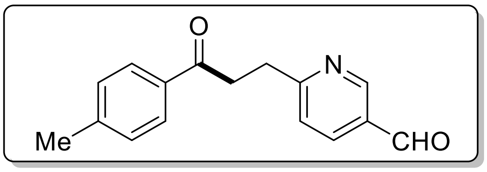

6-(3-oxo-3-(*p*-tolyl)propyl)nicotinaldehyde **3aa**

The reaction was carried out according to the general procedure on 0.2 mmol scale (48 h). The residue was purified by flash column chromatography (petroleum ether: ethyl acetate, 10/1-4/1) to afford **3aa**, (86%, 43.5 mg), yellowish solid, mp: 115 − 117 °C. ^{1}H NMR (400 MHz, Chloroform-*d*) δ 10.06 (s, 1H), 8.95 (s, 1H), 8.08 (dd, J = 8.0, 2.2 Hz, 1H), 7.89 (d, J = 8.2 Hz, 2H), 7.46 (d, J = 8.0 Hz, 1H), 7.26 (d, J = 8.7 Hz, 2H), 3.55 (t, J = 7.0 Hz, 2H), 3.33 (t, J = 7.0 Hz, 2H), 2.41 (s, 3H). ^{13}C NMR (100 MHz, Chloroform-*d*) δ 198.4, 190.5, 151.9, 144.0, 136.0, 134.2, 129.60, 129.3, 128.2, 124.0, 37.0, 32.4, 21.7. HRMS (ESI) Calculated for $C_{16}H_{16}NO_2^+$ ($[M+H]^+$): 254.1176, found: 254.1177.

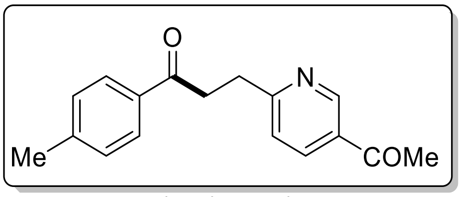

3-(5-acetylpyridin-2-yl)-1-(*p*-tolyl)propan-1-one **3bb**

The reaction was carried out according to the general procedure on 0.2 mmol scale (48 h). The residue was purified by flash column chromatography (petroleum ether: ethyl acetate, 10/1-4/1) to afford **3bb**, (82%, 43.9 mg), yellowish solid, mp: 78 − 80 °C. ^{1}H NMR (400 MHz, Chloroform-*d*) δ 9.06 (d, J = 1.9 Hz, 1H), 8.15 (dd, J = 8.1, 2.3 Hz, 1H), 7.89 (d, J = 8.2 Hz, 2H), 7.39 (d, J = 8.1 Hz, 1H), 7.25 (d, J = 8.0 Hz, 2H), 3.53 (t, J = 7.0 Hz, 2H), 3.31 (t, J = 7.0 Hz, 2H), 2.60 (s, 3H), 2.40 (s, 3H). ^{13}C NMR (100 MHz, Chloroform-*d*) δ 198.5, 196.6, 165.7, 149.6, 144.0, 135.9, 134.3, 130.3, 129.3, 128.2, 123.5, 37.1, 32.1, 26.7, 21.7. HRMS (ESI) Calculated for $C_{17}H_{18}NO_2^+$ ($[M+H]^+$): 268.1332, found: 268.1334.

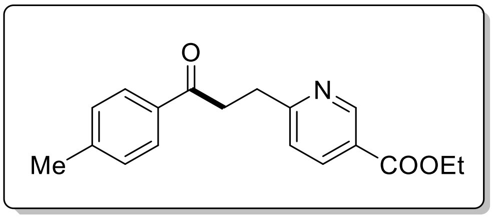

ethyl 6-(3-oxo-3-(*p*-tolyl)propyl)nicotinate **3cc**

The reaction was carried out on 5 mmol scale. To a 100 mL Schlenk tube equipped with a magnetic stir bar was added carboxylic acid (5.0 mmol, 1 equiv.), photocatalyst $Ir[dF(CF_3)ppy]_2(dtbbpy)PF_6$ (56.1 mg, 1 mol%), K_2HPO_4 (174 mg, 20 mol%), and Ph_3P (1.57 g, 1.2 equiv.) and the tube was evacuated and backfilled with Ar (three times). The ethyl 6-vinylnicotinate **2f** (1.33 g, 7.5 mmol, 1.5 equiv.) in DCM/H_2O (50 mL, 4:1 v/v) were added by syringe under Ar. The tube was then sealed and was placed at a distance (app. 5 cm) from 5 W blue LEDs lamp, and the mixture was stirred for 60 h at room temperature. After completion, the mixture was quenched with water and extracted with DCM (3 x 40 mL). The combined organic layer was dried over anhydrous Na_2SO_4, then the solvent was removed under vacuo. The residue was purified with chromatography column on silica gel (gradient eluent of petroleum ether/EtOAc: 20/1 to 4/1) to give ethyl 6-(3-oxo-3-(p-tolyl)propyl)nicotinate **3cc** (1.14 g, 77%) as yellowish solid, mp: 79 − 81 °C. ^{1}H NMR (400 MHz, Chloroform-*d*) δ 9.11 (s, 1H), 8.19 (d, J = 10.3 Hz, 1H), 7.89 (d, J = 8.2 Hz, 2H), 7.35 (d, J = 8.1 Hz, 1H), 7.29 - 7.19 (m, 2H), 4.39 (q, J = 7.1 Hz, 2H), 3.51 (t, J = 7.1 Hz, 2H), 3.29 (t, J = 7.1 Hz, 2H), 2.40 (s, 3H), 1.39 (t, J = 7.1 Hz, 3H). ^{13}C NMR (100 MHz, Chloroform-*d*) δ 198.5, 165.4, 150.5, 143.9, 137.3, 134.3, 129.3, 128.2, 124.0, 123.1, 61.2, 37.2, 32.2, 21.6, 14.3. HRMS (ESI) Calculated for $C_{16}H_{18}NO^+$ ($[M+H]^+$): 298.1438, found: 298.1439.

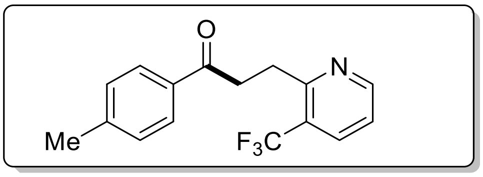

1-(*p*-tolyl)-3-(3-(trifluoromethyl)pyridin-2-yl)propan-1-one **3dd**

The reaction was carried out according to the general procedure on 0.2 mmol scale (48 h). The residue was purified by flash column chromatography (petroleum ether: ethyl

acetate, 10/1-4/1) to afford **3dd**, (84%, 49.2 mg), yellowish solid, mp: 90 − 92 °C. ^{1}H NMR (400 MHz, Chloroform-*d*) δ 8.63 (d, *J* = 4.6 Hz, 1H), 8.15 - 7.81 (m, 3H), 7.27 - 7.21 (m, 3H), 3.52 (t, *J* = 8.3 Hz, 2H), 3.44 (t, *J* = 8.3 Hz, 2H), 2.41 (s, 3H). ^{13}C NMR (100 MHz, Chloroform-*d*) δ 198.5, 159.1, 151.7, 143.7, 134.5, 133.9 (q, *J* = 5.3 Hz), 129.2, 128.2, 124.7 (q, *J* = 31.6 Hz), 124.0 (q, *J* = 272.3 Hz), 120.7, 36.5, 29.1, 21.6. HRMS (ESI) Calculated for $C_{16}H_{15}F_3NO^+$ ($[M+H]^+$): 294.1100, found: 294.1102.

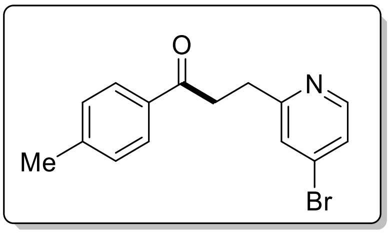

3-(4-bromopyridin-2-yl)-1-(*p*-tolyl)propan-1-one **3ee**

The reaction was carried out according to the general procedure on 0.2 mmol scale (48 h). The residue was purified by flash column chromatography (petroleum ether: ethyl acetate, 10/1-4/1) to afford **3ee**, (73%, 44.2 mg), yellowish oil. ^{1}H NMR (400 MHz, Chloroform-*d*) δ 8.31 (d, *J* = 5.3 Hz, 1H), 7.89 (d, *J* = 8.2 Hz, 2H), 7.46 (s, 1H), 7.26 (dd, *J* = 13.6, 7.6 Hz, 3H), 3.47 (t, *J* = 7.2 Hz, 2H), 3.20 (t, *J* = 7.2 Hz, 2H), 2.40 (s, 3H). ^{13}C NMR (100 MHz, Chloroform-*d*) δ 198.5, 162.5, 149.9, 143.9, 134.3, 133.0, 129.3, 128.2, 126.7, 124.6, 37.3, 31.8, 21.7. HRMS (ESI) Calculated for $C_{15}H_{15}BrNO^+$ ($[M+H]^+$): 304.0332, found: 304.0334.

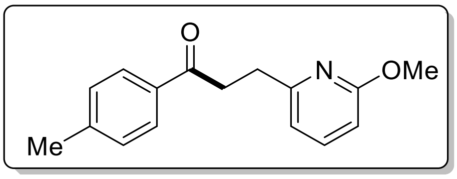

3-(6-methoxypyridin-2-yl)-1-(*p*-tolyl)propan-1-one **3ff**

The reaction was carried out according to the general procedure on 0.2 mmol scale (48 h). The residue was purified by flash column chromatography (petroleum ether: ethyl acetate, 10/1-4/1) to afford **3ff**, (50%, 25.5 mg), yellowish oil. ^{1}H NMR (400 MHz, Chloroform-*d*) δ 7.91 (d, *J* = 8.2 Hz, 2H), 7.46 (t, *J* = 7.7 Hz, 1H), 7.25 (d, *J* = 8.4 Hz, 2H), 6.79 (d, *J* = 7.2 Hz, 1H), 6.53 (d, *J* = 8.2 Hz, 1H), 3.82 (s, 3H), 3.44 (t, *J* = 7.2 Hz, 2H), 3.16 (t, *J* = 7.2 Hz, 2H), 2.41 (s, 3H). ^{13}C NMR (100 MHz, Chloroform-*d*) δ 199.3, 163.7, 158.4, 143.7, 138.8, 134.6, 129.2, 128.2, 115.5, 107.6, 53.1, 37.2, 31.8,

21.6. HRMS (ESI) Calculated for $C_{16}H_{18}NO_2^+$ ($[M+H]^+$): 256.1332, found: 256.1335.

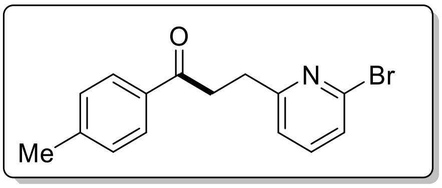

3-(6-bromopyridin-2-yl)-1-(*p*-tolyl)propan-1-one **3gg**

The reaction was carried out according to the general procedure on 0.2 mmol scale (48 h). The residue was purified by flash column chromatography (petroleum ether: ethyl acetate, 10/1-4/1) to afford **3gg**, (42%, 25.5 mg), yellowish solid, mp: 76 − 78 °C. ^{1}H NMR (400 MHz, Chloroform-*d*) δ 7.88 (d, *J* = 8.1 Hz, 2H), 7.44 (t, *J* = 7.7 Hz, 1H), 7.34 – 7.19 (m, 4H), 3.46 (t, *J* = 7.1 Hz, 2H), 3.20 (t, *J* = 7.1 Hz, 2H), 2.40 (s, 3H).^{13}C NMR (100 MHz, Chloroform-*d*) δ 198.6, 162.6, 143.9, 141.6, 138.7, 134.3, 129.3, 128.2, 125.6, 122.3, 37.6, 31.8, 21.7. HRMS (ESI) Calculated for $C_{15}H_{15}BrNO^+$ ($[M+H]^+$): 304.0332, found: 304.0333.

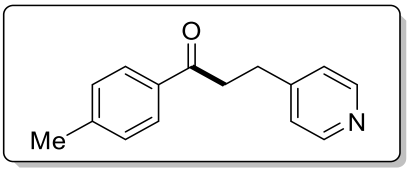

3-(pyridin-4-yl)-1-(*p*-tolyl)propan-1-one **3hh**

The reaction was carried out according to the general procedure on 0.2 mmol scale (48 h). The residue was purified by flash column chromatography (petroleum ether: ethyl acetate, 10/1-4/1) to afford **3hh**, (68%, 30.6 mg), yellowish oil. ^{1}H NMR (400 MHz, Chloroform-*d*) δ 8.52 (d, *J* = 5.9 Hz, 2H), 7.85 (d, *J* = 8.2 Hz, 2H), 7.27 (d, *J* = 8.2 Hz, 2H), 7.20 (d, *J* = 5.9 Hz, 2H), 3.30 (t, *J* = 7.4 Hz, 2H), 3.08 (t, *J* = 7.4 Hz, 2H), 2.41 (s, 3H). ^{13}C NMR (100 MHz, Chloroform-*d*) δ 197.9, 150.7, 149.6, 144.2, 134.1, 129.4, 128.1, 124.0, 38.7, 29.3, 21.7. HRMS (ESI) Calculated for $C_{15}H_{16}NO^+$ ($[M+H]^+$): 226.1226, found: 226.1228.

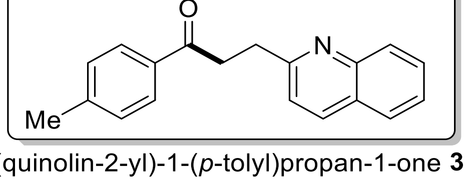

3-(quinolin-2-yl)-1-(*p*-tolyl)propan-1-one **3ii**

The reaction was carried out according to the general procedure on 0.2 mmol scale (48

h). The residue was purified by flash column chromatography (petroleum ether: ethyl acetate, 10/1-4/1) to afford **3ii**, (52%, 28.6 mg), yellowish solid, mp: 110 – 112 °C. ^{1}H NMR (400 MHz, Chloroform-*d*) δ 8.06 (d, *J* = 8.4 Hz, 1H), 7.98 (d, *J* = 8.5 Hz, 1H), 7.93 (d, *J* = 8.2 Hz, 2H), 7.77 (d, *J* = 8.1 Hz, 1H), 7.69 – 7.62 (m, 1H), 7.47 (t, *J* = 7.5 Hz, 1H), 7.39 (d, *J* = 8.4 Hz, 1H), 7.25 (d, *J* = 7.5 Hz, 2H), 3.60 (t, *J* = 7.2 Hz, 2H), 3.43 (t, *J* = 7.1 Hz, 2H), 2.40 (s, 3H). ^{13}C NMR (100 MHz, Chloroform-*d*) δ 161.3, 147.9, 143.7, 136.2, 134.6, 129.3, 129.2, 128.8, 128.3, 127.5, 126.8, 125.8, 122.0, 37.5, 32.8, 21.7. HRMS (ESI) Calculated for $C_{19}H_{18}NO^+$ ($[M+H]^+$): 276.1383, found: 276.1385.

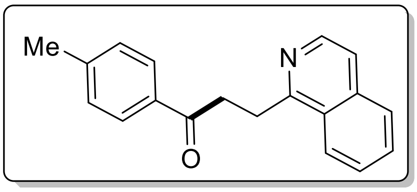

3-(isoquinolin-1-yl)-1-(*p*-tolyl)propan-1-one **3jj**

The reaction was carried out according to the general procedure on 0.2 mmol scale (60 h). The residue was purified by flash column chromatography (petroleum ether: ethyl acetate, 10/1-4/1) to afford **3jj**, (49%, 27.0 mg), yellowish solid, mp: 105 − 107 °C ^{1}H NMR (400 MHz, Chloroform-*d*) δ 8.40 (d, *J* = 5.7 Hz, 1H), 8.26 (d, *J* = 8.3 Hz, 1H), 7.96 (d, *J* = 9.6 Hz, 2H), 7.81 (d, *J* = 8.1 Hz, 1H), 7.64 (dt, *J* = 24.8, 7.3 Hz, 2H), 7.51 (d, *J* = 4.9 Hz, 1H), 7.26 (d, *J* = 6.9 Hz, 2H), 3.76 (t, *J* = 6.8 Hz, 2H), 3.65 (t, *J* = 6.8 Hz, 2H), 2.41 (s, 3H). ^{13}C NMR (100 MHz, Chloroform-*d*) δ 199.3, 160.2, 143.7, 141.7, 136.1, 134.6, 129.9, 129.2, 128.3, 127.4, 127.2, 127.1, 125.1, 119.4, 36.6, 28.6, 21.7. HRMS (ESI) Calculated for $C_{19}H_{18}NO^+$ ($[M+H]^+$): 276.1383, found: 276.1384.

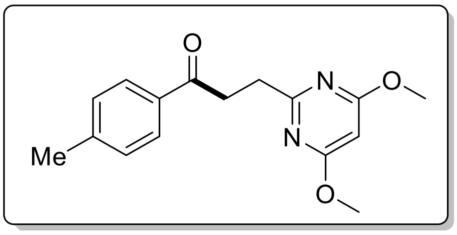

3-(4,6-dimethoxypyrimidin-2-yl)-1-(*p*-tolyl)propan-1-one **3kk**

The reaction was carried out according to the general procedure on 0.2 mmol scale (48 h). The residue was purified by flash column chromatography (petroleum ether: ethyl acetate, 10/1-4/1) to afford **3kk**, (87%, 49.7 mg), yellowish oil. ^{1}H NMR (400 MHz,

Chloroform-*d*) δ 7.93 (d, *J* = 8.2 Hz, 2H), 7.26 (d, *J* = 7.7 Hz, 2H), 5.83 (s, 1H), 3.82 (s, 6H), 3.47 (t, *J* = 6.9 Hz, 2H), 3.26 (t, *J* = 6.9 Hz, 2H), 2.41 (s, 3H). ^{13}C NMR (100 MHz, Chloroform-d) δ 199.0, 171.3, 169.1, 143.6, 134.7, 129.2, 128.2, 86.9, 53.8, 35.3, 32.9, 21.6. ^{13}C NMR (100 MHz, Chloroform-d) δ 199.0, 171.3, 169.1, 143.6, 134.7, 129.2, 128.2, 86.9, 53.8, 35.3, 32.9, 21.6. HRMS (ESI) Calculated for $C_{16}H_{19}N_2O_3^+$ ([M+H]$^+$): 287.1390, found: 287.1391.

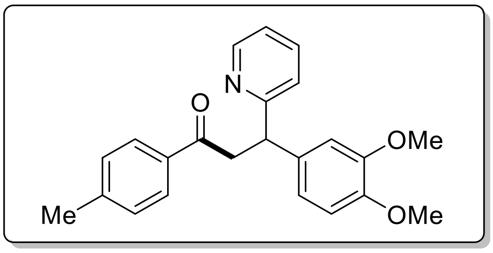

3-(3,4-dimethoxyphenyl)-3-(pyridin-2-yl)-1-(*p*-tolyl)propan-1-one **3ll**

The reaction was carried out according to the general procedure on 0.2 mmol scale (60 h). The residue was purified by flash column chromatography (petroleum ether: ethyl acetate, 10/1-4/1) to afford **3ll**, (43%, 31.0 mg), yellowish oil. ^{1}H NMR (400 MHz, Chloroform-*d*) δ 8.49 (d, *J* = 2.5 Hz, 1H), 7.89 (d, *J* = 8.1 Hz, 2H), 7.54 (t, *J* = 7.7 Hz, 1H), 7.22 (t, *J* = 8.6 Hz, 3H), 7.09 - 7.00 (m, 1H), 6.90 (d, *J* = 12.4 Hz, 2H), 6.77 (d, *J* = 8.1 Hz, 1H), 4.84 (t, *J* = 7.0 Hz, 1H), 4.25 (dd, *J* = 18.4, 7.5 Hz, 1H), 3.84 - 3.80 (m, 6H), 3.54 - 3.49 (m, 1H), 2.37 (s, 3H). ^{13}C NMR (100 MHz, Chloroform-*d*) δ 198.4, 162.6, 148.9, 148.8, 147.7, 143.7, 136.5, 136.3, 134.7, 129.2, 128.3, 123.8, 121.4, 120.0, 111.4, 111.2, 55.9, 55.8, 47.7, 43.9, 21.6. HRMS (ESI) Calculated for $C_{23}H_{24}NO_3^+$ ([M+H]$^+$): 362.1751, found: 362.1752.

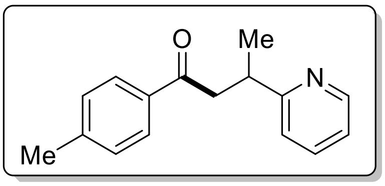

3-(pyridin-2-yl)-1-(*p*-tolyl)butan-1-one **3mm**

The reaction was carried out according to the general procedure on 0.2 mmol scale (60 h). The residue was purified by flash column chromatography (petroleum ether: ethyl acetate, 10/1-4/1) to afford **3mm**, (70%, 33.5 mg), yellowish oil. ^{1}H NMR (400 MHz, Chloroform-*d*) δ 8.50 (d, *J* = 4.7 Hz, 1H), 7.87 (d, *J* = 6.7 Hz, 2H), 7.68 - 7.53 (m, 1H), 7.29 - 7.16 (m, 3H), 7.13 - 7.01 (m, 1H), 3.66 - 3.60 (m, 2H), 3.26 - 3.08 (m, 1H), 2.38

(s, 3H), 1.37 (d, J = 6.6 Hz, 2H). ^{13}C NMR (100 MHz, Chloroform-d) δ 198.9, 165.1, 149.1, 143.7, 136.4, 134.7, 129.2, 128.3, 122.5, 121.3, 44.8, 37.3, 21.6, 21.1. HRMS (ESI) Calculated for $C_{16}H_{18}NO^+$ ([M+H]$^+$): 240.1383, found: 240.1385.

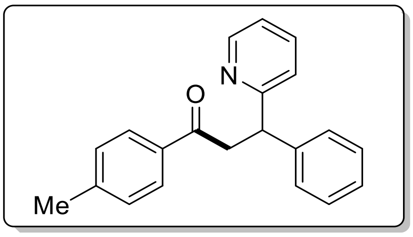

3-phenyl-3-(pyridin-2-yl)-1-(*p*-tolyl)propan-1-one **3nn**

The reaction was carried out according to the general procedure on 0.2 mmol scale (60 h). The residue was purified by flash column chromatography (petroleum ether: ethyl acetate, 10/1-4/1) to afford **3nn**, (80%, 48.2 mg), yellowish oil. ^1H NMR (400 MHz, Chloroform-d) δ 8.48 (d, J = 5.6 Hz, 1H), 7.89 (d, J = 8.2 Hz, 2H), 7.52 (td, J = 7.7, 1.8 Hz, 1H), 7.36 (d, J = 7.4 Hz, 2H), 7.31 - 7.14 (m, 6H), 7.04 (dd, J = 6.9, 5.4 Hz, 1H), 4.89 (dd, J = 8.7, 5.5 Hz, 1H), 4.31 (dd, J = 17.5, 8.7 Hz, 1H), 3.50 (dd, J = 17.5, 5.4 Hz, 1H), 2.37 (s, 3H). ^{13}C NMR (100 MHz, Chloroform-d) δ 198.3, 162.4, 148.9, 143.8, 143.7, 136.4, 134.7, 129.2, 128.6, 128.3, 128.1, 126.6, 123.9, 121.4, 48.1, 43.8, 21.6. HRMS (ESI) Calculated for $C_{21}H_{20}NO^+$ ([M+H]$^+$): 302.1539, found: 302.1541.

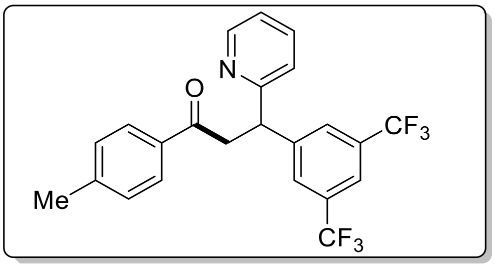

3-(3,5-bis(trifluoromethyl)phenyl)-3-(pyridin-2-yl)-1-(*p*-tolyl)propan-1-one **3oo**

The reaction was carried out according to the general procedure on 0.2 mmol scale (48 h). The residue was purified by flash column chromatography (petroleum ether: ethyl acetate, 10/1-4/1) to afford **3oo**, (89%, 77.8 mg), yellowish oil. ^1H NMR (400 MHz, Chloroform-d) δ 8.54 (d, J = 4.6 Hz, 1H), 7.95 - 7.82 (m, 4H), 7.71 (s, 1H), 7.59 (t, J = 7.7 Hz, 1H), 7.25 (dd, J = 17.4, 7.9 Hz, 3H), 7.16 - 7.07 (m, 1H), 5.00 (dd, J = 8.0, 5.9 Hz, 1H), 4.30 (dd, J = 17.7, 8.2 Hz, 1H), 3.57 (dd, J = 17.7, 5.8 Hz, 1H), 2.39 (s, 3H). ^{13}C NMR (100 MHz, Chloroform-d) δ 197.2, 160.6, 149.5, 146.1, 144.1, 136.8, 134.2, 131.7 (q, J = 33.1 Hz), 129.3, 128.5, 128.5, 128.3, 123.8, 123.3 (q, J = 273.1

Hz), 122.1, 121.1 - 120.5 (m), 47.7, 43.9, 21.6. HRMS (ESI) Calculated for $C_{23}H_{18}F_6NO^+$ ($[M+H]^+$): 438.1287, found: 438.1288.

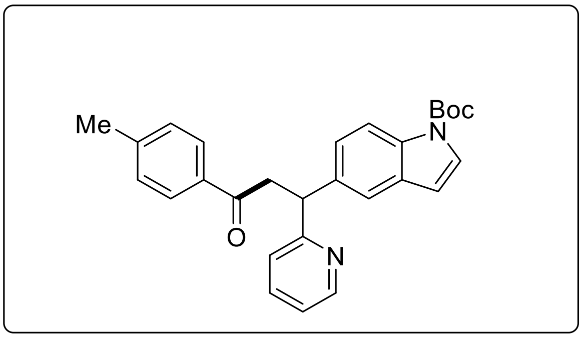

tert-butyl 5-(3-oxo-1-(pyridin-2-yl)-3-(*p*-tolyl)propyl)-1*H*-indole-1-carboxylate, **3pp**

The reaction was carried out according to the general procedure on 0.2 mmol scale (60 h). The residue was purified by flash column chromatography (petroleum ether: ethyl acetate, 10/1-4/1) to afford **3pp**, (56%, 49.3 mg), yellowish oil. ^1H NMR (400 MHz, Chloroform-*d*) δ 8.49 (d, *J* = 5.7 Hz, 1H), 8.05 (s, 1H), 7.90 (d, *J* = 8.2 Hz, 2H), 7.59 - 7.44 (m, 3H), 7.33 (d, *J* = 8.6 Hz, 1H), 7.24 - 7.12 (m, 3H), 7.07 - 6.96 (m, 1H), 6.49 (d, *J* = 3.6 Hz, 1H), 5.00 (dd, *J* = 8.4, 5.7 Hz, 1H), 4.35 (dd, *J* = 17.5, 8.5 Hz, 1H), 3.57 (dd, *J* = 17.5, 5.7 Hz, 1H), 2.36 (s, 3H), 1.63 (s, 9H). ^{13}C NMR (100 MHz, Chloroform-*d*) δ 198.4, 162.9, 149.8, 148.8, 143.6, 138.2, 136.4, 134.8, 134.0, 130.9, 129.2, 128.3, 126.2, 124.5, 123.9, 121.3, 120.3, 115.3, 107.4, 83.6, 47.9, 44.1, 28.2, 21.6. HRMS (ESI) Calculated for $C_{28}H_{29}N_2O_3^+$ ($[M+H]^+$): 441.2173, found: 441.2175.

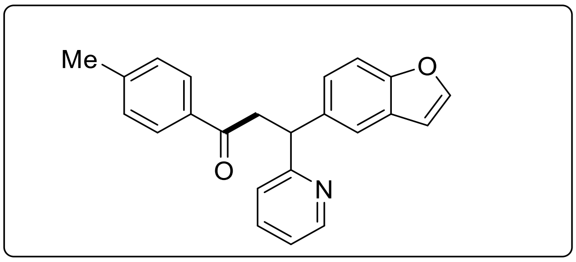

3-(benzofuran-5-yl)-3-(pyridin-2-yl)-1-(*p*-tolyl)propan-1-one, **3qq**

The reaction was carried out according to the general procedure on 0.2 mmol scale (48 h). The residue was purified by flash column chromatography (petroleum ether: ethyl acetate, 10/1-4/1) to afford **3qq**, (68%, 46.5 mg), yellowish oil. ^1H NMR (400 MHz, Chloroform-*d*) δ 8.49 (d, *J* = 4.8 Hz, 1H), 7.89 (d, *J* = 8.2 Hz, 2H), 7.59 (s, 1H), 7.56 - 7.47 (m, 2H), 7.39 (d, *J* = 8.5 Hz, 1H), 7.30 (dd, *J* = 8.5, 1.8 Hz, 1H), 7.22 (dd, *J* =

14.8, 7.9 Hz, 3H), 7.03 (dd, J = 8.4, 4.9 Hz, 1H), 6.67 (dd, J = 2.1, 0.7 Hz, 1H), 5.00 (dd, J = 8.5, 5.6 Hz, 1H), 4.34 (dd, J = 17.5, 8.5 Hz, 1H), 3.56 (dd, J = 17.5, 5.6 Hz, 1H), 2.36 (s, 3H). ^{13}C NMR (100 MHz, Chloroform-*d*) δ 198.3, 162.8, 153.9, 148.9, 145.3, 143.7, 138.4, 136.4, 134.7, 129.2, 128.3, 127.7, 124.6, 123.9, 121.4, 120.5, 111.4, 106.7, 47.9, 44.3, 21.6. HRMS (ESI) Calculated for $C_{23}H_{20}NO_2^+$ ([M+H]$^+$): 342.1489, found: 342.1490.

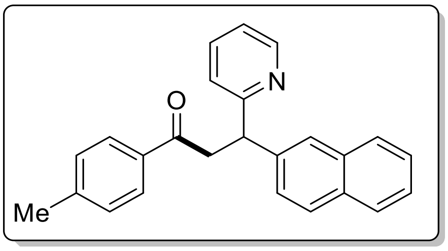

3-(naphthalen-2-yl)-3-(pyridin-2-yl)-1-(*p*-tolyl)propan-1-one **3rr**

The reaction was carried out according to the general procedure on 0.2 mmol scale (60 h). The residue was purified by flash column chromatography (10:1 petroleum ether: ethyl acetate) to afford **3rr**, (62%, 43.5 mg), yellowish solid, mp: 147 − 149 °C. ^{1}H NMR (400 MHz, Chloroform-*d*) δ 8.51 (d, J = 4.8 Hz, 1H), 7.90 (d, J = 8.2 Hz, 2H), 7.82 - 7.72 (m, 4H), 7.57 - 7.36 (m, 4H), 7.28 – 7.19(m, 3H), 7.10 - 6.98 (m, 1H), 5.07 (dd, J = 8.5, 5.6 Hz, 1H), 4.40 (dd, J = 17.5, 8.5 Hz, 1H), 3.60 (dd, J = 17.5, 5.5 Hz, 1H), 2.37 (s, 3H). ^{13}C NMR (100 MHz, Chloroform-*d*) δ 198.2, 162.3, 148.9, 143.7, 141.2, 136.4, 134.7, 133.6, 132.3, 129.2, 128.3, 127.8, 127.6, 126.5, 126.0, 125.6, 124.0, 121.4, 48.2, 43.7, 21.7. HRMS (ESI) Calculated for $C_{25}H_{22}NO^+$ ([M+H]$^+$): 352.1696, found: 352.1698.

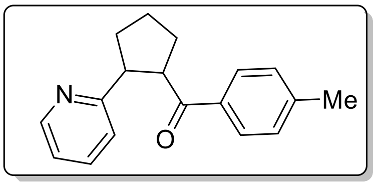

(2-(pyridin-2-yl)cyclopentyl)(*p*-tolyl)methanone **3ss**

The reaction was carried out according to the general procedure on 0.2 mmol scale (48 h). The residue was purified by flash column chromatography (petroleum ether: ethyl acetate, 10/1-4/1) to afford **3ss** (32.3 mg, 61%, d.r. = 3.8/1), yellowish oil. Major product (*trans* diastereomer): ^{1}H NMR (400 MHz, Chloroform-d) δ 8.28 (d, J = 4.7 Hz, 1H), 7.63 (d, J = 8.1 Hz, 2H), 7.39 (t, J = 8.4 Hz, 1H), 7.09 (d, J = 8.0 Hz, 2H), 7.04

(d, J = 7.9 Hz, 1H), 6.89 (dd, J = 7.3, 5.0 Hz, 1H), 4.33 (q, J = 8.0 Hz, 1H), 3.71 (q, J = 8.5 Hz, 1H), 2.33 (s, 3H), 2.28 - 2.17 (m, 3H), 2.14 - 2.02 (m, 2H), 1.91 - 1.75 (m, 1H). ^{13}C NMR (100 MHz, Chloroform-*d*) δ 202.2, 162.0, 148.5, 142.9, 135.8, 135.3, 128.8, 128.3, 122.1, 121.0, 52.0, 50.4, 31.3, 29.3, 24.8, 21.5. HRMS (ESI) Calculated for $C_{18}H_{20}NO^+$ ($[M+H]^+$): 266.1539, found: 266.1538. Minor product (*cis* diastereomer)：^1H NMR (400 MHz, Chloroform-*d*) δ 8.53 (d, J = 4.8 Hz, 1H), 7.79 (d, J = 8.2 Hz, 2H), 7.50 (td, J = 7.7, 1.9 Hz, 1H), 7.20 - 7.16 m, 3H), 7.06 (dd, J = 8.6, 4.9 Hz, 1H), 4.26 - 4.14 (m, 1H), 3.86 - 3.70 (m, 1H), 2.36 (s, 3H), 2.33 - 2.16 (m, 2H), 2.11 - 1.95 (m, 2H), 1.94 - 1.71 (m, 2H). HRMS (ESI) Calculated for $C_{18}H_{20}NO^+$ ($[M+H]^+$): 266.1539, found: 266.1540.

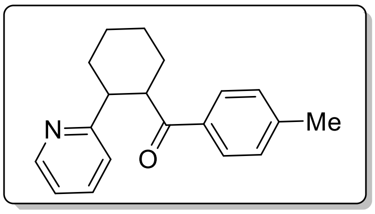

(2-(pyridin-2-yl)cyclohexyl)(*p*-tolyl)methanone **3tt**

The reaction was carried out according to the general procedure on 0.2 mmol scale (48 h). The residue was purified by flash column chromatography (petroleum ether: ethyl acetate, 10/1-4/1) to afford **3tt** (32.5 mg, 58%, d.r. = 4.3/1), yellowish oil. Major product (*trans* diastereomer)：^1H NMR (400 MHz, Chloroform-*d*) δ 8.38 (d, J = 4.2 Hz, 1H), 7.68 (d, J = 8.2 Hz, 2H), 7.52 (td, J = 7.8, 1.8 Hz, 1H), 7.29 (d, J = 7.9 Hz, 1H), 7.14 (d, J = 8.1 Hz, 2H), 6.98 (dd, J = 7.2, 5.0 Hz, 1H), 4.45 - 4.26 (m, 1H), 3.16 (dt, J = 12.1, 4.2 Hz, 1H), 2.77 - 2.56 (m, 1H), 2.34 (s, 3H), 2.10 - 2.03 (m, 1H), 2.02 - 1.85 (m, 3H), 1.60 - 1.40 (m, 3H). ^{13}C NMR (100 MHz, Chloroform-*d*) δ 202.9, 163.7, 148.4, 142.9, 136.1, 135.1, 129.0, 128.2, 121.6, 121.0, 46.9, 44.9, 29.1, 26.1, 25.9, 21.6, 21.5. HRMS (ESI) Calculated for $C_{16}H_{18}NO^+$ ($[M+H]^+$): 280.1696, found: 280.1699. Minor product (*cis* diastereomer)：^1H NMR (400 MHz, Chloroform-*d*) δ 8.37 (d, J = 5.5 Hz, 1H), 7.79 (d, J = 8.2 Hz, 2H), 7.47 (td, J = 7.7, 1.8 Hz, 1H), 7.19 - 7.15 (m, 3H), 6.95 (dd, J = 8.0, 4.4 Hz, 1H), 4.03 - 4.00 (m, 1H), 3.39 - 3.18 (m, 1H), 2.36 (s, 3H), 2.05 - 2.00 (m, 2H), 1.94 - 1.84 (m, 2H), 1.76 - 1.73 (m, 1H), 1.53 - 1.44 (m, 3H). HRMS (ESI) Calculated for $C_{16}H_{18}NO^+$ ($[M+H]^+$): 280.1696, found: 280.1697.

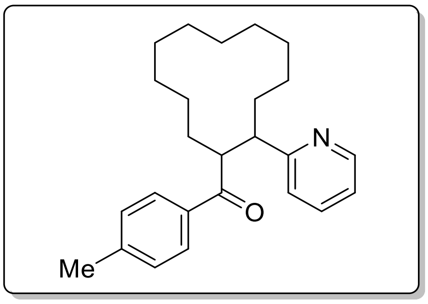

(2-(pyridin-2-yl)cyclododecyl)(*p*-tolyl)methanone **3uu**

The reaction was carried out according to the general procedure on 0.2 mmol scale (48 h). The residue was purified by flash column chromatography (petroleum ether: ethyl acetate, 10/1-4/1) to afford **3uu** (34.8 mg, 48%, d.r. = 7.8/1), yellowish oil. Major isomer : ^{1}H NMR (400 MHz, Chloroform-*d*) δ 8.47 (d, *J* = 8.2 Hz, 1H), 7.63 (d, *J* = 7.8 Hz, 2H), 7.41 (td, *J* = 7.7, 1.8 Hz, 1H), 7.16 (d, *J* = 8.1 Hz, 2H), 7.02 (dd, *J* = 7.8, 5.3 Hz, 1H), 6.85 (d, *J* = 7.8 Hz, 1H), 3.93 - 3.89 (m, 1H), 3.34 - 3.30 (m, 1H), 2.37 (s, 3H), 2.32 - 2.19 (m, 1H), 1.96 - 1.87 (m, 1H), 1.86 - 1.68 (m, 1H), 1.67 - 1.28 (m, 17H). ^{13}C NMR (100 MHz, Chloroform-*d*) δ 202.4, 162.2, 148.8, 143.1, 135.7, 135.4, 129.1, 128.2, 122.6, 121.4, 48.1, 47.4, 46.2, 43.7, 25.6, 24.5, 24.2, 23.8, 23.7, 23.4, 22.9 , 22.1, 21.6. HRMS (ESI) Calculated for $C_{25}H_{34}NO^{+}$ ($[M+H]^{+}$): 364.2635, found: 364.2636.

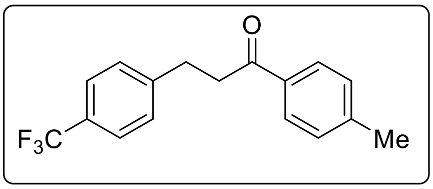

1-(*p*-tolyl)-3-(4-(trifluoromethyl)phenyl)propan-1-one **3vv**

The reaction was carried out according to the general procedure on 0.2 mmol scale (48 h). The residue was purified by flash column chromatography (petroleum ether: ethyl acetate, 80/1-50/1) to afford **3vv** (37.4 mg, 64%) as a white solid, mp: 98 − 100 °C. ^{1}H NMR (400 MHz, Chloroform-*d*) δ 7.85 (d, *J* = 8.2 Hz, 2H), 7.54 (d, *J* = 8.0 Hz, 1H), 7.37 (d, *J* = 8.0 Hz, 1H), 7.25 (d, *J* = 8.2 Hz, 2H), 3.30 (t, *J* = 7.6 Hz, 2H), 3.12 (t, *J* = 7.6 Hz, 2H), 2.41 (s, 3H). ^{13}C NMR (100 MHz, Chloroform-*d*) δ 198.2, 145.6, 144.1, 134.2, 129.4, 128.8, 128.1, 125.4 (q, *J* = 3.8 Hz), 124.3 (q, *J* = 272.2 Hz), 39.7, 29.9, 21.7. HRMS (ESI) Calculated for $C_{17}H_{16}F_{3}O^{+}$ ($[M+H]^{+}$): 293.1148, found: 293.1149.

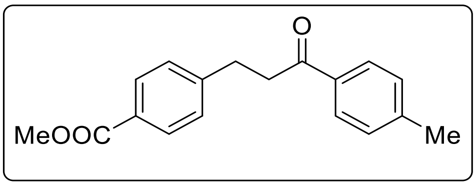

methyl 4-(3-oxo-3-(*p*-tolyl)propyl)benzoate **3ww**

The reaction was carried out according to the general procedure on 0.2 mmol scale (48 h). The residue was purified by flash column chromatography (petroleum ether: ethyl acetate, 50/1-20/1) to afford **3ww** (37.8 mg, 67%) as a white solid, mp: 110 − 112 °C. ^{1}H NMR (400 MHz, Chloroform-*d*) δ 7.97 (d, *J* = 8.2 Hz, 2H), 7.85 (d, *J* = 8.2 Hz, 2H), 7.32 (d, *J* = 8.2 Hz, 2H), 7.25 (d, *J* = 8.2 Hz, 2H), 3.90 (s, 3H), 3.29 (t, *J* = 7.6 Hz, 2H), 3.12 (t, *J* = 7.6 Hz, 2H), 2.41 (s, 3H). ^{13}C NMR (100 MHz, Chloroform-*d*) δ 198.4, 167.1, 146.9, 144.0, 134.2, 129.9, 129.3, 128.5, 128.2, 126.6, 52.0, 39.7, 30.1, 21.7. HRMS (ESI) Calculated for $C_{18}H_{19}O_3^+$ ($[M+H]^+$): 283.1329, found: 283.1332.

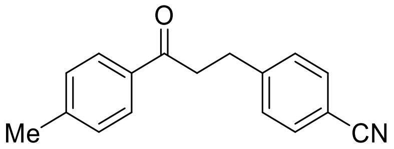

4-(3-oxo-3-(*p*-tolyl)propyl)benzonitrile **3xx**

The reaction was carried out according to the general procedure on 0.2 mmol scale (48 h). The residue was purified by flash column chromatography (petroleum ether: ethyl acetate, 50/1-30/1) to afford **3xx**, (68%, 33.9 mg), as a white solid, mp: 162 − 164 °C. ^{1}H NMR (400 MHz, Chloroform-*d*) δ 7.84 (d, *J* = 8.2 Hz, 2H), 7.58 (d, *J* = 8.2 Hz, 2H), 7.36 (d, *J* = 8.4 Hz, 2H), 7.26 (d, *J* = 8.4 Hz, 2H), 3.30 (t, *J* = 7.4 Hz, 2H), 3.13 (t, *J* = 7.4 Hz, 2H), 2.41 (s, 3H). ^{13}C NMR (100 MHz, Chloroform-*d*) δ 197.9, 147.1, 144.2, 134.1, 132.3, 129.4, 129.3, 128.1, 119.0, 110.0, 39.3, 30.1, 21.7. HRMS (ESI) Calculated for $C_{17}H_{16}NO^+$ ($[M+H]^+$): 250.1226, found: 250.1228.

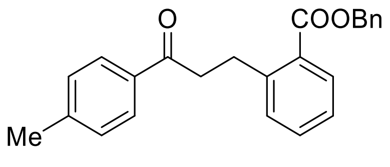

benzyl 2-(3-oxo-3-(*p*-tolyl)propyl)benzoate **3yy**

The reaction was carried out according to the general procedure on 0.2 mmol scale (48 h). The residue was purified by flash column chromatography (petroleum ether: ethyl acetate, 50/1-20/1) to afford **3yy**, (64%, 45.8 mg), as a white solid, mp: 112 − 114 °C. ^{1}H NMR (400 MHz, Chloroform-*d*) δ 7.95 (d, *J* = 9.0 Hz, 1H), 7.81 (d, *J* = 8.2 Hz, 2H), 7.41 (d, *J* = 7.5 Hz, 3H), 7.35 – 7.14 (m, 7H), 5.33 (s, 2H), 3.38 – 3.33 (m, 2H), 3.28 – 3.21 (m, 2H), 2.39 (s, 3H). ^{13}C NMR (100 MHz, Chloroform-*d*) δ 198.9, 167.2, 143.6, 143.5, 135.9, 134.4, 132.3, 131.5, 131.0, 129.5, 129.2, 128.6, 128.4, 128.3, 128.2, 126.3, 66.8, 40.5, 29.4, 21.7. HRMS (ESI) Calculated for $C_{24}H_{23}O_3^+$ ([M+H]$^+$): 359.1642, found: 359.1644.

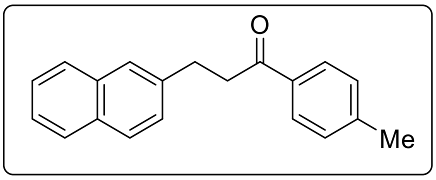

3-(naphthalen-2-yl)-1-(*p*-tolyl)propan-1-one **3zz**

The reaction was carried out according to the general procedure on 0.2 mmol scale (48 h). The residue was purified by flash column chromatography (petroleum ether: ethyl acetate, 100/1-50/1) to afford **3zz** (22.5 mg, 41%) as a white solid, mp: 108 − 110 °C . ^{1}H NMR (400 MHz, Chloroform-*d*) δ 7.88 (d, *J* = 8.2 Hz, 2H), 7.80 (t, *J* = 8.0 Hz, 3H), 7.69 (s, 1H), 7.50 – 7.35 (m, 3H), 7.25 (d, *J* = 7.9 Hz, 2H), 3.40 – 3.32 (m, 2H), 3.28 – 3.18 (m, 2H), 2.40 (s, 3H). ^{13}C NMR (100 MHz, Chloroform-*d*) δ 198.9, 143.9, 138.9, 134.4, 133.6, 132.0, 129.3, 128.2, 128.1, 127.6, 127.5, 127.2, 126.5, 126.0, 125.3, 40.3, 30.4, 21.7. HRMS (ESI) Calculated for $C_{20}H_{19}O^+$ ([M+H]$^+$): 275.1430, found: 275.1432.

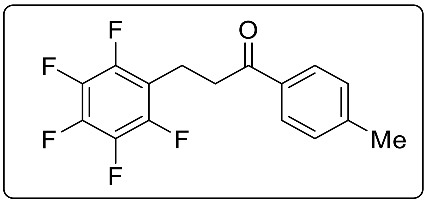

3-(perfluorophenyl)-1-(*p*-tolyl)propan-1-one **3aA**

The reaction was carried out according to the general procedure on 0.2 mmol scale (48 h). The residue was purified by flash column chromatography (petroleum ether: ethyl acetate, 50/1-20/1) to afford **3aA** (32.7 mg, 52%) as a white solid, mp: 63 − 65 °C. ^{1}H NMR (400 MHz, Chloroform-*d*) δ 7.84 (d, *J* = 8.2 Hz, 2H), 7.26 (d, *J* = 8.2 Hz, 2H),

3.27 (t, J = 7.4 Hz, 2H), 3.13 (t, J = 7.4 Hz, 2H), 2.41 (s, 3H). ^{13}C NMR (100 MHz, Chloroform-*d*) δ 197.3, 146.8 – 146.1 (m), 144.3, 144.2 – 143.7 (m), 139.1 – 138.4 (m), 136.3 – 135.8 (m), 133.8, 129.4, 128.1, 37.3, 21.7, 17.2. HRMS (ESI) Calculated for $C_{16}H_{12}F_5O^+$ ([M+H]$^+$): 315.0803, found: 315.0805.

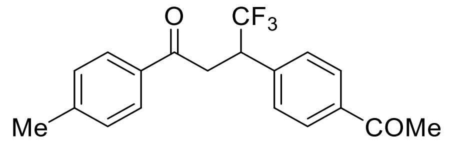

3-(4-acetylphenyl)-4,4,4-trifluoro-1-(*p*-tolyl)butan-1-one **3bB**

The reaction was carried out according to the general procedure on 0.2 mmol scale (48 h). The residue was purified by flash column chromatography (petroleum ether: ethyl acetate, 20/1-10/1) to afford **3bB**, (79%, 52.8 mg), as a white solid, mp: 191 – 193 °C. ^{1}H NMR (400 MHz, Chloroform-*d*) δ 7.92 (d, J = 8.4 Hz, 2H), 7.82 (d, J = 8.4 Hz, 2H), 7.50 (d, J = 8.2 Hz, 2H), 7.25 (d, J = 8.2 Hz, 2H), 4.33 – 4.29 (m, 1H), 3.72 (dd, J = 17.8, 9.7 Hz, 1H), 3.60 (dd, J = 17.8, 3.8 Hz, 1H), 2.57 (s, 3H), 2.40 (s, 3H). ^{13}C NMR (100 MHz, Chloroform-*d*) δ 197.5, 194.5, 144.7, 139.8, 136.9, 133.6, 129.5, 129.4, 128.7, 128.2, 126.6 (q, J = 279.6 Hz), 44.9 (q, J = 27.6 Hz), 37.9, 26.6, 21.7. HRMS (ESI) Calculated for $C_{19}H_{18}F_3O_2^+$ ([M+H]$^+$): 335.1253, found: 335.1255.

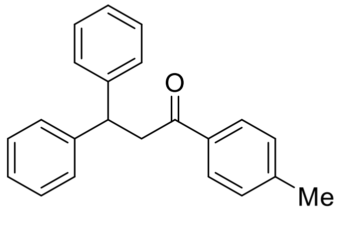

3,3-diphenyl-1-(*p*-tolyl)propan-1-one **3cC**

The reaction was carried out according to the general procedure on 0.2 mmol scale (48 h). The residue was purified by flash column chromatography (petroleum ether: ethyl acetate, 50/1-20/1) to afford **3cC** (43.2 mg, 72%) as a white solid, mp: 127 − 129 °C. ^{1}H NMR (400 MHz, Chloroform-*d*) δ 7.84 (d, J = 8.2 Hz, 2H), 7.28 – 7.20 (m, 10H), 7.19 – 7.14 (m, 2H), 4.82 (t, J = 7.3 Hz, 1H), 3.71 (d, J = 7.3 Hz, 2H), 2.39 (s, 3H). ^{13}C NMR (100 MHz, Chloroform-*d*) δ 197.6, 144.2, 143.9, 134.6, 129.3, 128.6, 128.2,

127.9, 126.4, 46.0, 44.6, 21.7. HRMS (ESI) Calculated for $C_{22}H_{21}O^+$ ($[M+H]^+$): 301.1587, found: 301.1588.

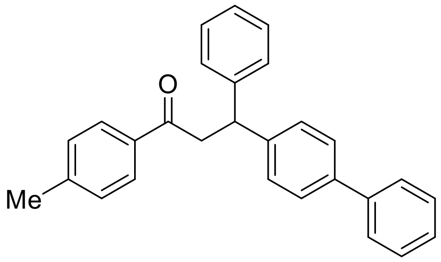

3-([1,1'-biphenyl]-4-yl)-3-phenyl-1-(*p*-tolyl)propan-1-one **3dD**

The reaction was carried out according to the general procedure on 0.2 mmol scale (48 h). The residue was purified by flash column chromatography (petroleum ether: ethyl acetate, 50/1-20/1) to afford **3dD** (52%, 39.1 mg) as a white solid, mp: 161 − 163 °C. ^{1}H NMR (400 MHz, Chloroform-*d*) δ 7.86 (d, *J* = 8.2 Hz, 2H), 7.57 – 7.51 (m, 2H), 7.49 (d, *J* = 8.3 Hz, 2H), 7.39 (t, *J* = 7.6 Hz, 2H), 7.35 – 7.15 (m, 10H), 4.87 (t, *J* = 7.3 Hz, 1H), 3.80 – 3.68 (m, 2H), 2.39 (s, 3H). ^{13}C NMR (100 MHz, Chloroform-*d*) δ 197.6, 144.2, 144.0, 143.4, 140.9, 139.2, 134.6, 129.3, 128.7, 128.6, 128.3, 128.2, 127.9, 127.3, 127.1, 127.0, 126.5, 45.7, 44.6, 21.7. HRMS (ESI) Calculated for $C_{28}H_{25}O^+$ ($[M+H]^+$): 377.1900, found: 377.1901.

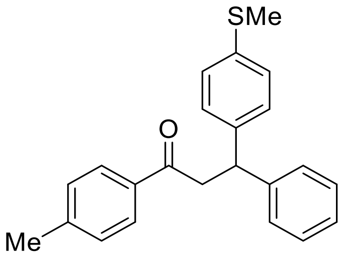

3-(4-(methylthio)phenyl)-3-phenyl-1-(*p*-tolyl)propan-1-one **3eE**

The reaction was carried out according to the general procedure on 0.2 mmol scale (48 h). The residue was purified by flash column chromatography (petroleum ether: ethyl acetate, 50/1-20/1) to afford **3eE**, (58%, 40.1 mg) as a white solid, mp: 111 − 113 °C. ^{1}H NMR (400 MHz, Chloroform-*d*) δ 7.83 (d, *J* = 8.2 Hz, 2H), 7.33 – 7.08 (m, 11H), 4.78 (t, *J* = 7.3 Hz, 1H), 3.70 (dd, *J* = 17.6, 7.8 Hz, 1H), 3.65 (dd, *J* = 17.6, 7.8 Hz, 1H),

2.42 (s, 3H), 2.39 (s, 3H). ^{13}C NMR (100 MHz, Chloroform-*d*) δ 197.5, 144.2, 144.0, 141.3, 136.1, 134.5, 129.3, 128a.6, 128.4, 128.2, 127.8, 127.0, 126.4, 45.5, 44.5, 21.7, 16.0. HRMS (ESI) Calculated for $C_{23}H_{23}OS^+$ ([M+H]$^+$): 347.1464, found: 347.1465.

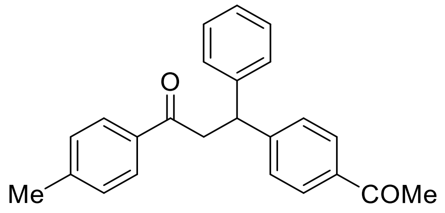

3-(4-acetylphenyl)-3-phenyl-1-(*p*-tolyl)propan-1-one **3fF**

The reaction was carried out according to the general procedure on 0.2 mmol scale (48 h). The residue was purified by flash column chromatography (petroleum ether: ethyl acetate, 20/1-10/1) to afford **3fF**, (67%, 45.8 mg) as a white solid, mp: 141 − 143 °C. ^{1}H NMR (400 MHz, Chloroform-*d*) δ 7.92 – 7.78 (m, 4H), 7.36 (d, *J* = 8.3 Hz, 2H), 7.31 – 7.16 (m, 7H), 4.88 (t, *J* = 7.3 Hz, 1H), 3.77 (dd, *J* = 17.2, 7.3 Hz, 1H), 3.69 (dd, *J* = 17.2, 7.3 Hz, 1H), 2.53 (s, 3H), 2.39 (s, 3H). ^{13}C NMR (100 MHz, Chloroform-*d*) δ 197.7, 197.4, 149.8, 144.2, 143.4, 135.4, 134.4, 129.4, 128.8, 128.7, 128.2, 128.1, 127.8, 126.7, 45.9, 44.2, 26.6, 21.7. HRMS (ESI) Calculated for $C_{24}H_{23}O_2^+$ ([M+H]$^+$): 343.1693, found: 343.1696.

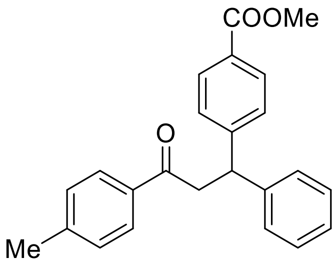

methyl 4-(3-oxo-1-phenyl-3-(*p*-tolyl)propyl)benzoate **3gG**

The reaction was carried out according to the general procedure on 0.2 mmol scale (48 h). The residue was purified by flash column chromatography (petroleum ether: ethyl acetate, 20/1-10/1) to afford **3gG**, (70%, 50.1 mg) as a white solid, mp: 165 − 167 °C. ^{1}H NMR (400 MHz, Chloroform-*d*) δ 7.93 (d, *J* = 8.4 Hz, 2H), 7.83 (d, *J* = 8.2 Hz, 2H),

7.33 (d, J = 8.3 Hz, 2H), 7.30 – 7.15 (m, 7H), 4.88 (t, J = 7.3 Hz, 1H), 3.86 (s, 3H), 3.78 – 3.66 (m, 2H), 2.39 (s, 3H). ^{13}C NMR (100 MHz, Chloroform-*d*) δ 197.2, 166.9, 149.5, 144.1, 143.5, 134.4, 129.9, 129.4, 128.7, 128.3, 128.2, 127.9 (2C), 126.7, 52.0, 46.0, 44.2, 21.7. HRMS (ESI) Calculated for $C_{24}H_{23}O_3^+$ ([M+H]$^+$): 359.1642, found: 359.1643.

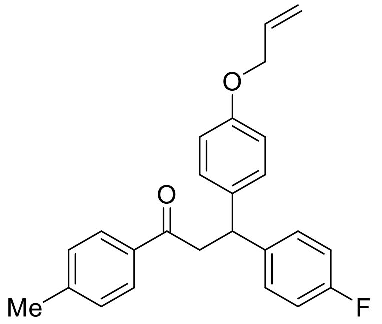

3-(4-(allyloxy)phenyl)-3-(4-fluorophenyl)-1-(*p*-tolyl)propan-1-one **3hH**

The reaction was carried out according to the general procedure on 0.2 mmol scale (48 h). The residue was purified by flash column chromatography (petroleum ether: ethyl acetate, 50/1-20/1) to afford **3hH**, (43%, 32.2 mg) as a white solid, mp: 131 − 133 °C . ^{1}H NMR (400 MHz, Chloroform-*d*) δ 7.83 (d, J = 8.2 Hz, 2H), 7.29 – 7.16 (m, 4H), 7.13 (d, J = 8.6 Hz, 2H), 6.94 (t, J = 8.7 Hz, 2H), 6.82 (d, J = 8.7 Hz, 2H), 6.28 – 5.82 (m, 1H), 5.38 (d, J = 18.8 Hz, 1H), 5.26 (d, J = 10.5 Hz, 1H), 4.75 (t, J = 7.3 Hz, 1H), 4.50 – 4.46 (m, 2H), 3.64 (d, J = 7.4 Hz, 2H), 2.40 (s, 3H). ^{13}C NMR (100 MHz, Chloroform-*d*) δ 197.6, 162.5, 160.1, 157.2, 144.0, 140.3 (d, J = 3.2 Hz), 135.5 (d, J = 188.8 Hz), 133.3, 129.3, 129.2 (2C), 128.4 (d, J = 47.6 Hz), 117.6, 115.3 (d, J = 21.2 Hz), 114.8, 68.8, 44.9, 44.5, 21.7. HRMS (ESI) Calculated for $C_{25}H_{24}FO_2^+$ ([M+H]$^+$): 375.1755, found: 375.1756.

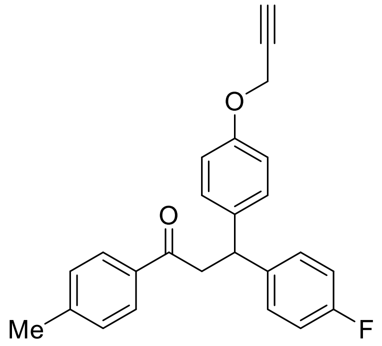

3-(4-fluorophenyl)-3-(4-(prop-2-yn-1-yloxy)phenyl)-1-(*p*-tolyl)propan-1-one **3il**

The reaction was carried out according to the general procedure on 0.2 mmol scale (48 h). The residue was purified by flash column chromatography (petroleum ether: ethyl acetate, 50/1-20/1) to afford **3il**, (47%, 35.0 mg) as a white solid mp: 116 − 118 °C . ^{1}H NMR (400 MHz, Chloroform-*d*) δ 7.83 (d, *J* = 8.2 Hz, 2H), 7.30 – 7.10 (m, 6H), 7.05 – 6.79 (m, 4H), 4.76 (t, *J* = 7.4 Hz, 1H), 4.64 (d, *J* = 2.4 Hz, 2H), 3.64 (d, *J* = 7.4 Hz, 2H), 2.50 (t, *J* = 2.4 Hz, 1H), 2.40 (s, 3H). ^{13}C NMR (100 MHz, Chloroform-*d*) δ 197.5, 161.4 (d, *J* = 244.6 Hz), 156.1, 144.0, 140.1, 137.2, 134.5, 129.3, 129.2 (d, *J* = 7.9 Hz), 128.7, 128.2, 115.3 (d, *J* = 21.2 Hz), 115.0, 78.6, 75.5, 55.8, 44.8, 44.4, 21.7. HRMS (ESI) Calculated for $C_{25}H_{22}FO_2^+$ ($[M+H]^+$): 373.1598, found: 373.1599.

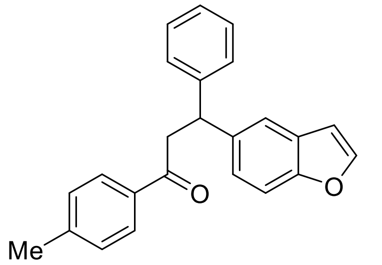

3-(benzofuran-5-yl)-3-phenyl-1-(*p*-tolyl)propan-1-one **3jJ**

The reaction was carried out according to the general procedure on 0.2 mmol scale (48 h). The residue was purified by flash column chromatography (petroleum ether: ethyl acetate, 50/1-20/1) to afford **3jJ**, (40%, 27.2 mg) as a white solid, mp: 141 − 143 °C. ^{1}H NMR (400 MHz, Chloroform-*d*) δ 7.85 (d, *J* = 8.2 Hz, 2H), 7.56 (d, *J* = 2.2 Hz, 1H), 7.48 (d, *J* = 1.8 Hz, 1H), 7.39 (d, *J* = 8.5 Hz, 1H), 7.32 – 7.14 (m, 8H), 6.68 (d, *J* = 3.1 Hz, 1H), 4.93 (t, *J* = 7.3 Hz, 1H), 3.76 (d, *J* = 7.3 Hz, 2H), 2.40 (s, 3H). ^{13}C NMR (100

MHz, Chloroform-*d*) δ 197.7, 153.7, 145.2, 144.7, 143.9, 138.9, 134.6, 129.3, 128.6, 128.2, 127.8, 127.6, 126.3, 124.5, 120.0, 111.3, 106.6, 45.8, 45.0, 21.7. HRMS (ESI) Calculated for $C_{24}H_{21}O_2^+$ ($[M+H]^+$): 341.1536, found: 341.1536.

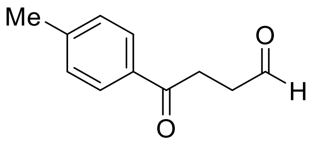

4-oxo-4-(*p*-tolyl)butanal **3kK**

The reaction was carried out according to the general procedure on 0.2 mmol scale (48 h). The residue was purified by flash column chromatography (petroleum ether: ethyl acetate, 20/1-10/1) to afford **3kK**, (51%, 18.0 mg) as colorless oil. ^{1}H NMR (400 MHz, Chloroform-*d*) δ 9.91 (s, 1H), 7.89 (d, *J* = 8.2 Hz, 2H), 7.27 (d, *J* = 8.2 Hz, 2H), 3.31 (t, *J* = 6.4 Hz, 2H), 2.93 (t, *J* = 6.4 Hz, 2H), 2.42 (s, 3H). ^{13}C NMR (100 MHz, Chloroform-*d*) δ 200.8, 197.4, 144.2, 134.0, 129.3, 128.2, 37.7, 30.9, 21.7. HRMS (ESI) Calculated for $C_{11}H_{13}O_2^+$ ($[M+H]^+$): 177.0910, found: 177.0912.

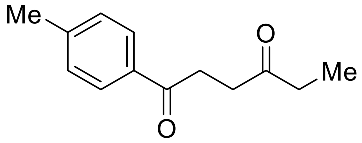

1-(*p*-tolyl)hexane-1,4-dione **3lL**

The reaction was carried out according to the general procedure on 0.2 mmol scale (48 h). The residue was purified by flash column chromatography (petroleum ether: ethyl acetate, 20/1-10/1) to afford **3lL**, (82%, 33.5 mg) as a white solid, mp: 58 − 60 °C. ^{1}H NMR (400 MHz, Chloroform-*d*) δ 7.88 (d, *J* = 8.2 Hz, 2H), 7.26 (d, *J* = 8.2 Hz, 2H), 3.27 (t, *J* = 6.3 Hz, 2H), 2.85 (t, *J* = 6.3 Hz, 2H), 2.57 (q, *J* = 7.3 Hz, 2H), 2.41 (s, 3H), 1.10 (t, *J* = 7.3 Hz, 3H). ^{13}C NMR (100 MHz, Chloroform-*d*) δ 210.3, 198.4, 143.9, 134.2, 129.3, 128.2, 36.1, 35.8, 32.3, 21.7, 7.9. HRMS (ESI) Calculated for $C_{13}H_{17}O_2^+$ ($[M+H]^+$): 205.1223, found: 205.1224.

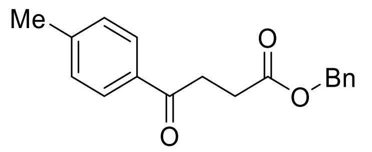

benzyl 4-oxo-4-(*p*-tolyl)butanoate **3mM**

The reaction was carried out according to the general procedure on 0.2 mmol scale (48 h). The residue was purified by flash column chromatography (petroleum ether: ethyl acetate, 20/1-10/1) to afford **3mM**, (79%, 44.6 mg) as a white solid, mp: 74 − 76 °C. ^{1}H NMR (400 MHz, Chloroform-*d*) δ 7.88 (d, *J* = 8.2 Hz, 2H), 7.43 – 7.17 (m, 7H), 5.15 (s, 2H), 3.31 (t, *J* = 6.7 Hz, 2H), 2.81 (t, *J* = 6.7 Hz, 2H), 2.41 (s, 3H). ^{13}C NMR (100 MHz, Chloroform-*d*) δ 197.6, 172.9, 144.0, 135.9, 134.1, 129.3, 128.6, 128.2, 128.2, 66.5, 33.2, 28.3, 21.7. HRMS (ESI) Calculated for $C_{18}H_{19}O_3^+$ ($[M+H]^+$): 283.1329, found: 283.1330.

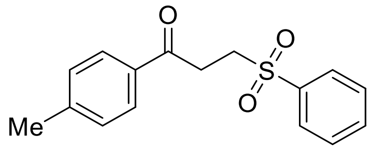

3-(phenylsulfonyl)-1-(*p*-tolyl)propan-1-one **3nN**

The reaction was carried out according to the general procedure on 0.2 mmol scale (48 h). The residue was purified by flash column chromatography (petroleum ether: ethyl acetate, 10/1-4/1) to afford **3nN** (44.9 mg, 78%) as a white solid, mp: 128 − 129 °C. ^{1}H NMR (400 MHz, Chloroform-*d*) δ 7.99 – 7.92 (m, 2H), 7.82 (d, *J* = 8.2 Hz, 2H), 7.67 (d, *J* = 6.2 Hz, 1H), 7.58 (t, *J* = 7.6 Hz, 2H), 7.30 – 7.23 (m, 2H), 3.58 – 3.54 (m, 2H), 3.55 – 3.42 (m, 2H), 2.42 (s, 3H). ^{13}C NMR (100 MHz, Chloroform-*d*) δ 195.0, 144.8, 139.1, 133.9, 133.4, 129.5, 129.4, 128.2, 128.0, 51.1, 31.2, 21.7. HRMS (ESI) Calcd for $C_{16}H_{17}O_3S$ $[M+H]^+$: 289.0893, found: 289.0895.

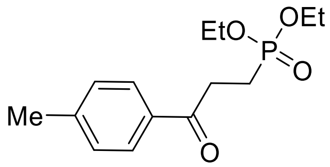

diethyl (3-oxo-3-(*p*-tolyl)propyl)phosphonate **3oO**

The reaction was carried out according to the general procedure on 0.2 mmol scale (48 h). The residue was purified by flash column chromatography (petroleum ether: ethyl acetate, 4/1-1/1) to afford **3oO** (39.2 mg, 69%) as colorless oil. ^{1}H NMR (400 MHz, Chloroform-*d*) δ 7.88 (d, *J* = 8.1 Hz, 2H), 7.27 (d, *J* = 8.1 Hz, 2H), 4.18 – 4.08 (m, 4H), 3.35 – 3.12 (m, 2H), 2.42 (s, 3H), 2.29 – 2.04 (m, 2H), 1.33 (t, *J* = 7.1 Hz, 6H). ^{13}C NMR (100 MHz, Chloroform-*d*) δ 197.1 (d, *J* = 15.7 Hz), 144.0, 133.8, 129.4, 128.2, 61.8 (d, *J* = 6.4 Hz), 31.6 (d, *J* = 3.0 Hz), 21.7, 19.8 (d, *J* = 144.5 Hz), 16.5 (d, *J* = 6.1 Hz). HRMS (ESI) Calcd for $C_{14}H_{22}O_4P$ $[M+H]^+$: 285.1250, found: 285.1251.

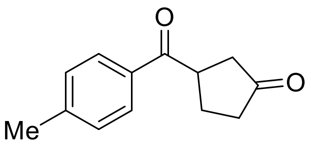

3-(4-methylbenzoyl)cyclopentan-1-one **3pP**

The reaction was carried out according to the general procedure on 0.2 mmol scale (48 h). The residue was purified by flash column chromatography (petroleum ether: ethyl acetate, 10/1-4/1) to afford **3pP**, (75%, 30.3 mg) as colorless oil. ^{1}H NMR (400 MHz, Chloroform-*d*) δ 7.90 (d, *J* = 8.2 Hz, 2H), 7.30 (d, *J* = 8.0 Hz, 2H), 4.15 – 4.07 (m, 1H), 2.71 (dd, *J* = 18.4, 7.9 Hz, 1H), 2.48 – 2.45 (m, 1H), 2.44 (s, 3H), 2.42 – 2.25 (m, 3H), 2.22 – 2.11 (m, 1H). ^{13}C NMR (100 MHz, Chloroform-*d*) δ 217.1, 199.8, 144.5, 133.1, 129.6, 128.6, 42.9, 41.1, 37.4, 27.1, 21.7. HRMS (ESI) Calculated for $C_{13}H_{15}O_2^+$ ($[M+H]^+$): 203.1067, found: 203.1069.

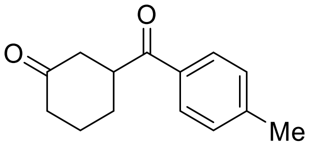

3-(4-methylbenzoyl)cyclohexan-1-one **3qQ**

The reaction was carried out according to the general procedure on 0.2 mmol scale (48 h). The residue was purified by flash column chromatography (petroleum ether: ethyl acetate, 50/1-20/1) to afford **3qQ** (29.8 mg, 69%) as colorless oil. ^{1}H NMR (400 MHz, Chloroform-*d*) δ 7.78 (d, *J* = 8.2 Hz, 2H), 7.20 (d, *J* = 8.2 Hz, 2H), 3.80 – 3.62 (m, 1H), 2.65 (dd, *J* = 14.9, 11.3 Hz, 1H), 2.46 – 2.23 (m, 6H), 2.06 – 2.02 (m, 2H), 1.87 – 1.52 (m, 2H). ^{13}C NMR (100 MHz, Chloroform-*d*) δ 209.4, 199.0, 143.4, 131.8, 128.5, 127.5, 44.1, 42.2, 40.0, 27.5, 23.9, 20.7. HRMS (ESI) Calculated for $C_{14}H_{17}O_2^+$ ([M+H]$^+$): 217.1223, found: 217.1224.

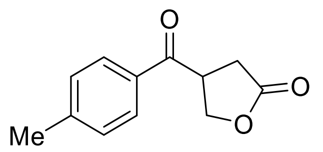

4-(4-methylbenzoyl)dihydrofuran-2(3*H*)-one **3rR**

The reaction was carried out according to the general procedure on 0.2 mmol scale (48 h). The residue was purified by flash column chromatography (petroleum ether: ethyl acetate, 10/1-4/1) to afford **3rR**, (73%, 29.8 mg) as a white solid, mp: 115 − 117 °C . ^{1}H NMR (400 MHz, Chloroform-*d*) δ 7.83 (d, *J* = 8.3 Hz, 2H), 7.32 (d, *J* = 8.0 Hz, 2H), 4.62 (t, *J* = 8.8 Hz, 1H), 4.47 (dd, *J* = 9.1, 6.9 Hz, 1H), 4.41 – 4.31 (m, 1H), 3.03 (dd, *J* = 17.8, 7.6 Hz, 1H), 2.79 (dd, *J* = 17.8, 9.4 Hz, 1H), 2.44 (s, 3H). ^{13}C NMR /(100 MHz, Chloroform-*d*) δ 195.8, 175.4, 145.4, 132.5, 129.8, 128.6, 69.1, 42.1, 30.9, 21.8. HRMS (ESI) Calculated for $C_{12}H_{13}O_3^+$ ([M+H]$^+$): 205.0859, found: 205.0861.

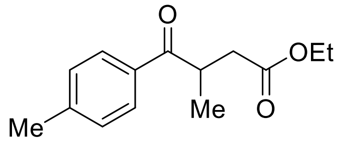

ethyl 3-methyl-4-oxo-4-(*p*-tolyl)butanoate **3sS**

The reaction was carried out according to the general procedure on 0.2 mmol scale (48 h). The residue was purified by flash column chromatography (petroleum ether: ethyl acetate, 10/1-4/1) to afford **3sS**, (72%, 33.7 mg) as colorless oil. ^{1}H NMR (400 MHz, Chloroform-*d*) δ 7.90 (d, *J* = 8.2 Hz, 2H), 7.27 (d, *J* = 8.2 Hz, 2H), 4.10 (q, *J* = 7.1 Hz,

2H), 3.97 – 3.88 (m, 1H), 2.99 – 2.88 (dd, *J* = 16.2, 8.1 Hz, 1H), 2.53 – 2.33 (m, 4H), 1.25 – 1.19 (m, 6H). ^{13}C NMR (100 MHz, Chloroform-*d*) δ 202.4, 172.4, 143.9, 133.4, 129.4, 128.6, 60.6, 37.6, 37.1, 21.7, 18.0, 14.2. HRMS (ESI) Calculated for $C_{14}H_{19}O_3^+$ ([M+H]$^+$): 235.1329, found: 235.1330.

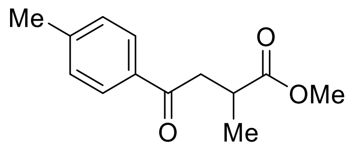

methyl 2-methyl-4-oxo-4-(*p*-tolyl)butanoate **3tT**

The reaction was carried out according to the general procedure on 0.2 mmol scale (48 h). The residue was purified by flash column chromatography (petroleum ether: ethyl acetate, 10/1-4/1) to afford **3tT**, (80%, 35.2 mg) as colorless oil. ^{1}H NMR (400 MHz, Chloroform-*d*) δ 7.87 (d, *J* = 8.2 Hz, 2H), 7.26 (d, *J* = 8.0 Hz, 2H), 3.70 (s, 3H), 3.46 (dd, *J* = 17.5, 7.8 Hz, 1H), 3.17 – 3.08 m, 1H), 3.01 (dd, *J* = 17.5, 5.5 Hz, 1H), 2.41 (s, 3H), 1.27 (d, *J* = 7.1 Hz, 3H). ^{13}C NMR (100 MHz, Chloroform-*d*) δ 197.6, 176.5, 144.0, 134.2, 129.3, 128.2, 51.9, 41.9, 34.9, 21.6, 17.3. HRMS (ESI) Calculated for $C_{13}H_{17}O_3^+$ ([M+H]$^+$): 221.1172, found: 221.1173.

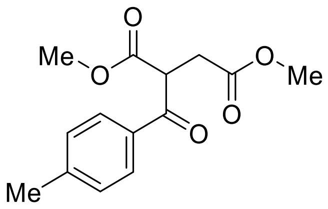

dimethyl 2-(4-methylbenzoyl)succinate **3uU**

The reaction was carried out according to the general procedure on 0.2 mmol scale (48 h). The residue was purified by flash column chromatography (petroleum ether: ethyl acetate, 10/1-2/1) to afford **3uU**, (86%, 45.4 mg) as colorless oil. ^{1}H NMR (400 MHz, Chloroform-*d*) δ 7.94 (d, *J* = 8.2 Hz, 2H), 7.29 (d, *J* = 8.2 Hz, 2H), 4.87 (t, *J* = 7.2 Hz, 1H), 3.68 (s, 3H), 3.68 (s, 3H), 3.23 – 3.01 (m, 2H), 2.42 (s, 3H). ^{13}C NMR (100 MHz, Chloroform-*d*) δ 193.6, 171.8, 169.3, 144.8, 133.2, 129.5, 129.1, 52.8, 52.1, 49.2, 33.1,

21.7. HRMS (ESI) Calculated for $C_{14}H_{17}O_5^+$ ($[M+H]^+$): 265.1071, found: 265.1074.

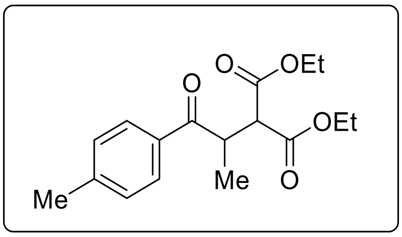

diethyl 2-(1-oxo-1-(*p*-tolyl)propan-2-yl)malonate **3vV**

The reaction was carried out according to the general procedure on 0.2 mmol scale (48 h). The residue was purified by flash column chromatography (petroleum ether: ethyl acetate, 40/1-10/1) to afford **3vV** (51.4 mg, 84%) as colorless oil. ^{1}H NMR (400 MHz, Chloroform-*d*) δ 7.91 (d, *J* = 8.2 Hz, 2H), 7.28 (d, *J* = 8.2 Hz, 2H), 4.32 – 4.05 (m, 5H), 3.98 (d, *J* = 10.8 Hz, 1H), 2.42 (s, 3H), 1.32 (t, *J* = 7.1 Hz, 3H), 1.21 – 1.11 (m, 6H). ^{13}C NMR (100 MHz, Chloroform-*d*) δ 201.2, 168.9, 168.4, 144.1, 133.0, 129.4, 128.7, 61.6, 54.9, 40.4, 21.7, 16.0, 14.2, 13.9. HRMS (ESI) Calcd for $C_{17}H_{23}O_5$ $[M+H]^+$: 307.1540, found: 307.1542.

Investigation of Examining Functional Group Compatibility

1a + **2a** + Biomolecules → **3a** (**cat-I** (1 mol%), Ph_3P (1.2 equiv); DCM, pH 7.4 PBS, Blue Leds, rt, 48 h)

entry	conditions	**3a** yield	entry	conditions	**3a** yield
1	*L*(+)-Cysteine (1 eq.) 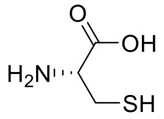	68%	5	naringin (1 eq.) 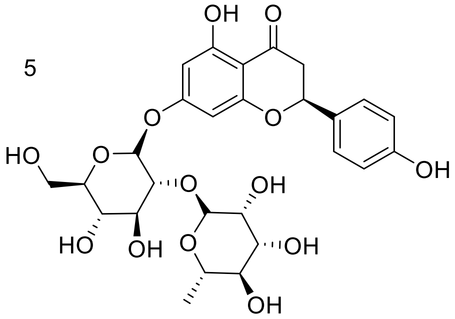	57%
2	*L*-Tyrosine (1 eq.) 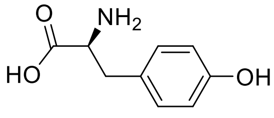	70%	6	10 mg/mL DNA	69%
3	*L*-Methionine (1 eq.) 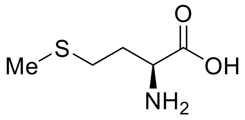	64%	7	10 mg/mL miRNA	62%
4	guanosine (1 eq.) 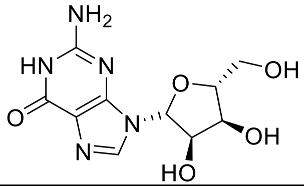	60%	8	10 mg/mL bovine serum albumin	71%

Supplementary Figure 2. Examining functional group compatibility. [a] Reaction conditions: Photocatalyst **I** (1 mol%), **1a** (0.1 mmol, 1.0 equiv), **2a** (0.15 mmol, 1.5 equiv), Ph_3P (0.12 mmol, 1.2 equiv), in 1 mL of 10 X PBS buffers and 1 mL dichloromethane mixed solvent for 48 h under argon with 5 W blue LEDs irradiation at 25 °C.

Experiment Procedure for Examining the Functional Group Compatability: To a solution of aromatic carboxylic acid **1a** (13.6 mg, 0.1 mmol, 1.0 equiv.), photocatalyst $Ir[dF(CF_3)ppy]_2(dtbbpy)PF_6$ (1.2 mg, 1 mol%), 2-vinylpyridine **2a** (15.8 mg, 0.15 mmol, 1.5 equiv.) and Ph_3P (31.5 mg, 0.12 mmol, 1.2 equiv.) in 2 mL pH 7.4 10 X PBS buffer and dichloromethane mixed solvents (V/V = 1:1) was added bimolecules to reach the indicated concentration. The tube was then sealed and was placed at a distance (app. 5 cm) from 5 W blue LEDs lamp, and the mixture was stirred for 48 h at room temperature. After completion, the mixture was quenched with water and extracted with DCM (3 x 10 mL). The combined organic layer was dried over anhydrous Na_2SO_4, then the solvent was removed under vacuo. The residue was purified with chromatography column on silica gel (gradient eluent of petroleum ether/EtOAc: 10/1-4/1) to give the corresponding ketone product **3a.**

In addition, all bimolecules was commercially available.

miRNA:141:5'-UAACACUGUCUGGUAAAGAUGG-3',

DNA: GCAGTCTACCATCTTTACCAGACAGTGTTATAGACTGC-3'.

In addition, amino acids biomolecules can be converted to salts by adding 1M HCl aq. to the reaction mixture after completion. These biomolecules can been proved by ^1H NMR and Mass spectra as shown below. From MS-ESI (negative mode), the added amino acid molecules can be found and its formation was analyzed by ^1H NMR analysis. This implies that added biomolecule remains intact in the reaction mixture.

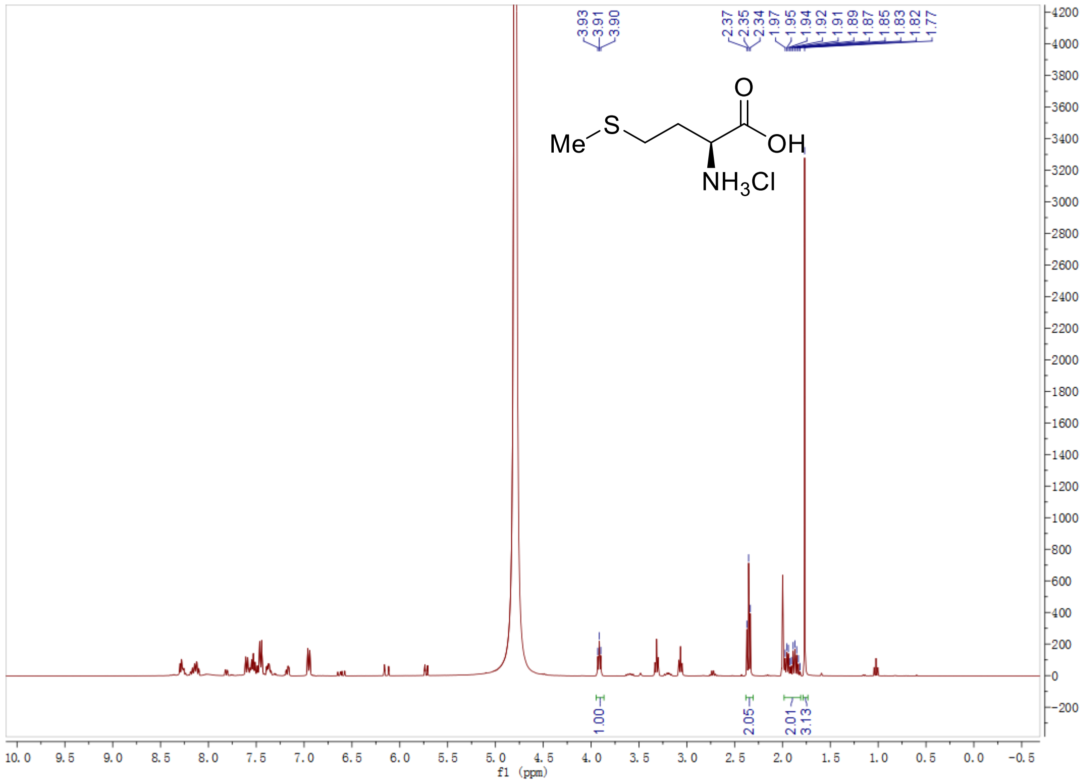

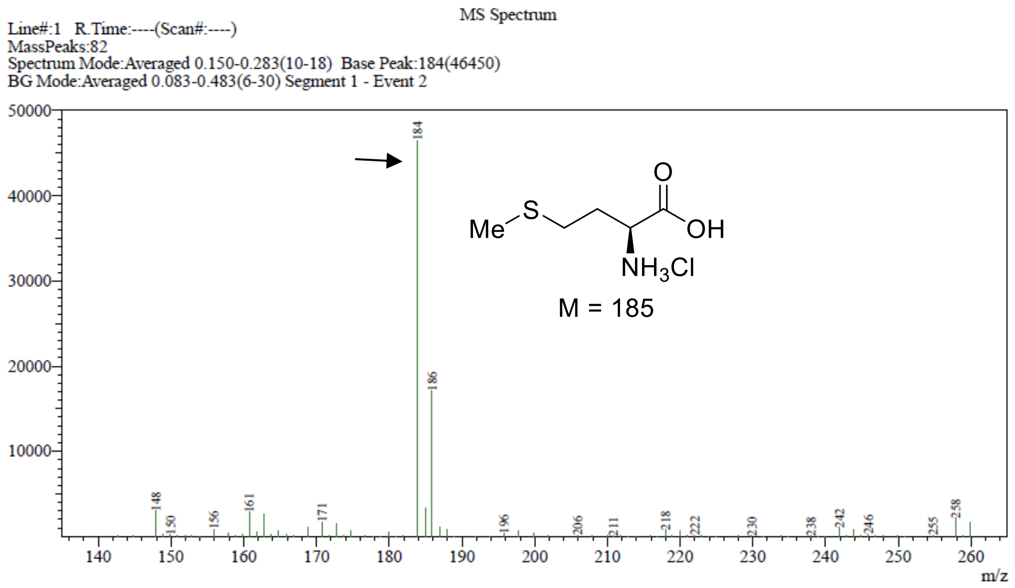

Supplementary Figure 3. ^{1}H NMR and mass spectra for biomolecules

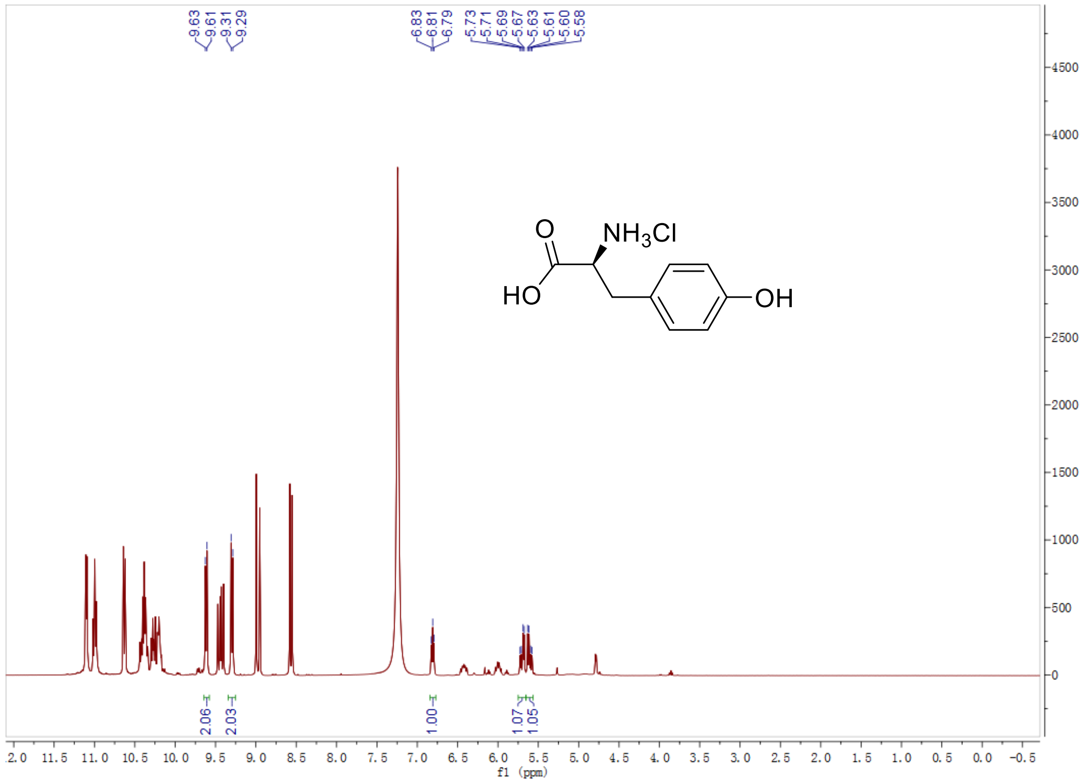

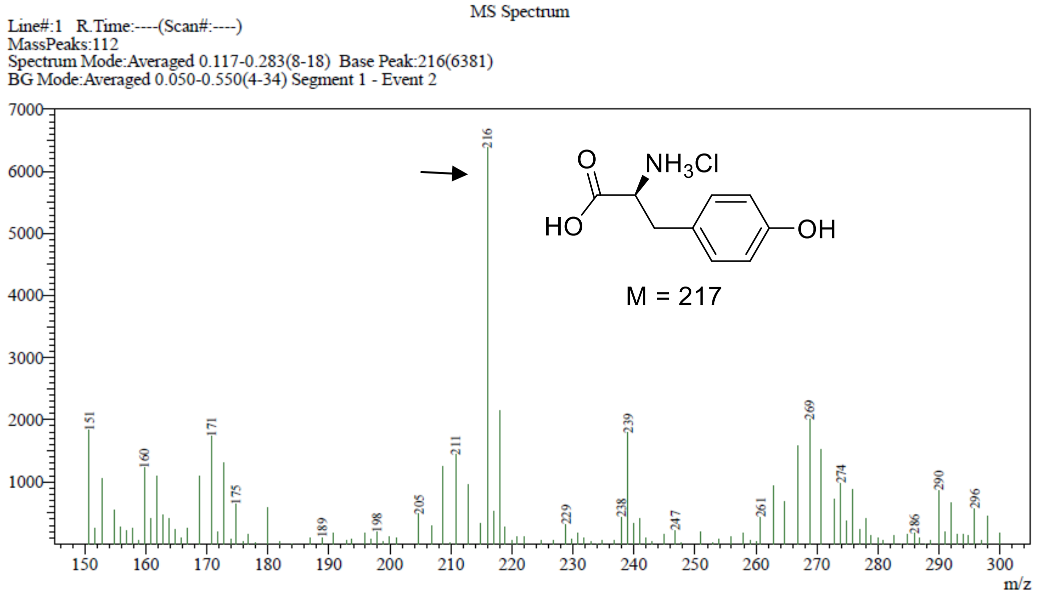

Supplementary Figure 4. ^{1}H NMR and mass spectra for biomolecules

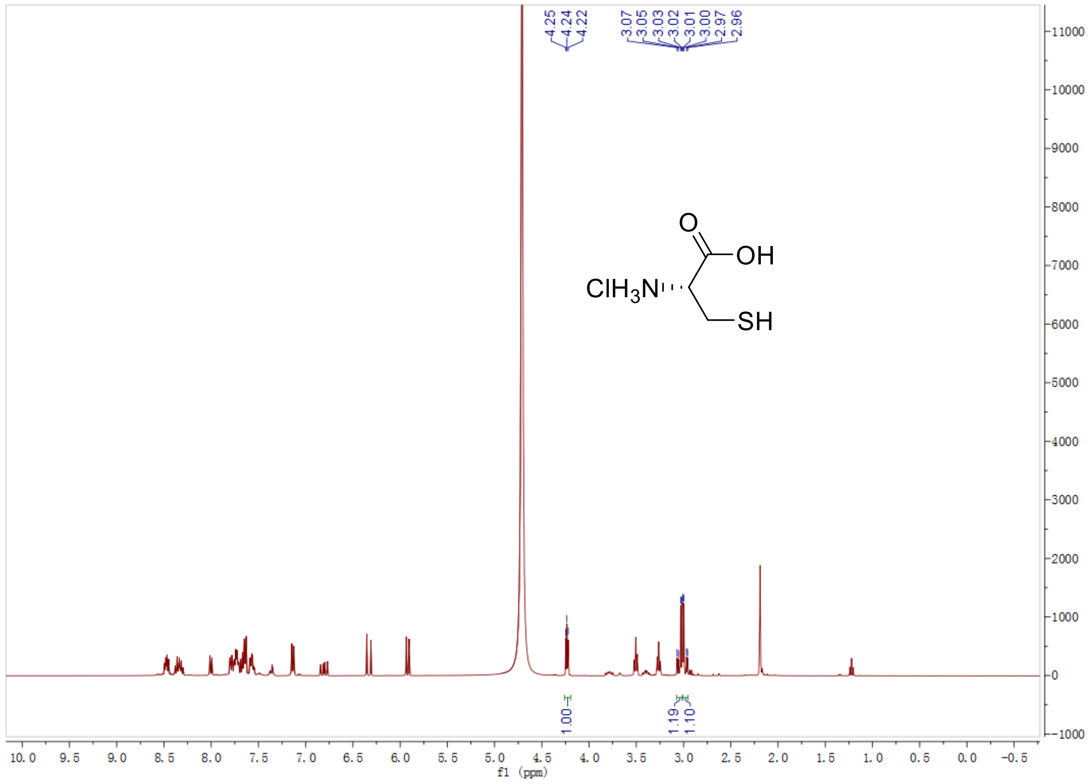

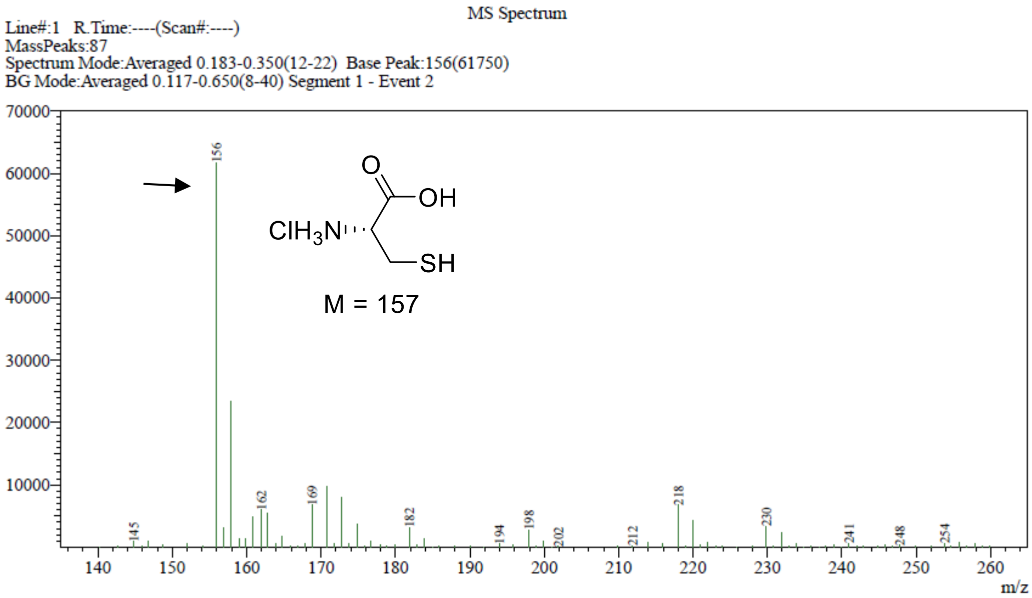

Supplementary Figure 5. ^{1}H NMR and mass spectra for biomolecules

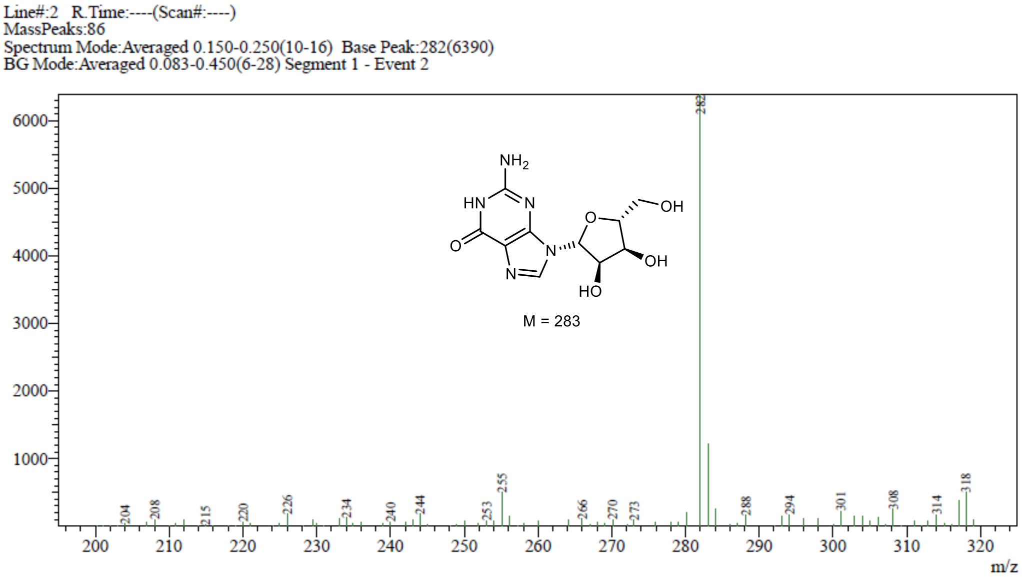

Supplementary Figure 6. mass spectra for biomolecules

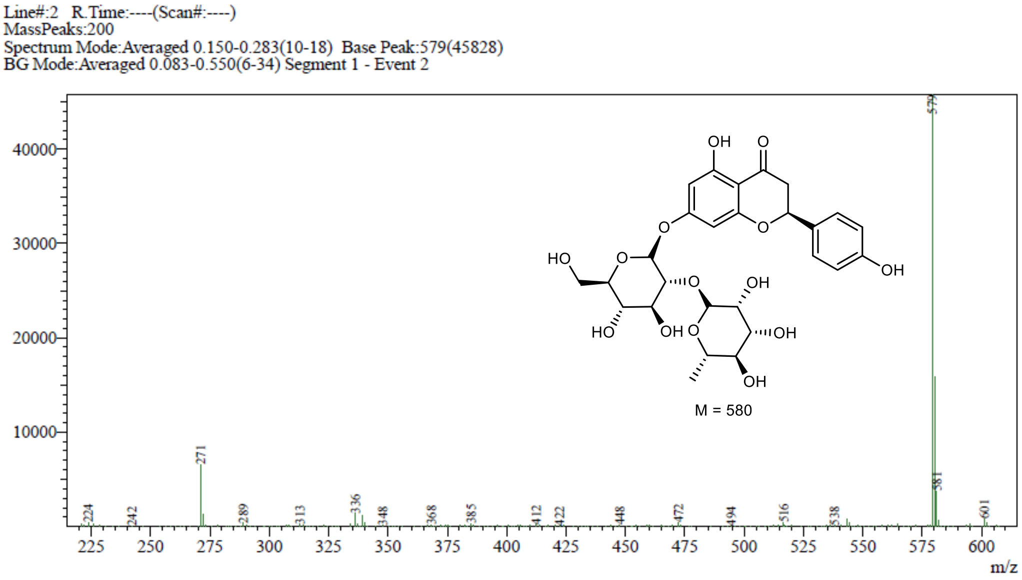

Supplementary Figure 7. mass spectra for biomolecules

General Procedure for Late-Stage Application

General procedure A: To a 10 mL Schlenk tube equipped with a magnetic stir bar was added aromatic carboxylic acid (0.1 mmol, 1.0 equiv.), photocatalyst Ir[dF(CF_3)ppy]$_2$(dtbbpy)PF_6 (2.3 mg, 2 mol%), K_2HPO_4 (3.5 mg, 20 mol%), and Ph_3P (31.5 mg, 0.12 mmol, 1.2 equiv.) and the tube was evacuated and backfilled with Ar (three times). The alkenes (0.15 mmol, 1.5 equiv.) in DCM/H_2O (2.0 mL, 4:1 v/v) were added by syringe under Ar. The tube was then sealed and was placed at a distance (app. 5 cm) from 5 W blue LEDs lamp, and the mixture was stirred for 48 h at room

temperature. After completion, the mixture was quenched with water and extracted with DCM (3 x 10 mL). The combined organic layer was dried over anhydrous Na_2SO_4, then the solvent was removed under vacuo. The residue was purified with chromatography column on silica gel (gradient eluent of petroleum ether/EtOAc) to give the corresponding ketone products.

General procedure B: To a 10 mL Schlenk tube equipped with a magnetic stir bar was added aromatic carboxylic acid (0.15 mmol, 1.5 equiv.), photocatalyst Ir[dF(CF_3)ppy]$_2$(dtbbpy)PF_6 (2.3 mg, 2 mol%), K_2HPO_4 (7.0 mg, 40 mol%), and Ph_3P (31.5 mg, 0.12 mmol, 1.2 equiv.) and the tube was evacuated and backfilled with Ar (three times). The alkenes (0.10 mmol, 1.0 equiv.) in DCM/H_2O (2.0 mL, 4:1 v/v) were added by syringe under Ar. The tube was then sealed and was placed at a distance (app. 5 cm) from 5 W blue LEDs lamp, and the mixture was stirred for 48 h at room temperature. After completion, the mixture was quenched with water and extracted with DCM (3 x 10 mL). The combined organic layer was dried over anhydrous Na_2SO_4, then the solvent was removed under vacuo. The residue was purified with chromatography column on silica gel (gradient eluent of petroleum ether/EtOAc) to give the corresponding ketone products.

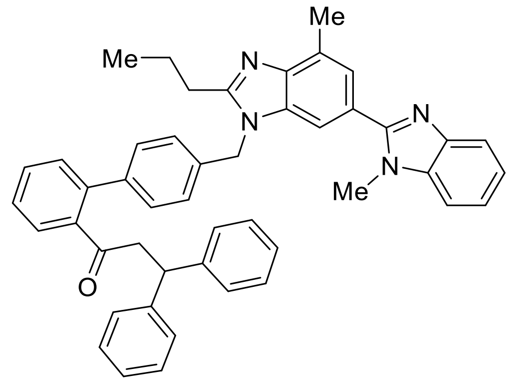

Telmisartan, **4**

The reaction was carried out according to the general procedure A on 0.1 mmol scale (48 h). The residue was purified by flash column chromatography (petroleum ether: ethyl acetate, 10/1-2/1) to afford **4**, (58%, 39.4 mg) as a white solid, mp: 90 − 92 °C. ^{1}H NMR (400 MHz, Chloroform-*d*) δ 7.83 – 7.79 (m, 1H), 7.48 – 7.39 (m, 3H), 7.34 – 7.24 (m, 5H), 7.20 (d, *J* = 7.6 Hz, 1H), 7.12 – 6.92 (m, 14H), 5.38 (s, 2H), 4.44 (t, *J* =

7.6 Hz, 1H), 3.70 (s, 3H), 3.18 (d, *J* = 7.6 Hz, 2H), 2.93 – 2.86 (m, 2H), 2.78 (s, 3H), 1.96 – 1.72 (m, 2H), 1.02 (t, *J* = 7.4 Hz, 3H). ^{13}C NMR (100 MHz, Chloroform-*d*) δ 204.3, 156.4, 154.7, 143.4, 143.2, 142.9, 140.3 (2C), 139.4, 136.7, 135.2, 135.1, 130.7, 130.4, 129.5 (2C), 128.4, 127.9, 127.7, 127.5, 126.4, 124.0, 123.9, 122.5, 122.3, 119.6, 109.6, 108.8, 48.6, 47.0, 46.5, 31.8, 29.8, 21.9, 17.0, 14.1. HRMS (ESI) Calculated for $C_{47}H_{43}N_4O^+$ ([M+H]$^+$): 679.3431, found: 679.3432.

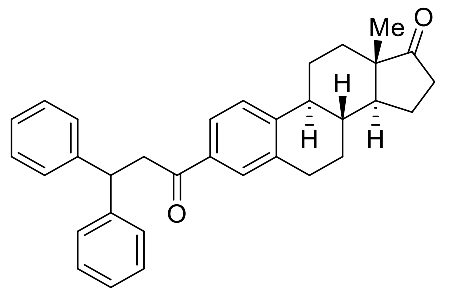

Hiestrone, **5**

The reaction was carried out according to the general procedure A on 0.1 mmol scale (48 h). The residue was purified by flash column chromatography (petroleum ether: ethyl acetate, 10/1-4/1) to afford **5**, (53%, 24.5 mg) as a white solid, mp: 151 − 153 °C . ^{1}H NMR (400 MHz, Chloroform-*d*) δ 7.72 (d, *J* = 8.2 Hz, 1H), 7.67 (s, 1H), 7.36 (d, *J* = 8.2 Hz, 1H), 7.26 (d, *J* = 4.2 Hz, 8H), 7.20 – 7.12 (m, 2H), 4.83 (t, *J* = 7.3 Hz, 1H), 3.70 (d, *J* = 7.3 Hz, 2H), 2.99 – 2.83 (m, 2H), 2.62 – 2.39 (m, 2H), 2.38 – 2.25 (m, 1H), 2.17 – 1.98 (m, 4H), 1.65 – 1.42 (m, 6H), 0.91 (s, 3H). ^{13}C NMR (100 MHz, Chloroform-*d*) δ 220.6, 197.8, 145.5, 144.3, 137.0, 134.7, 128.8, 128.6, 127.9, 126.4, 125.6, 125.5, 50.5, 47.9, 45.9, 44.7 (2C), 37.8, 35.8, 31.6, 29.3, 26.3, 25.6, 21.6, 13.8. HRMS (ESI) Calculated for $C_{33}H_{35}O_2^+$ ([M+H]$^+$): 463.2632, found: 463.2634.

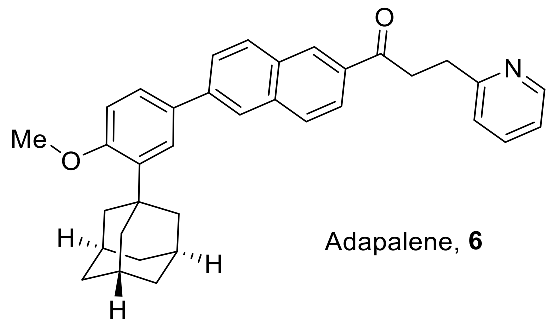

Adapalene, **6**

The reaction was carried out according to the general procedure A on 0.1 mmol scale

(48 h). The residue was purified by flash column chromatography (petroleum ether: ethyl acetate, 10/1-4/1) to afford **6**, (48%, 24.0 mg) as a white solid, mp: 246 – 248 °C. ^{1}H NMR (400 MHz, Chloroform-*d*) δ 8.56 (d, *J* = 4.3 Hz, 1H), 8.53 (s, 1H), 8.06 (d, *J* = 10.1 Hz, 1H), 7.99 (d, *J* = 10.5 Hz, 2H), 7.91 (d, *J* = 8.6 Hz, 1H), 7.80 (d, *J* = 6.9 Hz, 1H), 7.68 – 7.49 (m, 3H), 7.33 (d, *J* = 7.8 Hz, 1H), 7.19 – 7.11 (m, 1H), 7.00 (d, *J* = 8.5 Hz, 1H), 3.90 (s, 3H), 3.67 (t, *J* = 7.3 Hz, 2H), 3.33 (t, *J* = 7.3 Hz, 2H), 2.18 (s, 6H), 2.10 (s, 3H), 1.80 (s, 6H). ^{13}C NMR (100 MHz, Chloroform-*d*) δ 199.1, 160.7, 159.0 , 148.9, 141.6, 139.0, 136.8, 136.0, 133.7, 132.5, 131.3, 130.0, 129.7, 128.5, 126.5, 126.0, 125.7, 124.7, 124.2, 123.7, 121.4, 112.1, 55.2, 40.6, 37.9, 37.2, 37.1, 32.1, 29.1. HRMS (ESI) Calculated for $C_{35}H_{36}NO_2^+$ ($[M+H]^+$): 502.2741, found: 502.2742.

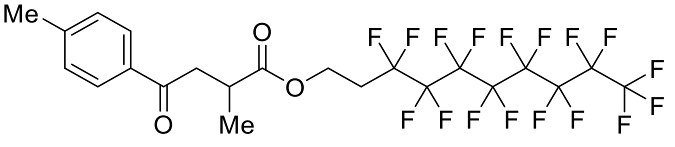

HDFDMA, **7**

The reaction was carried out according to the general procedure A on 0.1 mmol scale (48 h). The residue was purified by flash column chromatography (petroleum ether: ethyl acetate, 10/1-4/1) to afford **7**, (70%, 45.6 mg) as a white solid, mp: 90 − 92 °C. ^{1}H NMR (400 MHz, Chloroform-*d*) δ 7.86 (d, *J* = 8.2 Hz, 2H), 7.26 (d, *J* = 8.2 Hz, 2H), 4.47 – 4.35 (m, 2H), 3.45 (dd, *J* = 17.4, 8.0 Hz, 1H), 3.16 – 2.99 (m, 2H), 2.57 – 2.43 (m, 2H), 2.40 (s, 3H), 1.28 (d, *J* = 7.1 Hz, 3H). ^{13}C NMR (100 MHz, Chloroform-*d*) δ 197.4, 175.6, 144.1, 134.1, 129.3, 128.1, 122.00 – 107.44 (m, 8C), 56.4 (t, *J* = 4.3 Hz), 41.7, 34.9, 30.4 (t, *J* = 21.7 Hz), 21.5, 17.0. HRMS (ESI) Calculated for $C_{22}H_{18}F_{17}O_3^+$ ($[M+H]^+$): 653.0979, found: 653.0980.

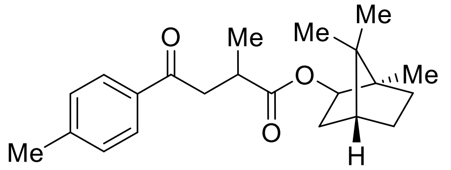

Acryester IBX, **8**

The reaction was carried out according to the general procedure A on 0.1 mmol scale (48 h). The residue was purified by flash column chromatography (petroleum ether: ethyl acetate, 10/1-4/1) to afford **8**, (74%, 25.3 mg) yellowish oil. ^{1}H NMR (400 MHz, Chloroform-*d*) δ 7.86 (d, *J* = 8.2 Hz, 2H), 7.32 – 6.98 (m, 2H), 4.82 – 4.29 (m, 1H), 3.46 (dd, *J* = 7.4, 4.5 Hz, 0.45H), 3.41 (dd, *J* = 7.4, 4.5 Hz, 0.55H), 3.15 – 3.05 (m, 1H), 3.02 – 2.90 (m, 1H), 2.40 (s, 3H), 1.89 – 1.77 (m, 1H), 1.75 – 1.64 (m, 2H), 1.59 – 1.49 (m, 1H), 1.29 – 1.25 (m, 3H), 1.19 – 1.03 (m, 3H), 0.80 - 0.78 (m, 3H), 0.69-0.65 (m, 6H). ^{13}C NMR (100 MHz, Chloroform-*d*) δ 197.7, 197.6, 175.3, 175.2, 143.9, 134.3 (2C), 129.2, 128.1(2C), 81.2, 81.1, 48.7, 48.6, 46.9 (2C), 45.0, 41.8, 41.7, 38.8, 38.7, 35.3 (2C), 33.8, 27.1, 27.0, 21.7, 20.1, 20.0, 19.9, 17.4, 17.3, 11.5, 11.4.

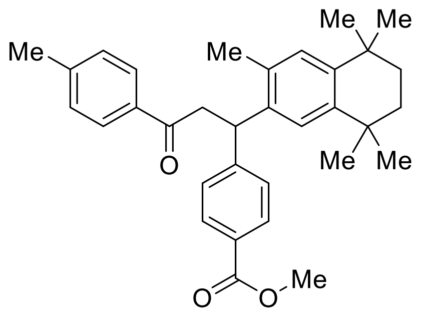

Bexarotene, **9**

The reaction was carried out according to the general procedure B on 0.1 mmol scale (48 h). The residue was purified by flash column chromatography (petroleum ether: ethyl acetate, 10/1-4/1) to afford **9**, (71%, 34.2 mg) as a white solid, mp: 191 − 193 °C. ^{1}H NMR (400 MHz, Chloroform-*d*) δ 7.91 (d, *J* = 8.4 Hz, 2H), 7.82 (d, *J* = 8.2 Hz, 2H), 7.28 (d, *J* = 8.3 Hz, 2H), 7.23 (d, *J* = 8.0 Hz, 2H), 7.12 (s, 1H), 7.01 (s, 1H), 4.99 (t, *J* = 8.1 Hz, 1H), 3.86 (s, 3H), 3.73 – 3.56 (m, 2H), 2.39 (s, 3H), 2.22 (s, 3H), 1.63 (s, 4H), 1.23 (s, 6H), 1.19 (s, 3H), 1.14 (s, 3H). ^{13}C NMR (100 MHz, Chloroform-*d*) δ 197.8, 167.0, 149.6, 144.0, 142.9, 142.4, 138.0, 134.6, 133.1, 129.7, 129.3, 128.7, 128.2 (2C), 128.0, 124.6, 52.0, 44.6, 42.4, 35.4, 34.0, 33.8, 32.0, 31.8 (3C), 21.7, 19.5. HRMS (ESI) Calculated for $C_{33}H_{39}O_3^+$ ([M+H]$^+$): 483.2894, found: 483.2895.

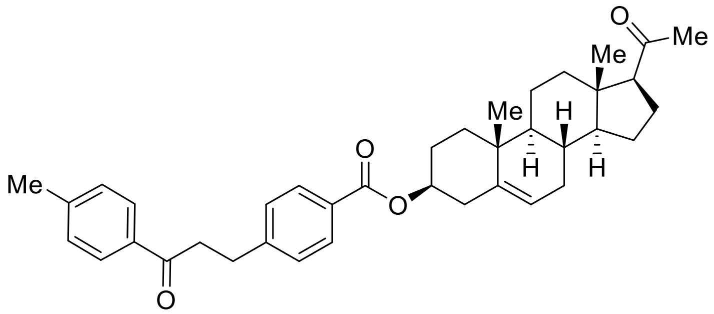

Pregnenolone, **10**

The reaction was carried out according to the general procedure B on 0.1 mmol scale (48 h). The residue was purified by flash column chromatography (petroleum ether: ethyl acetate, 10/1-4/1) to afford **10**, (76%, 43.0 mg), as a white solid, mp: 214 − 216 °C. ^{1}H NMR (400 MHz, Chloroform-*d*) δ 7.97 (d, *J* = 8.2 Hz, 2H), 7.85 (d, *J* = 8.2 Hz, 2H), 7.31 (d, *J* = 8.3 Hz, 2H), 7.25 (d, *J* = 8.3 Hz, 2H), 5.41 (d, *J* = 3.9 Hz, 1H), 4.98 – 4.73 (m, 1H), 3.29 (t, *J* = 7.5 Hz, 2H), 3.11 (t, *J* = 7.5 Hz, 2H), 2.54 (t, *J* = 8.9 Hz, 1H), 2.46 (d, *J* = 7.7 Hz, 2H), 2.40 (s, 3H), 2.19 (d, *J* = 9.5 Hz, 1H), 2.13 (s, 3H), 2.09 – 1.87 (m, 4H), 1.77 – 1.59 (m, 5H), 1.54 – 1.41 (m, 4H), 1.32 – 1.14 (m, 3H), 1.07 (s, 3H), 0.64 (s, 3H). ^{13}C NMR (100 MHz, Chloroform-*d*) δ 209.6, 198.3, 165.9, 146.8, 144.0, 139.7, 134.2, 129.8, 129.3, 128.7, 128.4, 128.1, 122.4, 74.3, 63.7, 56.8, 49.9, 44.0, 39.8, 38.8, 38.2, 37.1, 36.7, 31.8 (2C), 31.6, 30.1, 27.9, 24.5, 22.8, 21.7, 21.1, 19.4, 13.3. HRMS (ESI) Calculated for $C_{38}H_{47}O_4^+$ ($[M+H]^+$): 567.3469, found: 567.3471.

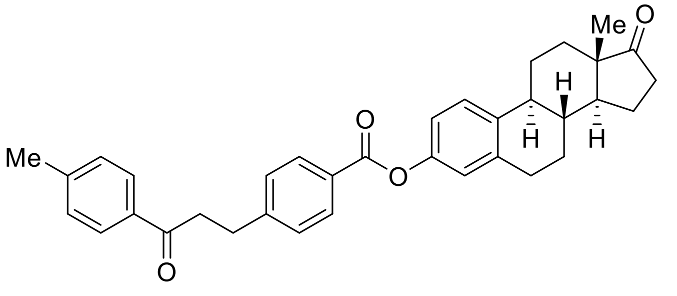

Esterone, **11**

The reaction was carried out according to the general procedure B on 0.1 mmol scale (48 h). The residue was purified by flash column chromatography (petroleum ether: ethyl acetate, 10/1-4/1) to afford **11**, (53%, 27.6 mg), as a white solid, mp: 180 − 182 °C.

^{1}H NMR (400 MHz, Chloroform-*d*) δ 8.11 (d, *J* = 8.3 Hz, 2H), 7.86 (d, *J* = 8.5 Hz, 2H), 7.38 (d, *J* = 8.3 Hz, 2H), 7.32 (d, *J* = 8.5 Hz, 1H), 7.26 (d, *J* = 3.7 Hz, 2H), 6.97 (d, *J* = 8.4 Hz, 1H), 6.93 (s, 1H), 3.32 (t, *J* = 7.4 Hz, 2H), 3.15 (t, *J* = 7.4 Hz, 2H), 2.96 – 2.87 (m, 2H), 2.51 (dd, *J* = 18.8, 8.6 Hz, 1H), 2.41 (s, 3H), 2.31 (t, *J* = 10.4 Hz, 1H), 2.20 – 2.12 (m, 1H), 2.11 – 1.94 (m, 4H), 1.68 – 1.58 (m, 2H), 1.56 – 1.40 (m, 4H), 0.92 (s, 3H). ^{13}C NMR (100 MHz, Chloroform-*d*) δ 220.9, 198.3, 165.4, 148.9, 147.7, 144.1, 138.1, 137.4, 134.2, 130.4, 129.4, 128.7, 128.2, 127.6, 126.5, 121.8, 118.9, 50.5, 48.0, 44.2, 39.7, 38.0, 35.9, 31.6, 30.2, 29.5, 26.4, 25.8, 21.7, 21.6, 13.9. HRMS (ESI) Calculated for $C_{35}H_{37}O_4^+$ ($[M+H]^+$): 521.2686, found: 521.2687.

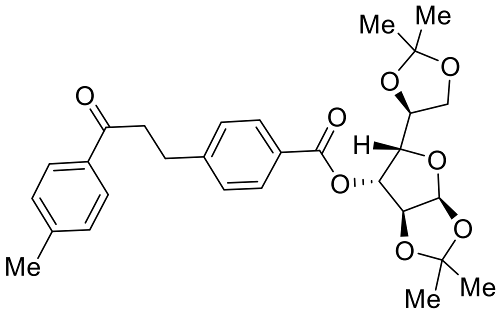

Diacetone-D-glucose, **12**

The reaction was carried out according to the general procedure B on 0.1 mmol scale (48 h). The residue was purified by flash column chromatography (petroleum ether: ethyl acetate, 10/1-2/1) to afford **12**, (63%, 32.1 mg), as a white solid, mp: 236 – 238 °C. ^{1}H NMR (400 MHz, Chloroform-*d*) δ 7.95 (d, *J* = 8.2 Hz, 2H), 7.85 (d, *J* = 8.2 Hz, 2H), 7.34 (d, *J* = 8.2 Hz, 2H), 7.25 (d, *J* = 8.0 Hz, 2H), 5.95 (d, *J* = 3.7 Hz, 1H), 5.49 (d, *J* = 2.5 Hz, 1H), 4.62 (d, *J* = 3.7 Hz, 1H), 4.44 – 4.27 (m, 1H), 4.13 – 4.07 (m, 1H), 3.30 (t, *J* = 7.3 Hz, 2H), 3.13 (t, *J* = 7.4 Hz, 2H), 2.40 (s, 3H), 1.56 (s, 3H), 1.42 (s, 3H), 1.32 (s, 3H), 1.27 (s, 3H). ^{13}C NMR (100 MHz, Chloroform-*d*) δ 198.2, 165.1, 147.7, 144.1, 134.2, 130.0, 129.3, 128.7, 128.1, 127.4, 112.3, 109.4, 105.1, 83.4, 80.0, 76.5, 72.6, 67.2, 39.6, 30.1, 26.8 (2C), 26.2, 25.2, 21.7. HRMS (ESI) Calculated for $C_{29}H_{35}O_8^+$ ($[M+H]^+$): 511.2326, found: 511.2327.

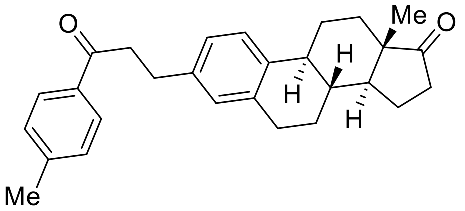

Esterone, **13**

The reaction was carried out according to the general procedure B on 0.1 mmol scale (48 h). The residue was purified by flash column chromatography (petroleum ether: ethyl acetate, 10/1-4/1) to afford **13**, (40%, 16.0 mg), as a white solid, mp: 158 − 160 °C. ^{1}H NMR (400 MHz, Chloroform-*d*) δ 7.86 (d, *J* = 8.2 Hz, 2H), 7.30 – 7.18 (m, 3H), 7.05 (d, *J* = 8.0 Hz, 1H), 7.00 (s, 1H), 3.27 (t, *J* = 8.5 Hz, 2H), 3.00 (t, *J* = 8.5 Hz, 2H), 2.93 – 2.86 (m, 2H), 2.51 (dd, *J* = 18.8, 8.5 Hz, 1H), 2.41 (s, 3H), 2.31 – 2.28 (m, 1H), 2.21 – 2.12 (m, 1H), 2.10 – 1.91 (m, 3H), 1.71 – 1.38 (m, 7H), 0.91 (s, 3H). ^{13}C NMR (100 MHz, Chloroform-*d*) δ 221.0, 199.0, 143.8, 138.9, 137.6, 136.6, 134.4, 129.3, 129.1 , 128.2, 125.9, 125.5, 50.5, 48.0, 44.3, 40.4, 38.2, 35.9, 31.6, 29.7, 29.4, 26.6, 25.8, 21.7, 21.6, 13.9. HRMS (ESI) Calculated for $C_{28}H_{33}O_2^+$ ($[M+H]^+$): 401.2475, found: 401.2477.

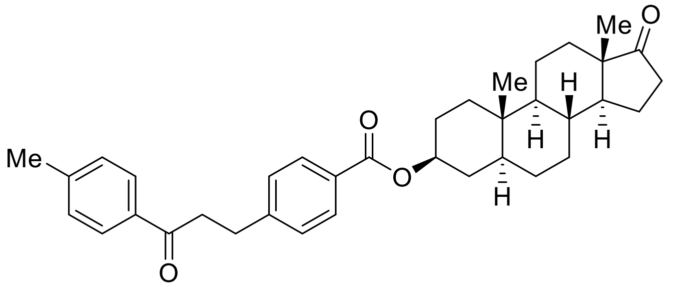

Epiandrosterone, **14**

The reaction was carried out according to the general procedure B on 0.1 mmol scale (48 h). The residue was purified by flash column chromatography (petroleum ether: ethyl acetate, 10/1-4/1) to afford **14**, (71%, 38.3 mg), as a white solid, mp: 248 − 250 °C . ^{1}H NMR (400 MHz, Chloroform-*d*) δ 7.96 (d, *J* = 8.2 Hz, 2H), 7.85 (d, *J* = 8.2 Hz, 2H), 7.31 (d, *J* = 8.3 Hz, 2H), 7.25 (d, *J* = 8.3 Hz, 2H), 5.00 – 4.79 (m, 1H), 3.28 (t, *J* = 7.5 Hz, 2H), 3.11 (t, *J* = 7.5 Hz, 2H), 2.50 – 2.42 (m, 1H), 2.40 (s, 3H), 2.13 – 2.01 (m, 1H), 1.98 – 1.90 (m, 2H), 1.84 – 1.72 (m, 4H), 1.69 – 1.46 (m, 5H), 1.38 – 1.21 (m, 6H),

1.14 – 0.99 (m, 2H), 0.90 (s, 3H), 0.87 (s, 3H), 0.79 – 0.73 (m, 1H). ^{13}C NMR (100 MHz, Chloroform-*d*) δ 221.3, 198.3, 166.0, 146.7, 144.0, 134.2, 129.8, 129.3, 128.8, 128.4, 128.1, 74.0, 54.3, 51.4, 47.8, 44.7, 39.8, 36.8, 35.9, 35.7, 35.1, 34.1, 31.5, 30.8, 30.1, 28.3, 27.5, 21.8, 21.7, 20.5, 13.8, 12.3. HRMS (ESI) Calculated for $C_{36}H_{45}O_4^+$ ([M+H]$^+$): 541.3312, found: 541.3313.

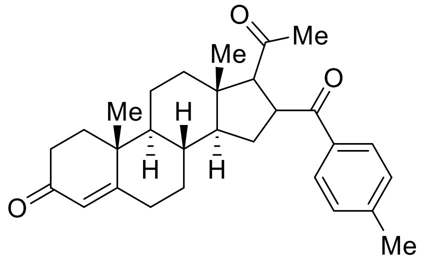

16-Dehydroprogesterone, **15**

The reaction was carried out according to the general procedure B on 0.1 mmol scale (48 h). The residue was purified by flash column chromatography (petroleum ether: ethyl acetate, 10/1-4/1) to afford **15**, (30.2 mg, 70%, d.r. = 4.5:1). Major product (*trans* diastereomer)：^{1}H NMR (400 MHz, Chloroform-*d*) δ 7.86 (d, *J* = 8.2 Hz, 2H), 7.26 (d, *J* = 8.2 Hz, 2H), 5.71 (s, 1H), 4.47 – 4.18 (m, 1H), 3.37 (d, *J* = 8.6 Hz, 1H), 2.41 (s, 3H), 2.40 – 2.29 (m, 2H), 2.26 – 2.21 (m, 1H), 2.17 (s, 3H), 2.13 – 2.00 (m, 2H), 1.86 (q, *J* = 12.5 Hz, 1H), 1.77 – 1.60 (m, 6H), 1.58 – 1.42 (m, 2H), 1.37 – 1.32 (m, 1H), 1.19 (s, 3H), 1.08 – 0.94 (m, 2H), 0.80 (s, 3H). ^{13}C NMR (100 MHz, Chloroform-*d*) δ 208.0, 201.2, 199.3, 170.4, 143.9, 133.6, 129.3, 128.9, 124.1, 64.7, 54.6, 53.3, 44.5, 44.4, 38.5, 35.7, 35.3, 33.9, 32.6, 31.6, 31.0, 21.7, 21.0, 17.4, 14.3. HRMS (ESI) Calculated for $C_{29}H_{37}O_3^+$ ([M+H]$^+$): 433.2737, found: 433.2739. Minor product (*cis* diastereomer)：^{1}H NMR (400 MHz, Chloroform-*d*) δ 7.64 (d, *J* = 8.2 Hz, 2H), 7.20 (d, *J* = 8.2 Hz, 2H), 5.72 (s, 1H), 3.90 – 3.76 (m, 1H), 3.42 (d, *J* = 8.0 Hz, 1H), 2.85 – 2.58 (m, 1H), 2.49 – 2.40 (m, 1H), 2.38 (s, 3H), 2.36 – 2.23 (m, 3H), 2.03 – 1.89 (m, 2H), 1.79 (s, 3H), 1.74 – 1.62 (m, 4H), 1.56 – 1.40 (m, 4H), 1.18 (s, 3H), 1.13 (s, 3H), 0.95 – 0.85 (m, 2H). HRMS (ESI) Calculated for $C_{29}H_{37}O_3^+$ ([M+H]$^+$): 433.2737, found: 433.2738.

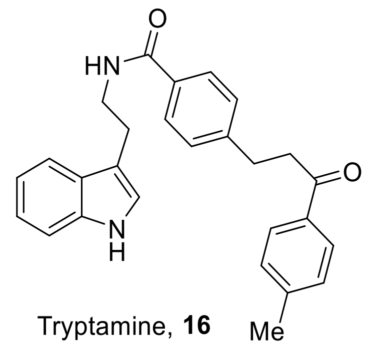

The reaction was carried out according to the general procedure B on 0.1 mmol scale (48 h). The residue was purified by flash column chromatography (petroleum ether: ethyl acetate, 5/1-1/1) to afford **16**, (48%, 19.7 mg), as a white solid, mp: 104 − 106 °C. ^{1}H NMR (400 MHz, Chloroform-*d*) δ 8.15 (br s, 1H), 7.84 (d, *J* = 8.2 Hz, 2H), 7.65 (d, *J* = 7.9 Hz, 1H), 7.59 (d, *J* = 8.2 Hz, 2H), 7.37 (d, *J* = 7.9 Hz, 1H), 7.28 – 7.19 (m, 5H), 7.13 (t, *J* = 7.5 Hz, 1H), 7.06 (s, 1H), 6.20 (br s, 1H), 3.79 (q, *J* = 6.5 Hz, 2H), 3.26 (t, *J* = 7.5 Hz, 2H), 3.12 – 3.05 (m, 4H), 2.40 (s, 3H). ^{13}C NMR (100 MHz, Chloroform-*d*) δ 198.5, 167.3, 145.1, 144.0, 136.5, 134.2, 132.6, 129.3, 128.6, 128.1, 127.3, 127.1, 122.3, 122.1, 119.6, 118.8, 113.1, 111.3, 40.2, 39.8, 29.9, 25.3, 21.7. HRMS (ESI) Calculated for $C_{27}H_{27}N_2O_2^+$ ([M+H]$^+$): 411.2067, found: 411.2067.

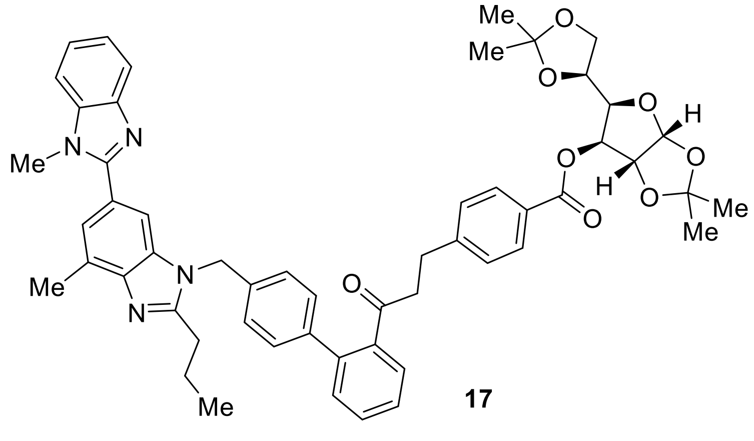

The reaction was carried out according to the general procedure B on 0.1 mmol scale (48 h). The residue was purified by flash column chromatography (petroleum ether: ethyl acetate, 4/1-1/1) to afford **17**, (51%, 45.3 mg) as a white solid, mp: 274 − 276 °C. ^{1}H NMR (400 MHz, Chloroform-*d*) δ 7.83 (d, *J* = 8.2 Hz, 2H), 7.80 – 7.75 (m, 1H),

7.52 (s, 1H), 7.49 – 7.46 (m, 1H), 7.42 (s, 1H), 7.40 – 7.33 (m, 3H), 7.32 – 7.27 (m, 3H), 7.23 (d, *J* = 8.2 Hz, 2H), 7.09 (d, *J* = 8.1 Hz, 2H), 7.02 (d, *J* = 8.2 Hz, 2H), 5.93 (d, *J* = 3.7 Hz, 1H), 5.55 – 5.41 (m, 3H), 4.60 (d, *J* = 3.7 Hz, 1H), 4.33 (t, *J* = 3.9 Hz, 2H), 4.08 (d, *J* = 4.6 Hz, 2H), 3.81 (s, 3H), 2.90 (t, *J* = 7.2 Hz, 2H), 2.79 – 2.75 (m, 5H), 2.63 (t, *J* = 7.2 Hz, 2H), 1.91 – 1.84 (m, 2H), 1.55 (s, 3H), 1.41 (s, 3H), 1.31 (s, 3H), 1.25 (s, 3H), 1.02 (t, *J* = 7.4 Hz, 3H). ^{13}C NMR (100 MHz, Chloroform-*d*) δ 205.4, 165.0, 156.4, 154.6, 146.9, 143.2, 142.9, 140.4, 140.3, 139.3, 136.7, 135.6, 135.1, 130.8, 130.4, 129.8, 129.5, 129.4, 128.5, 127.7 (2C), 127.4, 126.5, 124.0, 123.8, 122.6, 122.4, 119.6, 112.4, 109.5, 109.4, 108.8, 105.1, 83.4, 79.9, 76.5, 72.6, 67.2, 46.9, 43.7, 31.9, 30.4, 29.9, 26.9, 26.8, 26.2, 25.3, 21.8, 16.9. HRMS (ESI) Calculated for $C_{54}H_{57}N_4O_8^+$ ($[M+H]^+$): 889.4171, found: 889.4172.

The Synthesis of Top-Selling Drug Zolpidem

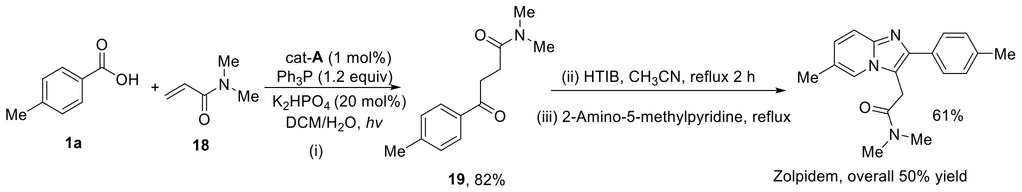

To a 10 mL Schlenk tube equipped with a magnetic stir bar was added carboxylic acid (0.25 mmol, 1 equiv.), photocatalyst Ir[dF(CF$_3$)ppy]$_2$(dtbbpy)PF$_6$ (2.8 mg, 1 mol%), K_2HPO_4 (8.7 mg, 20 mol%), and Ph_3P (78.5 mg, 1.2 equiv.) and the tube was evacuated and backfilled with Ar (three times). The *N,N*-dimethylacrylamide **18** (37.1 mg, 1.5 equiv.) in DCM/H_2O (2.5 mL, 4:1 v/v) were added by syringe under Ar. The tube was then sealed and was placed at a distance (app. 5 cm) from 5 W blue LEDs lamp, and the mixture was stirred for 48 h at room temperature. After completion, the mixture was quenched with water and extracted with DCM (3 x 10 mL). The combined organic layer was dried over anhydrous Na_2SO_4, then the solvent was removed under vacuo. The residue was purified with chromatography column on silica gel (gradient eluent of petroleum ether/EtOAc: 5/1 to 1/1) to give *N,N*-dimethyl-4-oxo-4-(p-tolyl)butanamide **18** (44.9 mg , 82%).^{1}H NMR (400 MHz, Chloroform-*d*) δ 7.92 (d, *J* = 8.2 Hz, 2H), 7.26 (d, *J* = 8.2 Hz, 2H), 3.33 (t, *J* = 6.7 Hz, 2H), 3.09 (s, 3H), 2.96 (s, 3H), 2.77 (t, *J* = 6.7 Hz, 2H), 2.40 (s, 3H). ^{13}C NMR (100 MHz, Chloroform-*d*) δ 199.0, 171.8, 143.8, 134.4,

129.2, 128.2, 37.2, 35.6, 33.6, 27.3, 21.7. HRMS (ESI) Calculated for $C_{13}H_{18}NO_2^+$ ($[M+H]^+$): 220.1332, found: 220.1334.

A solution of *N,N*-dimethyl-4-oxo-4-tolylbutanamide (**18**, 43.8 mg, 0.2 mmol) and hydroxy(tosyloxy)iodo]benzene (HTIB) (78.4 mg, 0.2 mmol) in acetonitrile (5 mL) was refluxed for 2 hrs. After the completion of reaction, 2-amino-5-methylpyridine (21.6 g, 0.2 mmol) was added to the reaction mixture which was further refluxed for 2h. After completion, the mixture was quenched with water and extracted with DCM (3 x 10 mL). The combined organic layer was dried over anhydrous Na_2SO_4, then the solvent was removed under vacuo. The residue was purified with chromatography column on silica gel (gradient eluent of petroleum ether/EtOAc: 4/1 to 1/1) to yield zolpidem as a yellow solid (37.5 mg, 61%), mp.192–194 ºC. ^{1}H NMR (400 MHz, Chloroform-*d*) δ 7.98 (s, 1H), 7.53 (t, *J* = 8.4 Hz, 3H), 7.35 – 7.20 (m, 2H), 7.04 (d, *J* = 9.2 Hz, 1H), 4.07 (s, 2H), 2.94 (s, 3H), 2.88 (s, 3H), 2.39 (s, 3H), 2.33 (s, 3H). ^{13}C NMR (100 MHz, Chloroform-*d*) δ 168.3, 144.2, 143.8, 137.4, 131.8, 129.3, 128.4, 127.5, 122.2, 121.7, 116.6, 113.6, 37.5, 35.9, 30.3, 21.3, 18.5. HRMS (ESI) Calculated for $C_{19}H_{22}N_3O^+$ ($[M+H]^+$): 308.1757, found: 308.1760.

General Procedure for the Deoxygenative Macrocyclization

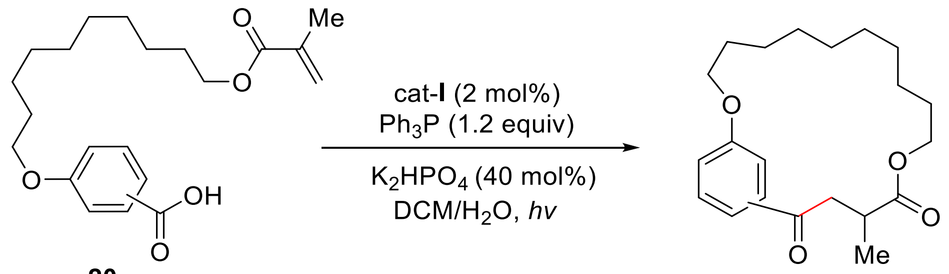

20 21

To a 10 mL Schlenk tube equipped with a magnetic stir bar was added aromatic carboxylic acid **20** (36.2 mg, 0.1 mmol, 1.0 equiv.), photocatalyst Ir[dF(CF_3)ppy]$_2$(dtbbpy)PF_6 (2.3 mg, 2 mol%), K_2HPO_4 (7.0 mg, 40 mol%), and Ph_3P (31.5 mg, 0.12 mmol, 1.2 equiv.) and the tube was evacuated and backfilled with Ar (three times). DCM/H_2O (2.0 mL, 4:1 v/v) were added by syringe under Ar. The tube was then sealed and was placed at a distance (app. 5 cm) from 5 W blue LEDs lamp, and the mixture was stirred for 48 h at room temperature. After completion, the mixture

was quenched with water and extracted with DCM (3 x 10 mL). The combined organic layer was dried over anhydrous Na_2SO_4, then the solvent was removed under vacuo. The residue was purified with chromatography column on silica gel (gradient eluent of petroleum ether/EtOAc, 50/1-20/1) to give the corresponding macrocyclic products.

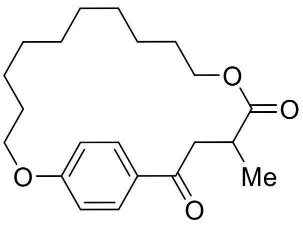

15-methyl-2,13-dioxa-1(1,4)-benzenacycloheptadecaphane-14,17-dione **21a**

The reaction was carried out according to the general procedure on 0.1 mmol scale (48 h). The residue was purified by flash column chromatography (petroleum ether: ethyl acetate, 50/1-20/1) to afford **21a**, (43%, 14.9 mg) as a white solid, mp: 106 - 108 °C. ^{1}H NMR (400 MHz, Chloroform-*d*) δ 7.95 (d, *J* = 8.8 Hz, 2H), 6.97 (d, *J* = 8.8 Hz, 2H), 4.35 – 4.25 (m, 2H), 3.86 (t, *J* = 6.2 Hz, 2H), 3.52 (dd, *J* = 15.9, 10.6 Hz, 1H), 3.24 – 3.16 (m, 1H), 2.66 (dd, *J* = 15.9, 3.6 Hz, 1H), 1.80 – 1.61 (m, 2H), 1.45 – 1.36 (m, 2H), 1.33 (d, *J* = 7.2 Hz, 3H), 1.30 – 1.25 (m, 2H), 1.23 – 1.15 (m, 2H), 1.09 – 1.02 (m, 2H), 0.98 – 0.92 (m, 2H), 0.88 – 0.80 (m, 2H), 0.78 – 0.72 (m, 2H). ^{13}C NMR (100 MHz, Chloroform-*d*) δ 197.1, 175.0, 162.4, 130.5, 129.7, 115.4, 67.2, 64.8, 41.2, 36.5, 29.7, 28.6, 27.6, 27.5, 26.8, 26.5, 26.2, 22.9, 17.6. HRMS (ESI) Calculated for $C_{21}H_{31}O_4^+$ ($[M+H]^+$): 347.2217, found: 347.2218.

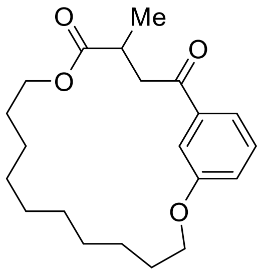

15-methyl-2,13-dioxa-1(1,3)-benzenacycloheptadecaphane-14,17-dione **21b**

The reaction was carried out according to the general procedure on 0.1 mmol scale (48

h). The residue was purified by flash column chromatography (petroleum ether: ethyl acetate, 50/1-20/1) to afford **21b**, (48%, 16.6 mg) as colorless oil. ^{1}H NMR (400 MHz, Chloroform-*d*) δ 7.77 – 7.49 (m, 2H), 7.37 (t, *J* = 8.1 Hz, 1H), 7.11 (dd, *J* = 7.7, 3.0 Hz, 1H), 4.26 – 4.07 (m, 3H), 4.01 – 3.96 (m, 1H), 3.51 (dd, *J* = 17.2, 9.0 Hz, 1H), 3.25 – 3.08 (m, 1H), 2.91 (dd, *J* = 17.2, 4.7 Hz, 1H), 1.84 – 1.66 (m, 2H), 1.52 – 1.46 (m, 3H), 1.41 – 1.35 (m, 2H), 1.31 (d, *J* = 7.2 Hz, 3H), 1.29 – 1.18 (m, 9H). ^{13}C NMR (100 MHz, Chloroform-*d*) δ 197.7, 175.5, 158.8, 137.8, 129.7, 120.9, 120.5, 113.5, 67.7, 64.4, 42.2, 35.6, 28.9, 28.7, 28.3, 27.6, 27.3, 26.7, 25.8, 23.5, 17.2. HRMS (ESI) Calculated for $C_{21}H_{31}O_4^+$ ([M+H]$^+$): 347.2217, found: 347.2219.

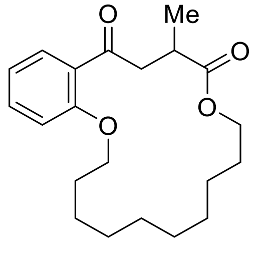

14-methyl-2,3,4,5,6,7,8,9,10,11,14,15-dodecahydrobenzo[*b*][1,8]dioxacyclooctadecine-13,16-dione **21c**

The reaction was carried out according to the general procedure on 0.1 mmol scale (48 h). The residue was purified by flash column chromatography (petroleum ether: ethyl acetate, 50/1-20/1) to afford **21c**, (40%, 13.8 mg) as colorless oil. ^{1}H NMR (400 MHz, Chloroform-*d*) δ 7.74 (dd, *J* = 7.7, 1.8 Hz, 1H), 7.53 – 7.37 (m, 1H), 7.13 – 6.81 (m, 2H), 4.35 – 4.26 (m, 1H), 4.16 – 3.99 (m, 3H), 3.43 (dd, *J* = 17.5, 4.1 Hz, 1H), 3.31 – 3.07 (m, 2H), 1.86 – 1.80 (m, 2H), 1.71 – 1.65 (m, 2H), 1.55 – 1.50 (m, 2H), 1.45 – 1.30 (m, 10H), 1.18 (d, *J* = 6.7 Hz, 3H). ^{13}C NMR (100 MHz, Chloroform-*d*) δ 199.7, 176.3, 158.4, 133.7, 130.5, 128.0, 120.4, 112.3, 68.5, 63.8, 47.4, 35.5, 29.1, 27.9, 27.1 (2C), 26.4, 25.9, 24.6, 16.6. HRMS (ESI) Calculated for $C_{21}H_{31}O_4^+$ ([M+H]$^+$): 347.2217, found: 347.2218.

Organic Transformations to Construct Nitrogen-Containing Heterocycles

Synthesis of 1-(p-tolyl)pyrrolo[2,1-a]isoquinoline

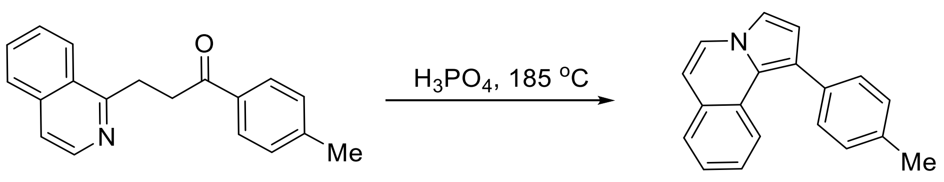

3jj **22**

Following the modified procedure of reported literature,[1] a solution of 3-(isoquinolin-1-yl)-1-(p-tolyl)propan-1-one **3jj** (0.2 mmol, 55.2 mg) in 2 mL of 100% phosphoric acid was heated at 185 ºC for 30 minutes. When the mixture was cooled and was quenched with ice water and extracted with EtOAc (3 x 10 mL). The combined organic layer was dried over anhydrous Na_2SO_4, then the solvent was removed under vacuo. The residue was purified with chromatography column on silica gel (gradient eluent of petroleum ether/EtOAc: 40/1 to 20/1) to give the corresponding 1-(p-tolyl)pyrrolo[2,1-a]isoquinoline **22** as a white solid (90%, 46.3 mg), mp: 165 − 167 °C. ^{1}H NMR (400 MHz, Chloroform-*d*) δ 8.05 (dd, *J* = 17.8, 7.7 Hz, 2H), 7.56 (d, *J* = 7.8 Hz, 1H), 7.49 (d, *J* = 8.0 Hz, 3H), 7.34 (dd, *J* = 16.5, 7.9 Hz, 3H), 7.06 (d, *J* = 3.8 Hz, 1H), 6.79 (d, *J* = 5.3 Hz, 1H), 6.72 (d, *J* = 7.5 Hz, 1H), 2.46 (s, 3H). ^{13}C NMR (100 MHz, Chloroform-*d*) δ 137.2, 130.6, 129.6, 129.5, 128.5, 128.4, 127.4, 126.7, 126.7, 126.7, 125.5, 122.2, 122.1 (2C), 110.9, 100.2, 21.3. HRMS (ESI) Calculated for $C_{19}H_{16}N^+$ ($[M+H]^+$): 258.1277, found: 258.1278.

Synthesis of 2,4-diphenyl-6-(pyridin-2-yl)pyrimidine

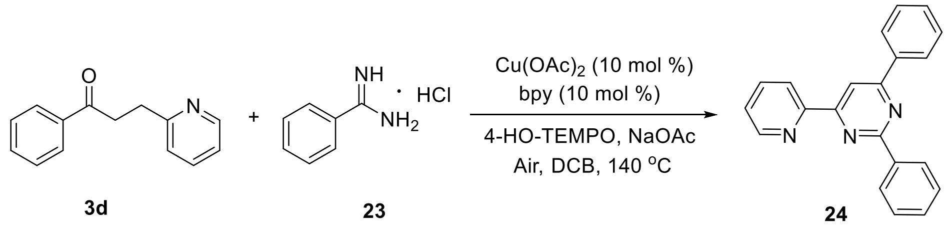

3d **23** **24**

Following the modified procedure of reported literature,[2] to a 10 mL Schlenk tube equipped with a magnetic stir bar was added amidine (15.6 mg, 0.1 mmol, 1 equiv.), $Cu(OAc)_2$ (1.8 mg, 10 mol%), 2,2′-bipyridine (1.58 mg, 10 mol%), 4-HO-TEMPO (17.2 mg, 0.1 mmol), and NaOAc(12.3 mg, 0.15 mmol). Then 1,2-dichlorobenzene (2 mL) and 1-phenyl-3-(pyridin-4-yl)propan-1-one **3d** (42.2 mg, 0.2 mmol) were added to the tube. The tube was then sealed, and the mixture was stirred at 140 °C for 24 h. Upon cooling to room temperature, the reaction mixture was diluted with 10 mL of ethyl

acetate, followed by filtration through a pad of silica gel. The filtrate was concentrated under reduced pressure and then purified by flash chromatography on silica gel (gradient eluent of hexane/ethyl acetate: 40/1 to 20/1) to provide the corresponding product 2,4-diphenyl-6-(pyridin-2-yl)pyrimidine **24** as a white solid (65%, 20.1 mg), mp: 211−213 °C. ^{1}H NMR (400 MHz, Chloroform-*d*) δ 8.87 - 8.66 (m, 5H), 8.51 - 8.33 (m, 2H), 7.92 (td, *J* = 7.7, 1.8 Hz, 1H), 7.65 - 7.49 (m, 6H), 7.47 -7.40 (m, 1H). ^{13}C NMR (100 MHz, Chloroform-*d*) δ 165.2, 164.2, 163.6, 154.7, 149.4, 138.1, 137.4, 137.1, 130.9, 130.7, 128.9, 128.5, 128.4, 127.5, 125.3, 122.0, 110.6. HRMS (ESI) Calculated for $C_{19}H_{16}N^{+}$ ($[M+H]^{+}$): 310.1339, found: 310.1340.

Synthesis tert-butyl (3-(1-(N-(methoxycarbonyl)sulfamoyl)indolizin-3-yl)phenyl)carbamate

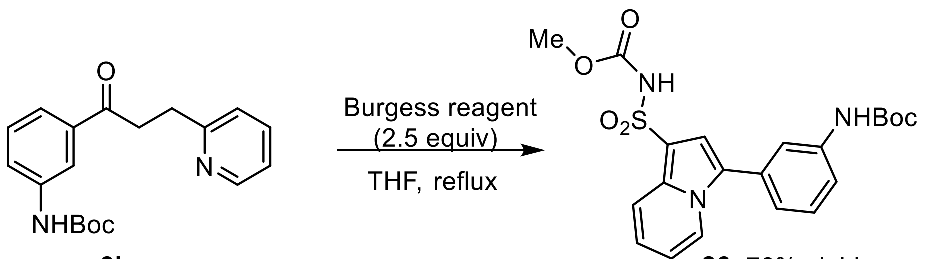

3j **26**, 76% yield

Following the modified procedure of reported literature,[3] to a solution of substrate **3j** (0.1 mmol, 32.6 mg) in THF (2 mL) was added the Burgess reagent **25** (0.25 mmol, 59.5 mg,). The reaction solution was refluxed for 0.5 h and cooled to room temperature. The solvent was removed in vacuo and the residual solid was purified via a flash chromatography eluting with 1:1 hexanes/EtOAc to give the desired product **26** (33.8 mg, 76%) as a crystalline solid. mp =120 - 123 °C. ^{1}H NMR (400 MHz, Chloroform-*d*) δ 8.31 (d, *J* = 7.1 Hz, 1H), 8.18 (d, *J* = 9.1 Hz, 1H), 7.65 (s, 1H), 7.42 (t, *J* = 7.8 Hz, 1H), 7.34 (d, *J* = 8.1 Hz, 1H), 7.25 (s, 1H), 7.19 (d, *J* = 8.6 Hz, 2H), 6.82 (d, *J* = 6.8 Hz, 1H), 6.64 (s, 1H), 3.69 (s, 3H), 1.53 (s, 9H). ^{13}C NMR (100 MHz, Chloroform-*d*) δ 152.7, 151.5, 139.3, 135.1, 130.9, 129.8, 126.7, 123.8, 123.6, 123.3, 118.9, 118.8, 118.6, 115.2, 113.5, 107.2, 81.0, 53.3, 28.3.

Synthesis of 1-(*p*-tolyl)-1, 2, 3, 3a, 4, 5-hexahydropyrrolo[1, 2-a]quinoline

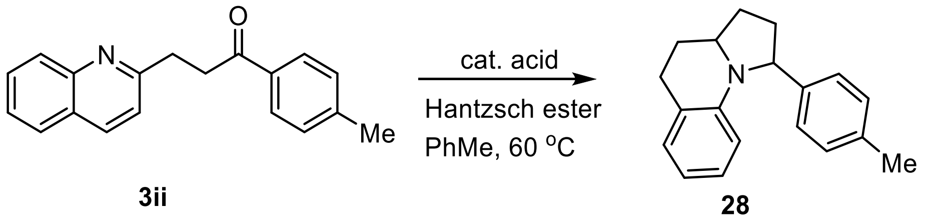

Following the modified procedure of reported literature,[4] to a 10 mL Schlenk tube equipped with a magnetic stir bar was added quinolinylketone **3ii** (0.1 mmol, 27.5 mg, 1 equiv.), Hantzsch ester (83.5 mg, 0.33 mmol, 3.3 equiv.) and *S*-3,3'-Bis(9-anthracenyl)-1,1'-binaphthyl-2,2'-diyl hydrogenphosphate **27** (3.5 mg, 5 mol%). The tube was evacuated and backfilled with Ar (three times). The dry toluene 1.00 mL were added by syringe under Ar. The tube was then sealed and was stirred at 60 °C for 36 h the mixture was concentrated in vacuo. The crude product was further purified by column chromatography (petroleum ether/ethyl acetate = 60/1-30/1) on silica gel to yield the corresponding product 1-(*p*-tolyl)-1, 2, 3, 3a, 4, 5-hexahydropyrrolo[1, 2-a]quinolone **28** as a white solid (24.3 mg, 92%, d.r. = 12/1), mp: 151 − 153 °C. Major diastereomer：^{1}H NMR (400 MHz, Chloroform-*d*) δ 7.06 (d, J = 8.0 Hz, 2H), 7.00 (d, J = 7.9 Hz, 2H), 6.93 (d, J = 7.3 Hz, 1H), 6.74 (t, J = 7.5 Hz, 1H), 6.42 (t, J = 7.3 Hz, 1H), 5.98 (d, J = 8.1 Hz, 1H), 4.57 (d, J = 9.2 Hz, 1H), 3.56 - 3.36 (m, 1H), 2.97 - 2.87 (m, 1H), 2.81 - 2.77 (m, 1H), 2.37 - 2.25 (m, 1H), 2.22 (s, 3H), 2.19 - 2.15 (m, 1H), 1.87 – 1.81 (m, 1H), 1.77 - 1.72 (m, 2H), 1.65 - 1.52 (m, 1H). ^{13}C NMR (100 MHz, Chloroform-*d*) δ 142.9, 140.8, 134.9, 128.1, 127.7, 125.6, 124.9, 114.2, 111.0, 60.0, 58.3, 33.8, 28.7, 27.1, 26.9, 20.0. The enantiomeric excess was determined by chiral HPLC analysis (ChiralPak OD-H, 1% *i*-PrOH in hexanes, 0.5 mL/min, 254 nm). Minor enantiomer r_t= 11.232 min (30.9%), Major enantiomer r_t = 15.643 min (69.1%), er = 69:31.

Mechanistic Investigations

Control Experiment with Additives

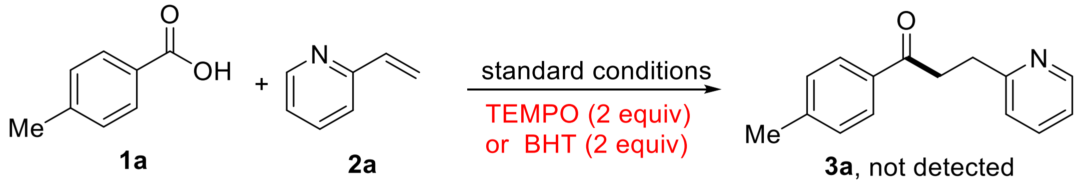

To a 10 mL Schlenk tube equipped with a magnetic stir bar was added carboxylic acid

1a (27.2 mg, 0.2 mmol, 1.0 equiv.), photocatalyst Ir[dF(CF_3)ppy]$_2$(dtbbpy)PF_6 (2.3 mg, 1 mol%), K_2HPO_4 (7.0 mg, 20 mol%), Ph_3P (62.9 mg, 0.24 mmol, 1.2 equiv.) and additives (TEMPO or BHT, 0.4 mmol, 2 equiv.) and the tube was evacuated and backfilled with Ar (three times). The 2-vinylpyridine **2a** (31.5 mg, 0.3 mmol, 1.5 equiv.) in DCM/H_2O (2 mL, 4:1 v/v) were added by syringe under Ar. The tube was then sealed and was placed at a distance (app. 5 cm) from 5 W blue LEDs lamp, and the mixture was stirred for 48 h at room temperature. The corresponding pyridine product **3a** was not detected according to both TLC and GC-Mass analysis. The product 2,2,6,6-tetramethylpiperidin-1-yl 4-methylbenzoate was detected by ESI-HRMS.

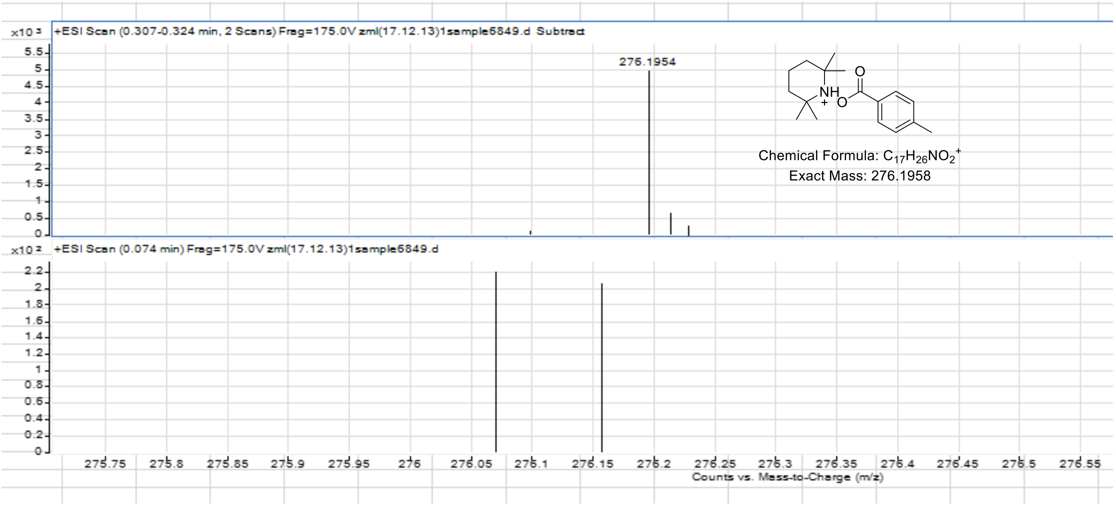

Supplementary Figure 8. ESI-HRMS Spectra after TEMPO added

Radical Cyclization Experiment

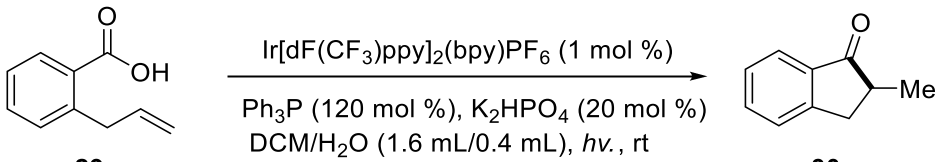

To a 10 mL Schlenk tube equipped with a magnetic stir bar was added 2-allylbenzoic acid **29** (32.4 mg, 0.2 mmol, 1.0 equiv.), photocatalyst Ir[dF(CF_3)ppy]$_2$(dtbbpy)PF_6 (2.3 mg, 1 mol%), K_2HPO_4 (7.0 mg, 20 mol%), and Ph_3P (62.9 mg, 0.24 mmol, 1.2 equiv.) and the tube was evacuated and backfilled with Ar (three times), DCM/H_2O (2 mL, 4:1 v/v) were added by syringe under Ar. The tube was then sealed and was placed at a distance (app. 5 cm) from 5 W blue LEDs lamp, and the mixture was stirred for 48 h at room temperature. After completion, the mixture was quenched with water and

extracted with DCM (3 x 10 mL). The combined organic layer was dried over anhydrous Na_2SO_4, then the solvent was removed under vacuo. The residue was purified with chromatography column on silica gel (gradient eluent of petroleum ether/EtOAc: 40/1 to 20/1) to give the corresponding product **30** 2-methyl-2,3-dihydro-1H-inden-1-one as colorless liquid (30%, 8.8 mg). ^{1}H NMR (400 MHz, Chloroform-*d*) δ 7.76 (d, J = 7.7 Hz, 1H), 7.59 (t, J = 7.4 Hz, 1H), 7.45 (d, J = 8.5 Hz, 1H), 7.37 (t, J = 7.8 Hz, 1H), 3.44 – 3.37 (m, 1H), 2.80 – 2.66 (m, 2H), 1.32 (d, J = 7.3 Hz, 3H). ^{13}C NMR (100 MHz, Chloroform-*d*) δ 209.5, 153.5, 136.4, 134.7, 127.4, 126.5, 124.0, 42.0, 35.0, 16.3. These results indicated that a free radical process was involved.

Deuterium-Labeling Experiments

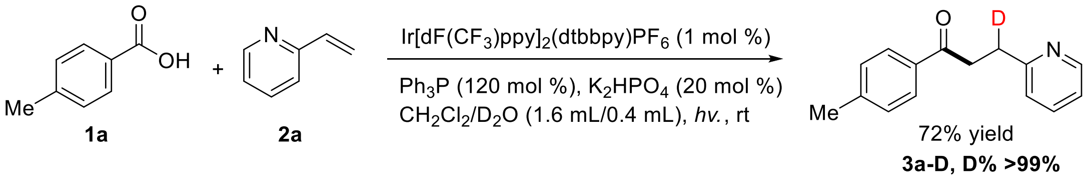

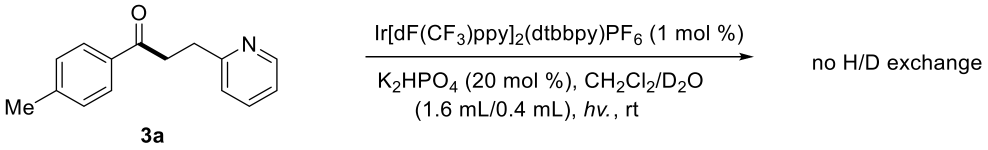

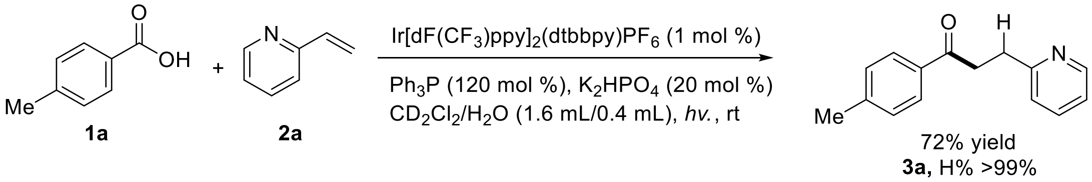

To a 10 mL Schlenk tube equipped with a magnetic stir bar was added carboxylic acid **1a** (27.2 mg, 0.2 mmol, 1.0 equiv.), the photocatalyst Ir[dF(CF_3)ppy]$_2$(dtbbpy)PF_6 (2.3 mg, 1 mol%), K_2HPO_4 (7.0 mg, 20 mol%), and Ph_3P (62.9 mg, 0.24 mmol, 1.2 equiv.) and the tube was evacuated and backfilled with Ar (three times), After that CH_2Cl_2 (1.6 mL), D_2O (0.4 mL) and 2-vinylpyridine **2a** (31.5 mg, 0.3 mmol, 1.5 equiv.) were added sequentially by means of syringe under argon. The tube was then sealed and was placed at a distance (app. 5 cm) from 5 W blue LEDs lamp, and the mixture was stirred for 48 h at room temperature. It gave the deuterated product **3a-D**. Furthermore, when product **3a** was subjected to the same reaction conditions or the reaction was conducted in CD_2Cl_2 (1.6 mL) and H_2O (0.4 mL), no H/D exchange occurred.

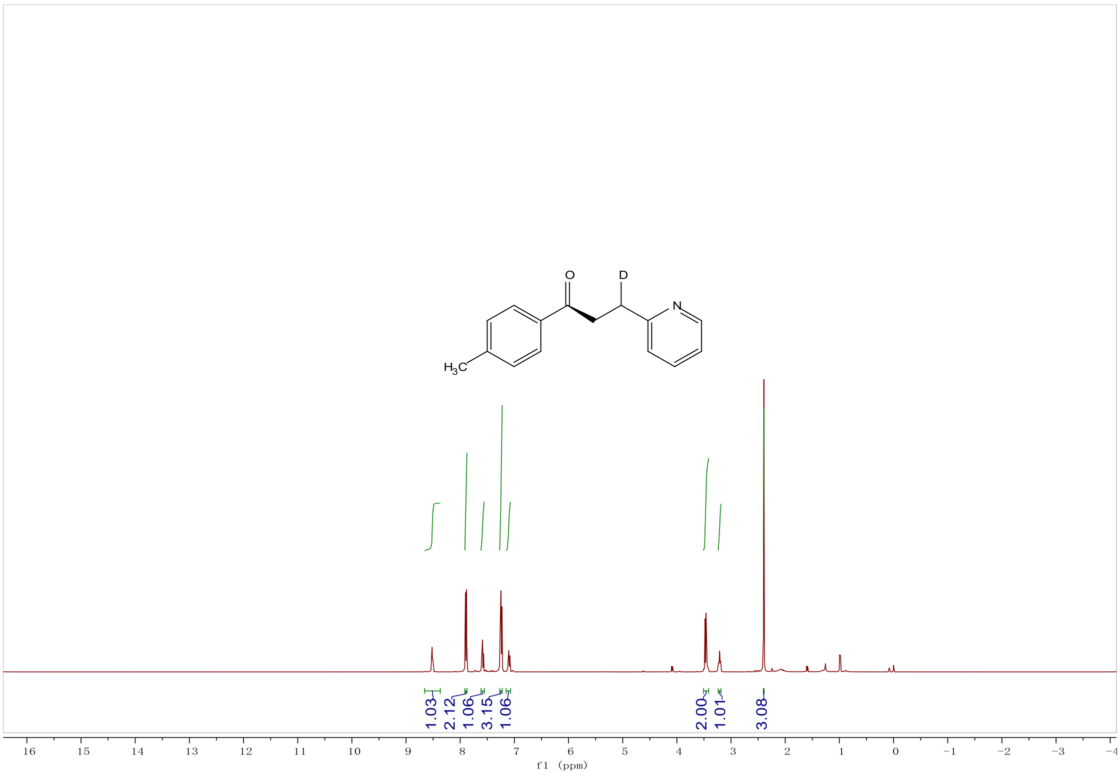

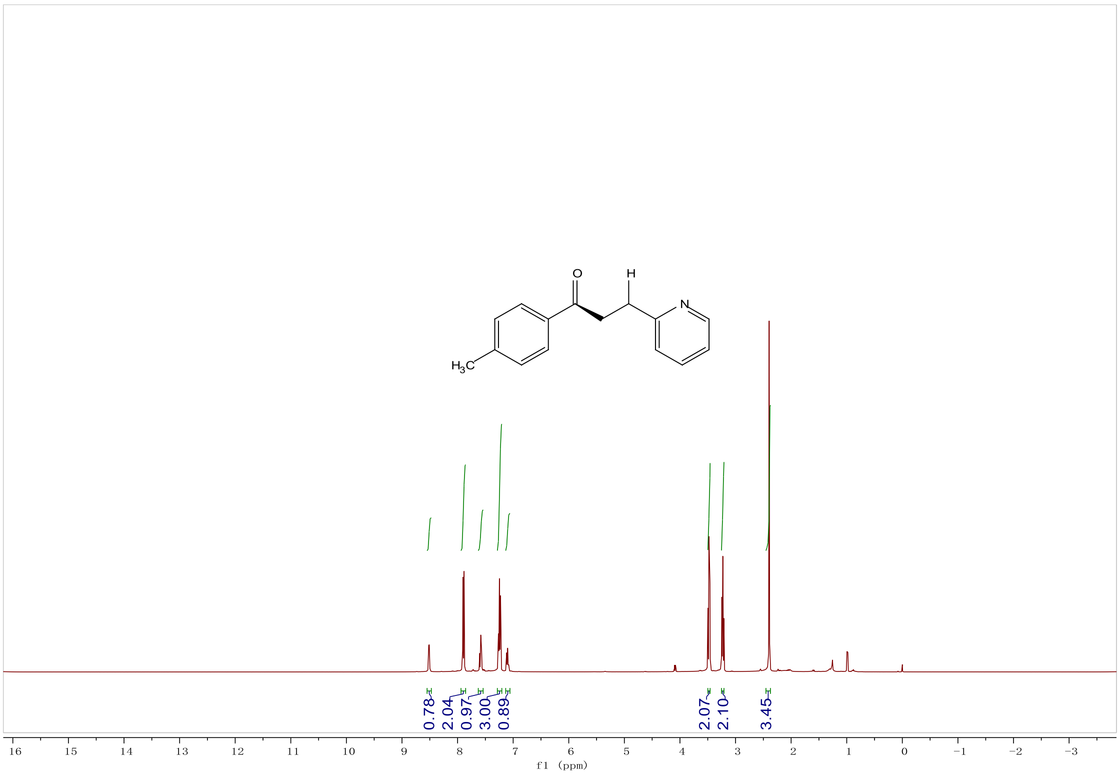

Supplementary Figure 9. ^{1}H NMR spectra for deuterium-labeling experiments

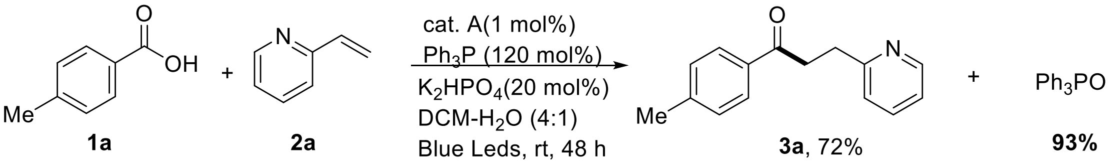

In addition, under standard conditions the reaction was carried out on 0.2 mmol scale (48 h). The residue was purified by flash column chromatography (petroleum ether: ethyl acetate, 4/1-1/1) to afford triphenylphosphine oxide (93%, 62.2 mg) as white solid. ^{1}H NMR (400 MHz, Chloroform-d) δ 7.70 – 7.65 (m, 6H), 7.59 – 7.50 (m, 3H), 7.49 – 7.41 (m, 6H). ^{31}P NMR (162 MHz, Chloroform-*d*) δ 29.06.

A little amount of 1,2-diketones were detected by GC−MS as the side products are produced.

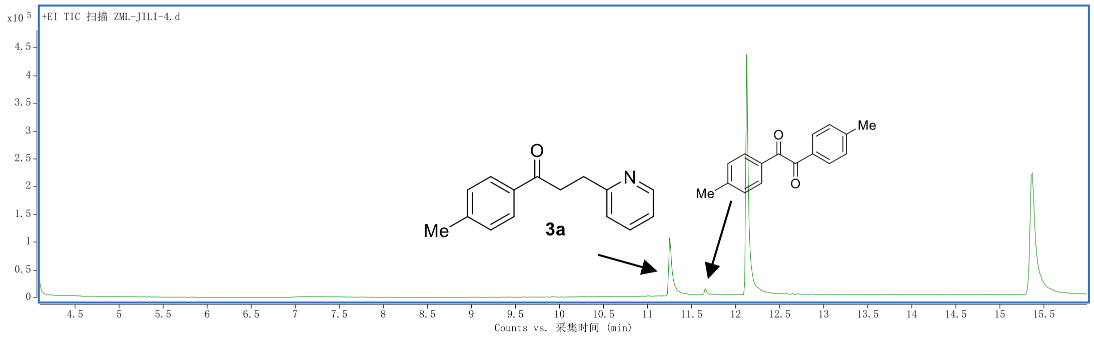

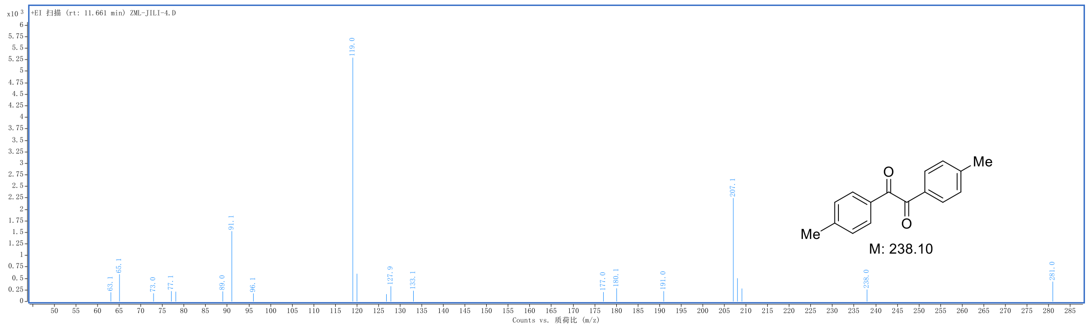

Supplementary Figure 10. Mass spectra for GC-MS traces

^{18}O-Labeling Experiments

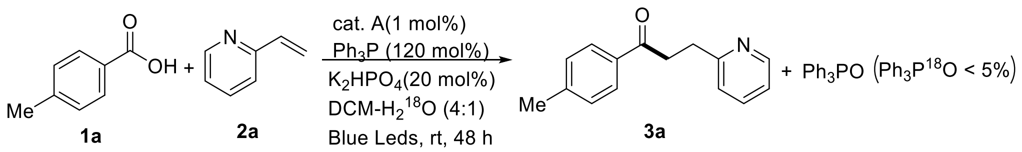

To a 10 mL Schlenk tube equipped with a magnetic stir bar was added carboxylic acid **1a** (27.2 mg, 0.2 mmol, 1.0 equiv.), the photocatalyst Ir[dF(CF$_3$)ppy]$_2$(dtbbpy)PF$_6$ (2.3 mg, 1 mol%), K_2HPO_4 (7.0 mg, 20 mol%), and Ph_3P (62.9 mg, 0.24 mmol, 1.2 equiv.) and the tube was evacuated and backfilled with Ar (three times), After that CH_2Cl_2 (1.6 mL), $H_2{}^{18}O$ (0.4 mL) and 2-vinylpyridine **2a** (31.5 mg, 0.3 mmol, 1.5 equiv.) were added sequentially by means of syringe under argon. The tube was then sealed and was placed at a distance (app. 5 cm) from 5 W blue LEDs lamp, and the mixture was stirred

for 48 h at room temperature.

The MS data of producing triphenylphosphine oxide

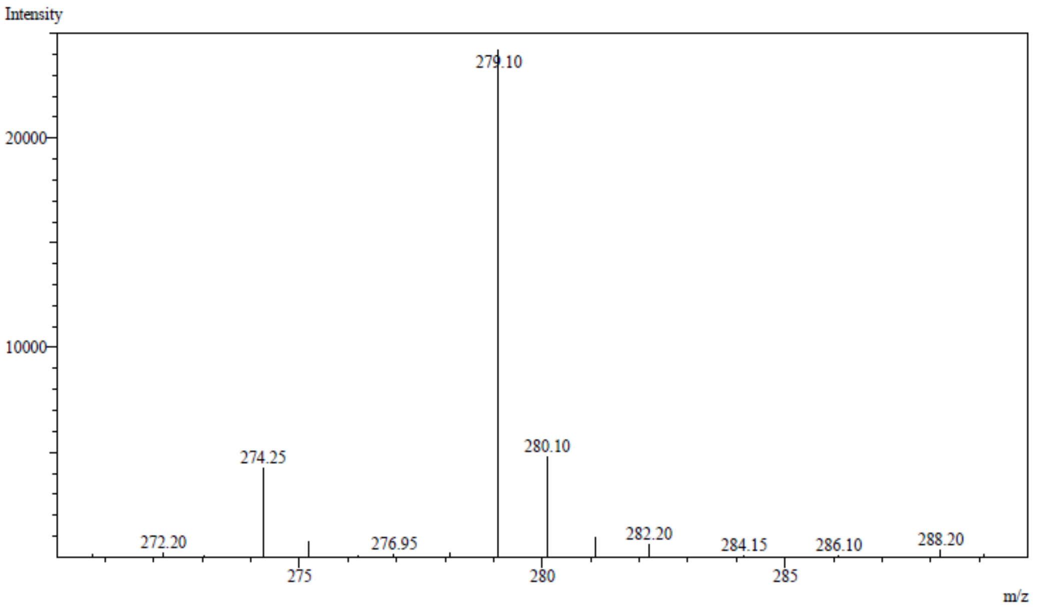

Supplementary Figure 11. Mass spectrum for triphenylphosphine oxide

Preparation of ^{18}O-labeled 4-methylbenzoic acid (1a'): To a solution of 4-methylbenzoyl chloride (385 mg, 2.50 mmol) in THF (8 mL) was added $H_2{}^{18}O$ (100.4 mg, 5.0 mmol, 98% ^{18}O incorporation) and the mixture was stirred at room temperature for 6 h. After removal of the solvent, the residue was purified by silica gel chromatography (PE/EA, 5:1) to give **1a'** (289 mg, 85% yield, 93% ^{18}O incorporation) as a white solid.

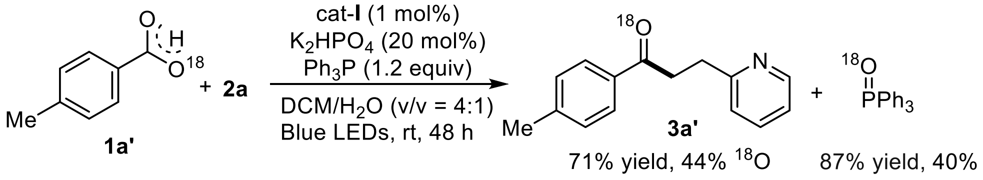

(Eq 11)

To a 10 mL Schlenk tube equipped with a magnetic stir bar was added carboxylic acid **1a'** (27.6 mg, 0.2 mmol, 1.0 equiv.), the photocatalyst Ir[dF(CF_3)ppy]$_2$(dtbbpy)PF_6 (2.3 mg, 1 mol%), K_2HPO_4 (7.0 mg, 20 mol%), and Ph_3P (62.9 mg, 0.24 mmol, 1.2 equiv.) and the tube was evacuated and backfilled with Ar (three times), After that CH_2Cl_2 (1.6 mL), H_2O (0.4 mL) and 2-vinylpyridine **2a** (31.5 mg, 0.3 mmol, 1.5 equiv.) were added sequentially by means of syringe under argon. The tube was then sealed and was placed at a distance (app. 5 cm) from 5 W blue LEDs lamp, and the mixture was stirred for 48

h at room temperature.

The HRMS data of producing triphenylphosphine oxide and **3a'**

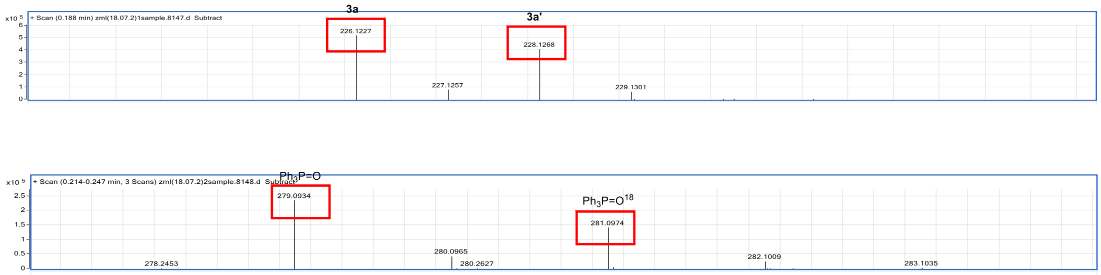

Supplementary Figure 12. Mass spectra for triphenylphosphine oxide and **3a'**

Aromatic Carboxylate Anion as Substrate

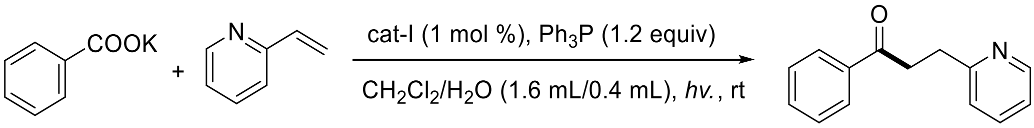

2a **3d**, 42% yield

To a 10 mL Schlenk tube equipped with a magnetic stir bar was added potassium benzoate (32.0 mg, 0.2 mmol, 1.0 equiv.), the photocatalyst Ir[dF(CF_3)ppy]$_2$(dtbbpy)PF_6 (2.3 mg, 1 mol%) and Ph_3P (62.9 mg, 0.24 mmol, 1.2 equiv.) and the tube was evacuated and backfilled with Ar (three times), After that CH_2Cl_2 (1.6 mL), H_2O (0.4 mL) and 2-vinylpyridine **2a** (31.5 mg, 0.3 mmol, 1.5 equiv.) were added sequentially by means of syringe under argon. The tube was then sealed and was placed at a distance (app. 5 cm) from 5 W blue LEDs lamp, and the mixture was stirred for 48 h at room temperature. The residue was purified by flash column chromatography (petroleum ether: ethyl acetate, 10/1-4/1) to afford **3d** (42%, 17.7 mg). We suggested reactions of triphenylphosphine radical cation with aromatic carboxylate anion under the present reaction conditions generated the corresponding acyl radicals.

Luminescence Quenching Experiment

The luminescence quenching experiment was taken using a Cary Eclipse fluorescence spectrophotometer (Varian, USA). The experiments were carried out in 2.5 x 10^{-5} mol/L of [Ir{dF(CF_3)ppy}$_2${dtbbpy}]PF_6 in DCM at 25 °C. The emission intensity was collected at 475 nm. The concentrations of quenchers (**1a** and Ph_3P) in DCM were 0, 2, 4, 6, 10, 14, 24 mM.

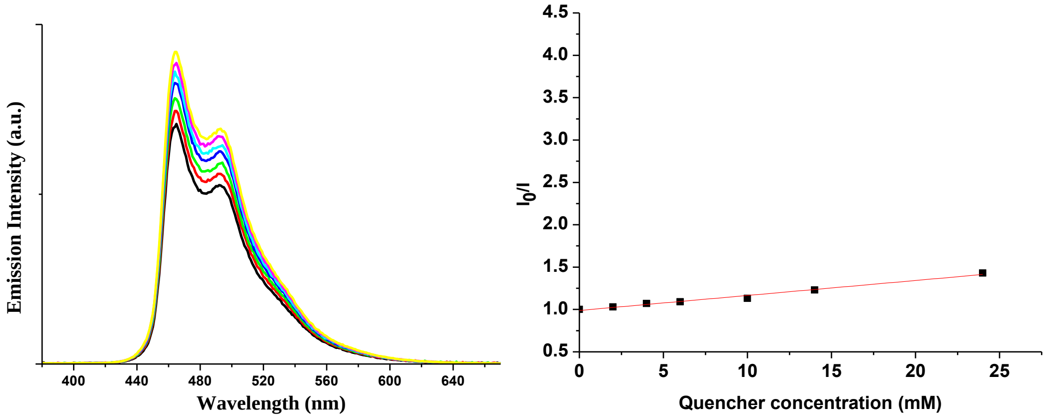

Supplementary Figure 13. Luminescence quenching of [Ir{dF(CF_3)ppy}$_2${dtbbpy}]PF_6 by acid **1a**

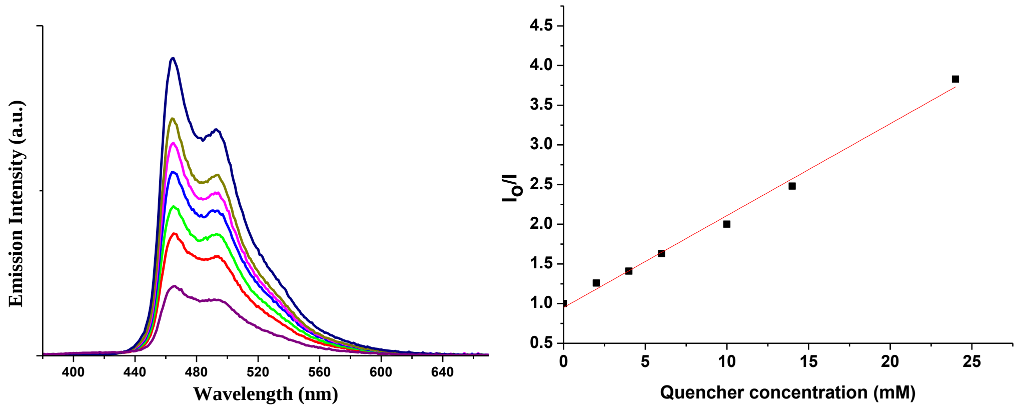

Supplementary Figure 14. Luminescence quenching of [Ir{dF(CF_3)ppy}$_2${dtbbpy}]PF_6 by Ph_3P

General procedure for three-component reductive coupling reaction

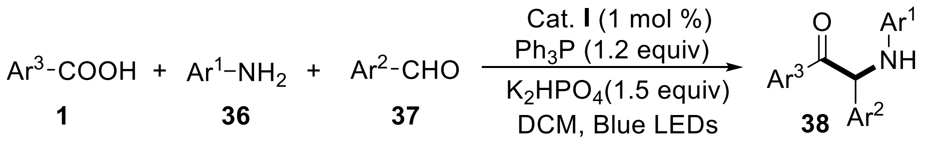

To a 10 mL Schlenk tube equipped with a magnetic stir bar was added aromatic carboxylic acid **1** (0.15 mmol, 1.5 equiv.), photocatalyst A Ir[dF(CF_3)ppy]$_2$(dtbbpy)PF_6 (1.2 mg, 1 mol%), K_2HPO_4 (26.1 mg, 1.5 equiv.), and Ph_3P (31.5 mg, 0.12 mmol, 1.2 equiv.) and the tube was evacuated and backfilled with Ar (three times). The amines **36** (0.1 mmol, 1.0 equiv.), aldehydes **37** (0.15 mmol, 1.5 equiv.) in DCM (2.0 mL) were added by syringe under Ar. The tube was then sealed and was placed at a distance (app. 5 cm) from 5 W blue LEDs lamp, and the mixture was stirred for 48 h at room temperature. After completion, the solvent was removed under vacuo. The residue was

purified with chromatography column on silica gel (gradient eluent of petroleum ether/EtOAc: 100/1-50/1) to give the corresponding amino ketone products **38**.

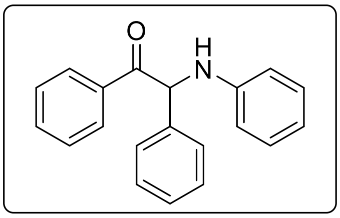

1,2-diphenyl-2-(phenylamino)ethan-1-one **38a**

The reaction was carried out according to the general procedure on 0.1 mmol scale (48 h). The residue was purified by flash column chromatography (petroleum ether: ethyl acetate, 100/1-50/1) to afford **38a** (13.8 mg, 48%) as a yellow solid, mp: 123 − 125 °C.^{1}H NMR (400 MHz, Chloroform-*d*) δ 7.99 (d, *J* = 8.5 Hz, 2H), 7.53 (t, *J* = 7.4 Hz, 1H), 7.47 – 7.38 (m, 4H), 7.32 – 7.24 (m, 2H), 7.20 (t, *J* = 7.3 Hz, 1H), 7.15 – 7.09 (m, 2H), 6.68 (t, *J* = 8.1 Hz, 3H), 6.02 (s, 1H), 5.41 (br s, 1H). ^{13}C NMR (100 MHz, Chloroform-*d*) δ 197.1, 146.1, 137.7, 135.1, 133.6, 129.3, 129.1, 128.9, 128.7, 128.1 (2C), 117.9, 113.5, 62.7. HRMS (ESI) Calcd for $C_{20}H_{18}NO$ $[M+H]^+$: 288.1383, found: 288.1384.

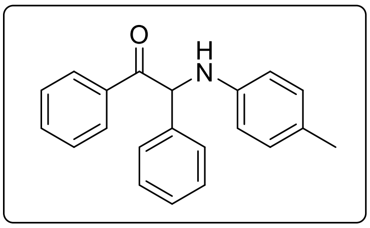

1,2-diphenyl-2-(*p*-tolylamino)ethan-1-one **38b**

The reaction was carried out according to the general procedure on 0.1 mmol scale (48 h). The residue was purified by flash column chromatography (petroleum ether: ethyl acetate, 100/1-50/1) to afford **38b** (15.4 mg, 51%) as a yellow solid, mp: 184 − 186 °C. ^{1}H NMR (400 MHz, Chloroform-*d*) δ 7.99 (d, *J* = 8.5 Hz, 2H), 7.52 (t, *J* = 7.4 Hz, 1H), 7.42 (t, *J* = 7.7 Hz, 2H), 7.32 (d, *J* = 8.1 Hz, 2H), 7.17 – 7.03 (m, 4H), 6.67 (t, *J* = 7.7 Hz, 3H), 5.99 (s, 1H), 5.36 (br s, 1H), 2.24 (s, 3H). ^{13}C NMR (100 MHz, Chloroform-*d*) δ 197.1, 146.2, 137.9, 135.1, 134.6, 133.5, 129.8, 129.2, 128.9, 128.7, 128.0, 117.8, 113.5, 62.4, 21.1. HRMS (ESI) Calcd for $C_{21}H_{20}NO$ $[M+H]^+$: 302.1539, found: 302.1540.

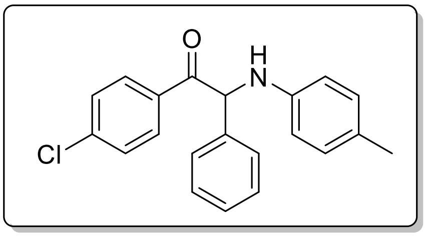

1-(4-chlorophenyl)-2-phenyl-2-(*p*-tolylamino)ethan-1-one **38c**

The reaction was carried out according to the general procedure on 0.1 mmol scale (48 h). The residue was purified by flash column chromatography (petroleum ether: ethyl acetate, 100/1-50/1) to afford **38c** (14.4 mg, 43%) as a yellow solid, mp: 145 − 147 °C. ^{1}H NMR (400 MHz, Chloroform-*d*) δ 7.93 (d, *J* = 8.6 Hz, 2H), 7.39 (d, *J* = 8.5 Hz, 2H), 7.30 (d, *J* = 8.0 Hz, 2H), 7.19 – 7.01 (m, 4H), 6.73 - 6.67 (m, 3H), 5.93 (s, 1H), 2.26 (s, 3H). ^{13}C NMR (100 MHz, Chloroform-*d*) δ 195.8, 145.7, 140.0, 138.2, 134.1, 133.3, 130.3, 129.9, 129.3, 129.0, 128.1, 118.3, 113.8, 62.8, 21.1. HRMS (ESI) Calcd for $C_{21}H_{19}ClNO$ [M+H]$^+$: 336.1150, found: 336.1151.

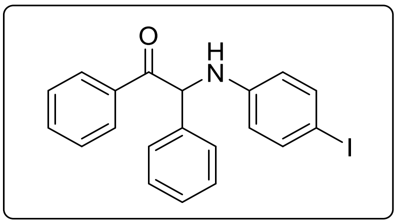

2-((4-iodophenyl)amino)-1,2-diphenylethan-1-one **38d**

The reaction was carried out according to the general procedure on 0.1 mmol scale (48 h). The residue was purified by flash column chromatography (petroleum ether: ethyl acetate, 100/1-50/1) to afford **38d** (19.0 mg, 41%) as a yellow solid, mp: 202 − 204 °C. ^{1}H NMR (400 MHz, Chloroform-*d*) δ 7.98 (d, *J* = 8.6 Hz, 2H), 7.54 (t, *J* = 7.4 Hz, 1H), 7.47 – 7.34 (m, 6H), 7.30 – 7.25 (m, 2H), 7.22 (d, *J* = 7.3 Hz, 2H), 6.45 (d, *J* = 8.8 Hz, 2H), 5.97 (s, 1H), 5.50 (br s, 1H). ^{13}C NMR (100 MHz, Chloroform-*d*) δ 196.5, 145.6, 137.8, 137.1, 134.8, 133.7, 129.2, 128.9, 128.8, 128.3, 128.1, 78.6, 62.4. HRMS (ESI) Calcd for $C_{20}H_{17}INO$ [M+H]$^+$: 414.0349, found: 414.0350.

Starting Materials

Numbers of Substrates

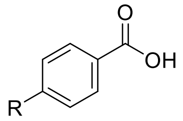

1a, R = Me
1b, R = Ph
1c, R = OBn
1d, R = H
1e, R = F
1f, R = Cl
1g, R = Br
1h, R = I

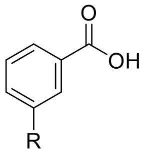

1i, R = Me
1j, R = NHBoc
1k, R = CHO
1l, R = COOMe

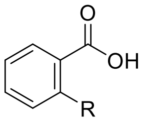

1m, R = Me
1n, R = OAc
1o, R = OTs

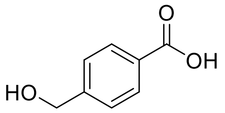

1p

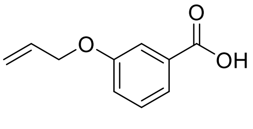

1q

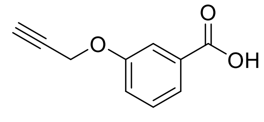

1r

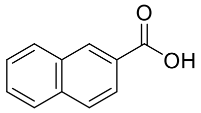

1s

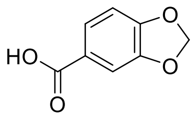

1t

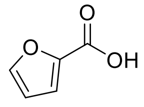

1u

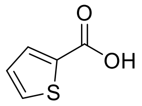

1v

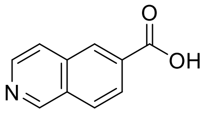

1w

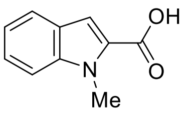

1x

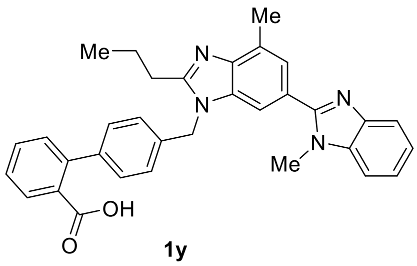

1y

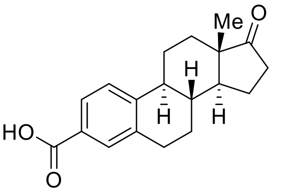

1z

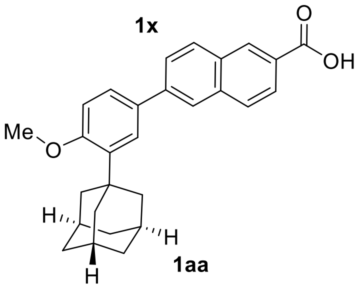

1aa

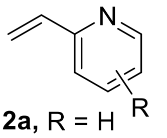

2a, R = H
2b, R = 5-Me
2c, R = 5-Cl
2d, R = 5-CHO
2e, R = 5-COMe
2f, R = 5-COOEt
2g, R = 3-CF_3
2h, R = 4-Br
2i, R = 6-OMe
2j, R = 6-Br

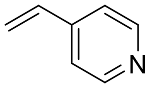

2k

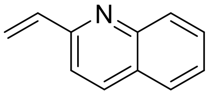

2l

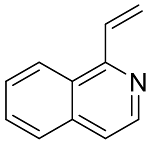

2m

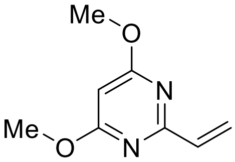

2n

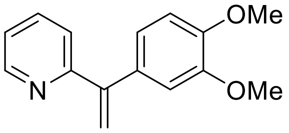

2o

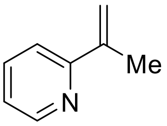

2p

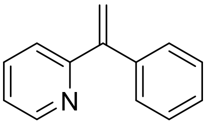

2q

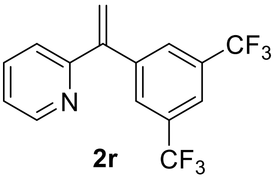

2r

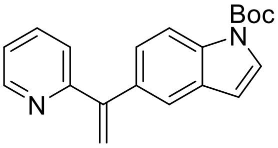

2s

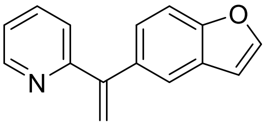

2t

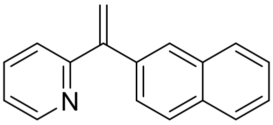

2u

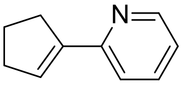

2v

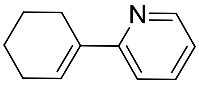

2w

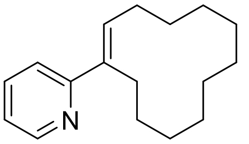

2x

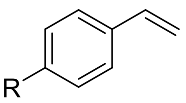

2y, R = CF_3,
2z, R = COOMe
2aa, R = CN

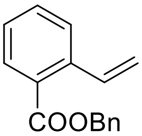

2bb

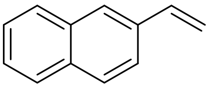

2cc

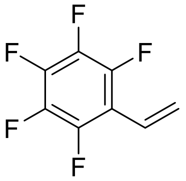

2dd

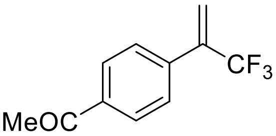

2ee

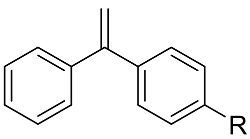

2ff, R = H
2gg, R = Ph
2hh, R = SMe
2ii, R = COMe
2jj, R = COOMe

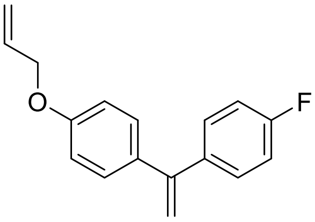

2kk

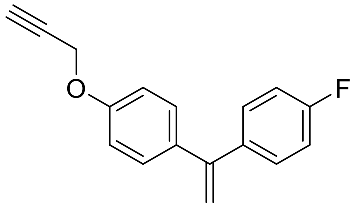

2ll

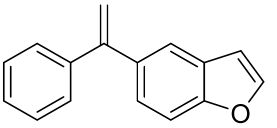

2mm

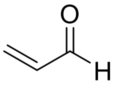

2nn

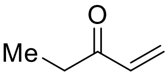

2oo

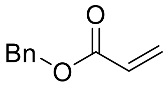

2pp

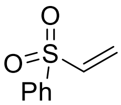

2qq

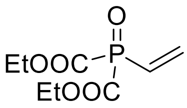

2rr

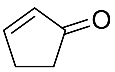

2ss

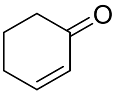

2tt

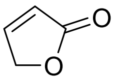

2uu

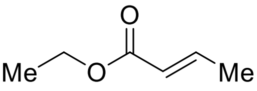

2vv

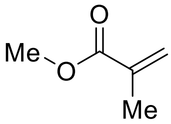

2ww

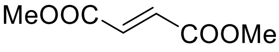

2xx

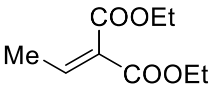

2yy

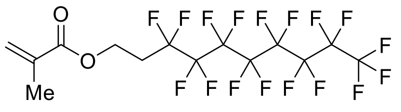
HDFDMA

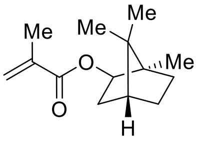
Acryester IBX

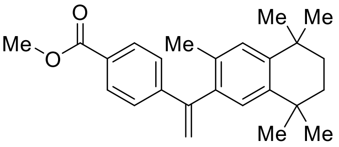
Bexarotene

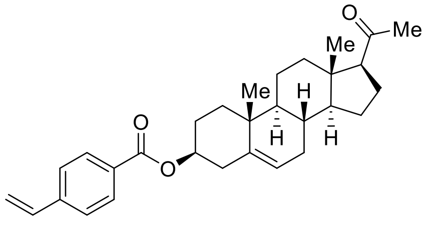
Pregnenolone

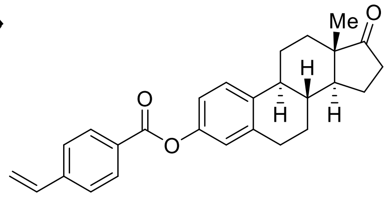
Esterone

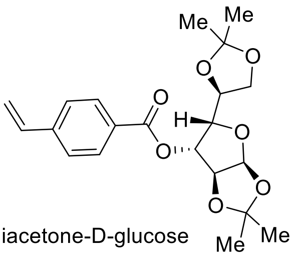
Diacetone-D-glucose

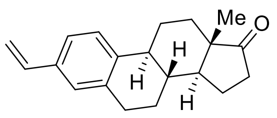
Esterone

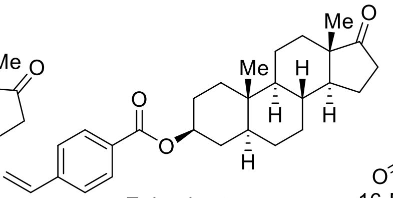
Epiandrosterone

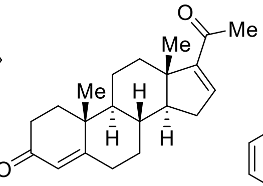
16-Dehydroprogesterone

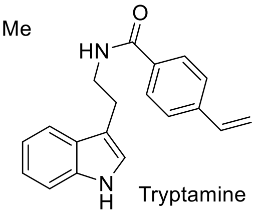
Tryptamine

Synthesis of Carboxylic Acid

Synthesis of carboxylic acids: 1q, **1r**.

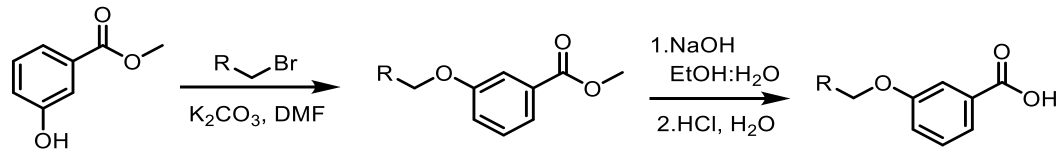

Following the modified procedure of reported literature,[5] a 100 mL oven-dried Schlenk-tube was charged with potassium carbonate (2.49 g, 18.0 mmol), methyl 3-hydroxybenzoate (6.0 mmol, 1.0 equiv.) and the flask was purged and filled with argon. DMF (30 mL, 0.2 M) was added by syringe and the mixture was stirred. The corresponding bromide (7.5 mmol, 1.25 equiv.) was added by syringe and the reaction mixture was stirred under argon at room temperature for 24 h. The mixture was diluted with EtOAc and water, shaken, and separated. The aqueous layer was extracted one time with EtOAc. The combined organic layers were washed with water, washed once with brine, dried over $MgSO_4$, filtered, and concentrated under vacuum to provide the crude ester product.

The corresponding ester (1.0 mmol), 3.0 M aqueous NaOH (3.5 mL), and ethanol (5.9 mL) were combined in a flask with a stir bar and stirred at 60 °C for 36 h. The reaction

mixture was cooled to room temperature. Aqueous HCl (1.0 M) was slowly added to the stirring mixture (pH = 1.0 - 2.0). The aqueous layer was extracted with EtOAc (4 x 20 mL) and the combined organic extracts were washed with brine (100 mL), dried over Na_2SO_4 and concentrated under reduced pressure and the crude products were purified by flash column chromatography (hexanes : EtOAc 2 : 1).

Synthesis of carboxylic acid 1o.

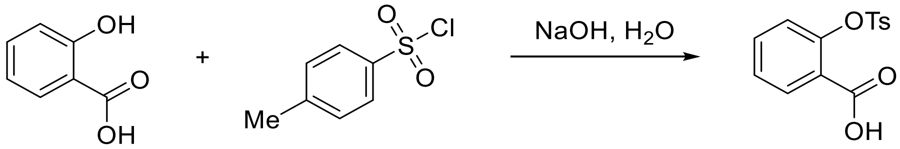

Following the modified procedure of reported literature,[6] 2-Hydroxy-benzoic acid (20.0 g, 0.145 mol) was dissolved in a solution of NaOH (11.6 g, 0.29 mol) in 100 mL of water. The *p*-toluenesulfonyl chloride (27.6 g, 0.145 mol) was then added in small portions. When addition was complete, stirring at room temperature was continued overnight. The solid formed was filtered and re-dissolved in a NaOH aqueous solution and the resulting solution carefully acidified with diluted HCl. The precipitate was filtered and washed with boiling water. On cooling, unreacted 2-hydroxy-benzoic acid was recovered from the filtrate. The remaining solid were purified by flash column chromatography to give the acid product **1o**.

Synthesis of estrone acid 1z

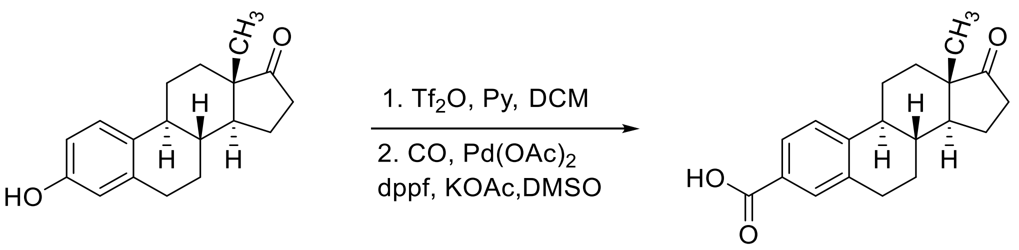

Following the modified procedure of reported literature,[7] Step 1. Pyridine (10.0 mmol, 2.0 equiv) was added to a stirring solution of estrone (5.0 mmol, 1.0 equiv) in DCM (25 mL) under Ar. Then, triflic anhydride (6.0 mmol, 1.2 equiv) was added dropwise to the mixture in an ice bath. The mixture was warmed to room temperature and stirred for 5 h. Then, the reaction was quenched by the addition of water. The layers were separated, and the aqueous phase was extracted with DCM (30 mL × 3). The combined

organic phase was washed with brine, dried over sodium sulfate, filtered, and evaporated under reduced pressure to afford the corresponding crude trifluoromethane-sulfonate substituted compound. The crude compound was used without further purification.

Step 2: $Pd(OAc)_2$ (0.25 mmol, 5 mol %), 1,1′-bis(diphenylphosphino) ferrocene (1 mmol, 20 mol %), and potassium acetate (20 mmol, 4 equiv) were added to the crude compound in DMSO (50 mL). The reaction mixture was stirred at 60 °C under a balloon of CO overnight. The mixture was then cooled to room temperature, quenched with 1 M HCl (pH < 3), and extracted with EtOAc (50 mL× 3). The combined organic phase was washed with brine, dried over sodium sulfate, filtered, and evaporated under reduced pressure to afford the corresponding crude carboxylic acid. The crude carboxylic acid was purified by column chromatography on silica gel to obtain the corresponding acid.

Synthesis of 10-(methacryloyloxy)decylbenzoic acids: 20a, 20b, 20c

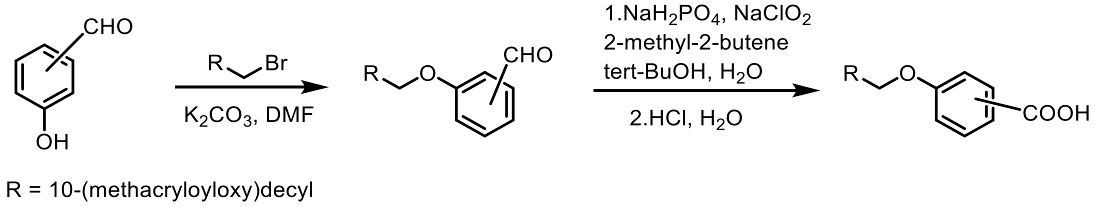

Following the modified procedure of reported literature,[5] a 100 mL oven-dried Schlenk-tube was charged with potassium carbonate (2.49 g, 18.0 mmol), methyl 3-hydroxybenzaldehyde (6.0 mmol, 1.0 equiv.) and the flask was purged and filled with argon. DMF (30 mL, 0.2 M) was added by syringe and the mixture was stirred. The corresponding bromide (7.5 mmol, 1.25 equiv.) was added by syringe and the reaction mixture was stirred under argon at room temperature for 24 h. The mixture was diluted with EtOAc and water, shaken, and separated. The combined organic extracts washed with brine (200 mL), dried (Na_2SO_4), filtered and concentrated *in vacuo*. Purification by flash chromatography (petroleum ether/EtOAc, 40:1), afforded the corresponding 10-(methacryloyloxy)decylbenzaldehyde products.

Following the general procedure of reported literature,[8] To a solution of 10-(methacryloyloxy)decylbenzaldehyde (0.83 g, 2.40 mmol), NaH_2PO_4 (288 mg, 2.40

mmol), 2-methyl-2-butene (1.12 mL, 10.6 mmol) in *tert*-BuOH (15 mL) and water (4 mL) was added $NaClO_2$ (739 mg, 8.17 mmol) and the mixture was stirred for 50 min at room temperature. The reaction mixture was adjusted to pH of 4 by addition of 1 M HCl. The aqueous layer was extracted with CH_2Cl_2. The organic layers were combined, washed with brine, dried over anhydrous Na_2SO_4. Purification by flash chromatography (petroleum ether/EtOAc, 10:1 - 5:1), afforded the corresponding 10-(methacryloyloxy)decylbenzoic acids **20a, 20b, 20c**.

Synthesis of 2-allyl benzoic acid 29

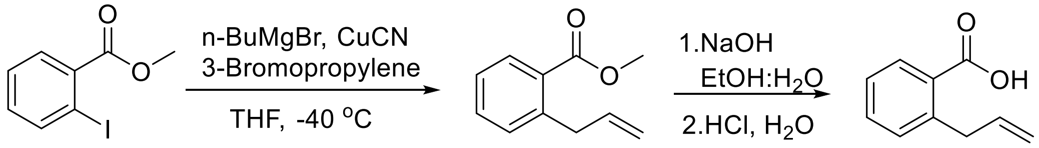

Following the modified procedure of reported literature,[9] to a suspension of magnesium turnings (2.0 g, 67.0 mmol) and a crystal of iodine in THF (30 mL) was added dropwise bromobutane (7.2 mL, 67.0 mmol). The mixture was stirred for 15 minutes then cooled to 40 °C before dropwise addition of methyl-2-iodobenzoate (5.0 mL, 34.0 mmol). The mixture was stirred at 40 °C for 1.5 h. A freshly prepared solution of LiCl (3.4 g, 80.0 mmol) and CuCN (3.4 g, 40.0 mmol) in THF (60 mL) was added and the mixture was stirred for a further 15 min, followed by the addition of allyl bromide (12.0 mL, 140 mmol). The mixture was stirrer at 40 °C for a further 10 min, then warmed to room temperature. The mixture was diluted with EtOAc (200 mL) and filtered over Celite®. The filtrate was washed with 25 % aq. NH_4OH (200 mL). The aqueous layer was further extracted with EtOAc (2 x 200 mL), and the combined organic extracts washed with brine (200 mL), dried (Na_2SO_4), filtered and concentrated *in vacuo*. Purification by flash chromatography (petroleum ether/EtOAc, 9:1), afforded methyl-2-allyl-benzoate as a colourless oil.

Methyl-2-allyl benzoate (2.7 g, 17.0 mmol) was dissolved in EtOH (250 mL), and 2.0 M aq. NaOH (200 mL) added. The mixture was stirred for at room temperature for 4 h, then EtOH was removed *in vacuo.* The residue was extracted with Et_2O (2 x 150 mL), acidified to pH 3 with 2.0 M aq. HCl and extracted with EtOAc (3 x 150 mL). The combined organics were dried (Na_2SO_4), filtered and concentrated in vacuo and

purified by flash chromatography (petroleum ether/EtOAc, 5:1) to afford 2-allyl benzoic acid **29**.

Synthesis of alkenes

Synthesis of Alkenylpyridines 2b, 2c, 2h, 2j

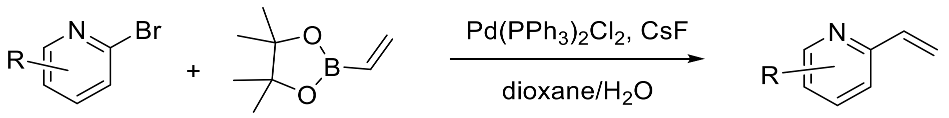

Following the modified procedure of reported literature,[10] to a solution of bromide-substituted azaarene (10.0 mmol, 1.0 equiv.), CsF (4.6 g, 30.0 mmol) and pinacol vinylboronate (2.3 g, 15.0 mmol) in dioxane/H_2O (20 mL/10 mL) was added $Pd(PPh_3)_2Cl_2$ (0.7 g, 1.0 mmol). After stirring for 16 h at 80 °C under Ar, the mixture was concentrated in vacuo. The crude product was further purified by column chromatography (petroleum ether/ethyl acetate = 40/1 - 10/1) on silica gel to yield the corresponding alkenylpyridines **2b**, **2c**, **2h**, **2j**.

Synthesis of Alkenylpyridines 2d, 2e, 2f, 2g, 2i, 2l, 2m, 2n.

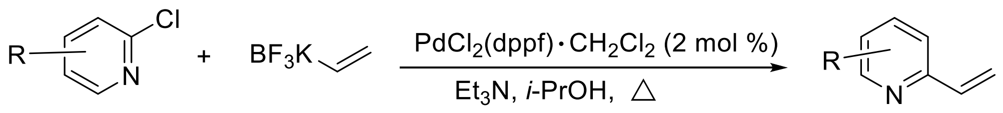

Following the modified procedure of reported literature,[11] a solution of chlor-substituted azaarene (8.0 mmol, 1.0 equiv.), potassium vinyltrifluoroborate (1.29 g, 9.6 mmol), $PdCl_2(dppf){\cdot}CH_2Cl_2$ (131 mg, 0.16 mmol), and Et_3N (1.12 mL, 8.0 mmol) in *i*-PrOH (125 mL) was heated to reflux for 16 h. The mixture was cooled to room temperature and partitioned between CH_2Cl_2 (100 mL) and H_2O (40 mL). The aqueous layer was separated and extracted with CH_2Cl_2 (2 x 50 mL) and the combined organic layers were washed with brine (100 mL), dried ($MgSO_4$), filtered, and concentrated in vacuo. Purification of the residue by column chromatography (petroleum ether/ethyl acetate = 40/1 - 10/1) gave the vinylpyridines **2d**, **2e**, **2f**, **2g**, **2i**, **2l**, **2m**, **2n**.

Synthesis of Alkenylpyridine 2o, 2q, 2r, 2u.

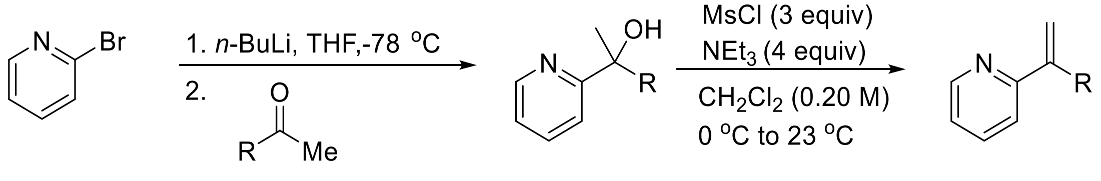

Following the modified procedure of reported literature,[12] a solution of 2-bromopyridine (2.37 g, 1.43 mL, 15.0 mmol, 1.0 equiv.) in THF (19 mL, 0.8 M) was cooled to -78 °C. *n*-Butyllithium (15.8 mmol, 1.1 equiv.) was then slowly added and the reaction mixture was stirred at -78 °C for 10 min. After that time, the corresponding ketone (15.0 mmol, 1.0 equiv.) was added to the above solution and the reaction mixture was stirred for 15 h while slowly warming up to 23 °C. NH_4Cl (aq) (20 mL) and EtOAc (20 mL) were added, the layers were separated and the aqueous layer was extracted with EtOAc (3 × 20 mL). The organic extracts were combined, dried ($MgSO_4$), filtered and concentrated in vacuo. The residue was purified by chromatography on silica gel, eluting with hexanes/ EtOAc (9:1 to 4:2 (v/v)), to afford the title compound pyridine alcohol.

A solution of pyridine alcohol (5.0 mmol, 1.0 equiv.) in CH_2Cl_2 (25 mL, 0.2 M) was cooled to 0 °C. Triethylamine (2.02 g, 2.8 mL, 20.0 mmol, 4.0 equiv.) and methanesulfonyl chloride (1.73 g, 15.0 mmol, 3.0 equiv.) were then added and the reaction mixture was stirred for 15 h while warming up to 23 °C. Saturated solution of $NaHCO_3$ (aq.) (40 mL) was added, the layers were separated and the aqueous layer was extracted with CH_2Cl_2 (3 × 20 mL). The organic extracts were combined, dried ($MgSO_4$), filtered and concentrated in vacuo. The residue was purified by chromatography on silica gel, eluting with hexanes/EtOAc (40:1 to 20:1 (v/v)), to afford the title compounds **2o**, **2q**, **2r**, **2u**.

Synthesis of Alkenylpyridine 2s

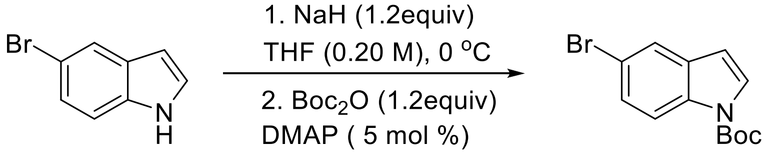

Following the modified procedure of reported literature,[12] a solution of 5-bromoindole (2.0 g, 10.2 mmol, 1.0 equiv.) in THF (51 mL, 0.2 M) was cooled to 0 °C. NaH (60% dispersion in mineral oil) (490 mg, 12.2 mmol, 1.2 equiv.) was then added and the reaction mixture was stirred at 0 °C for 1 h. After that time, Boc anhydride (2.67 g, 12.2 mmol, 1.2 equiv.) and 4-dimethylaminopyridine (62.3 mg, 0.51 mmol, 5 mol%) were

added and the reaction mixture was stirred for 12 h while warming up to 23 °C. NH_4Cl (aq.) (50 mL) and EtOAc (50 mL) were added, the layers were separated and the aqueous layer was extracted with EtOAc (3 × 40 mL). The organic extracts were combined, dried ($MgSO_4$), filtered and concentrated in vacuo. The residue was purified by chromatography on silica gel, eluting with hexanes/EtOAc (9:1 (v/v)), to afford the title compound as a white solid.

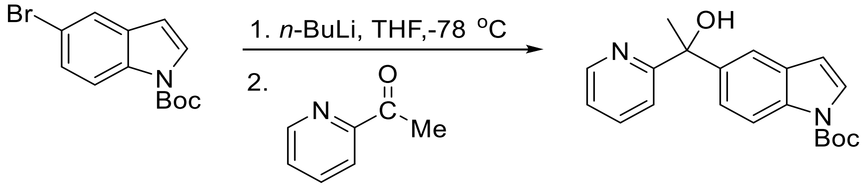

A solution of tert-butyl 5-bromo-1H-indole-1-carboxylate (2.0 g, 6.7 mmol, 1.0 equiv.) in THF (23 mL, 0.3 M) was cooled to -78 °C. *n*-Butyllithium (7.1 mmol, 1.1 equiv.) was then slowly added and the reaction mixture was stirred at -78 °C for 10 min. After that time, 2-acetylpyridine (0.82 g, 6.7 mmol, 1.0 equiv.) was added to the above solution and the reaction mixture was stirred for 15 h while slowly warming up to 23 °C. NH_4Cl (aq.) (20 mL) and EtOAc (20 mL) were added, the layers were separated and the aqueous layer was extracted with EtOAc (3 × 20 mL). The organic extracts were combined, dried ($MgSO_4$), filtered and concentrated in vacuo. The residue was purified by chromatography on silica gel, eluting with hexanes/EtOAc (7:3 (v/v)), to afford the title compound as a pink solid.

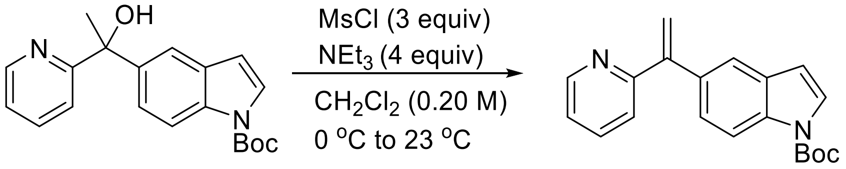

A solution of tert-butyl 5-(1-hydroxy-1-(pyridin-2-yl)ethyl)-1H-indole-1-carboxylate (0.54 g, 1.58 mmol, 1.0 equiv.) in CH_2Cl_2 (7.9 mL, 0.2 M) was cooled to 0 °C. Triethylamine (0.64 g, 6.3 mmol, 4.0 equiv.) and methanesulfonyl chloride (0.54 g, 4.7 mmol, 3.0 equiv.) were then added and the reaction mixture was stirred for 15 h while warming up to 23 °C. Saturated solution of $NaHCO_3$ (aq.) (20 mL) was added, the layers were separated and the aqueous layer was extracted with CH_2Cl_2 (3 × 20 mL). The organic extracts were combined, dried ($MgSO_4$), filtered and concentrated in vacuo.

The residue was purified by chromatography on silica gel, eluting with hexanes/EtOAc (17:3 to 4:1 (v/v)), to afford the title compound **2s**.

Synthesis of Alkenylpyridine 2t

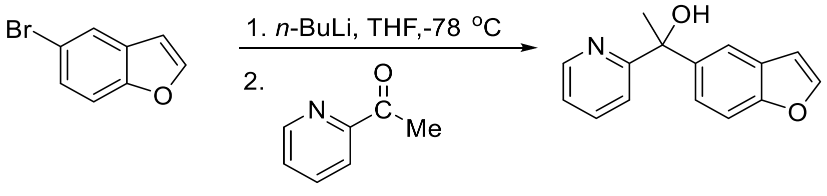

Following the modified procedure of reported literature,[12] a solution of 5-bromobenzofuran (2.01 g, 10.2 mmol, 1.0 equiv.) in THF (34 mL, 0.3 M) was cooled to -78 °C. *n*-Butyllithium (10.2 mmol, 1.05 equiv.) was then slowly added and the reaction mixture was stirred at -78 °C for 10 min. After that time, 2-acetylpyridine (1.24 g, 10.2 mmol, 1.0 equiv.) was added to the above solution and the reaction mixture was stirred for 15 h while slowly warming up to 23 °C. NH_4Cl (aq.) (40 mL) and EtOAc (30 mL) were added, the layers were separated and the aqueous layer was extracted with EtOAc (3 × 30 mL). The organic extracts were combined, dried ($MgSO_4$), filtered and concentrated in vacuo. The residue was purified by chromatography on silica gel, eluting with hexanes/EtOAc (15:3 to 7:3 (v/v)), to afford the title compound as a yellow oil.

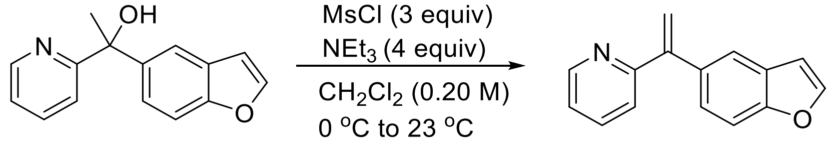

A solution of 1-(benzofuran-5-yl)-1-(pyridin-2-yl)ethan-1-ol (0.769 g, 3.2 mmol, 1.0 equiv.) in CH_2Cl_2 (16 mL, 0.2 M) was cooled to 0 °C. Triethylamine (1.3 g, 1.8 mL, 12.8 mmol, 4.00 equiv.) and methanesulfonyl chloride (1.1 g, 0.75 mL, 9.6 mmol, 3.0 equiv.) were then added and the reaction mixture was stirred for 15 h while warming up to 23 °C. Saturated solution of $NaHCO_3$ (aq.) (30 mL) was added, the layers were separated and the aqueous layer was extracted with CH_2Cl_2 (3 × 20 mL). The organic extracts were combined, dried ($MgSO_4$), filtered and concentrated in vacuo. The residue was purified by chromatography on silica gel, eluting with hexanes/EtOAc (4:1 to 7:3 (v/v)), to afford the title compound **2t**.

Synthesis of Alkenes: 2p, 2gg, 2hh, 2ii, 2jj, 2kk, 2ll, 2mm

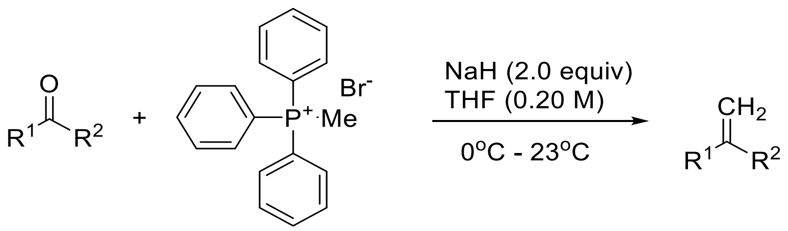

Following the modified procedure of reported literature,[12] a suspension of methyltriphenylphosphonium bromide (4.61 g, 12.9 mmol, 1.2 equiv.) in THF (54 mL, 0.2 M) was cooled to 0 °C. NaH (60% dispersion in mineral oil) (0.86 g, 21.6 mmol, 2.0 equiv.) was added and the reaction mixture was stirred at 0 °C for 25 min. ketone (10.8 mmol, 1 equiv.) was then added and the reaction mixture was stirred for 15 h while slowly warming up to 23 °C. NH_4Cl (aq.) (50 mL) and EtOAc (30 mL) were added, the layers were separated and the aqueous layer was extracted with EtOAc (3 × 30 mL). The organic extracts were combined, dried ($MgSO_4$), filtered and concentrated in vacuo. The residue was purified by chromatography on silica gel, eluting with hexanes/EtOAc (19:1 to 9:1 (v/v)), to afford the alkenes **2p, 2gg, 2hh, 2ii, 2jj, 2kk, 2ll, 2mm**.

Synthesis of Alkenylpyridine 2v, **2w**, **2x**

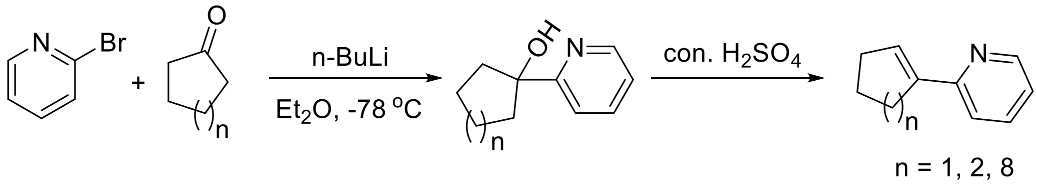

Following the modified procedure of reported literature,[13] to a solution of 2-bromopyridine (10.0 mmol, 1.58 g) in anhydrous Et_2O (20 mL) was added *n*-BuLi (11.0 mmol, 2.5 M in hexane, 4.4 mL) at -78 °C. The mixture was stirred for 1 h and the corresponding ketone was added by a disposable syringe. The reaction was allowed to warm to room temperature and stirred for further 12 h. After completion, the mixture was quenched with HCl (10 mL, 1.0 M) and extracted with ethyl acetate (20 mL×3). The organic layer was dried over anhydrous sodium sulfate and concentrated by rotary evaporation. The residue was purified by silica gel column chromatography to afford the alcohol derivative. Concentrated H_2SO_4 (5 mL) was added to the above alcohol and stirred at room temperature for 1 h. Crashed ice was added and neutralized with NaOH

solution to PH = 7 - 8. The reaction mixture was extracted with ethyl ether (20 mL×3) then washed with brine and dried over anhydrous sodium sulfate. The solvent was removed by rotary evaporation and purified by silica gel column chromatography to afford the pure products **2v**, **2w**, **2x**.

Synthesis of alkene 2ee

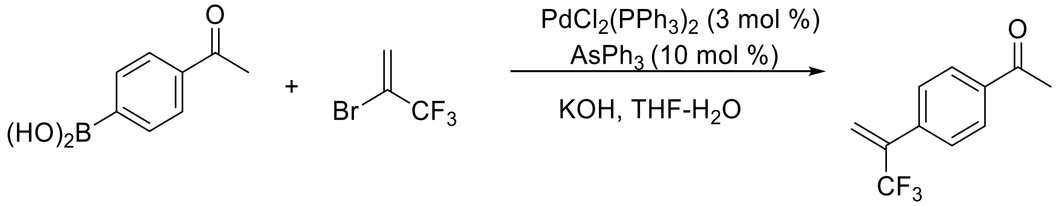

Following the modified procedure of reported literature,[14] to a THF (15.0 mL) solution were added 4-ethanoylphenylboronic acid (796 mg, 4.85 mmol), 2-bromo-3,3,3-trifluoropropene (1.32 g, 7.55 mmol), $PdCl_2(PPh_3)_2$ (105 mg, 0.15 mmol), $AsPh_3$ (230 mg, 0.751 mmol), aqueous KOH (2.0 M, 10 mL, 20 mmol), under reflux conditions for 18 h. NH_4Cl (aq.) (40 mL) and EtOAc (20 mL) were added, the layers were separated and the aqueous layer was extracted with EtOAc (3 × 30 mL). The organic extracts were combined, dried ($MgSO_4$), filtered and concentrated in vacuo. Purification by silica gel column chromatography (hexane/EtOAc = 20:1~10:1).

Synthesis of alkenes: 2bb, Esterone, Diacetone-D-glucose, Epiandrosterone, Tryptamine

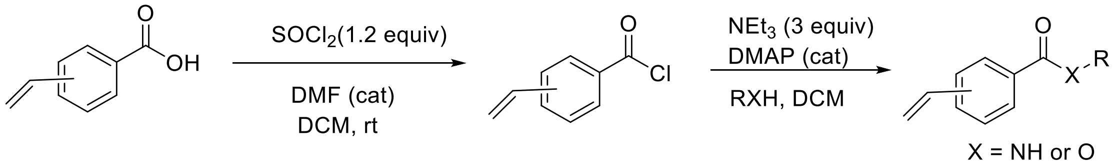

Following the modified procedure of reported literature,[15] to a dried 50 mL two necked round bottom flask were added vinylbenzoic acid (0.74 g, 5.0 mmol), DCM (20 mL), and DMF (25 μL) sequentially. Then, thionyl chloride (0.44 mL, 6.0 mmol) was added dropwise. The resulting mixture was stirred at room temperature for 2 h and concentrated under reduced pressure. The residue was dissolved in DCM (20 mL) again. Then, to a solution of RXH (4.0 mol) and DMAP (10 mg) in DCM (20 mL) previously prepared in another 50 mL round bottom flask was added this acyl chloride solution via

cannula. Then, NEt_3 (2.1 mL, 15 mmol) was added dropwise at 0 °C. The reaction mixture was allowed to be stirred at room temperature. After 2 h, the solution was diluted with EtOAc (10 mL) and quenched with 1N HCl aq. (10 mL). After separation, the aqueous layer was extracted with EtOAc (2 × 10 mL). The combined organic layers were washed with brine (10 mL), dried over $MgSO_4$, and evaporated under reduced pressure. The resulting crude product was purified by silica-gel column chromatography to afford alkenes **2bb, Esterone, Diacetone-D-glucose, Epiandrosterone, Tryptamine**.

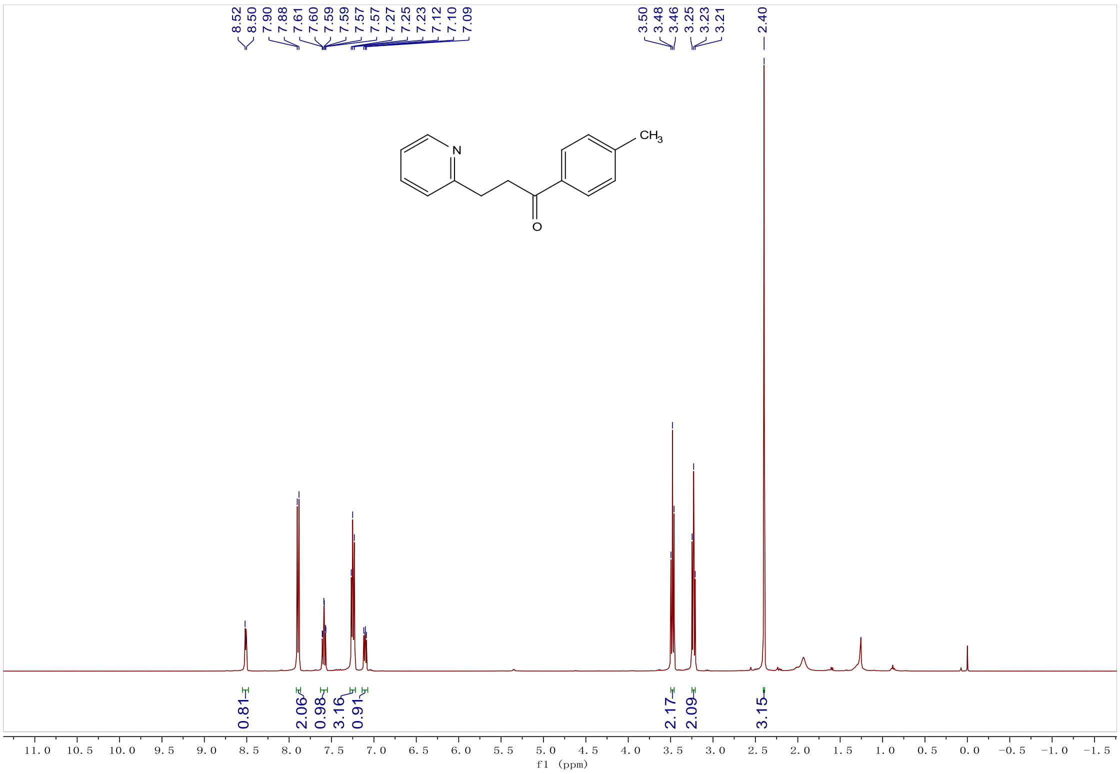

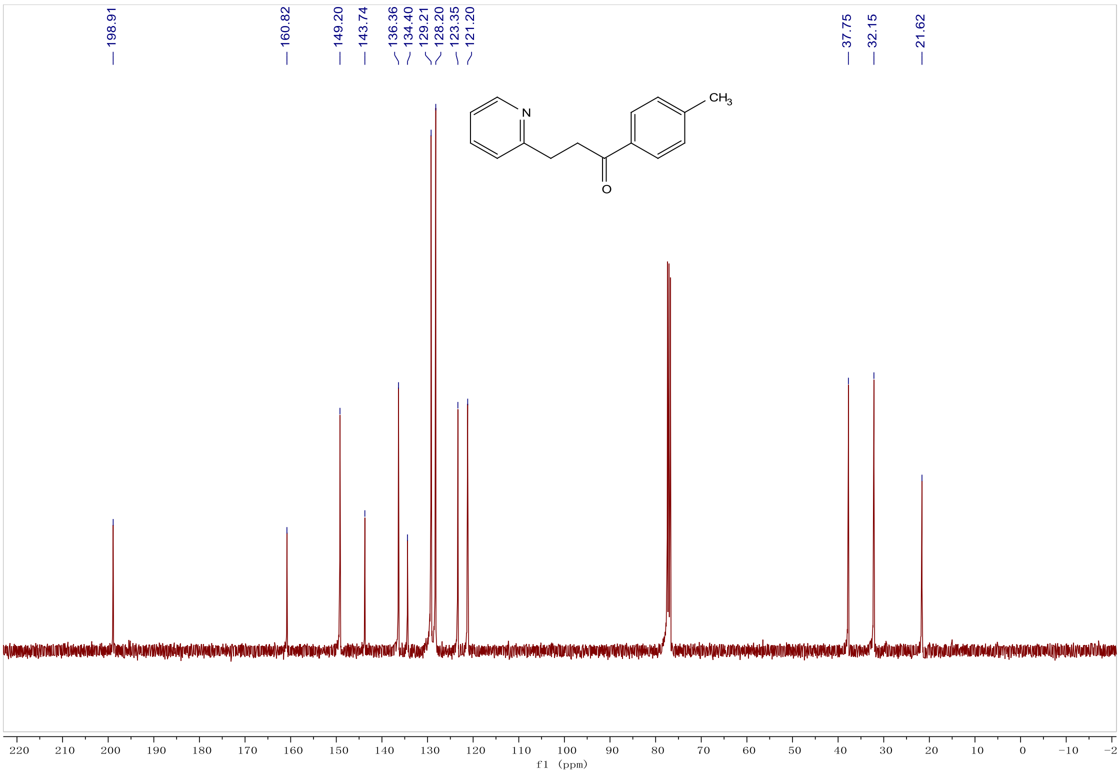

Supplementary Figure 15. ^{1}H and ^{13} C NMR spectra for compound **3a**

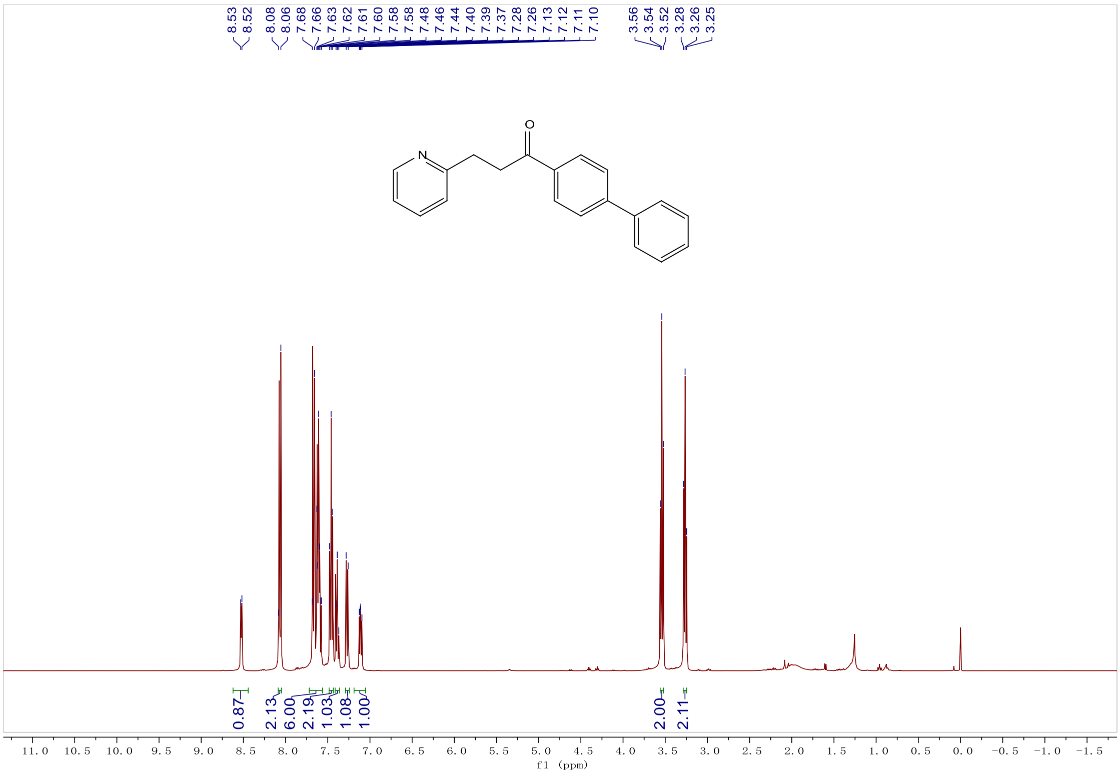

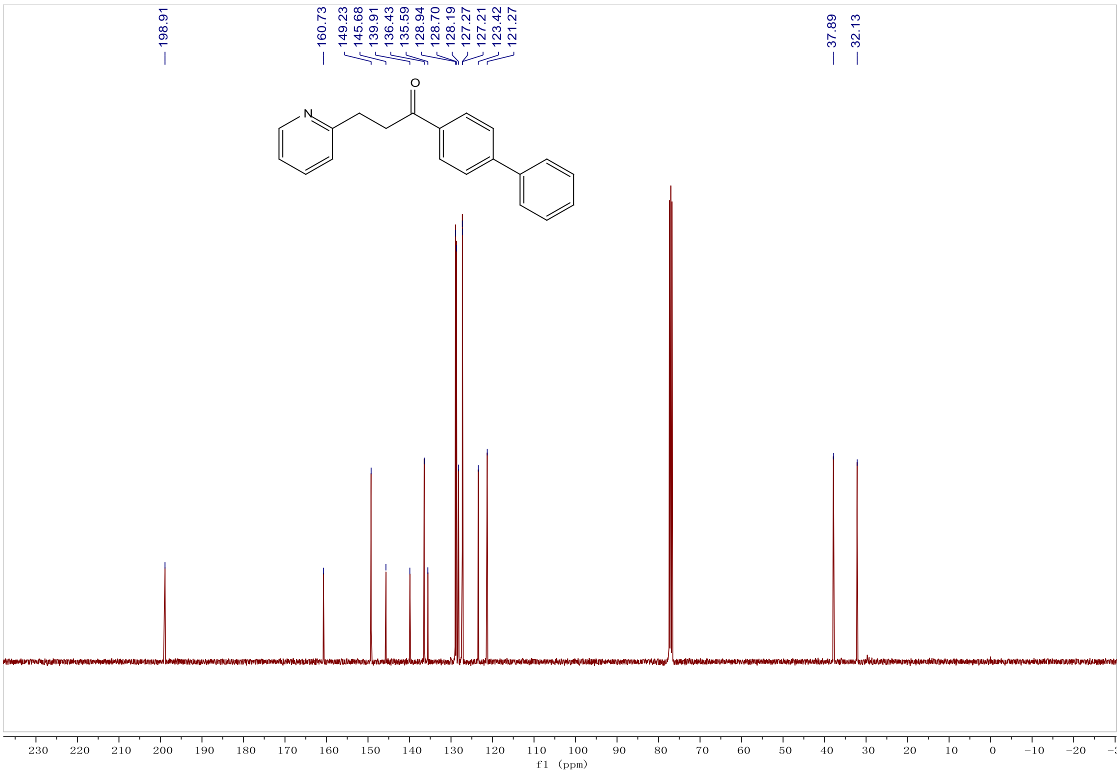

Supplementary Figure 16. ^{1}H and ^{13} C NMR spectra for compound **3b**

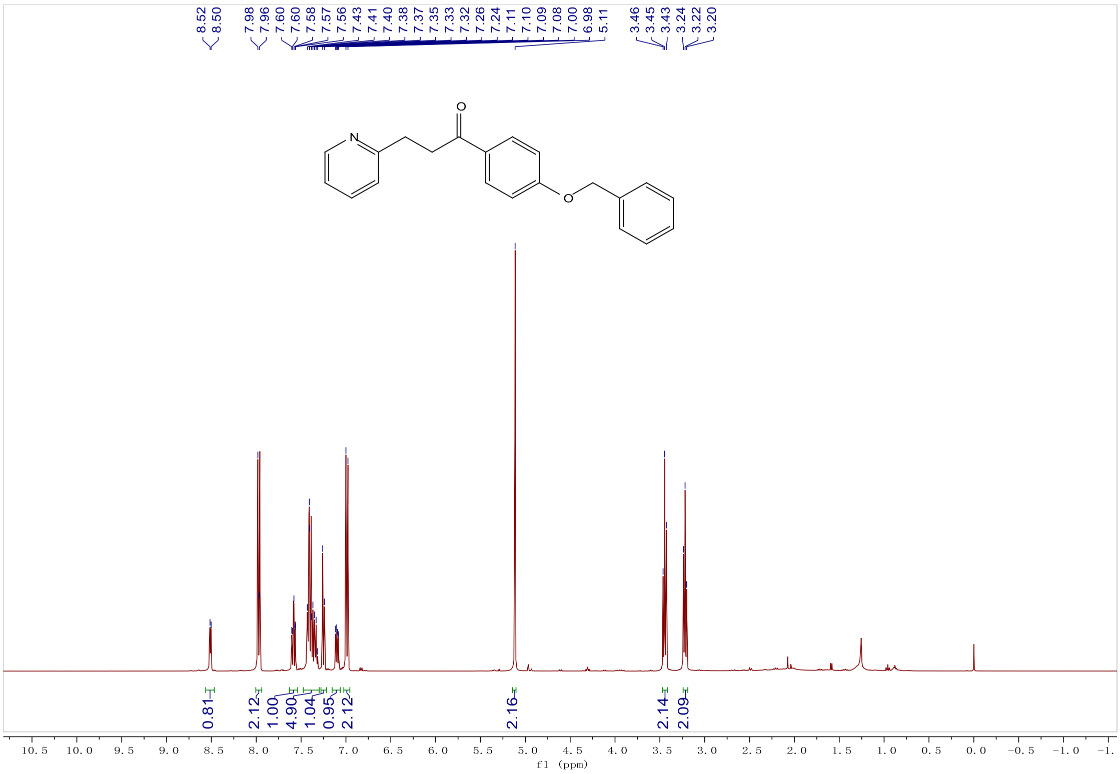

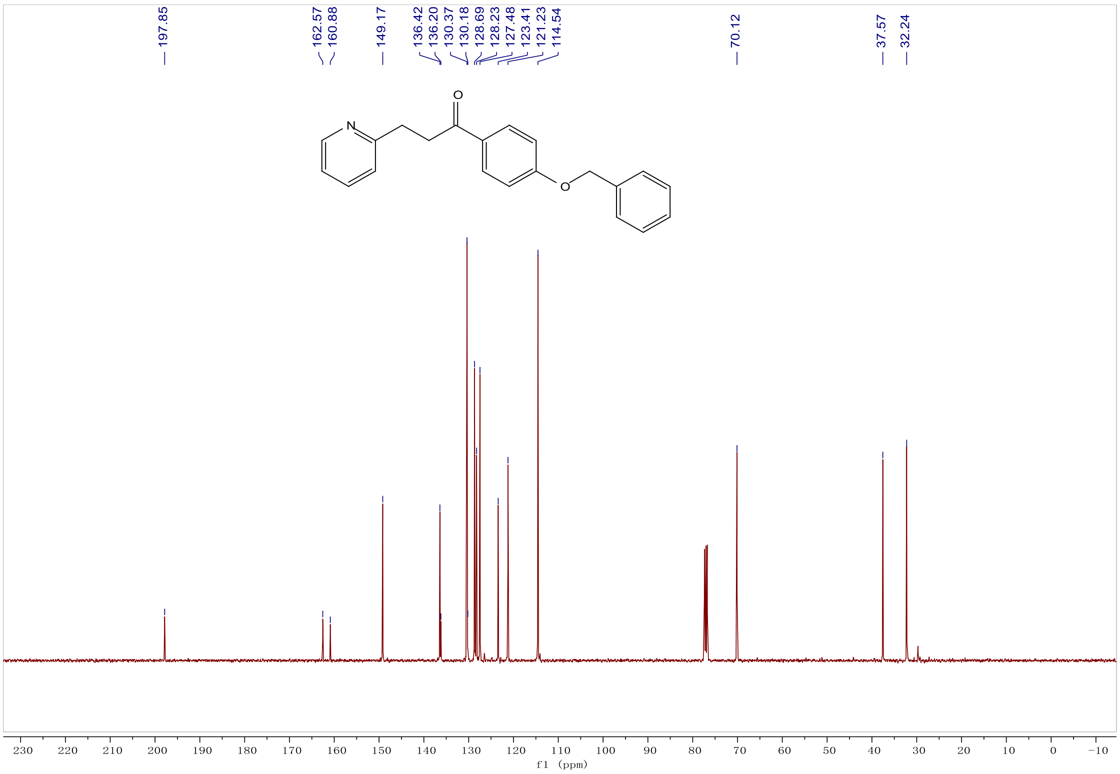

Supplementary Figure 17. ^{1}H and ^{13} C NMR spectra for compound **3c**

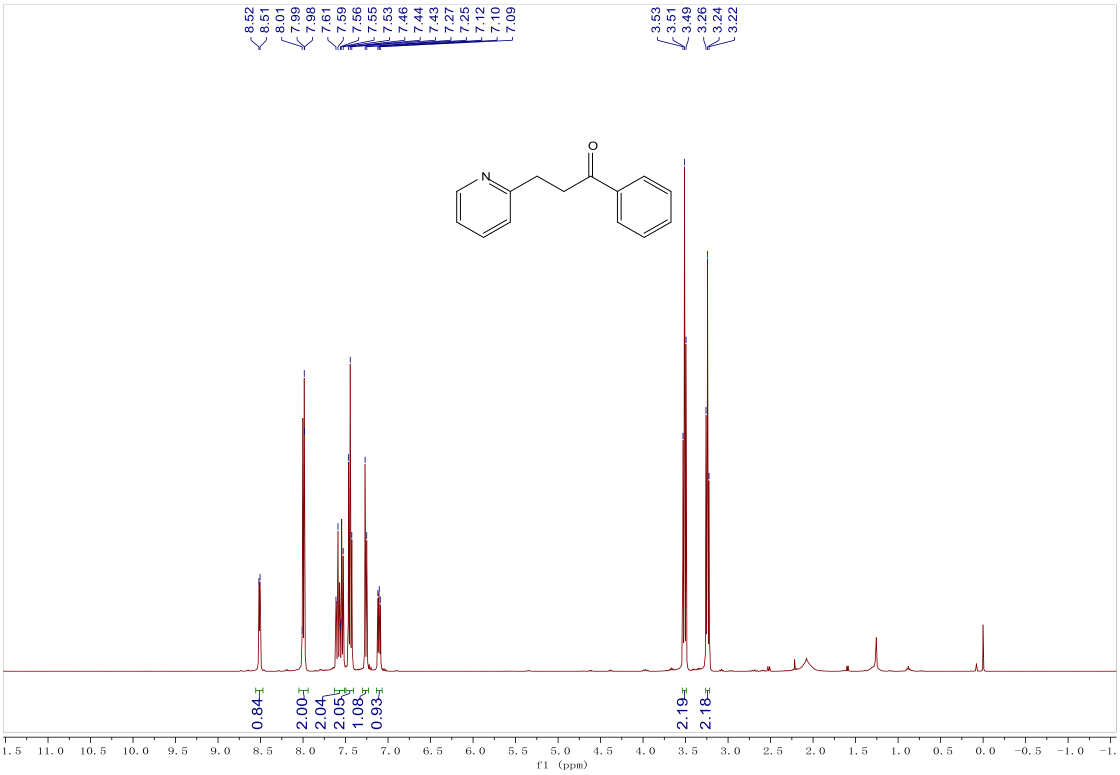

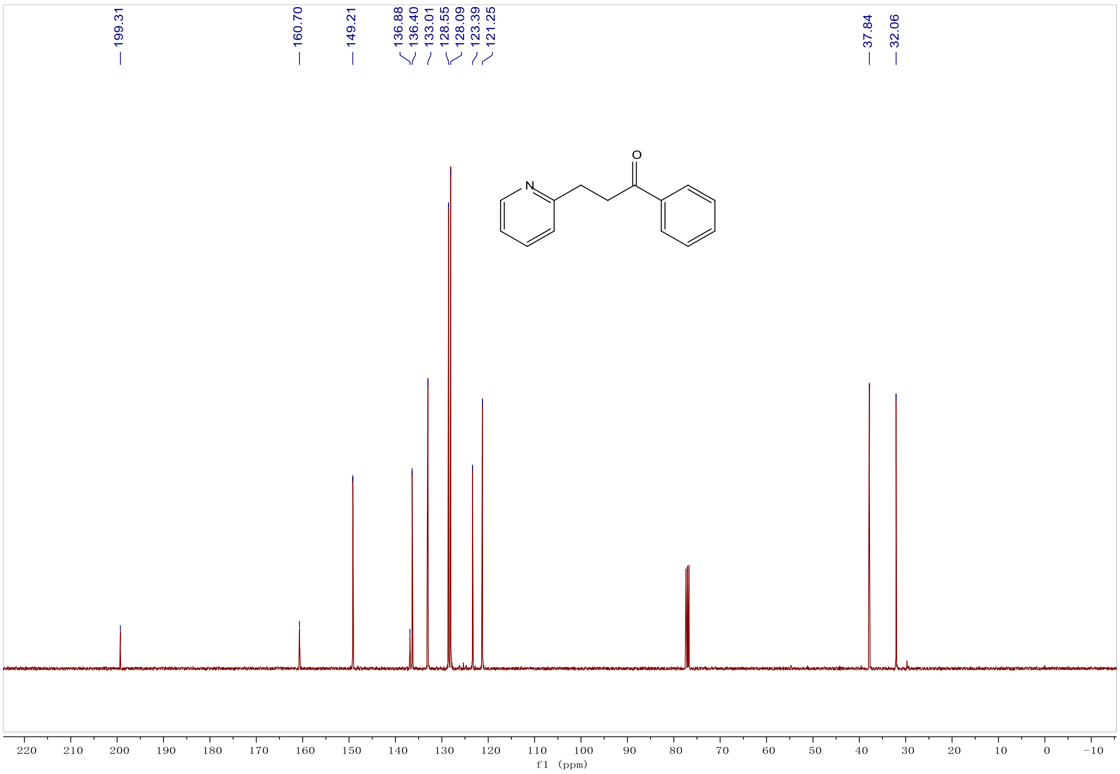

Supplementary Figure 18. ^{1}H and ^{13} C NMR spectra for compound **3d**

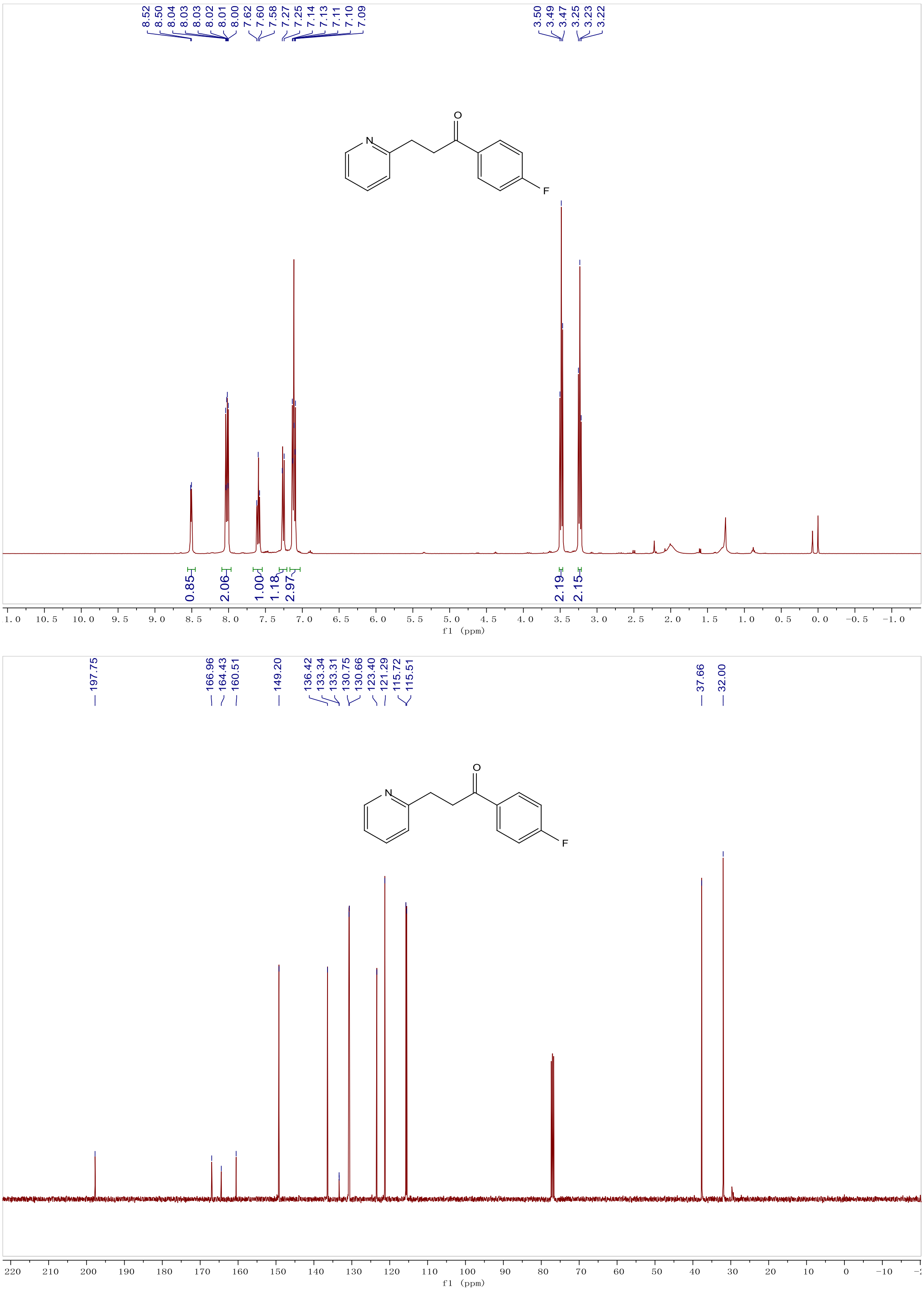

Supplementary Figure 19. ^{1}H and ^{13}C NMR spectra for compound **3e**

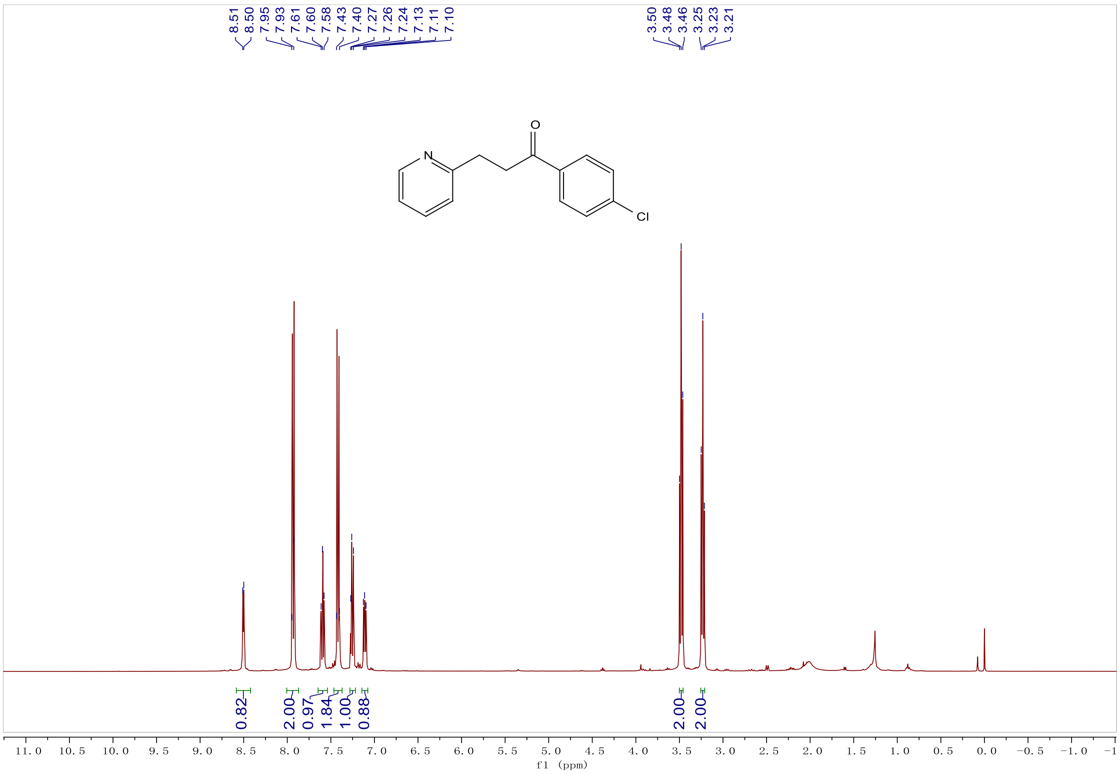

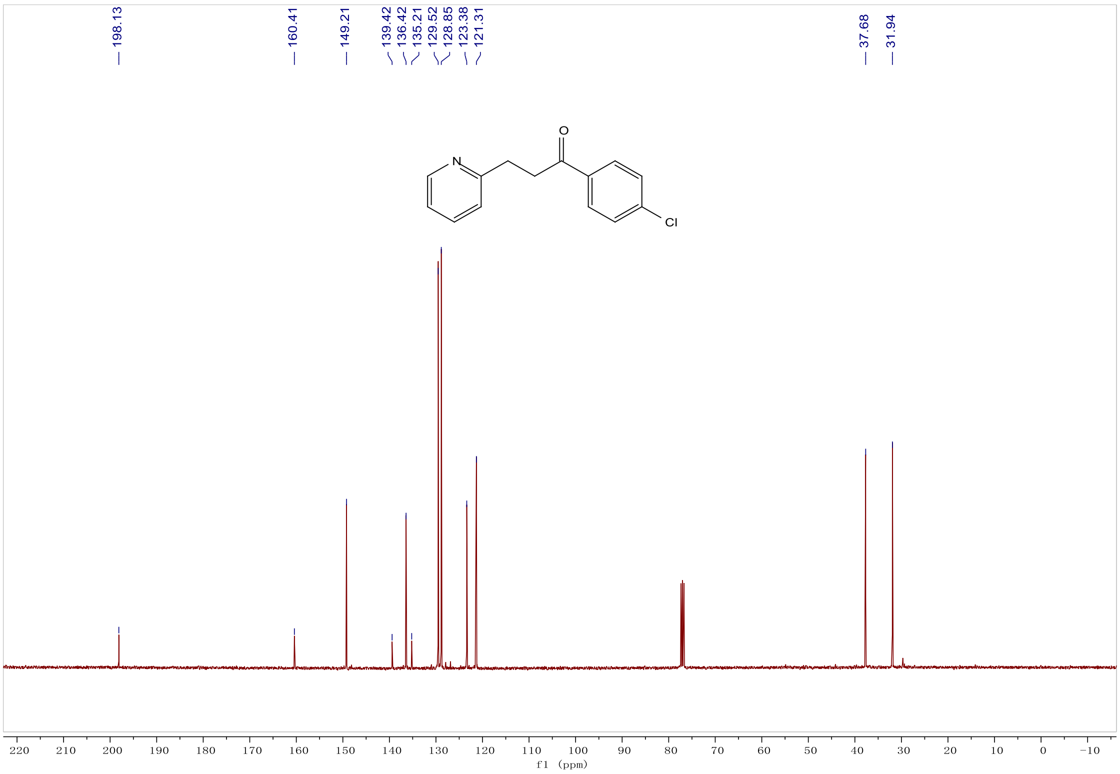

Supplementary Figure 20. ^{1}H and ^{13}C NMR spectra for compound **3f**

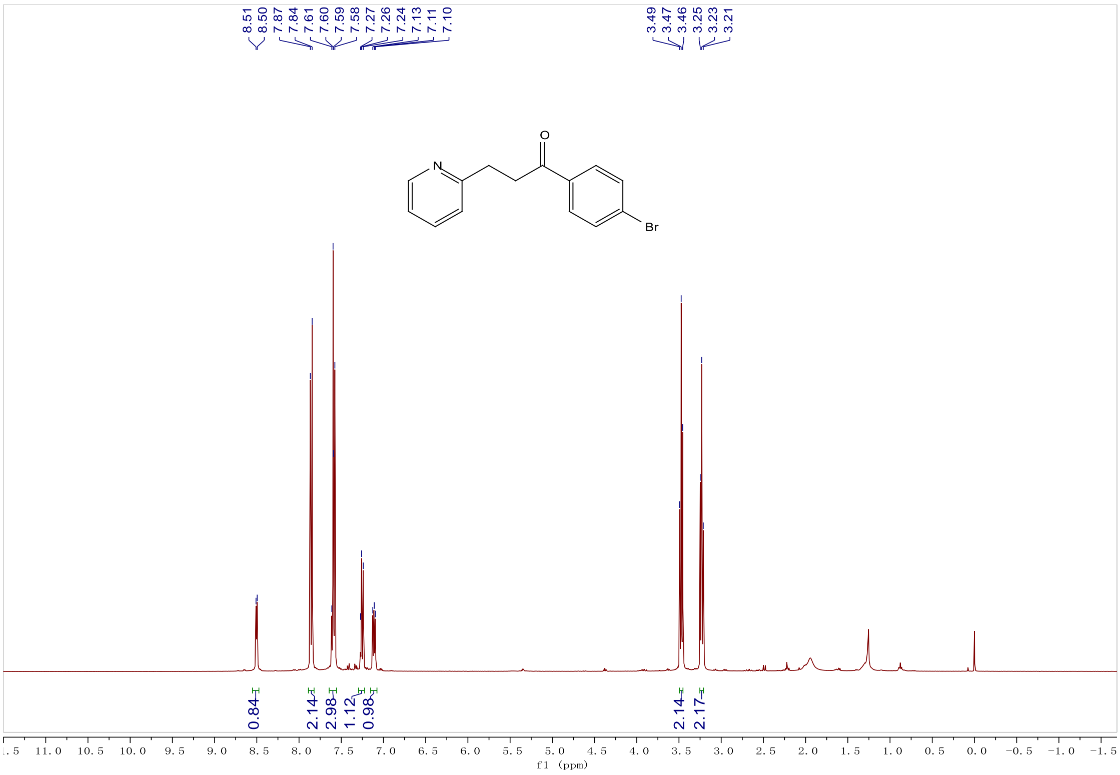

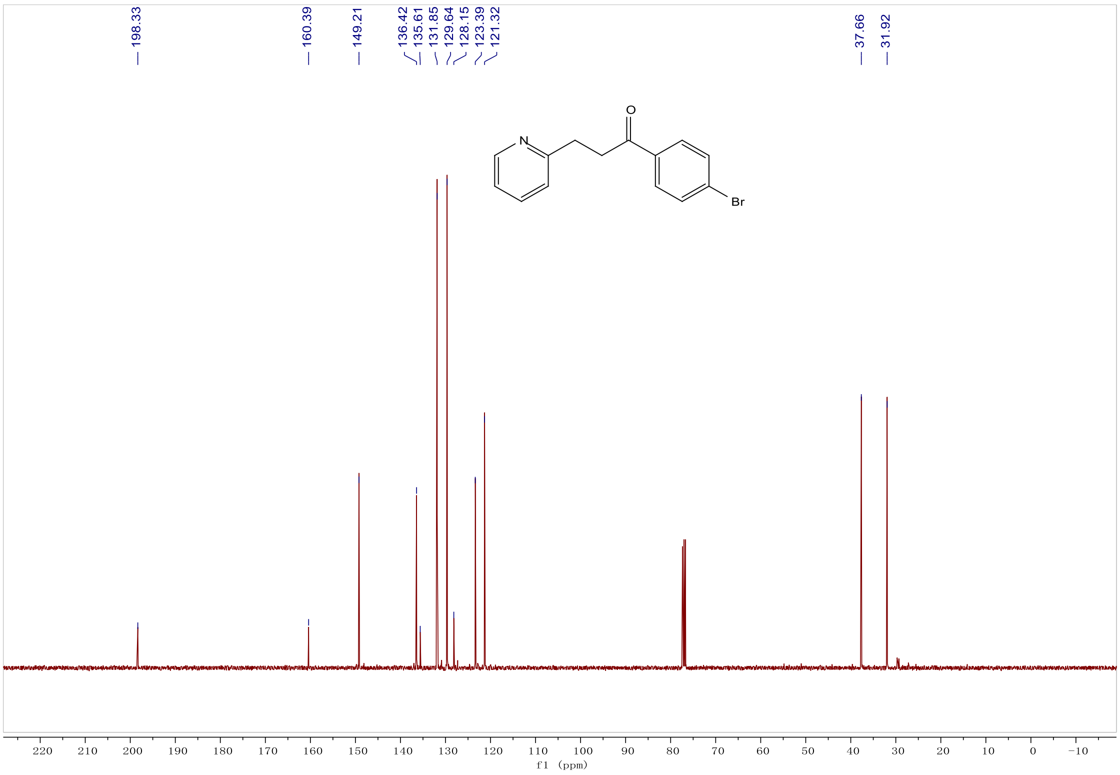

Supplementary Figure 21. ^{1}H and ^{13}C NMR spectra for compound **3g**

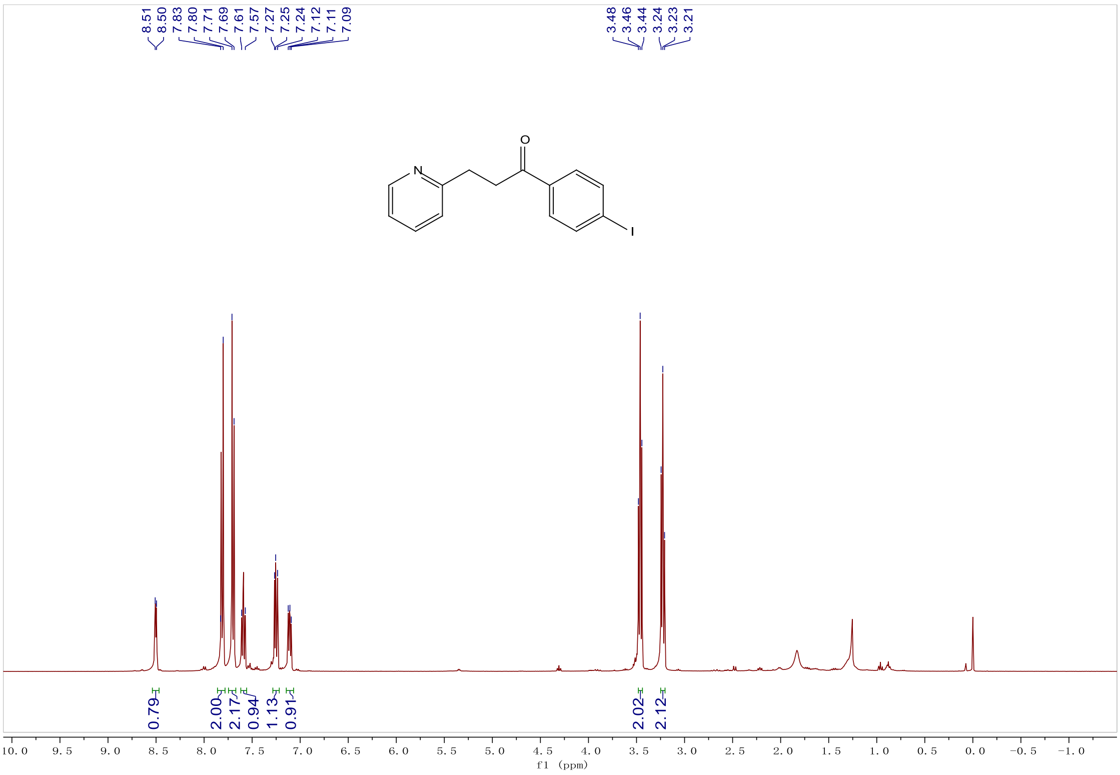

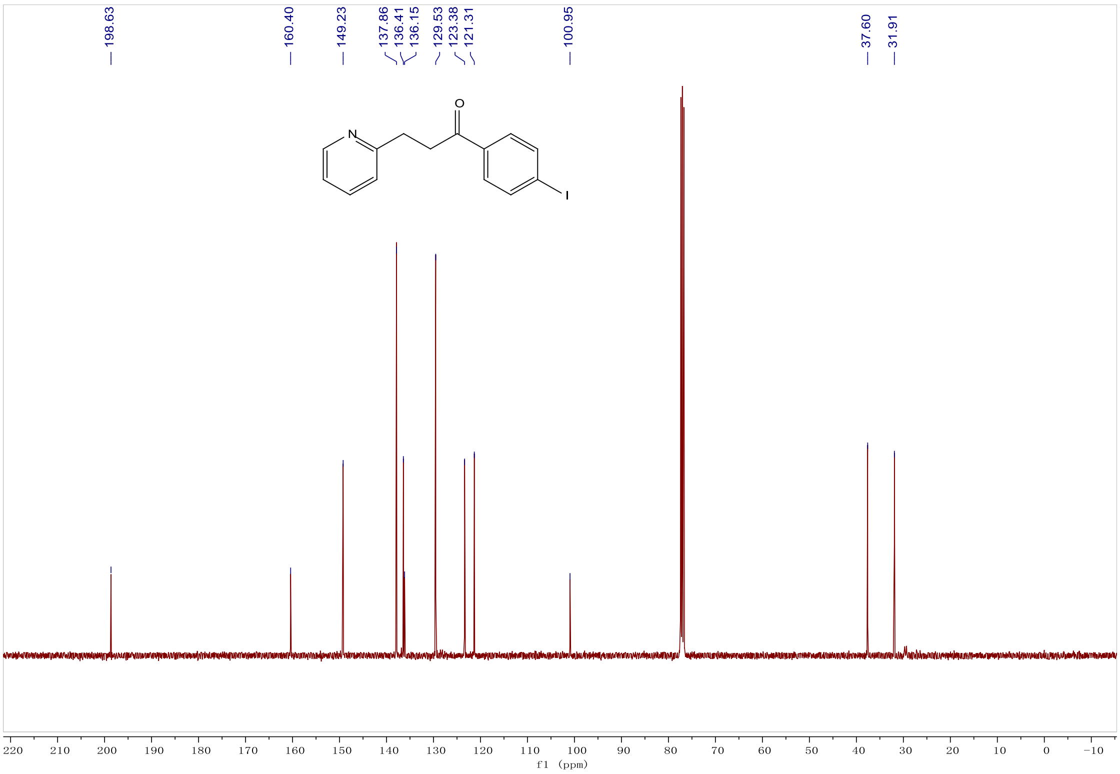

Supplementary Figure 22. ^{1}H and ^{13}C NMR spectra for compound **3h**

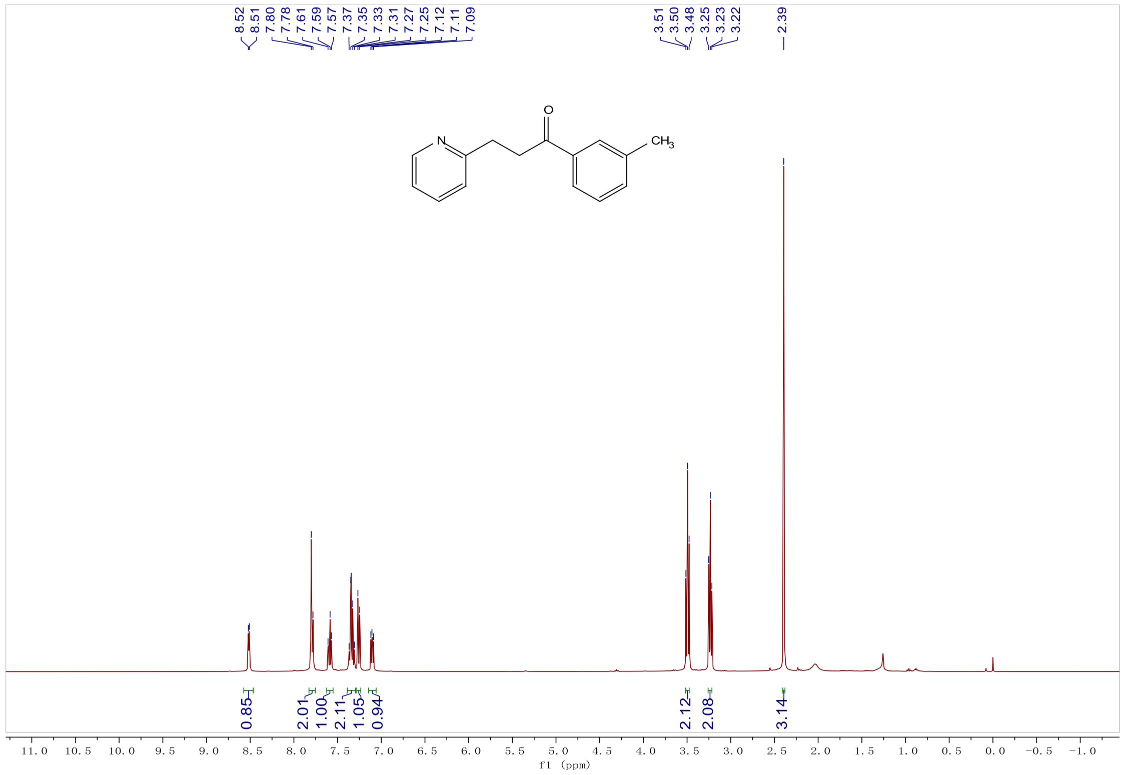

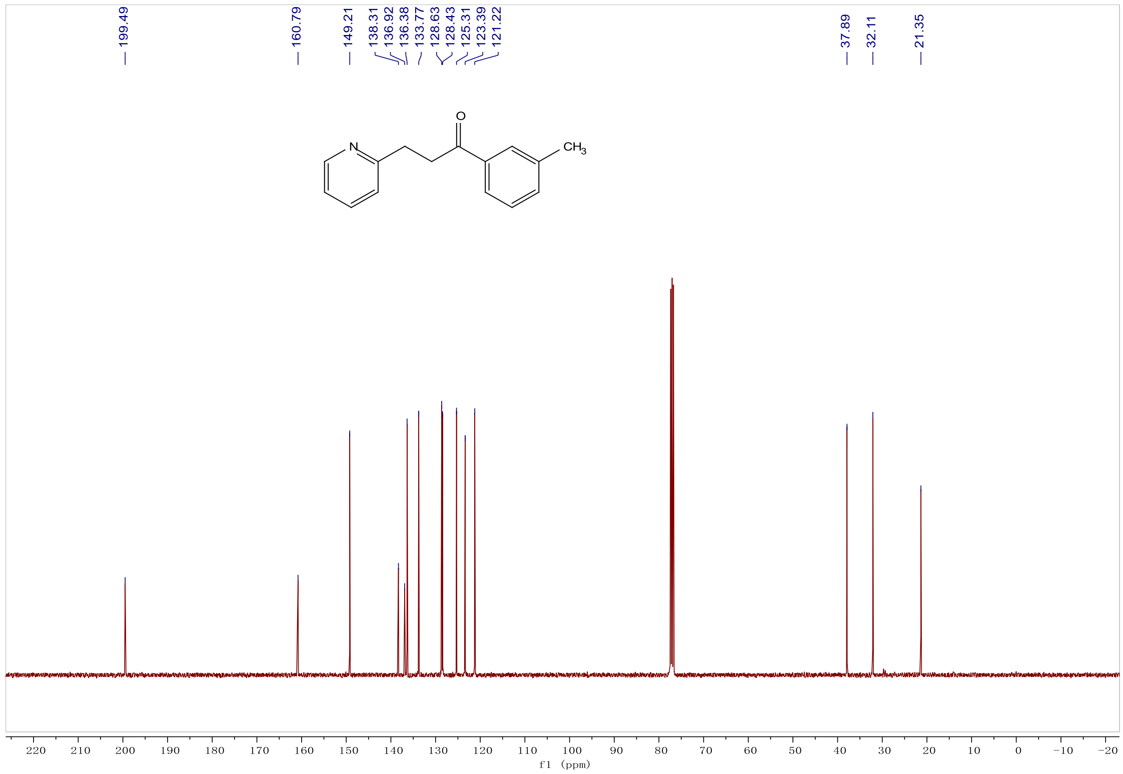

Supplementary Figure 23. ^{1}H and ^{13}C NMR spectra for compound **3i**

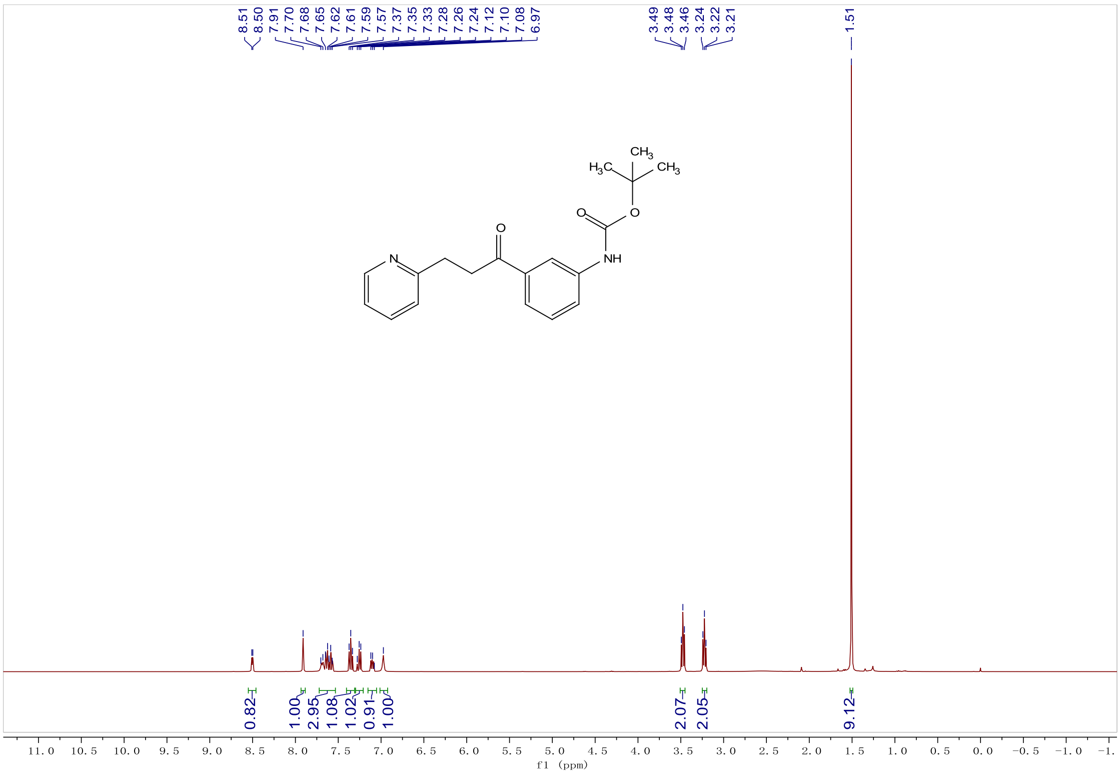

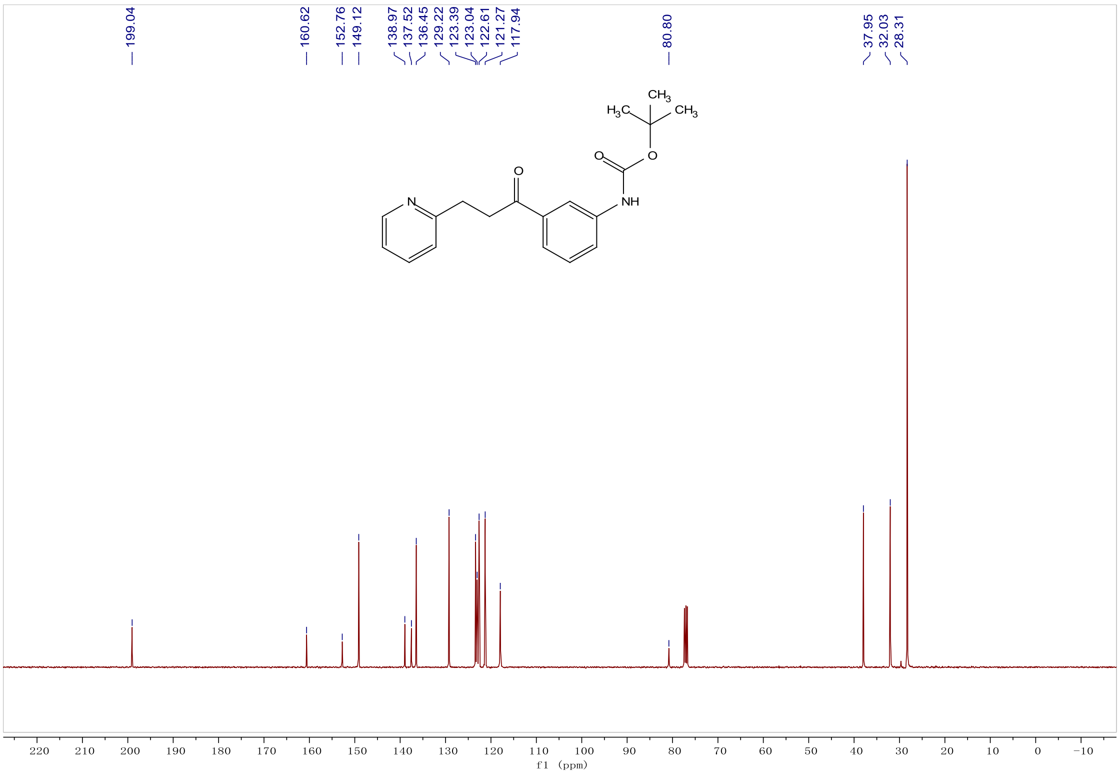

Supplementary Figure 24. ^{1}H and ^{13}C NMR spectra for compound **3j**

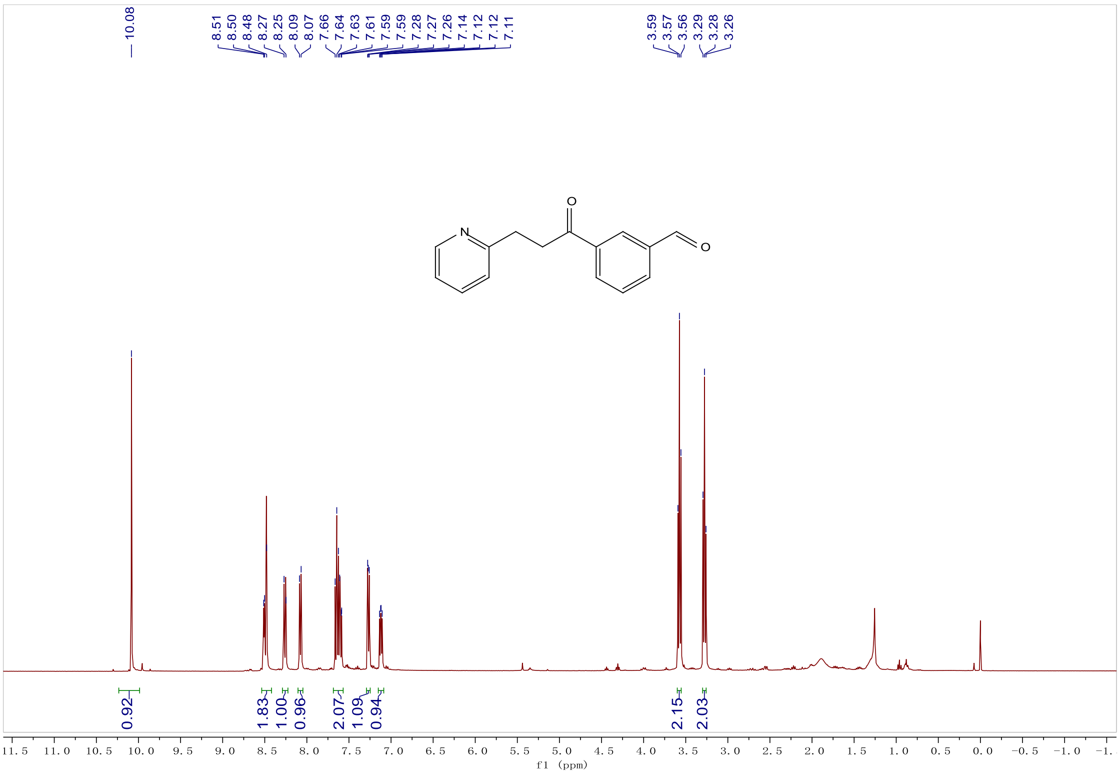

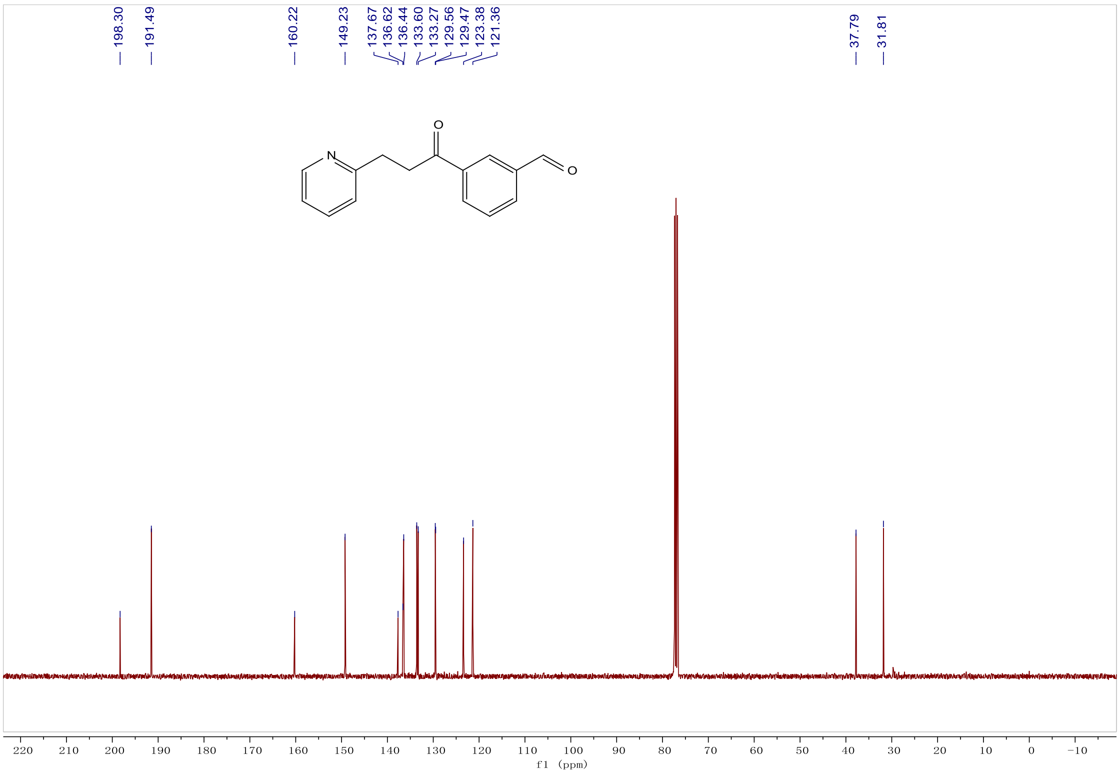

Supplementary Figure 25. ^{1}H and ^{13}C NMR spectra for compound **3k**

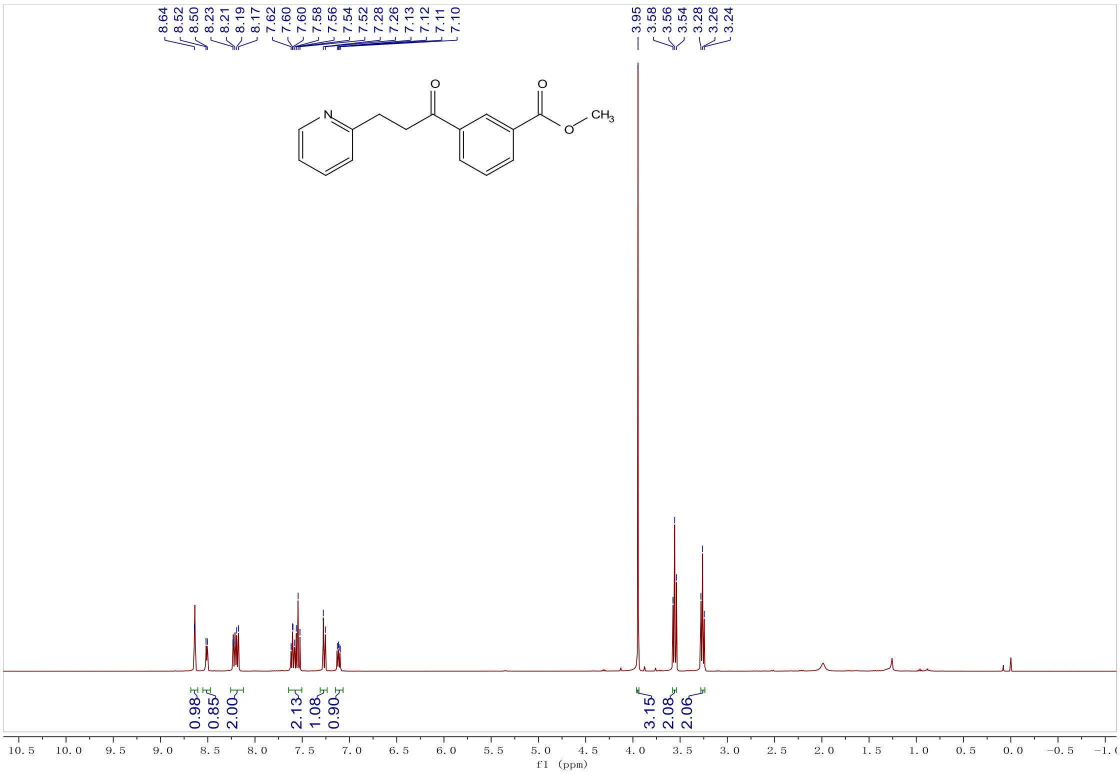

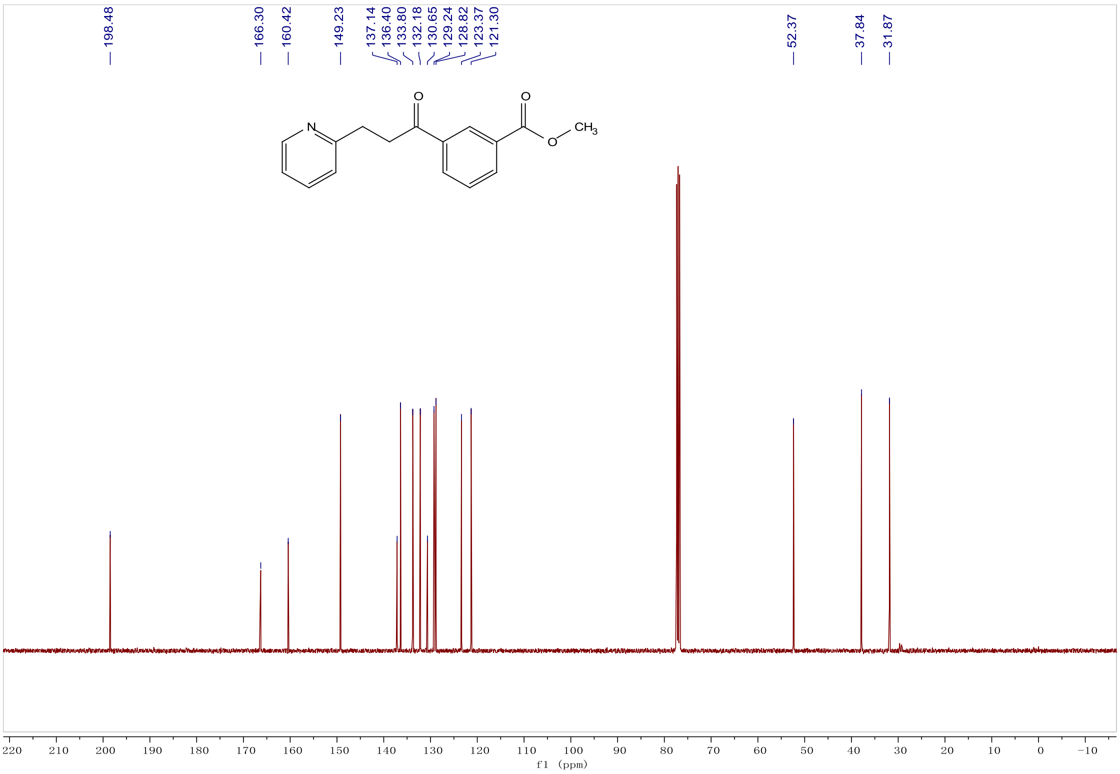

Supplementary Figure 26. ^{1}H and ^{13} C NMR spectra for compound **3l**

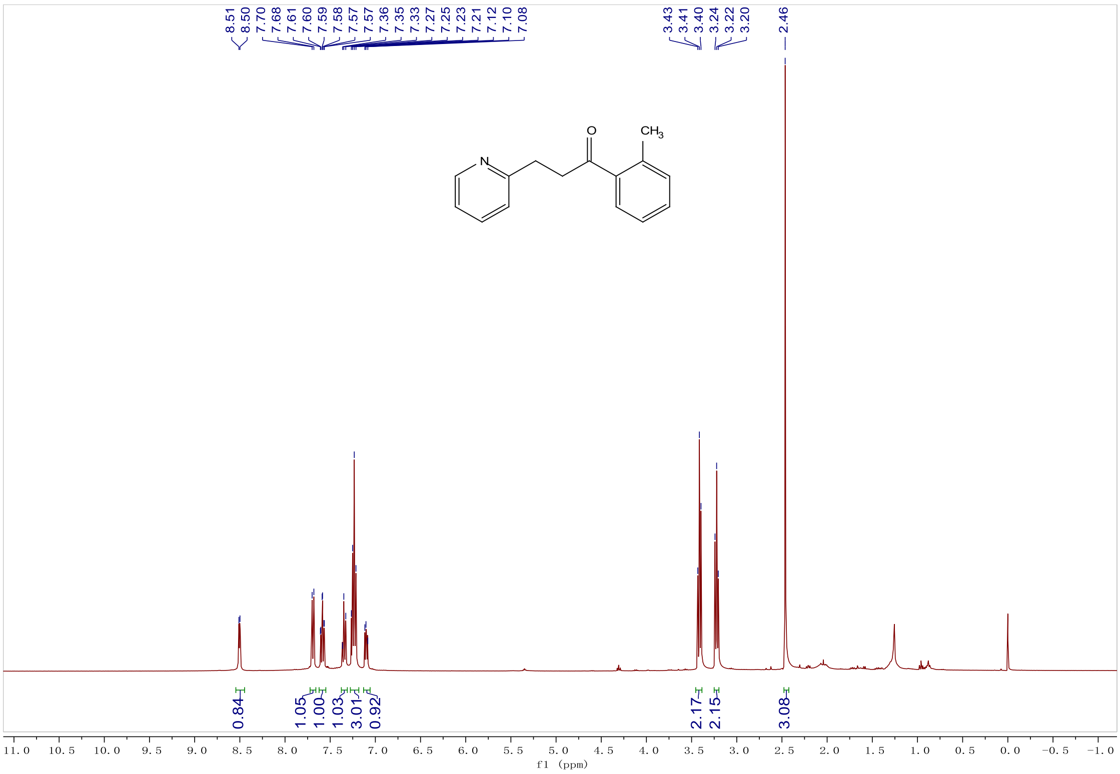

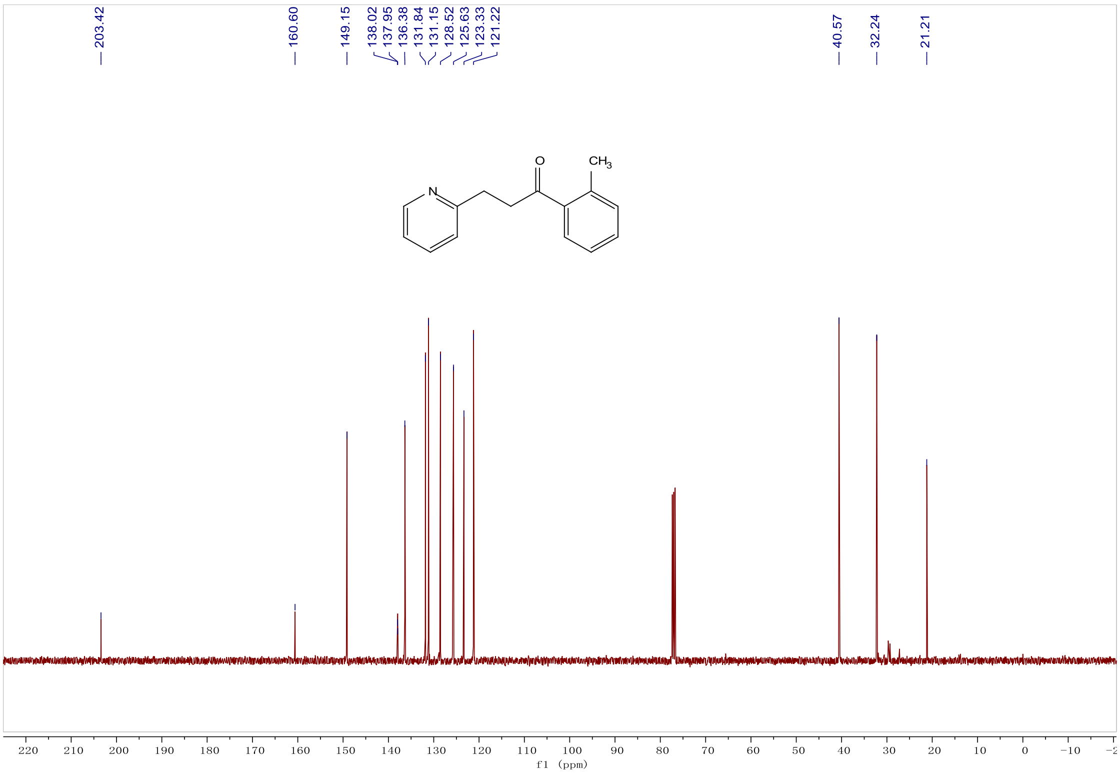

Supplementary Figure 27. ^{1}H and ^{13}C NMR spectra for compound **3m**

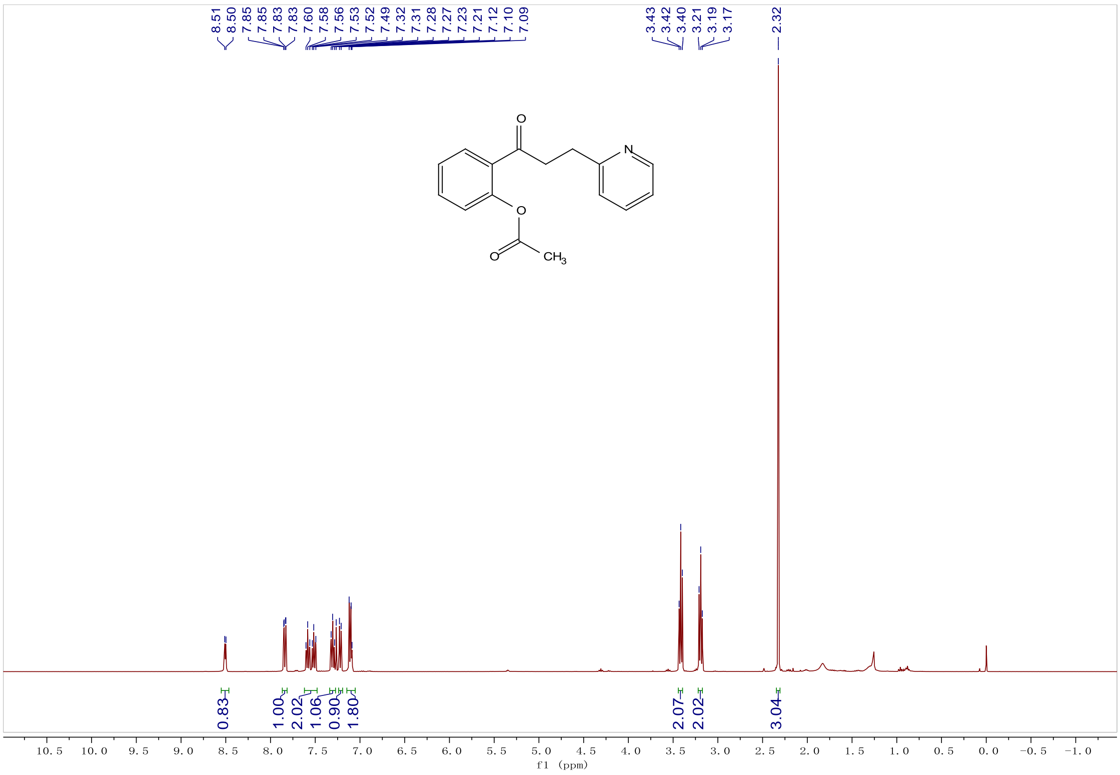

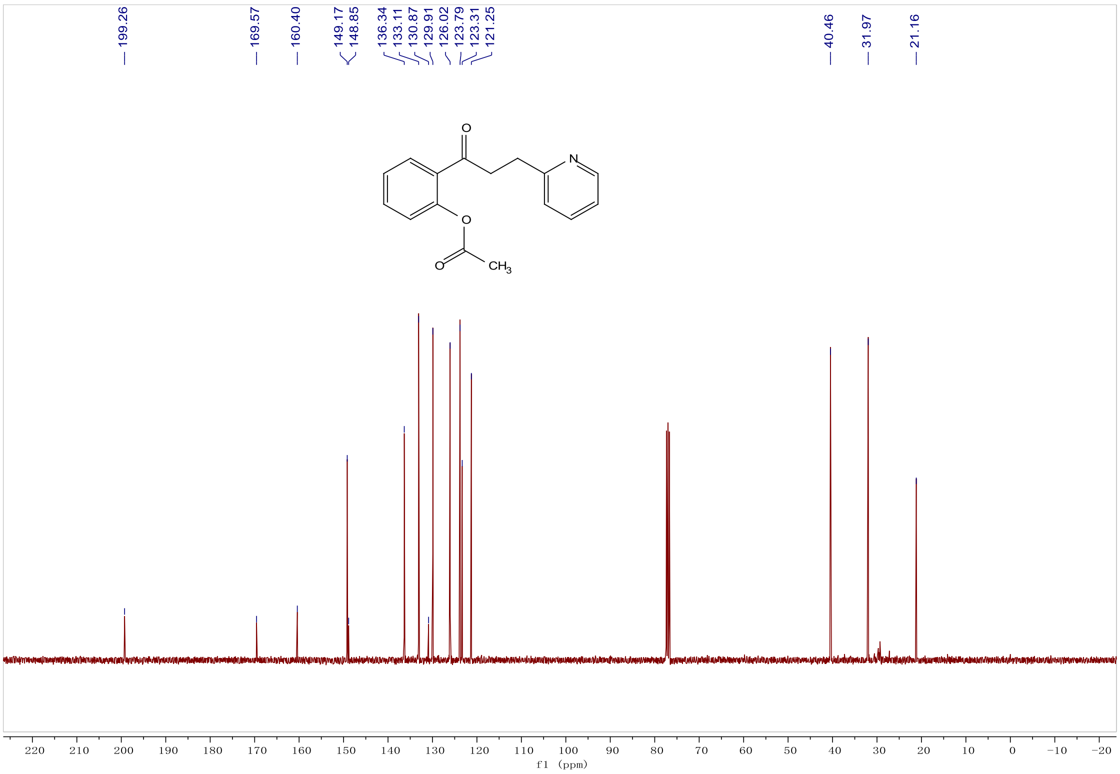

Supplementary Figure 28. ^{1}H and ^{13}C NMR spectra for compound **3n**

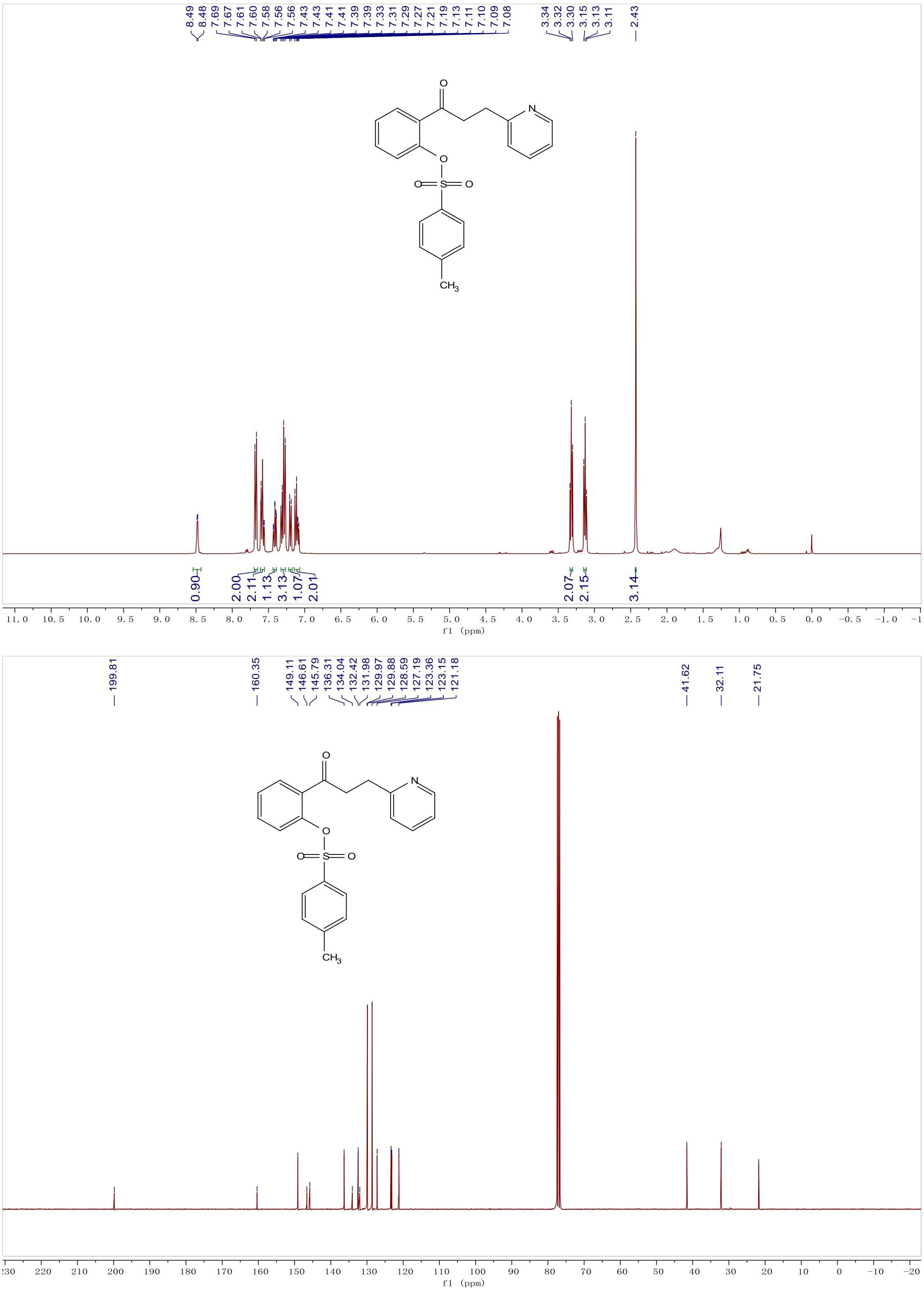

Supplementary Figure 29. ^{1}H and ^{13} C NMR spectra for compound **3o**

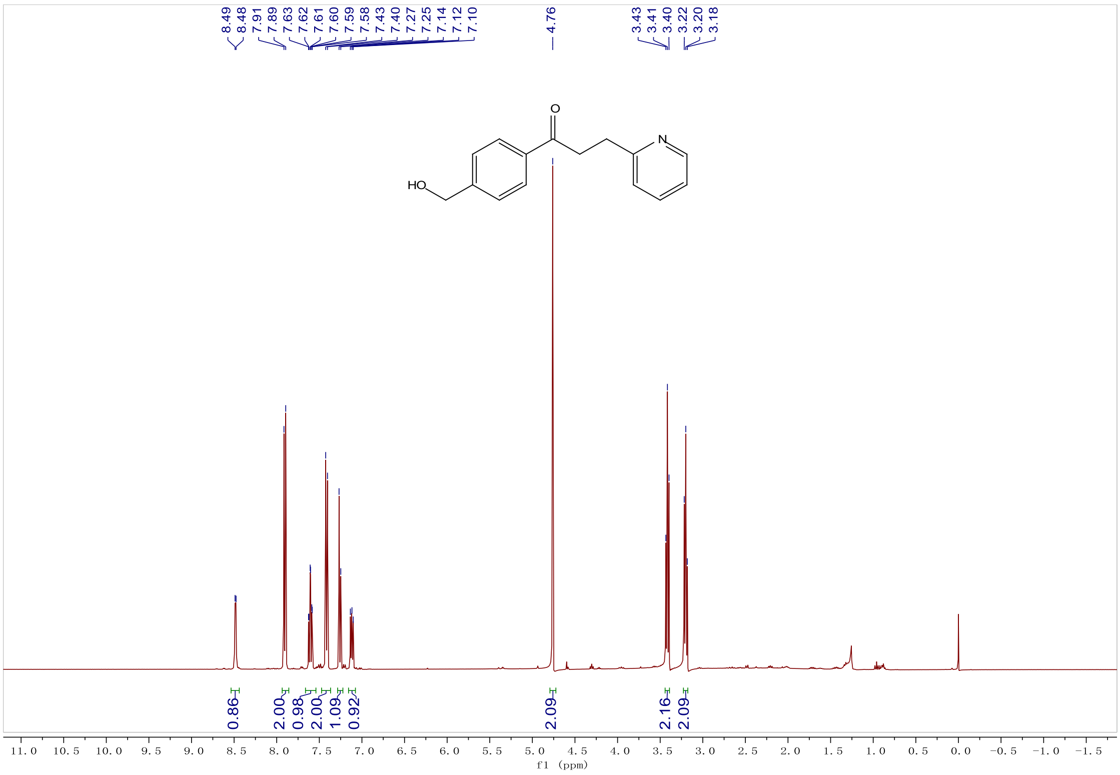

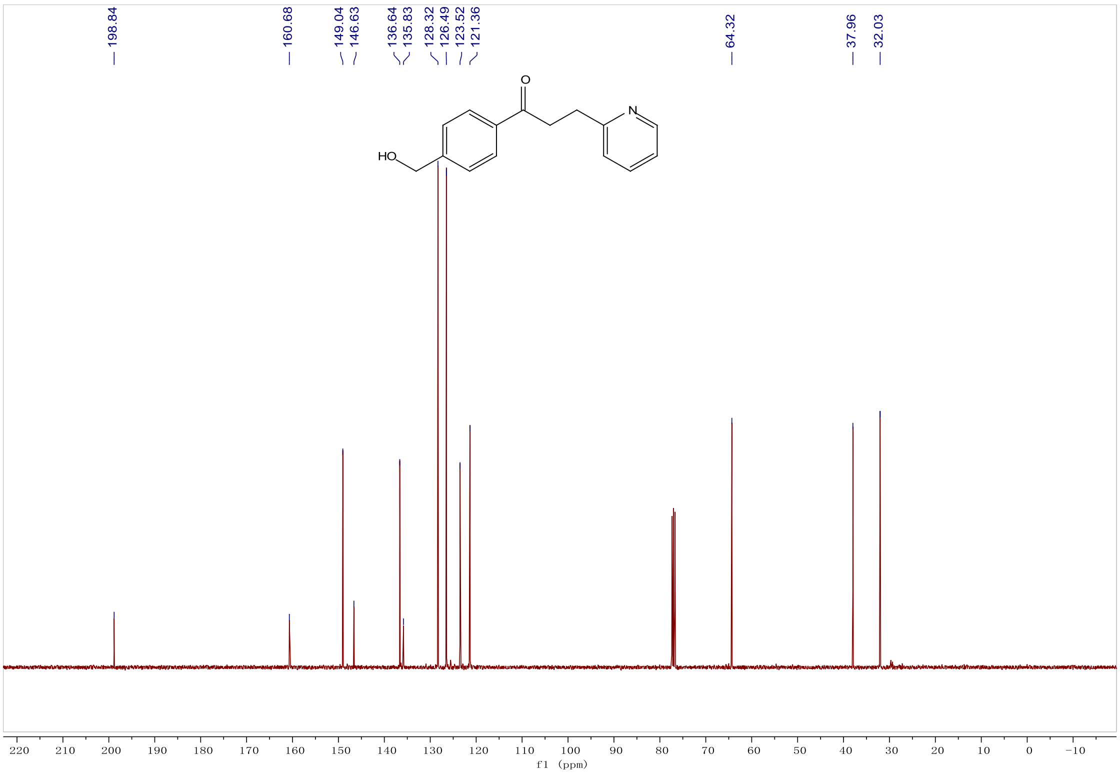

Supplementary Figure 30. ^{1}H and ^{13} C NMR spectra for compound **3p**

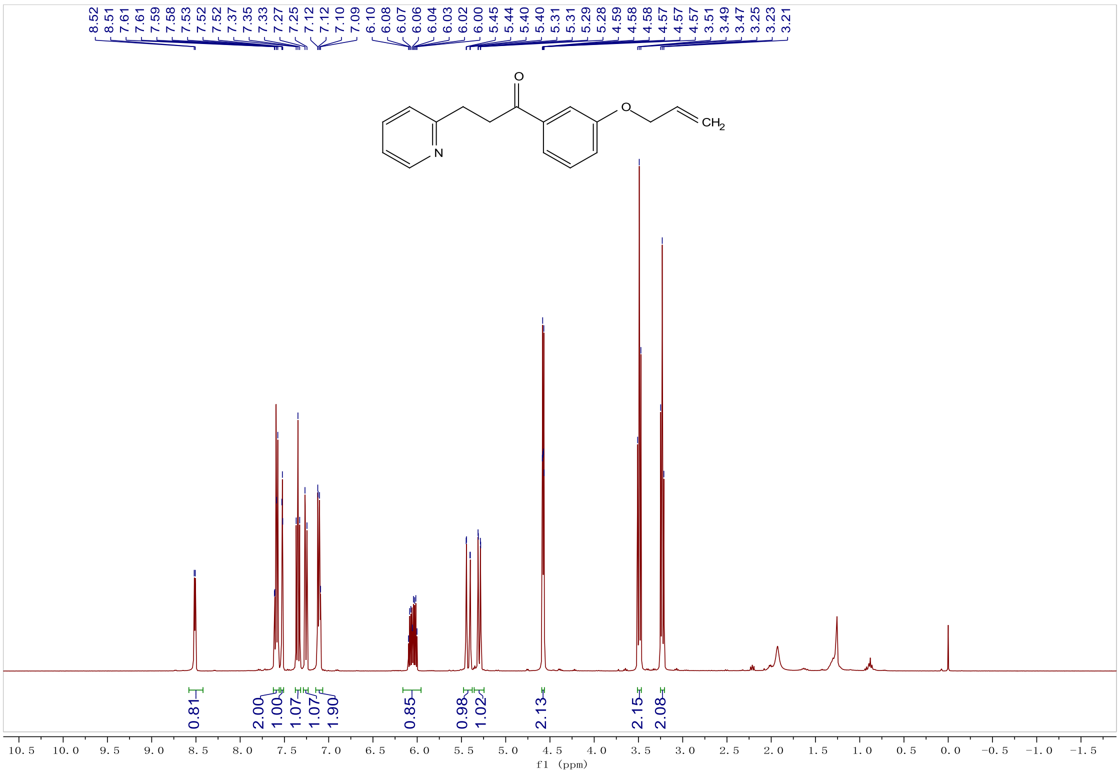

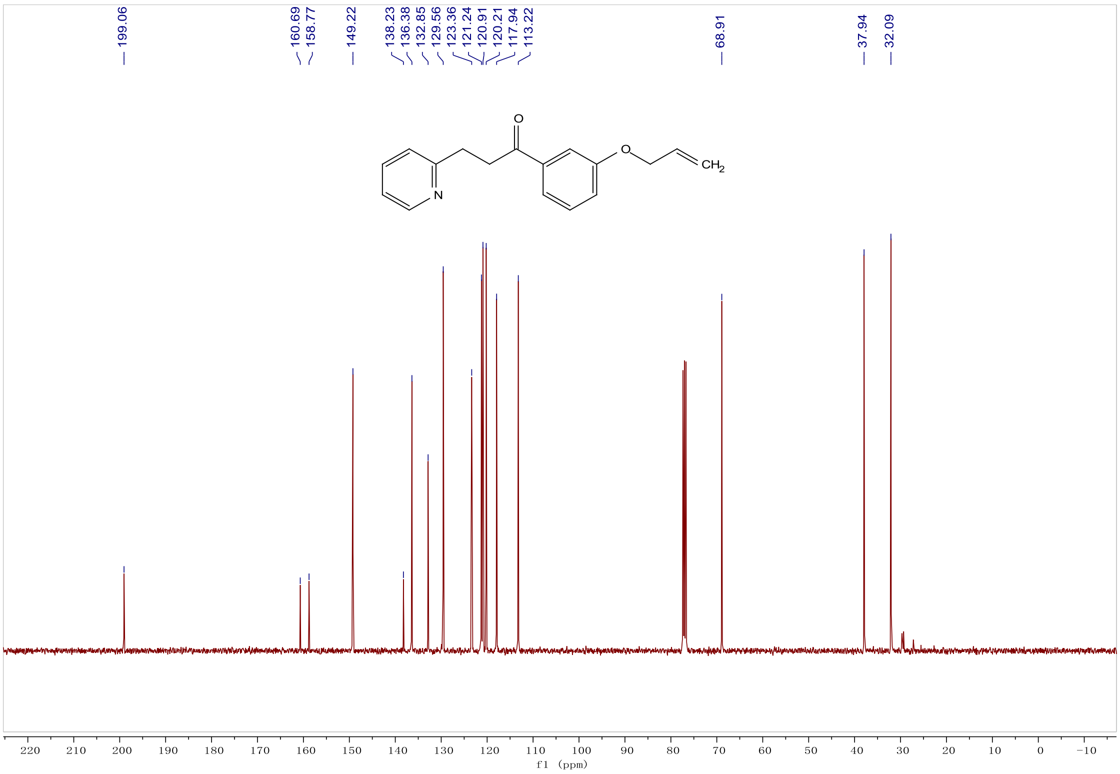

Supplementary Figure 31. ^{1}H and ^{13} C NMR spectra for compound **3q**

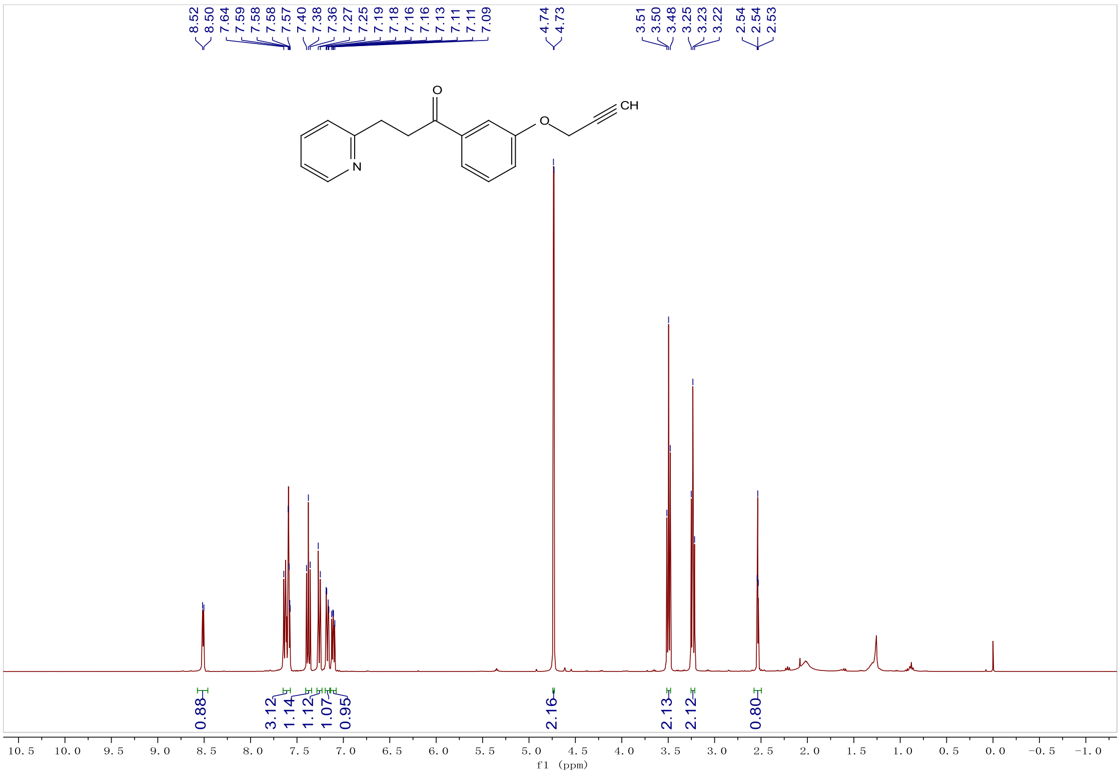

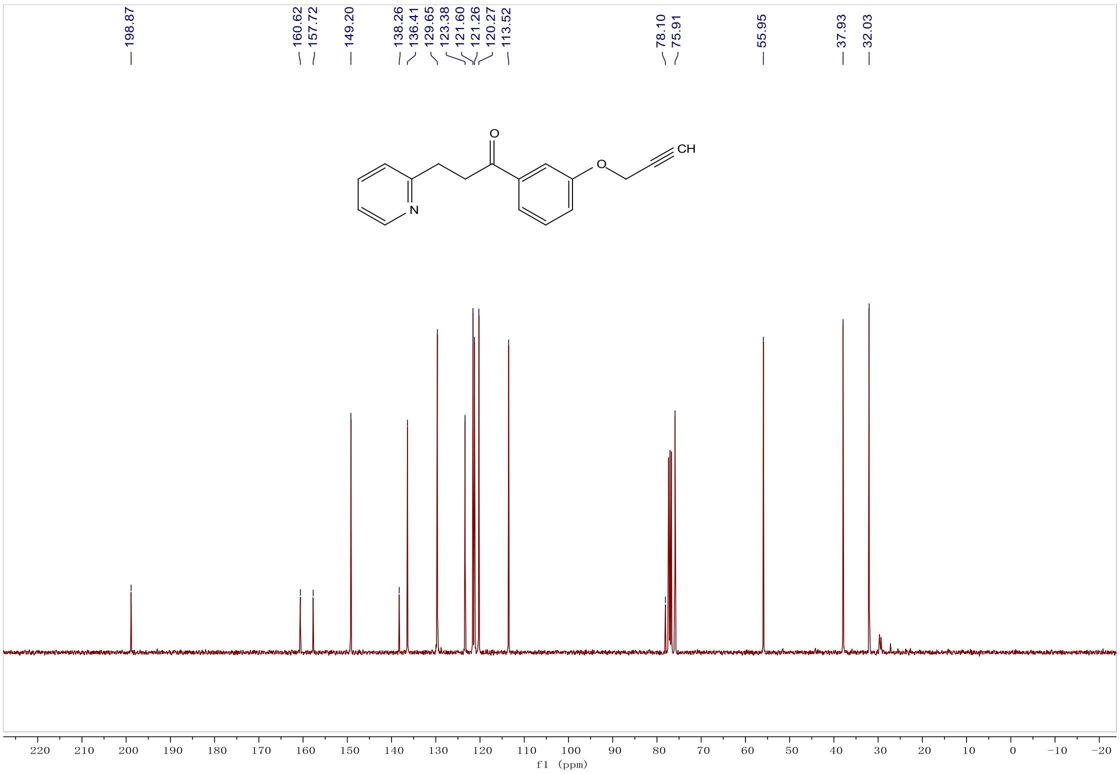

Supplementary Figure 32. ^{1}H and ^{13} C NMR spectra for compound **3r**

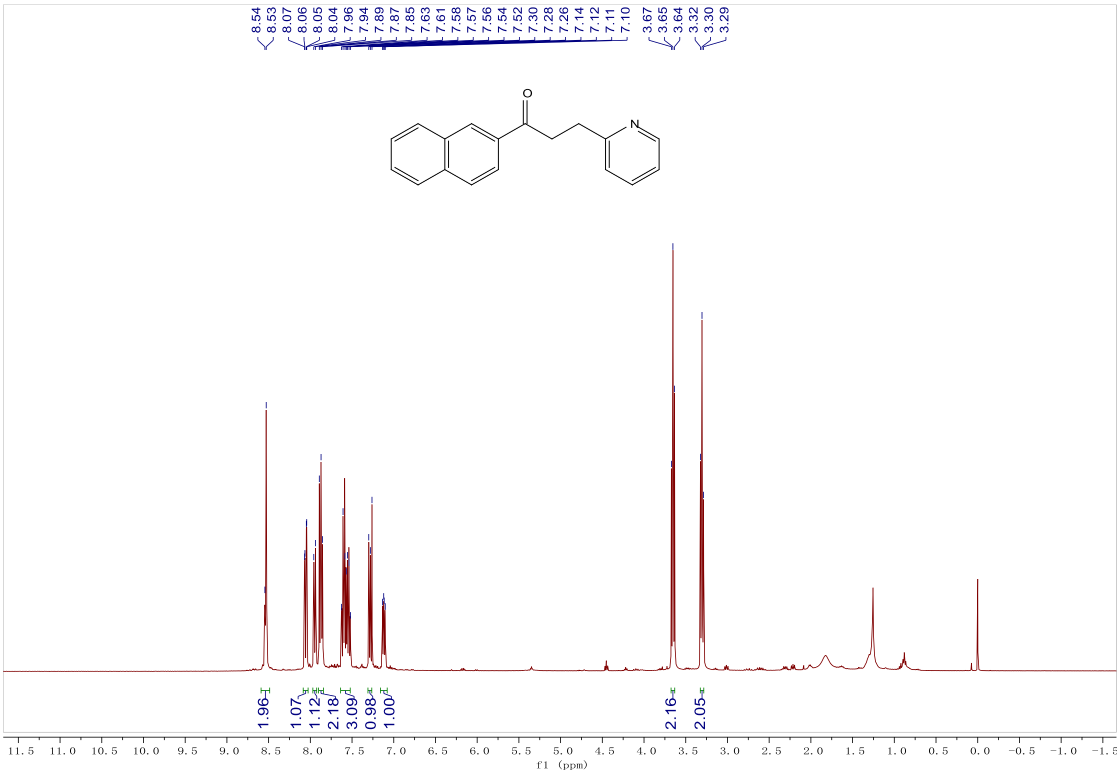

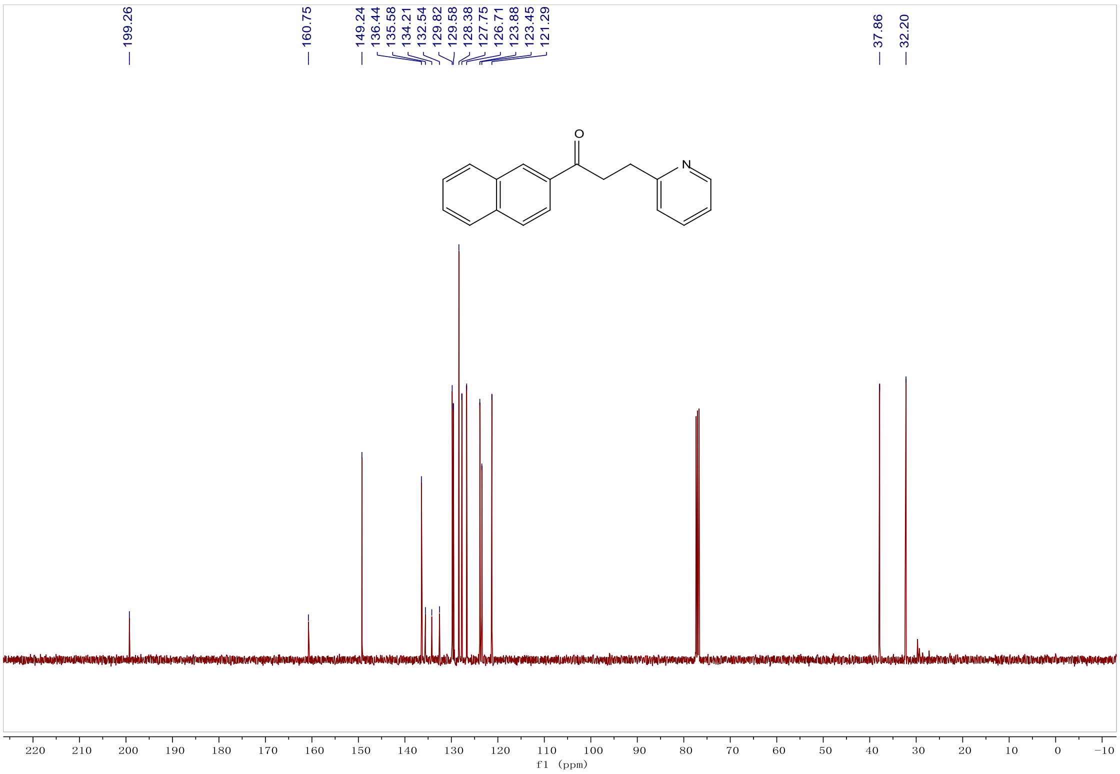

Supplementary Figure 33. ^{1}H and ^{13}C NMR spectra for compound **3s**

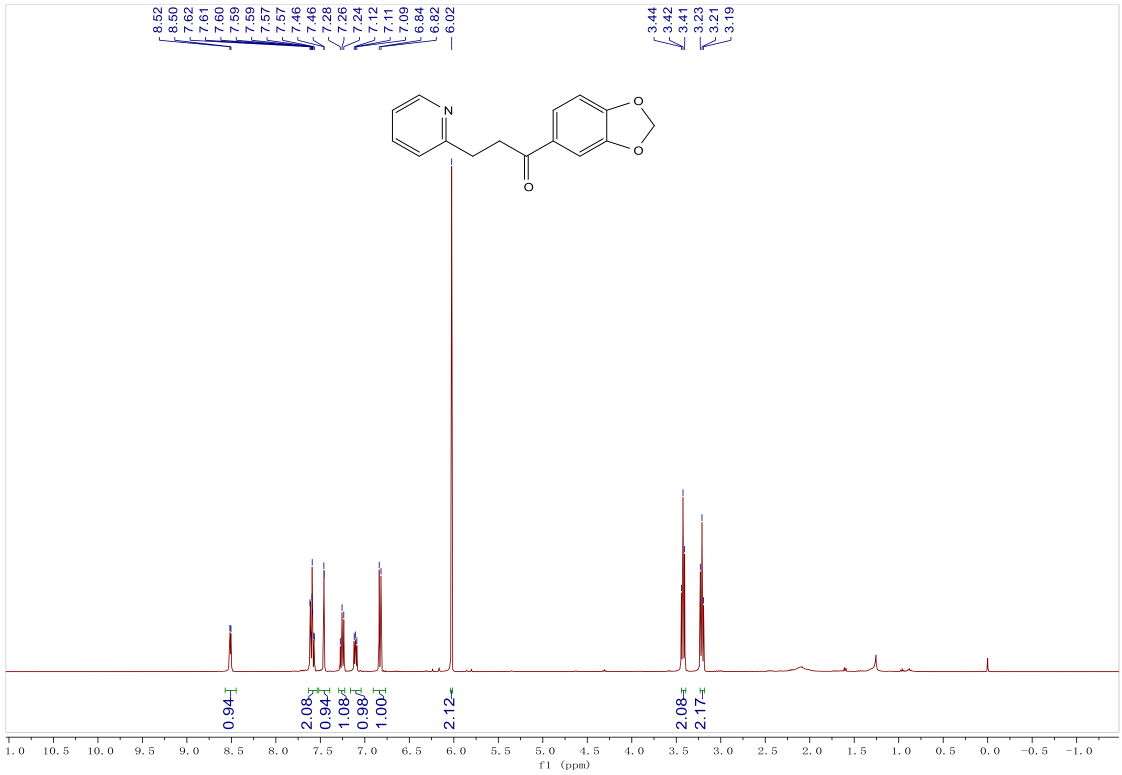

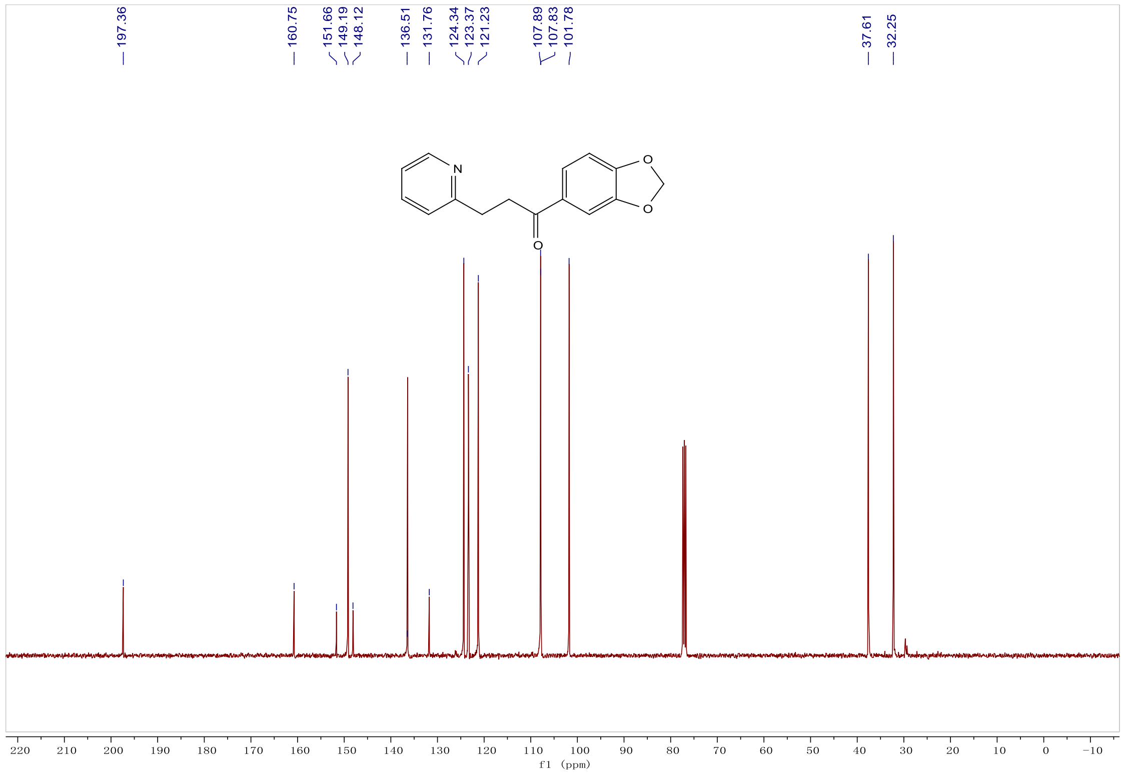

Supplementary Figure 34. ^{1}H and ^{13} C NMR spectra for compound **3t**

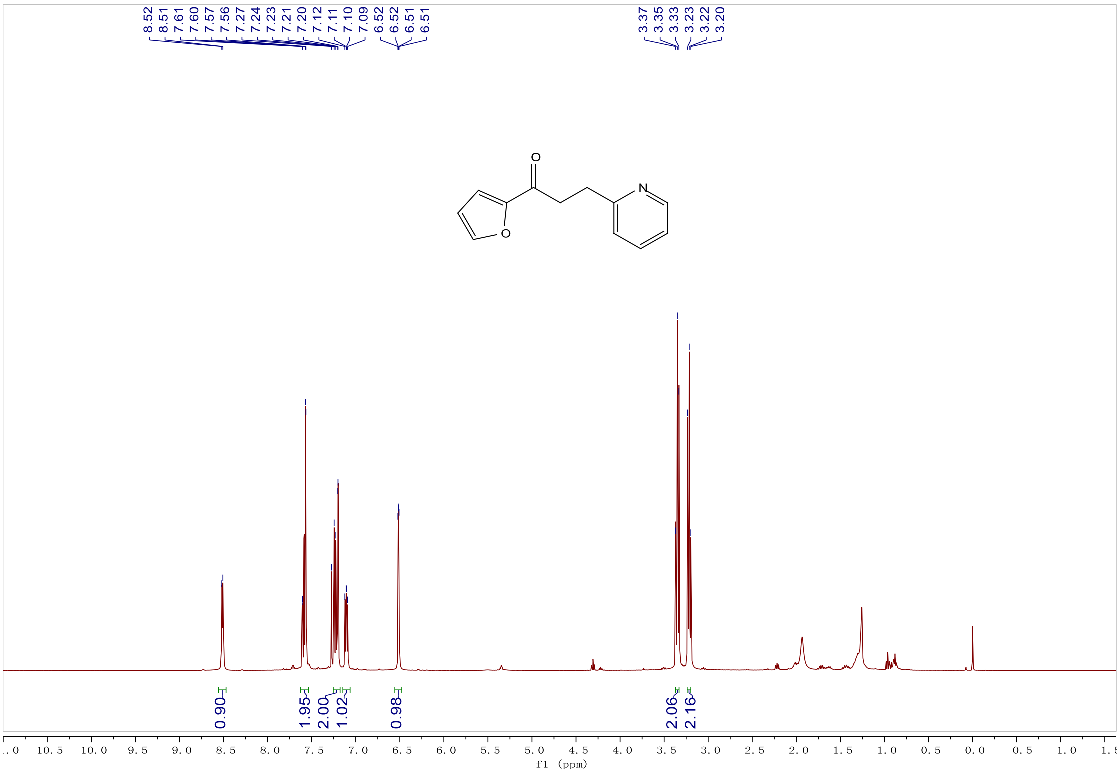

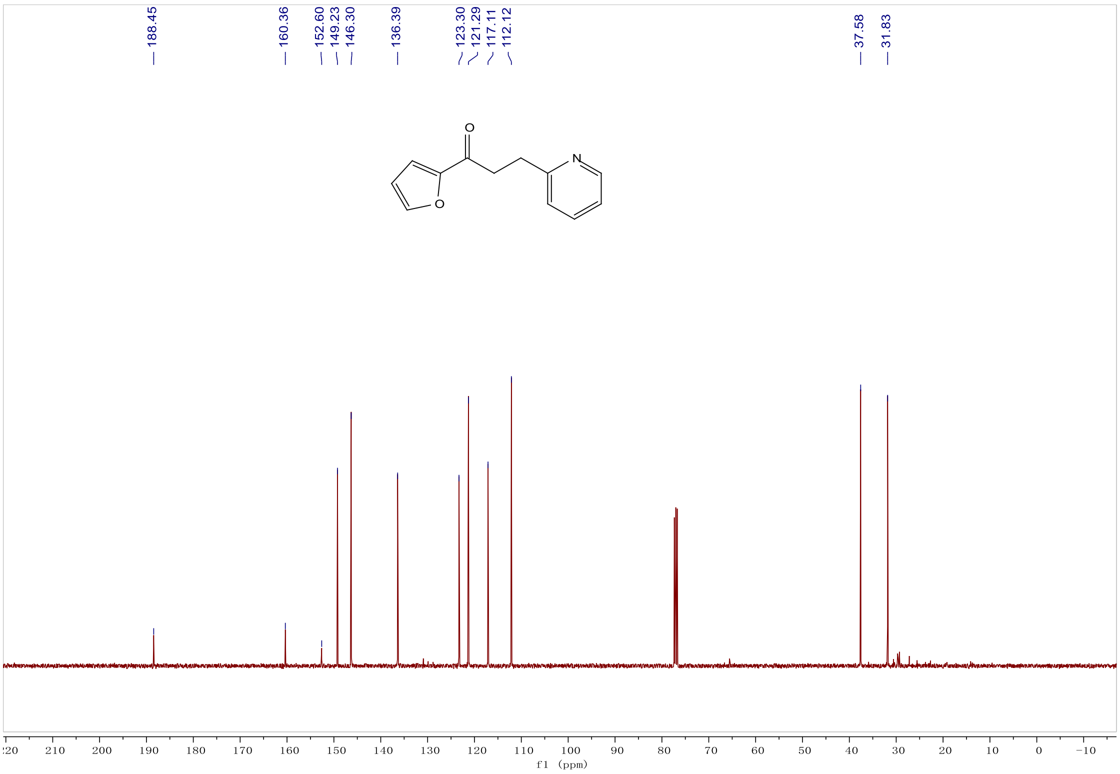

Supplementary Figure 35. ^{1}H and ^{13} C NMR spectra for compound **3u**

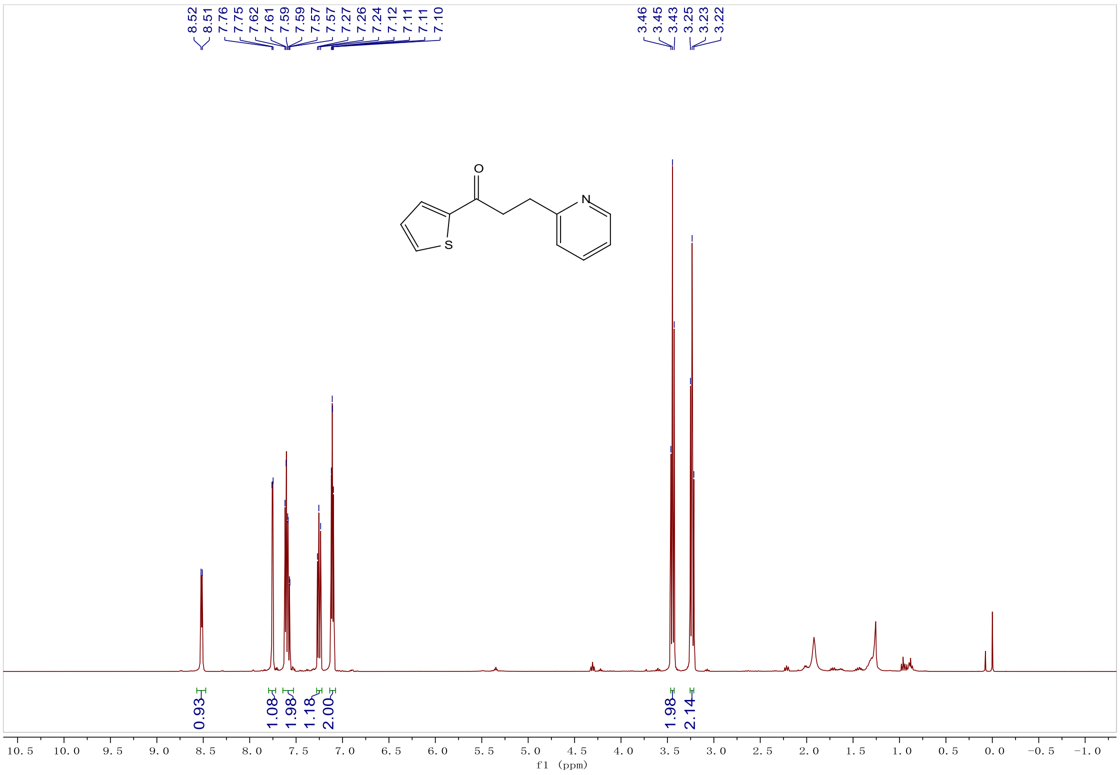

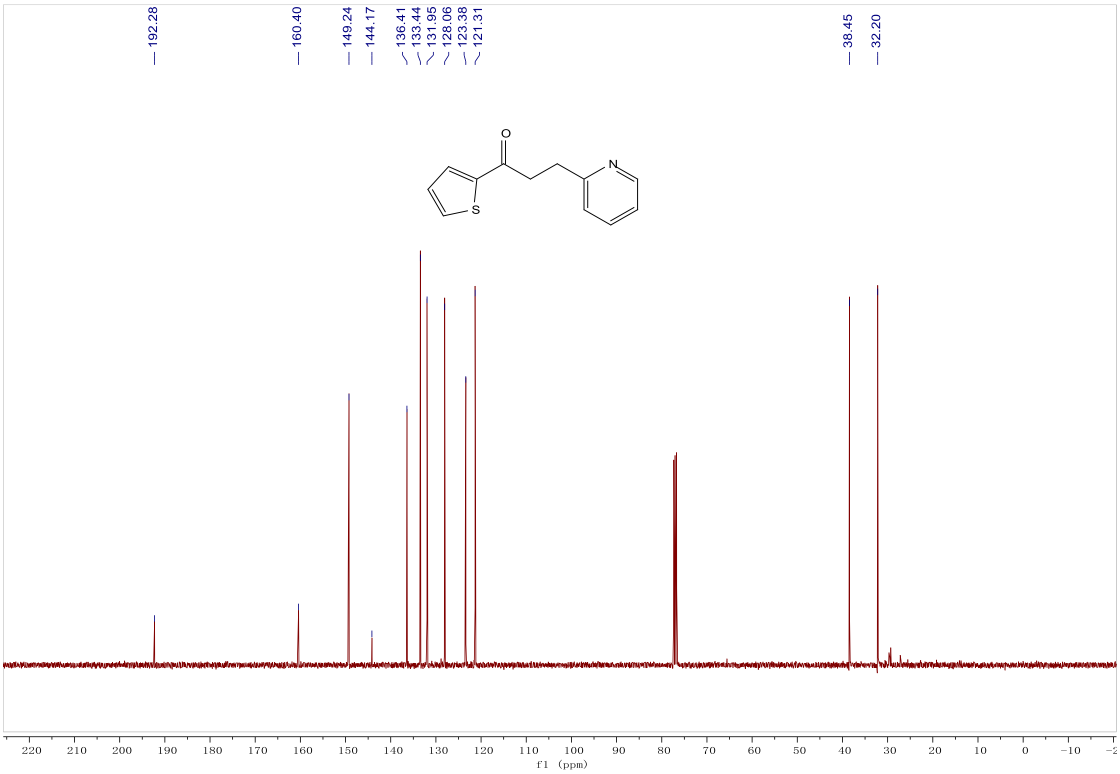

Supplementary Figure 36. ^{1}H and ^{13}C NMR spectra for compound **3v**

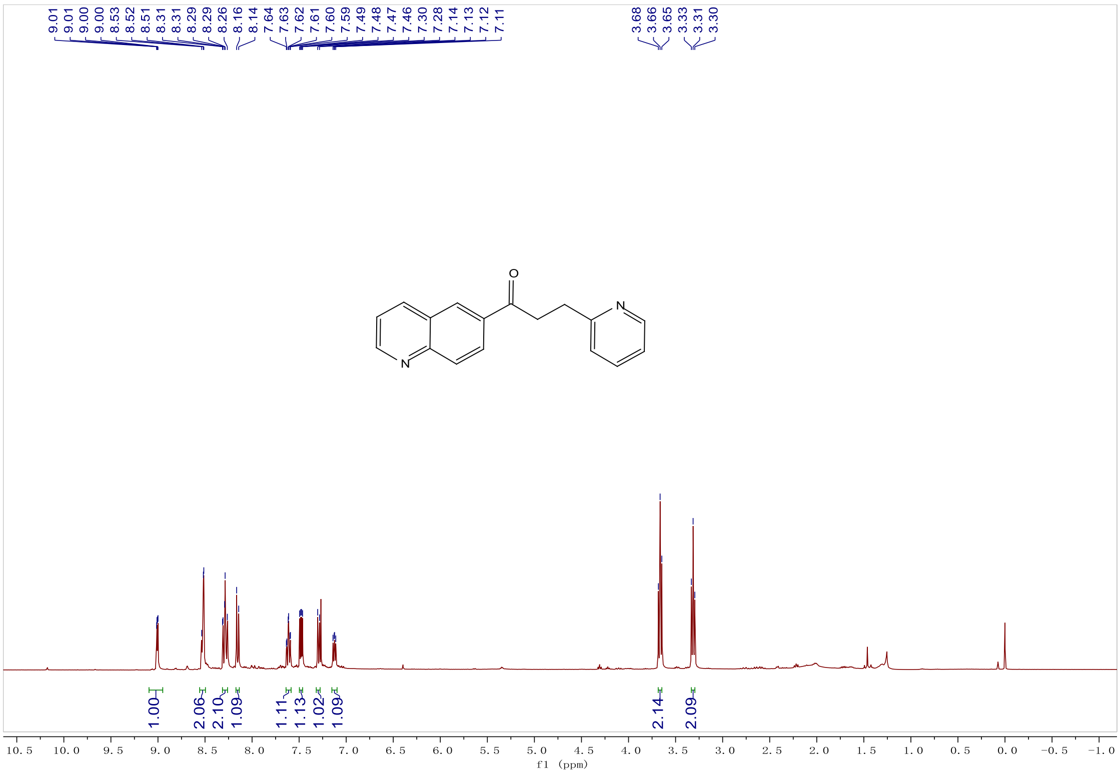

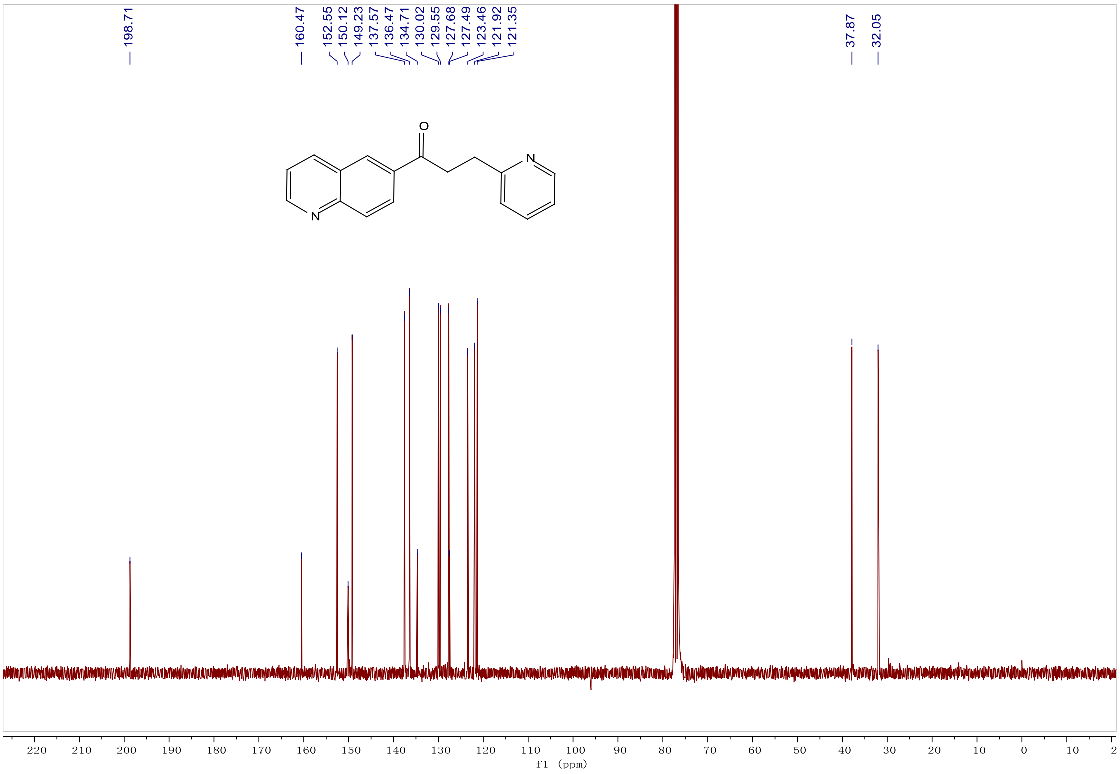

Supplementary Figure 37. ^{1}H and ^{13}C NMR spectra for compound **3w**

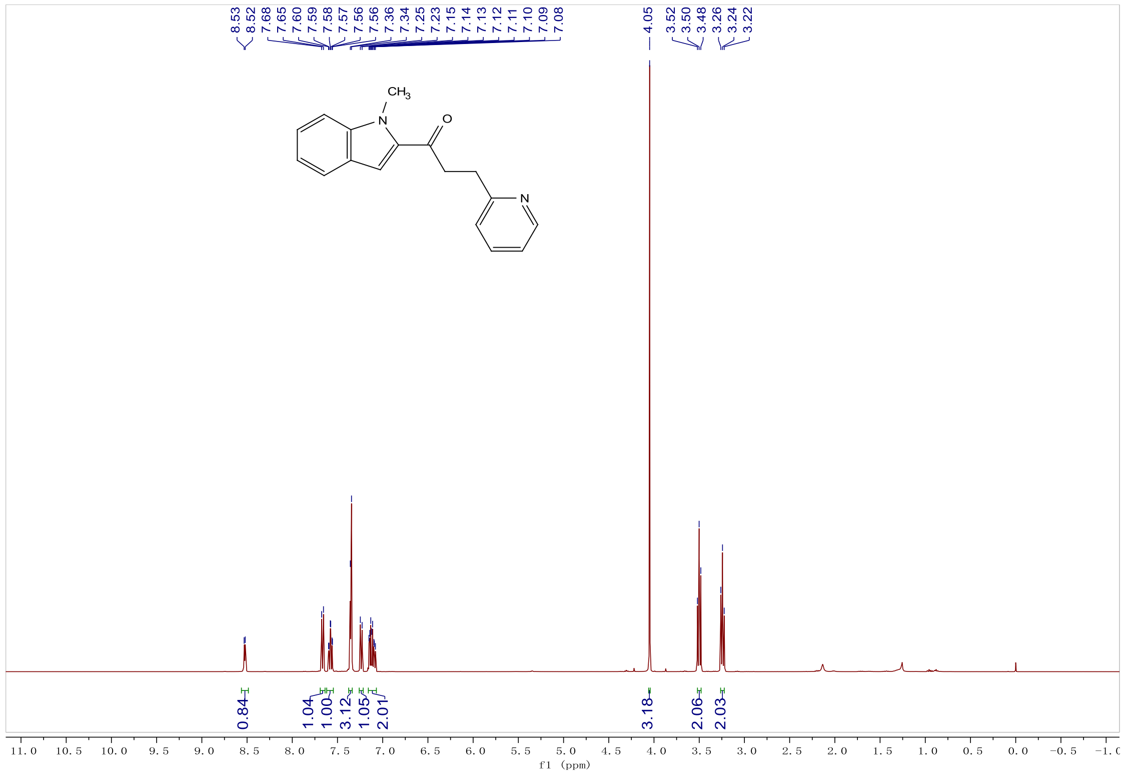

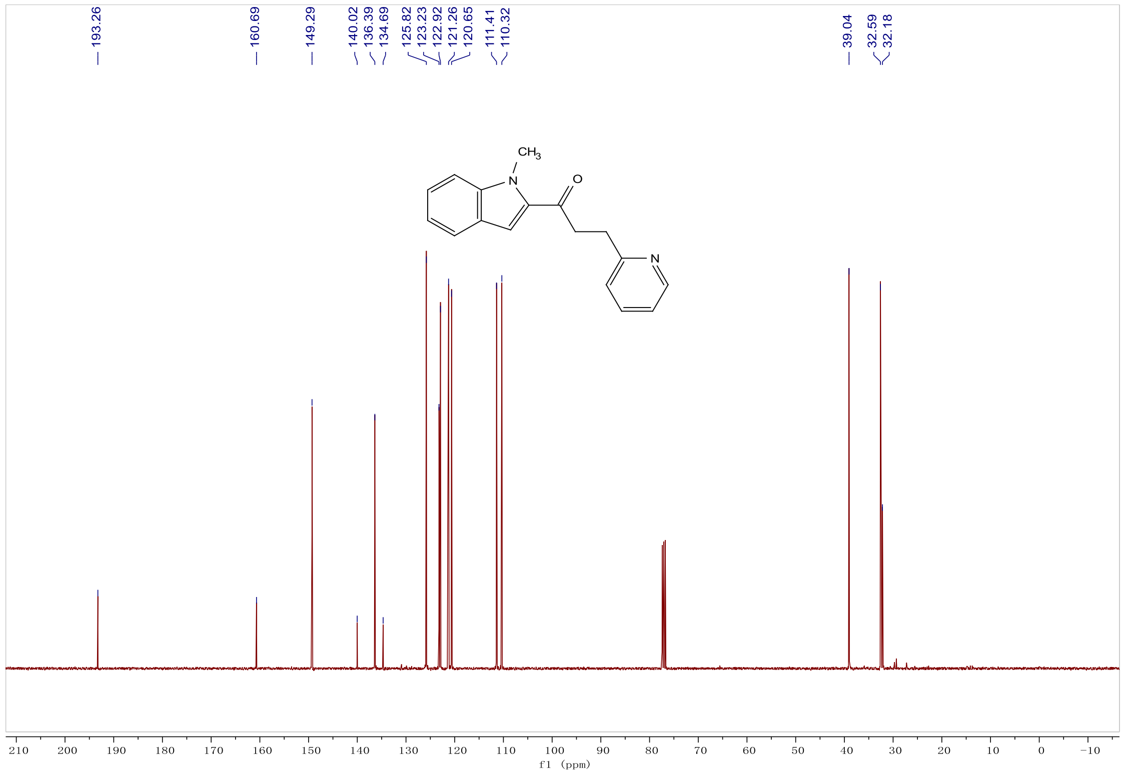

Supplementary Figure 38. ^{1}H and ^{13} C NMR spectra for compound **3x**

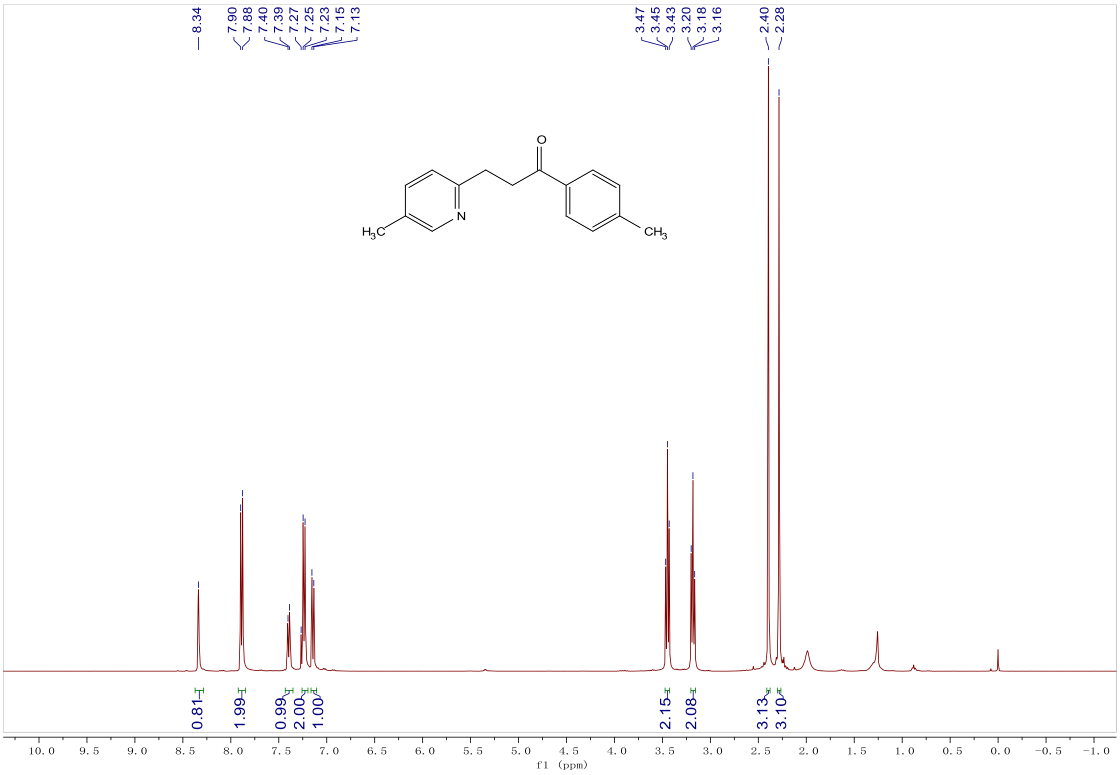

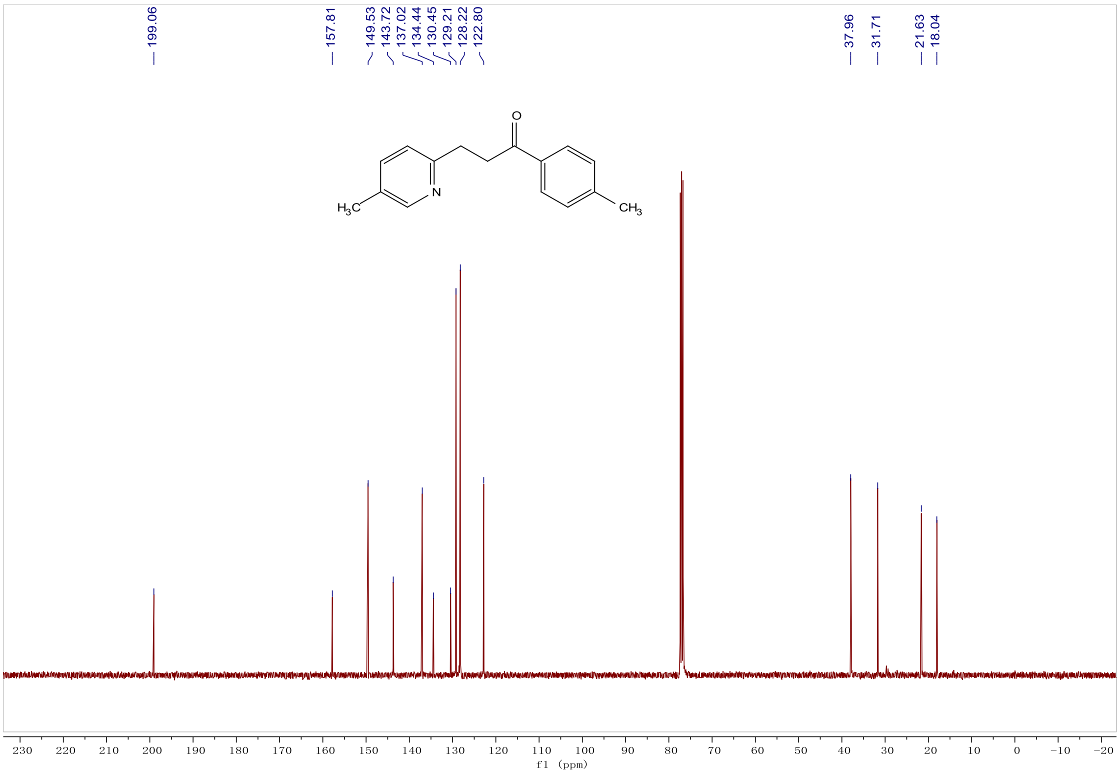

Supplementary Figure 39. ^{1}H and ^{13} C NMR spectra for compound **3y**

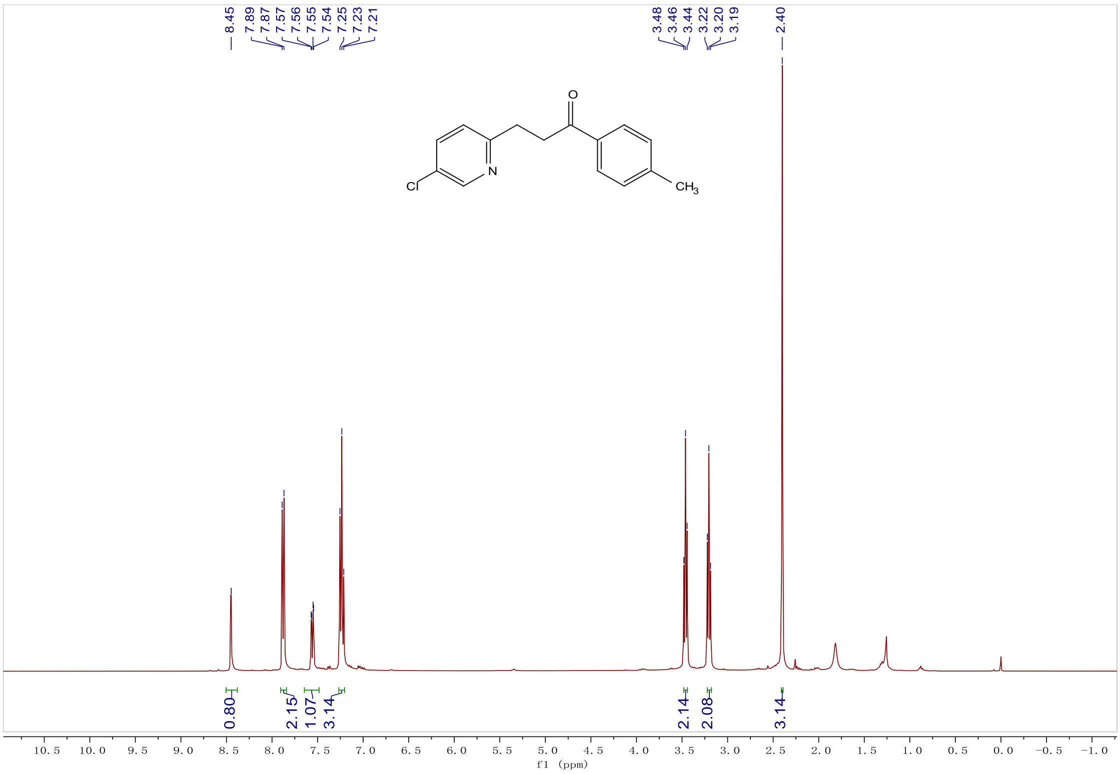

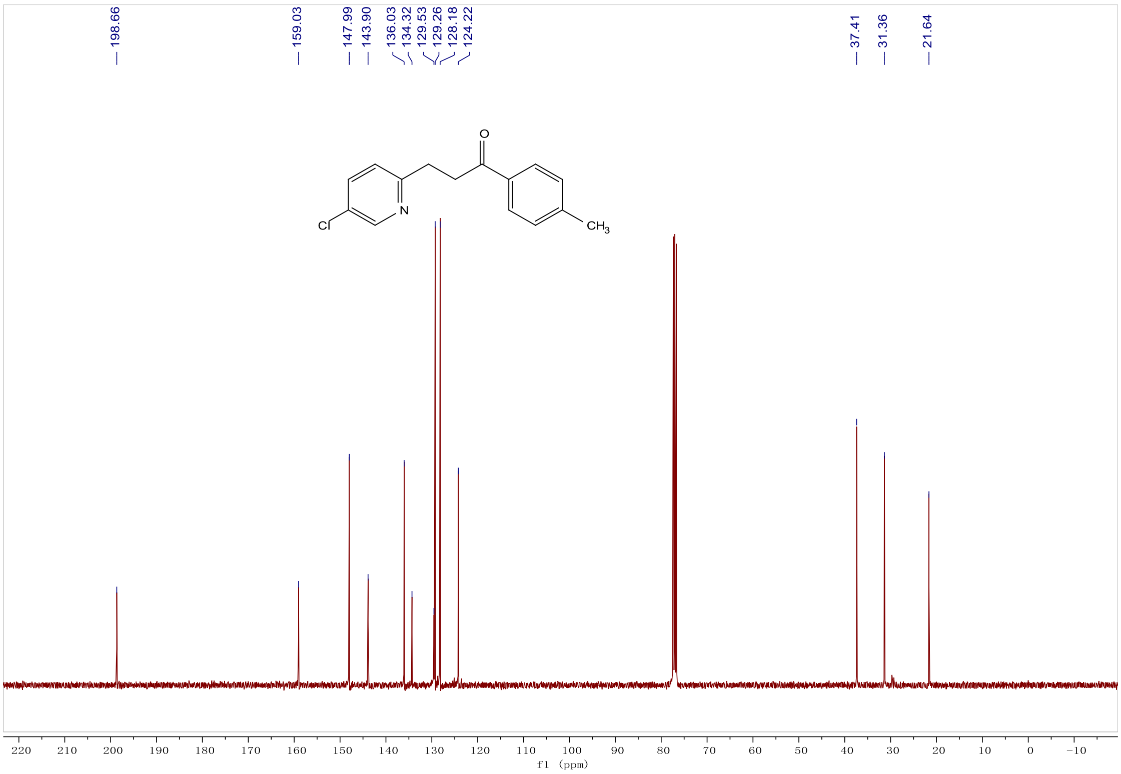

Supplementary Figure 40. ^{1}H and ^{13} C NMR spectra for compound **3z**

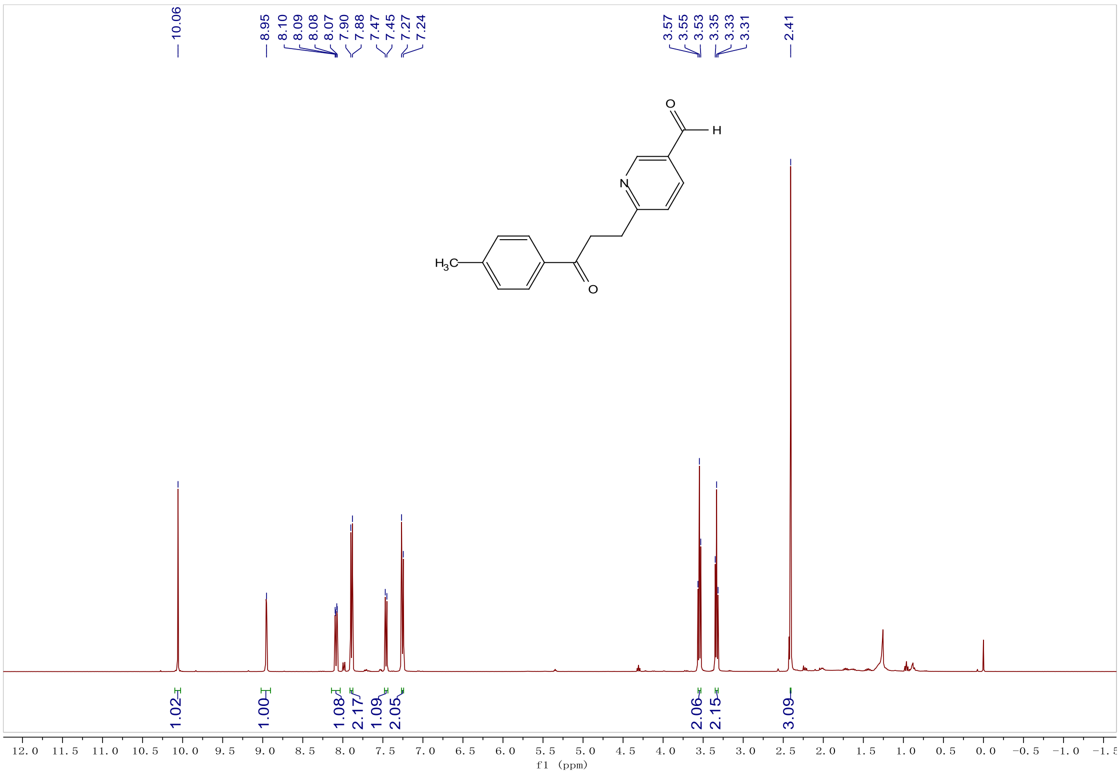

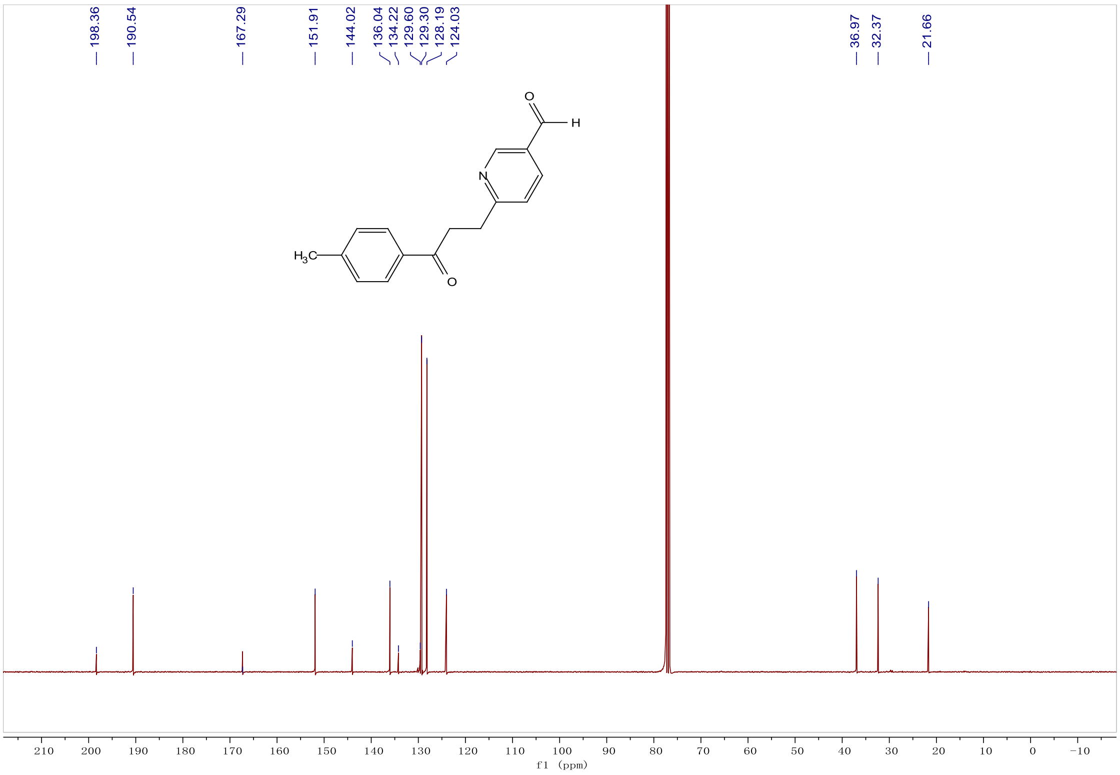

Supplementary Figure 41. ^{1}H and ^{13} C NMR spectra for compound **3aa**

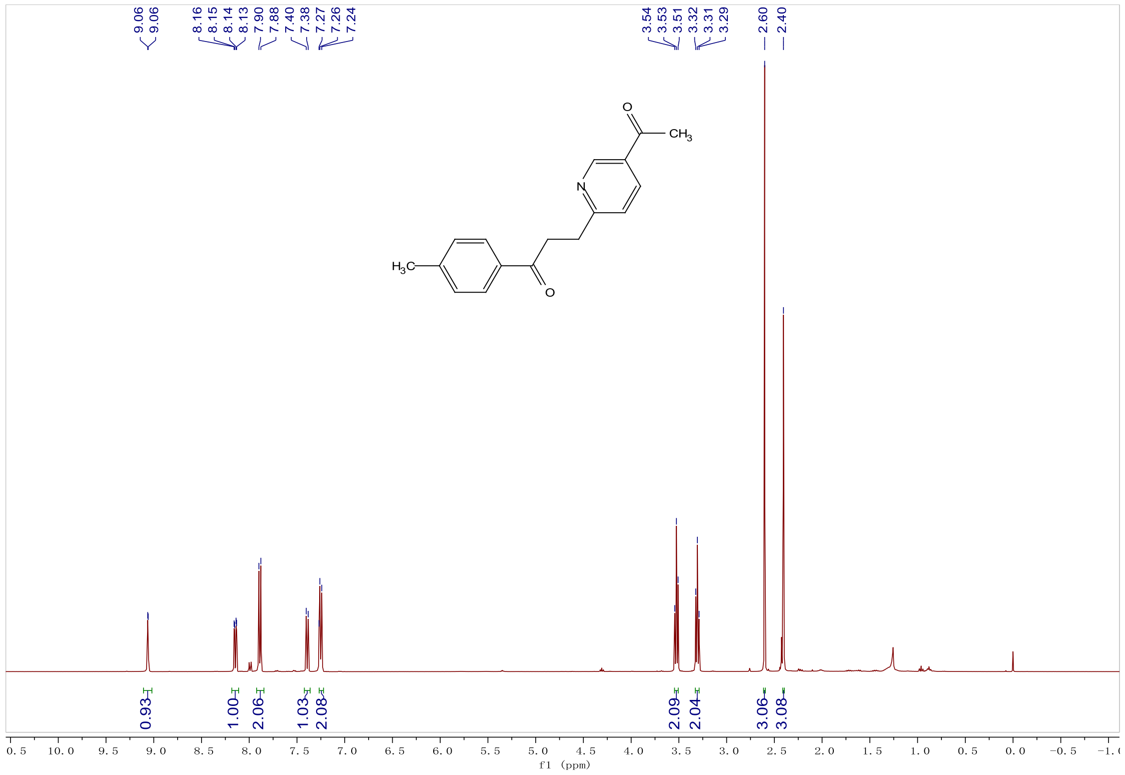

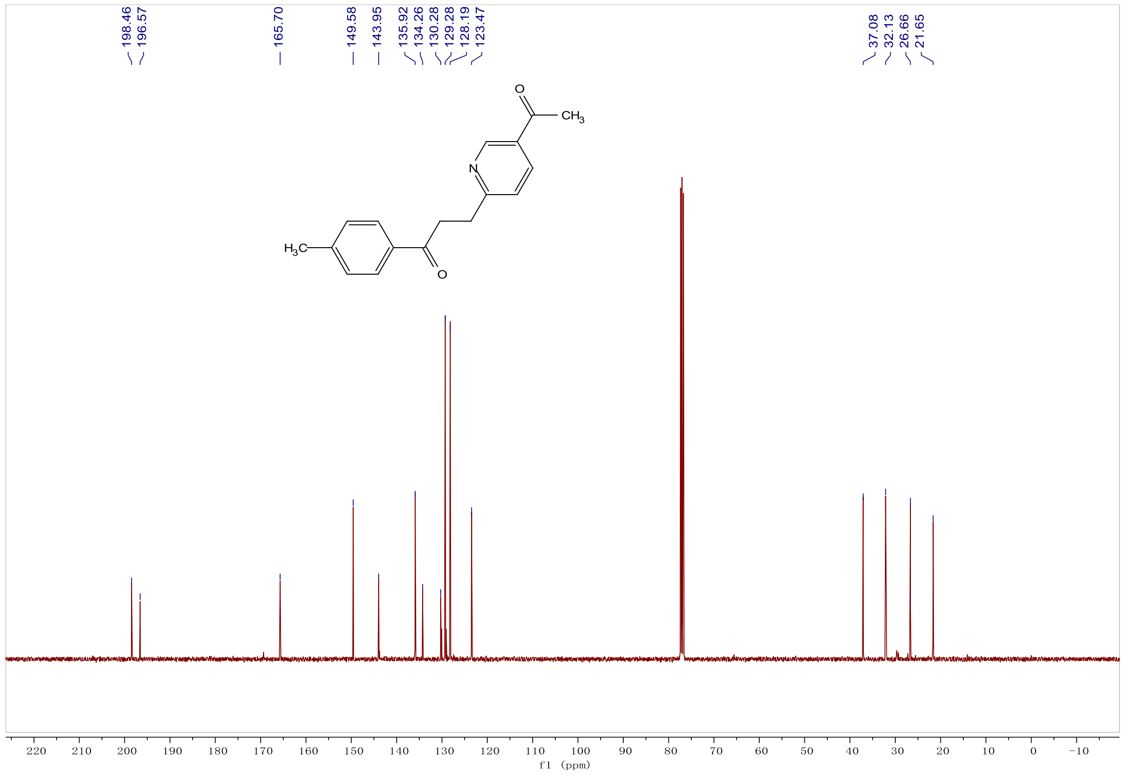

Supplementary Figure 42. ^{1}H and ^{13}C NMR spectra for compound **3bb**

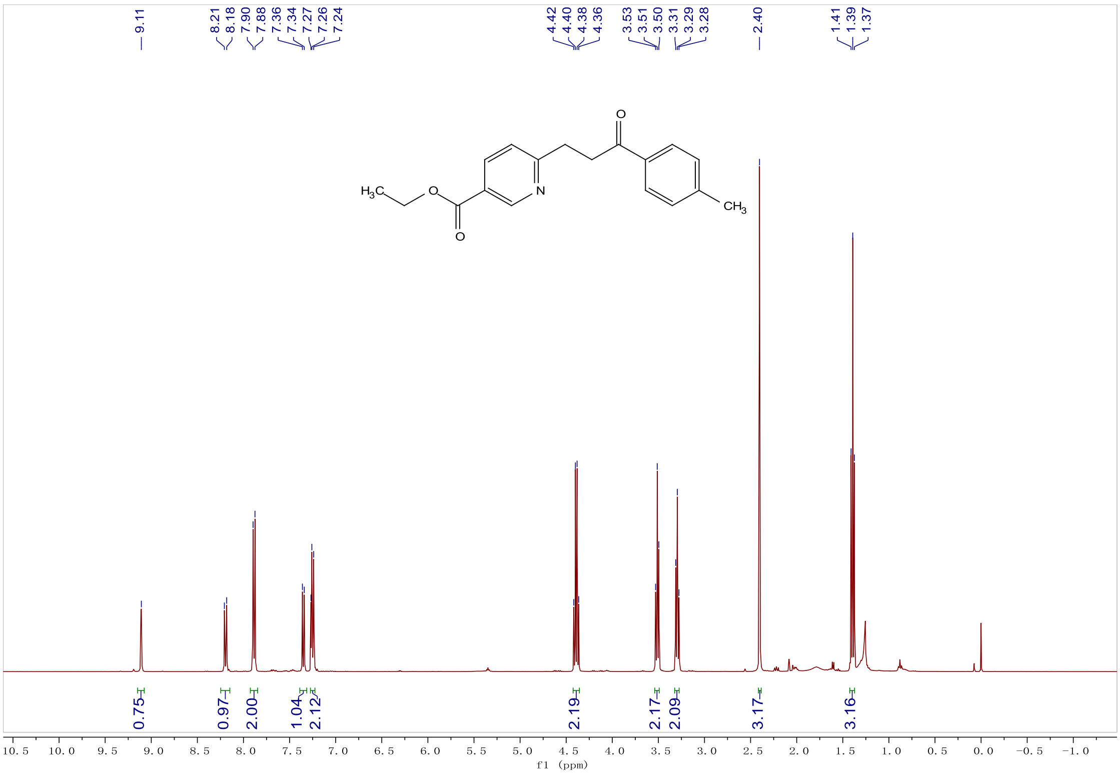

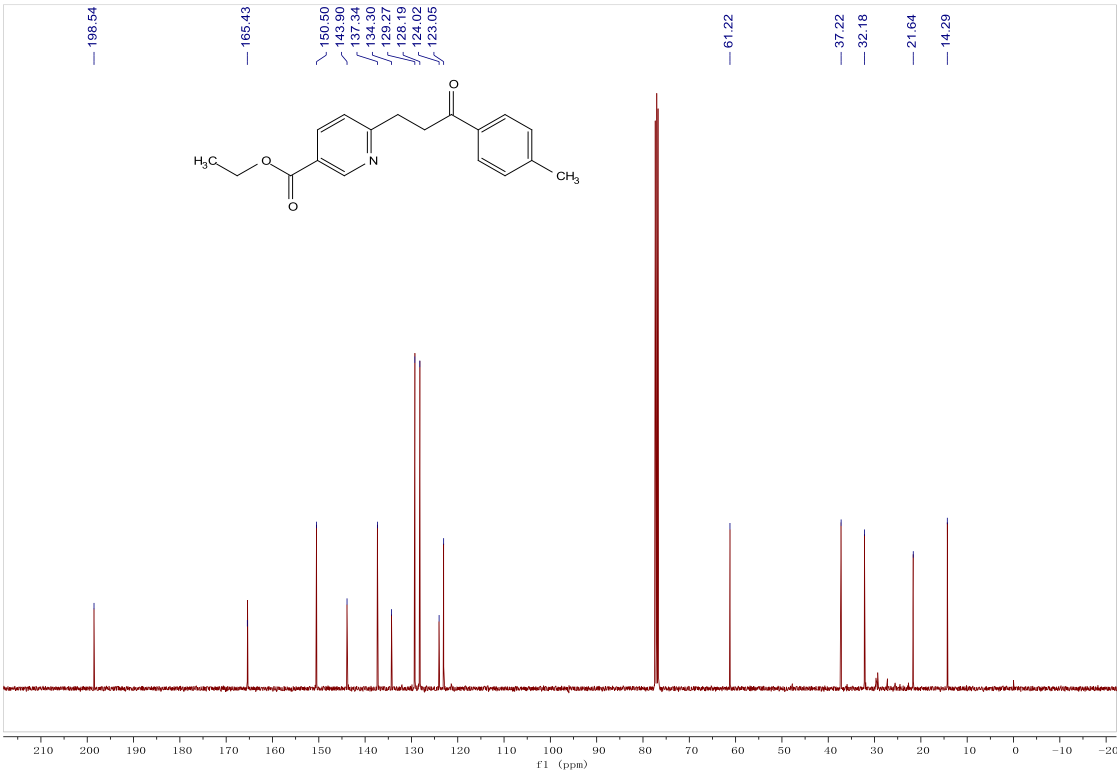

Supplementary Figure 43. ^{1}H and ^{13}C NMR spectra for compound **3cc**

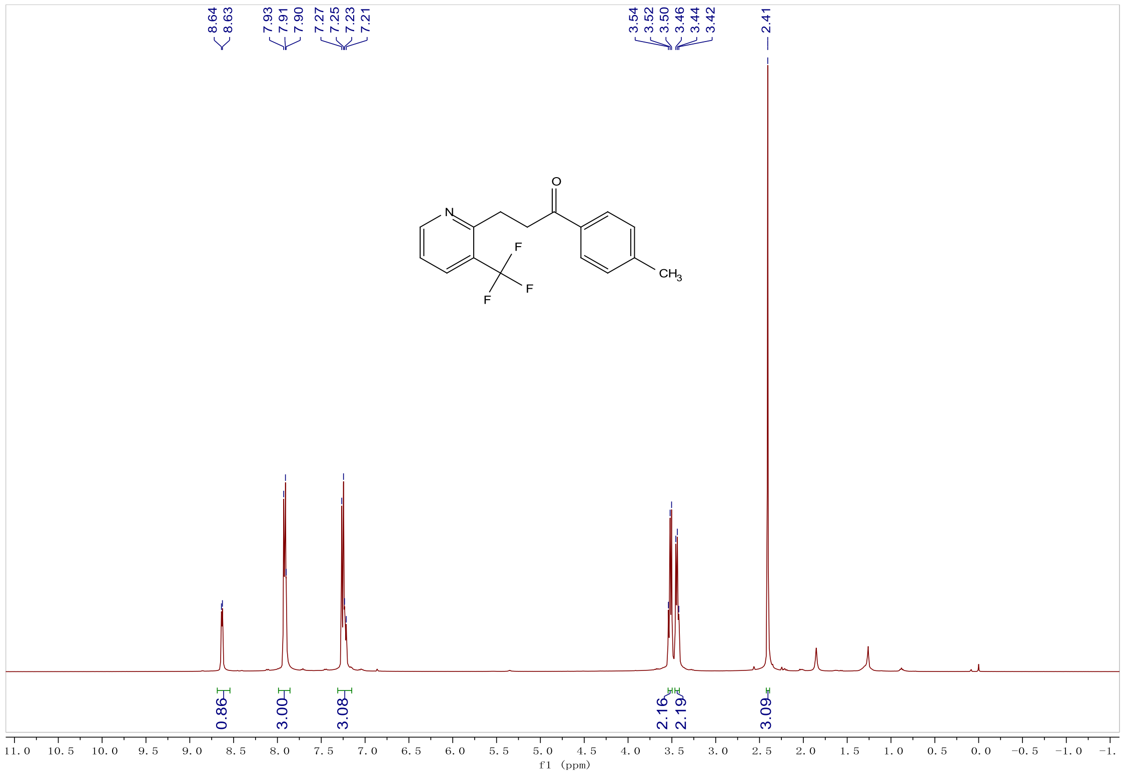

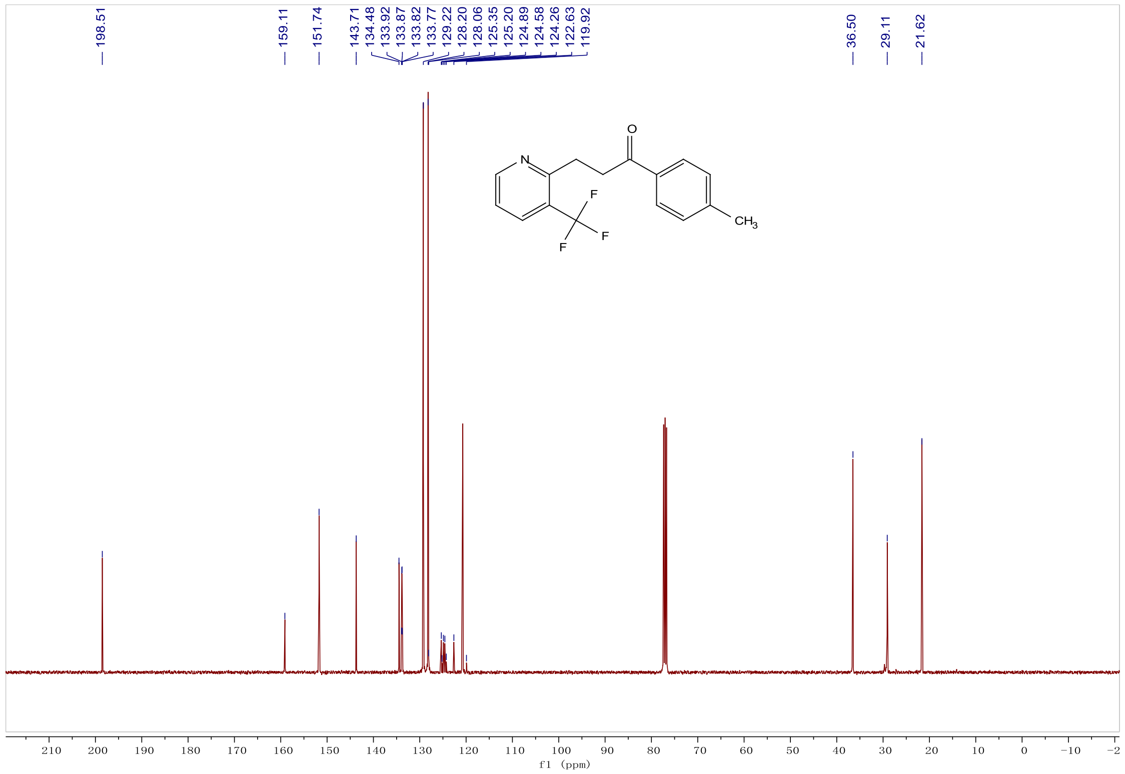

Supplementary Figure 44. ^{1}H and ^{13} C NMR spectra for compound **3dd**

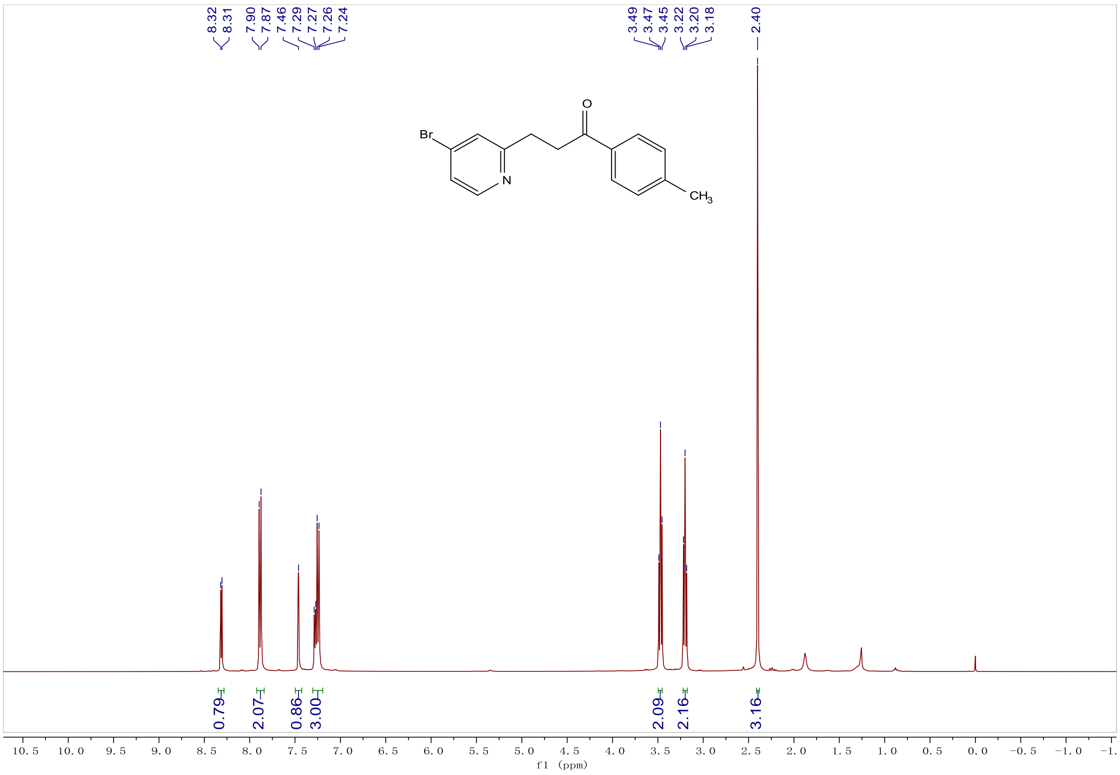

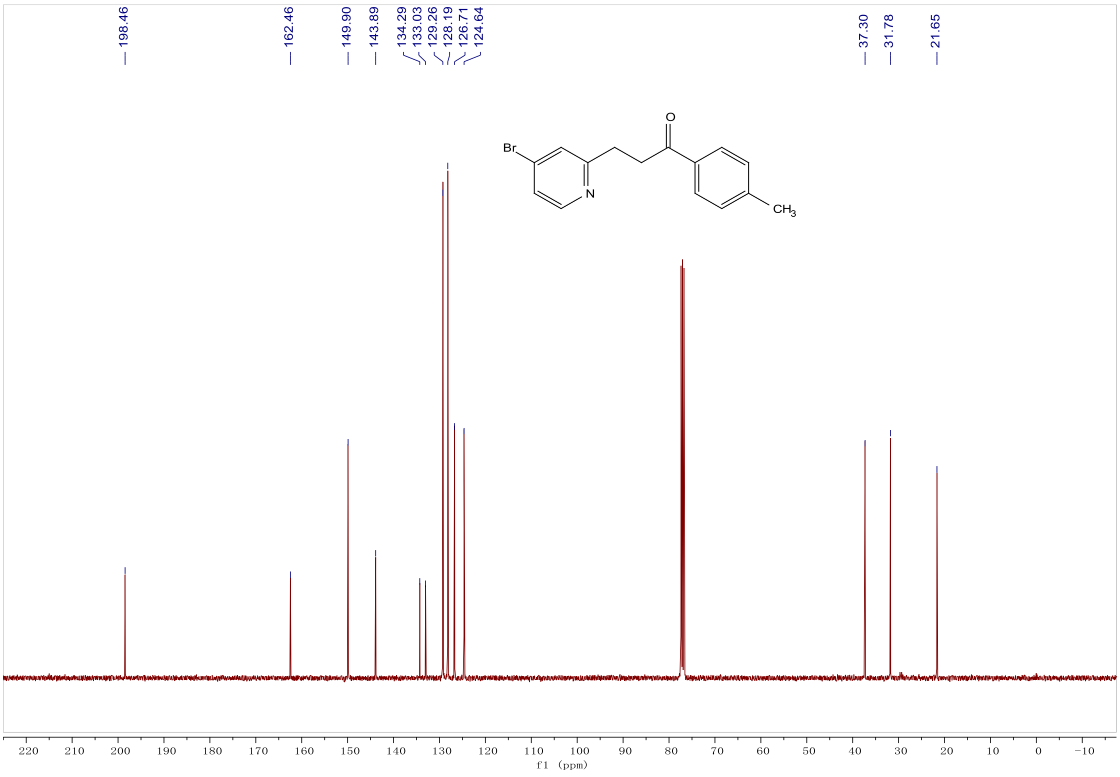

Supplementary Figure 45. ^{1}H and ^{13}C NMR spectra for compound **3ee**

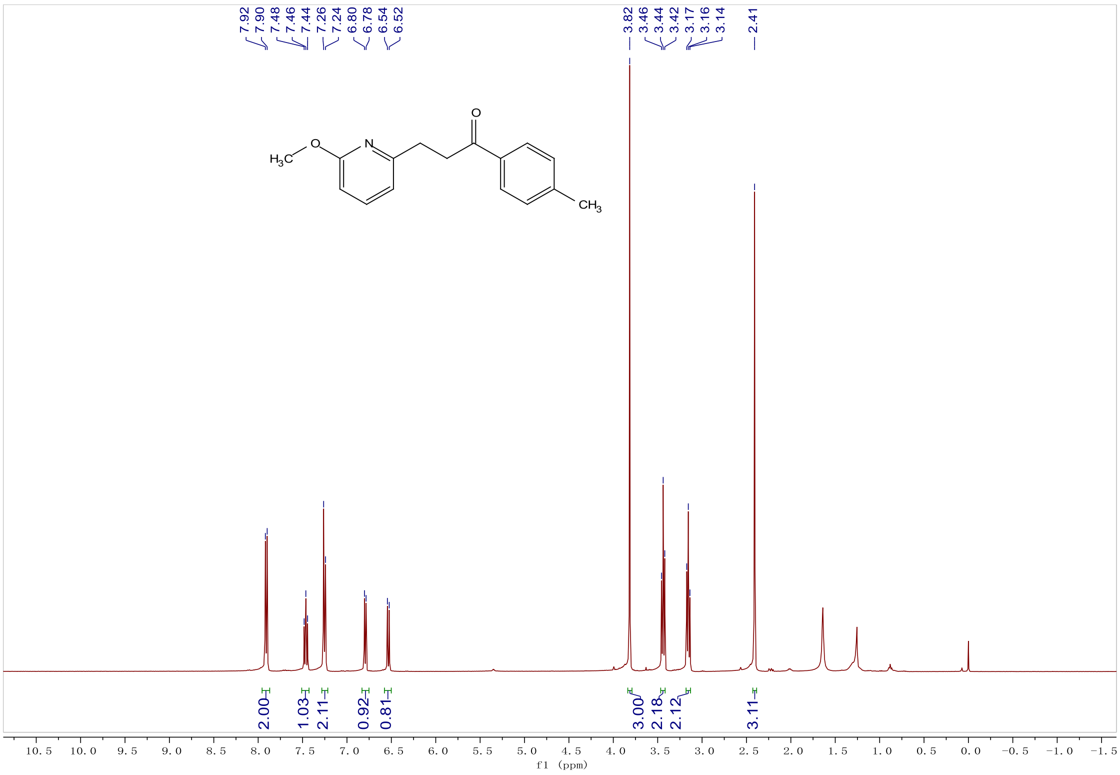

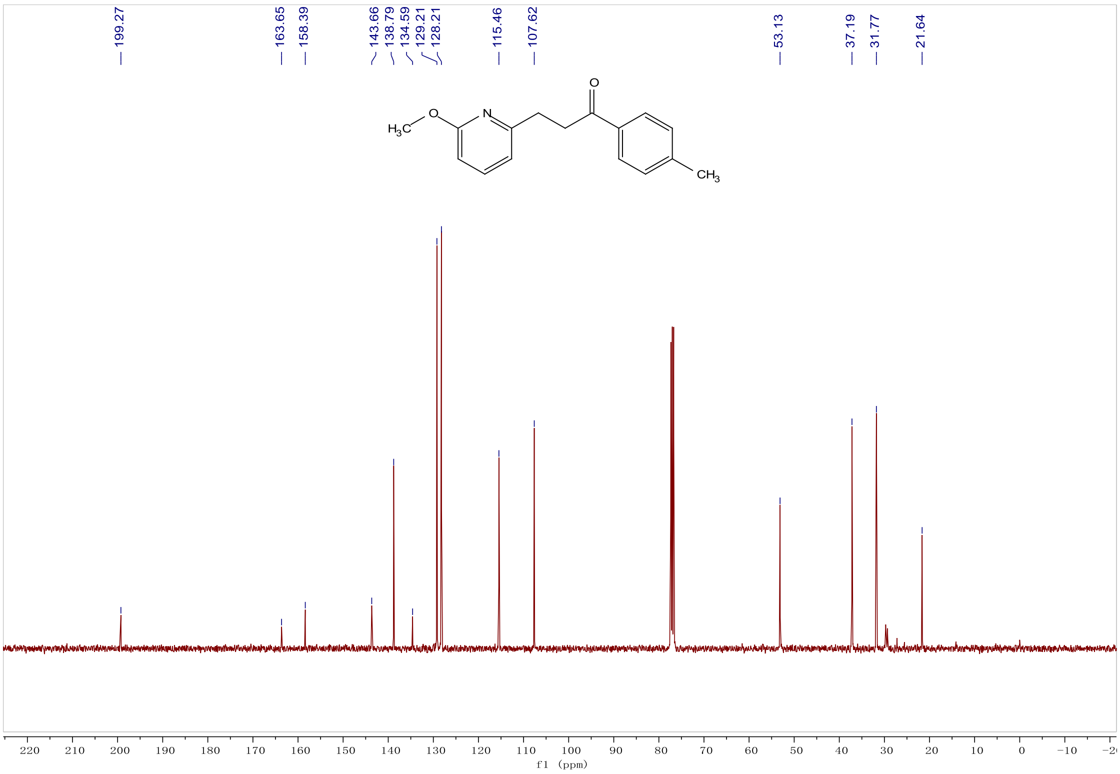

Supplementary Figure 46. ^{1}H and ^{13} C NMR spectra for compound **3ff**

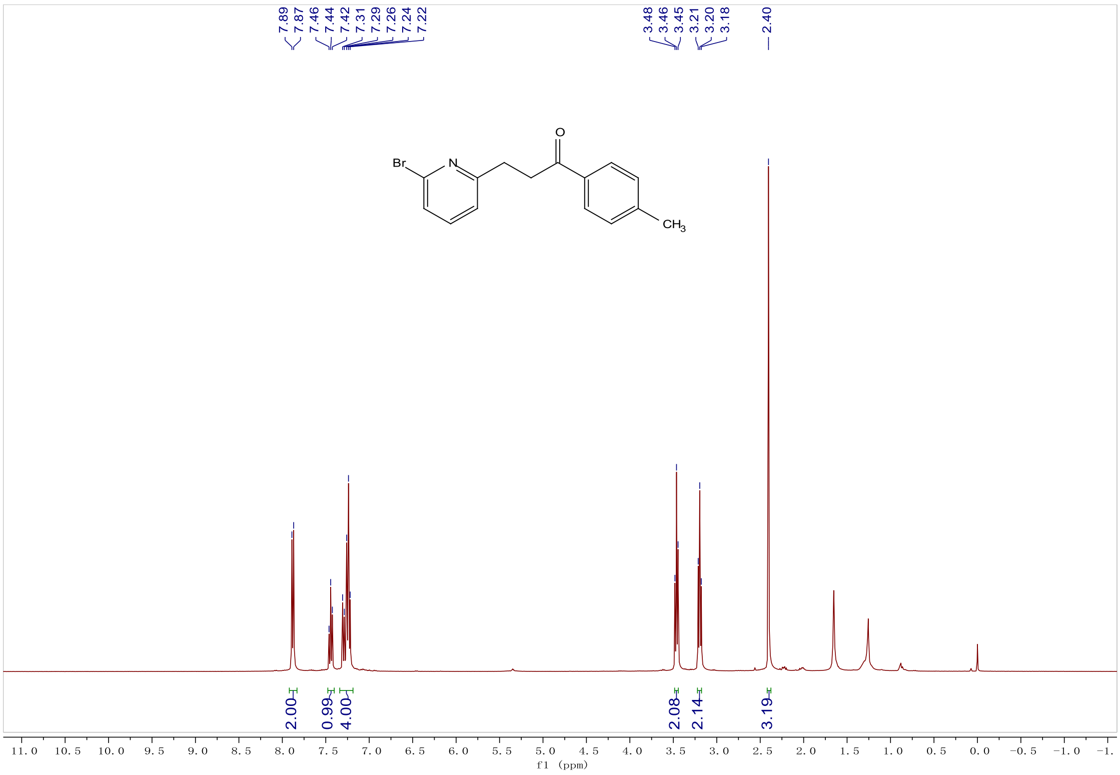

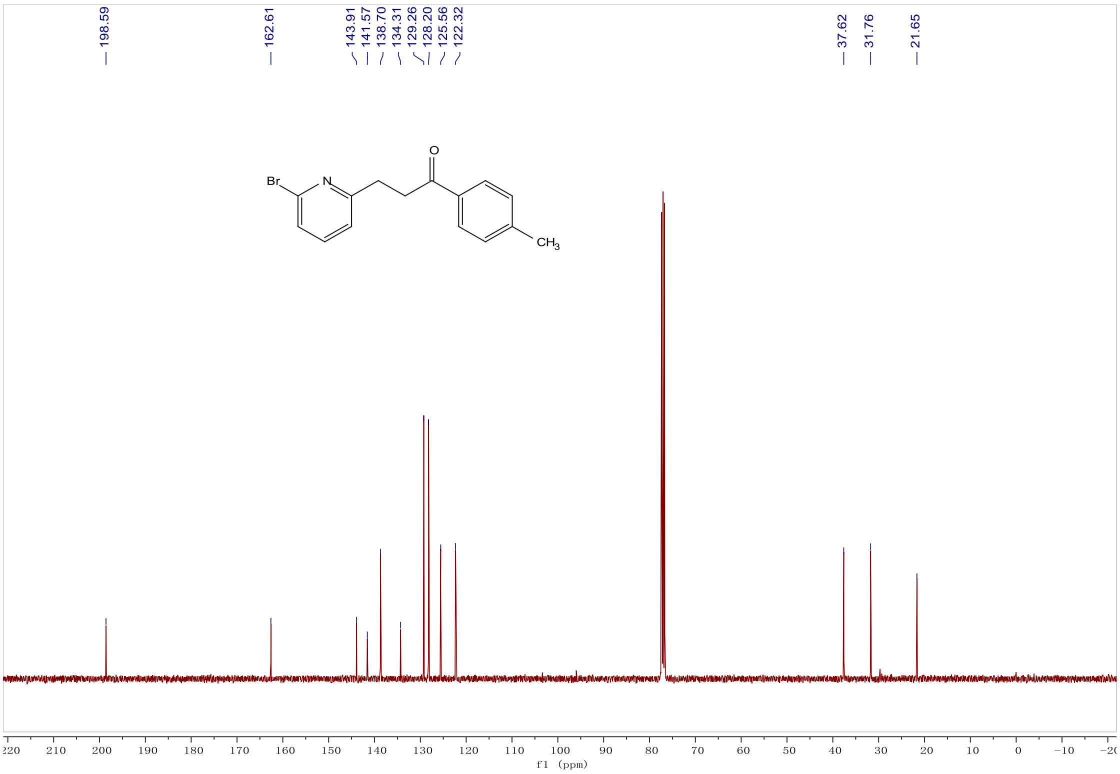

Supplementary Figure 47. ^{1}H and ^{13}C NMR spectra for compound **3gg**

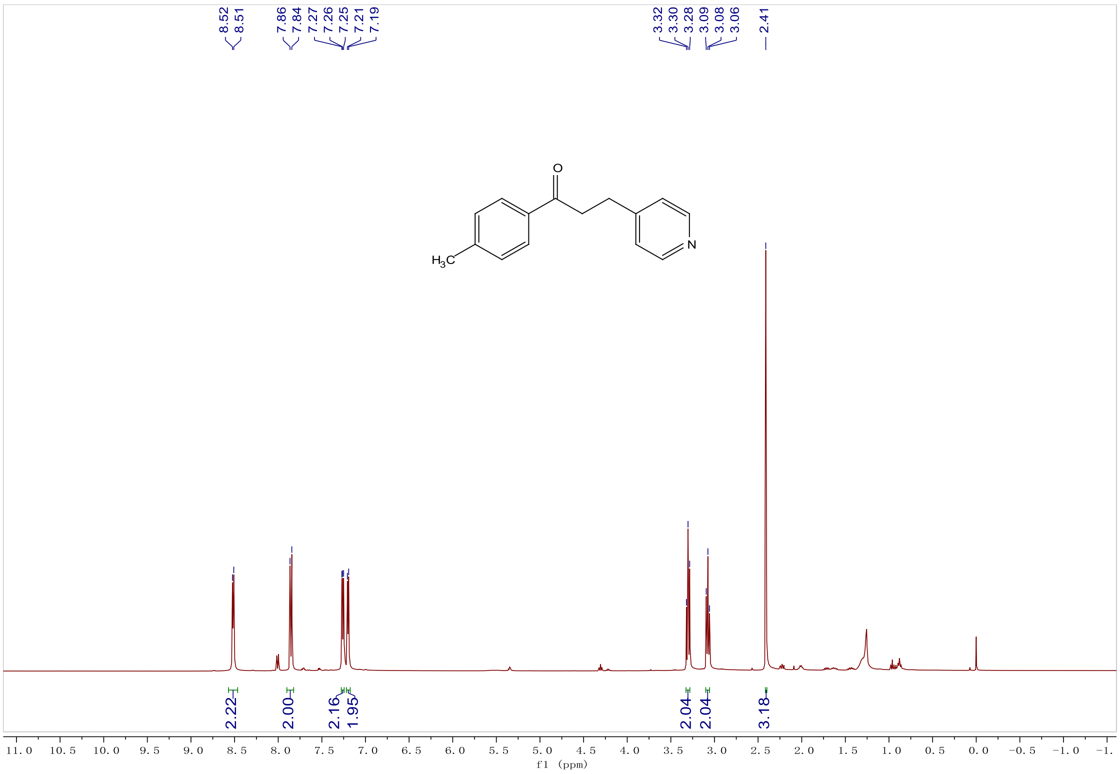

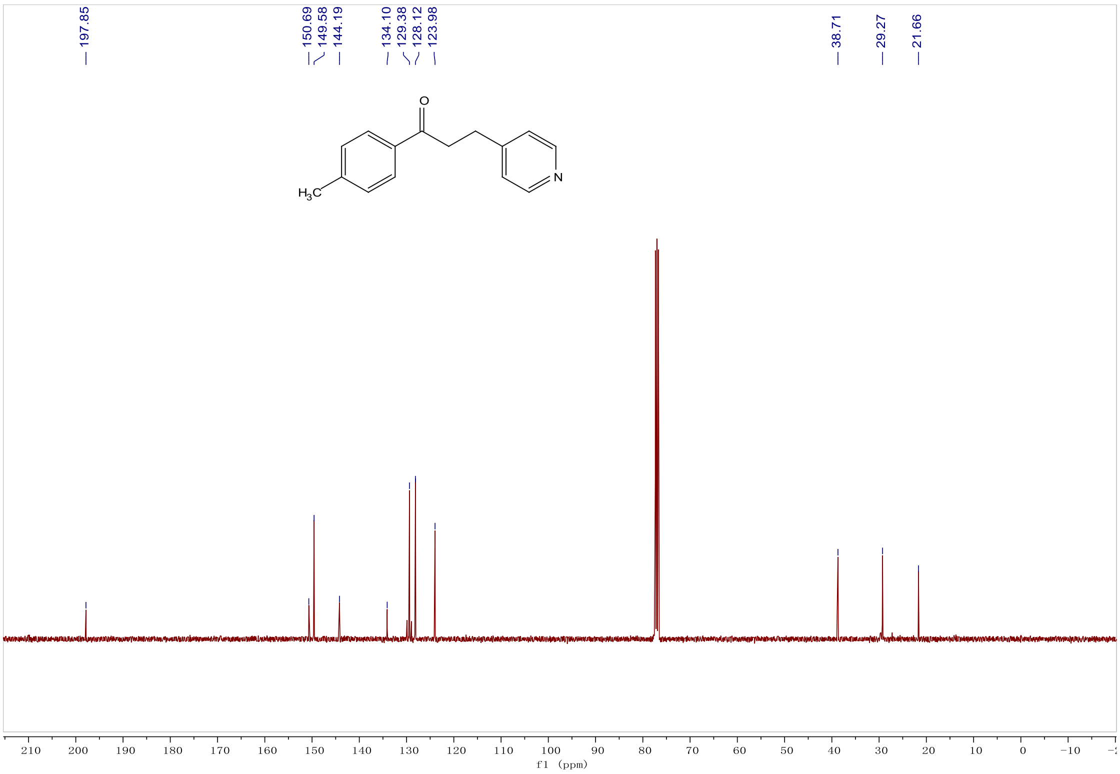

Supplementary Figure 48. ^{1}H and ^{13}C NMR spectra for compound **3hh**

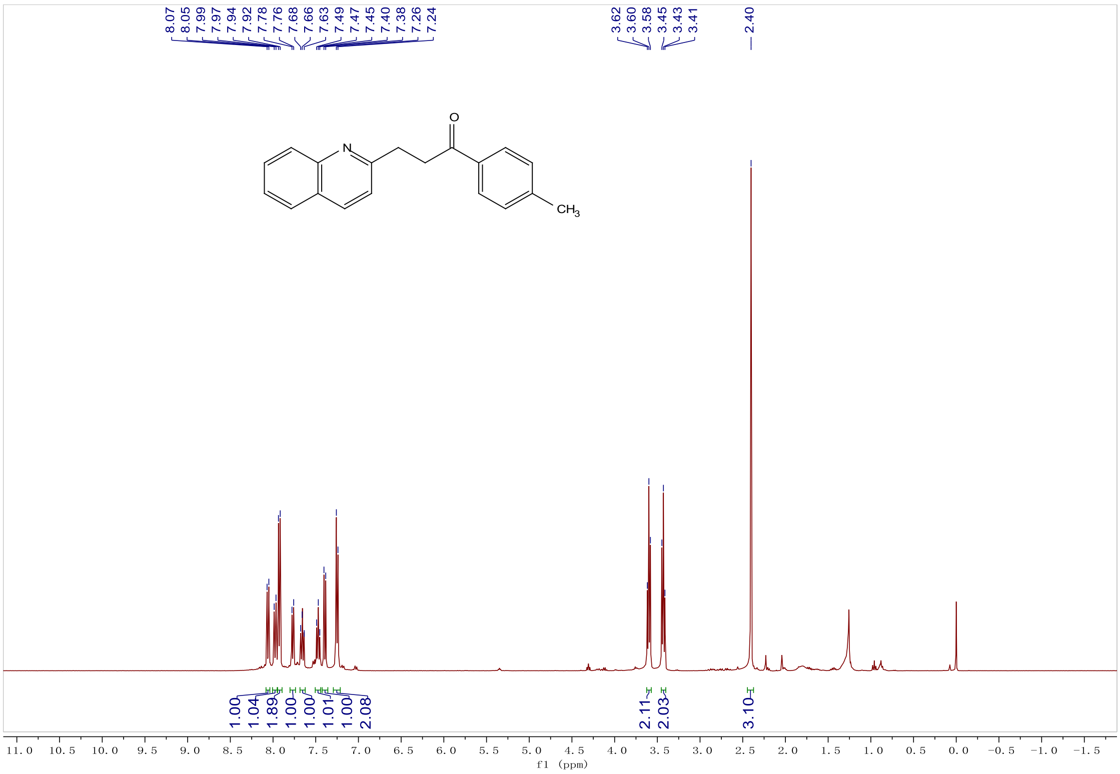

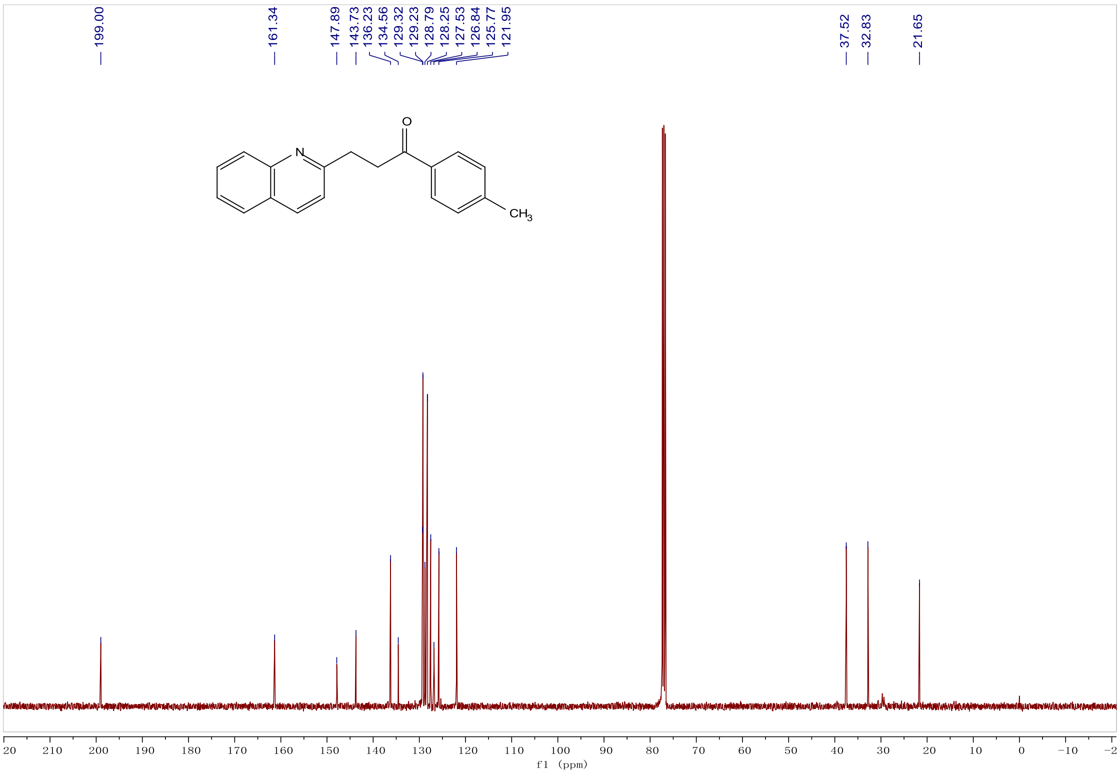

Supplementary Figure 49. ^{1}H and ^{13} C NMR spectra for compound **3ii**

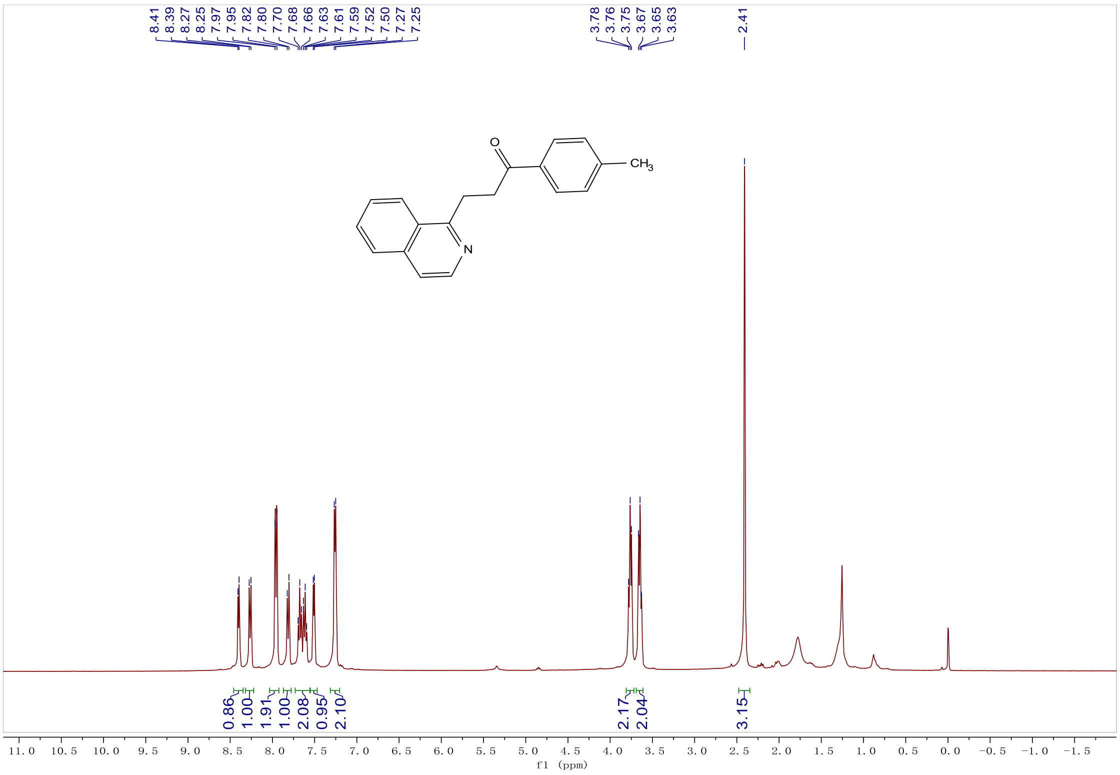

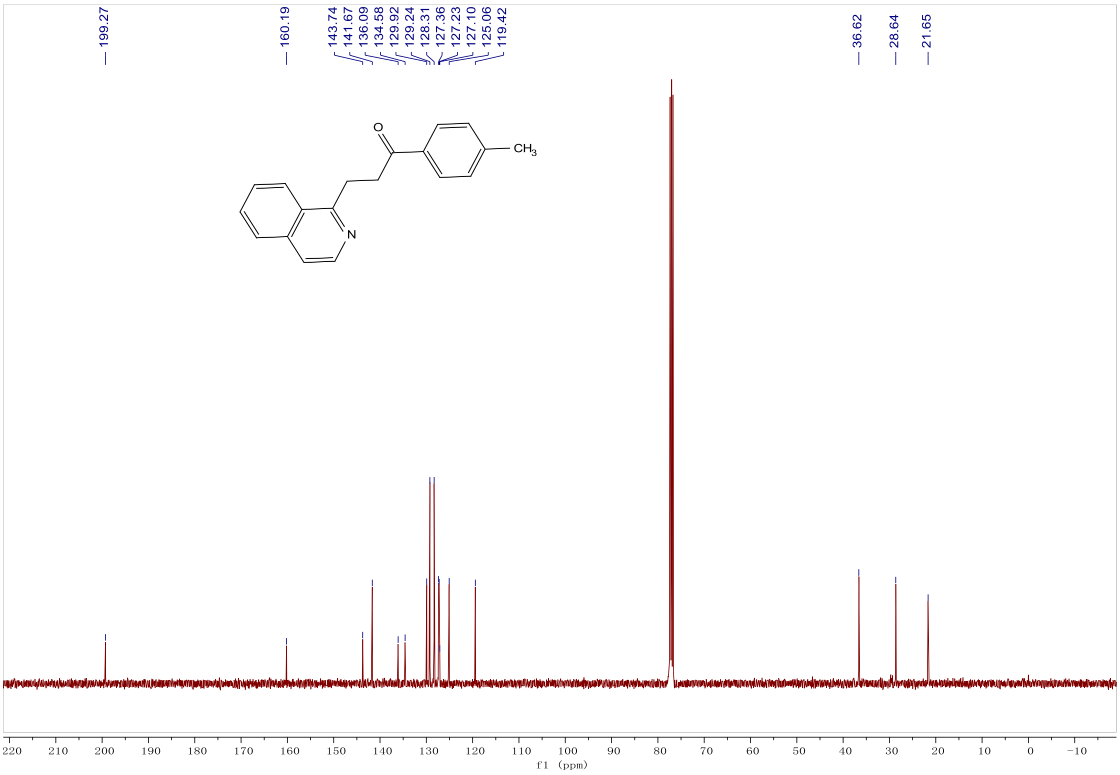

Supplementary Figure 50. ^{1}H and ^{13} C NMR spectra for compound **3jj**

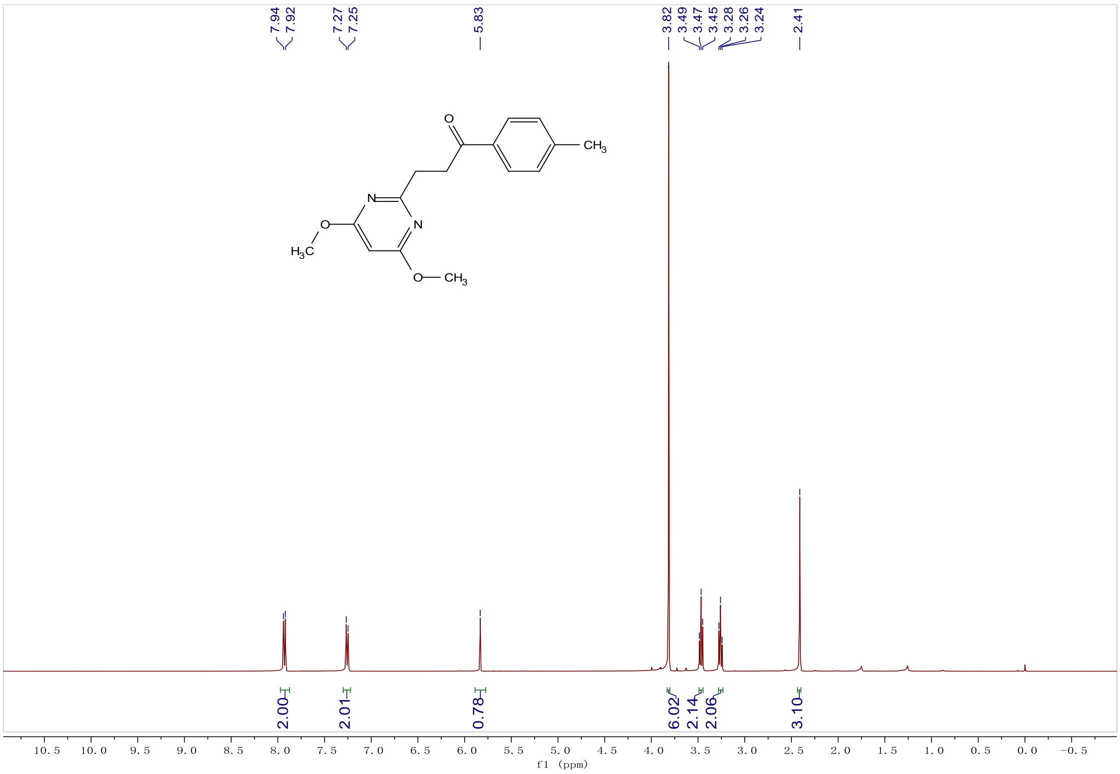

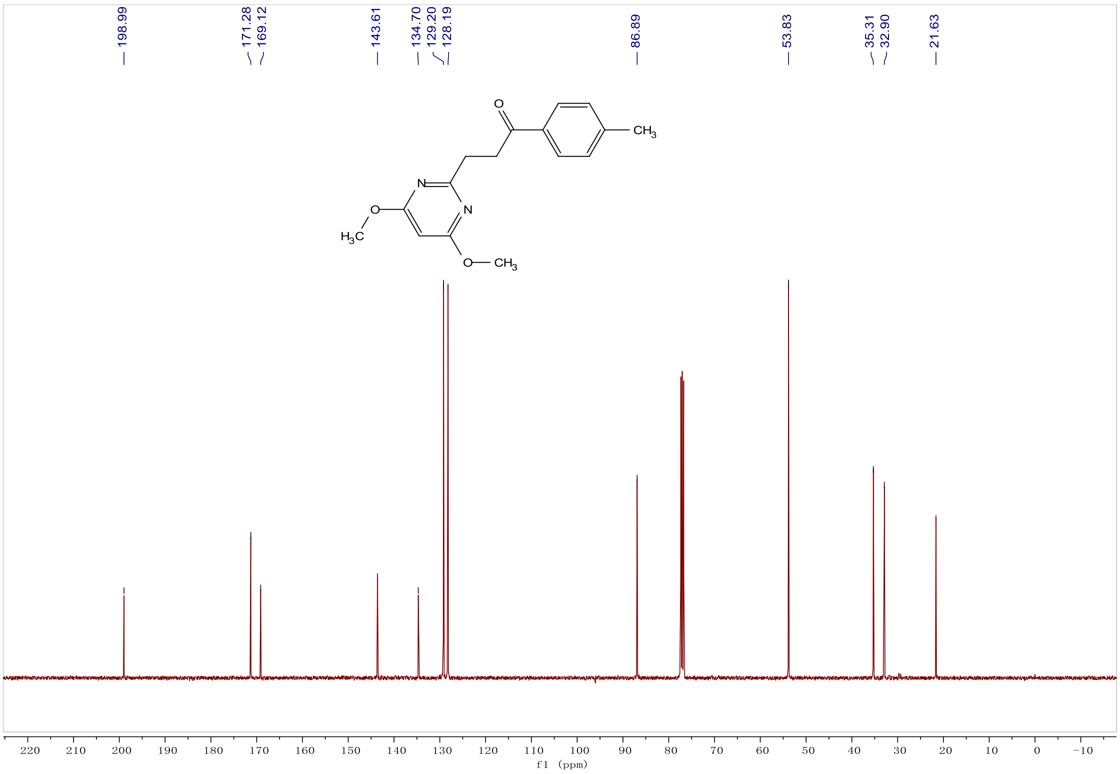

Supplementary Figure 51. ^{1}H and ^{13} C NMR spectra for compound **3kk**

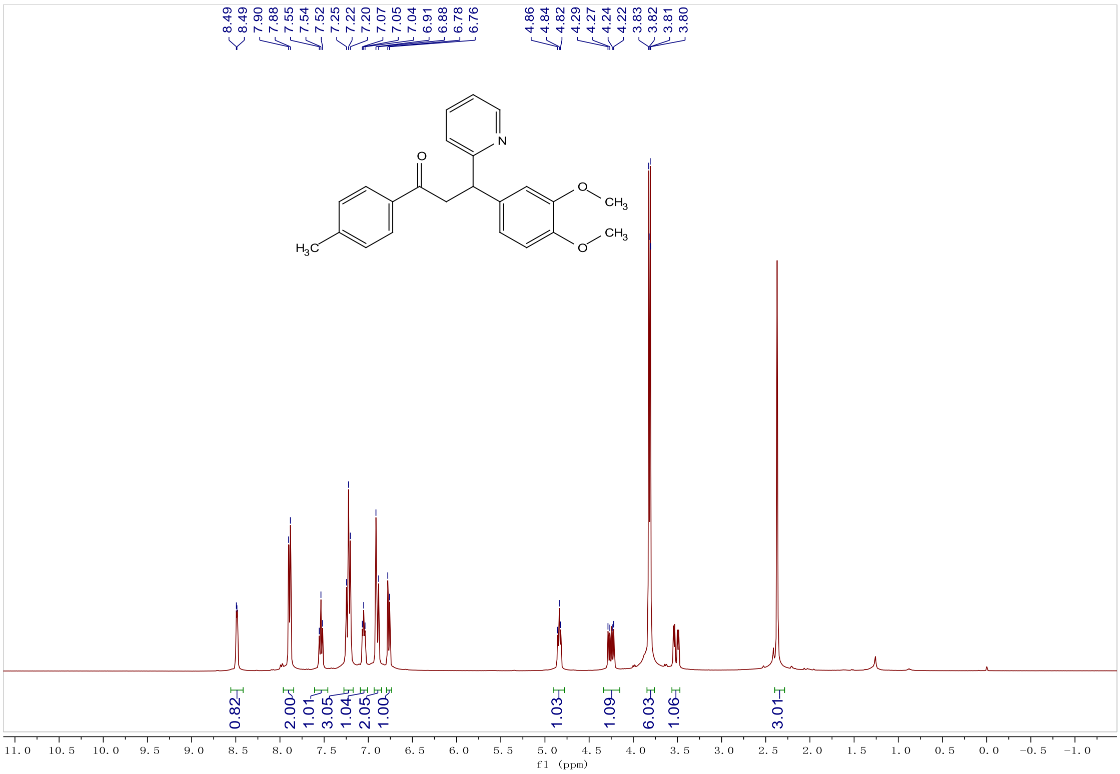

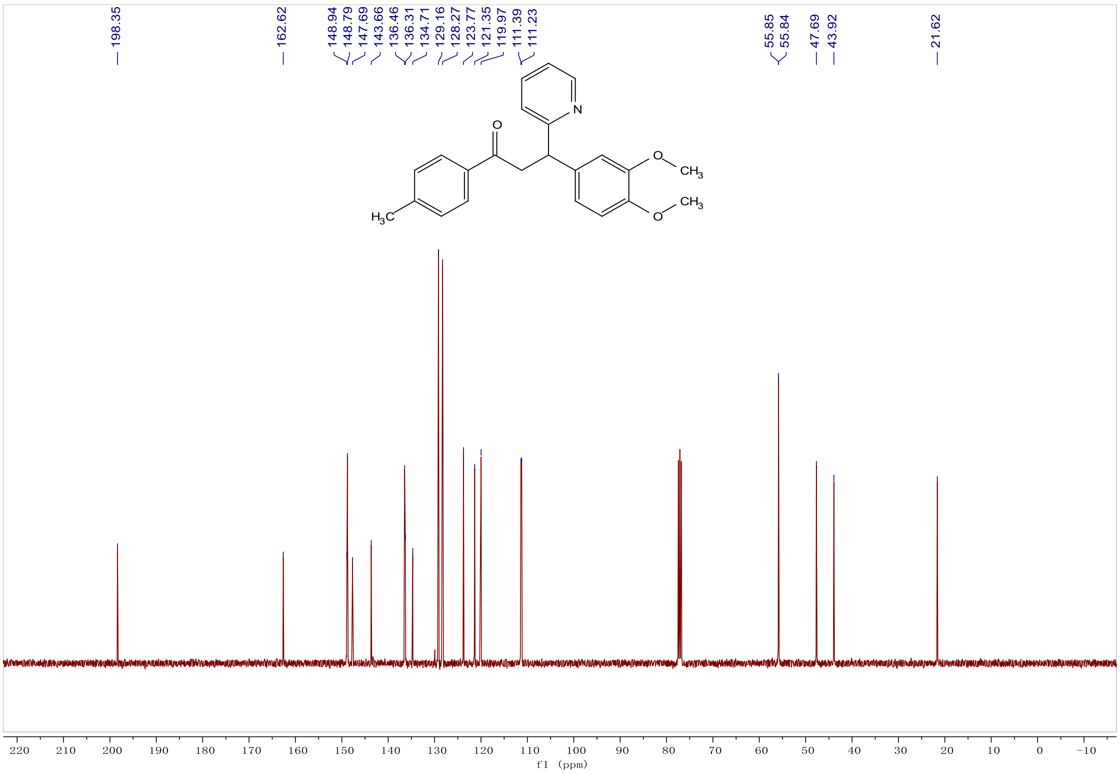

Supplementary Figure 52. ^{1}H and ^{13}C NMR spectra for compound **3ll**

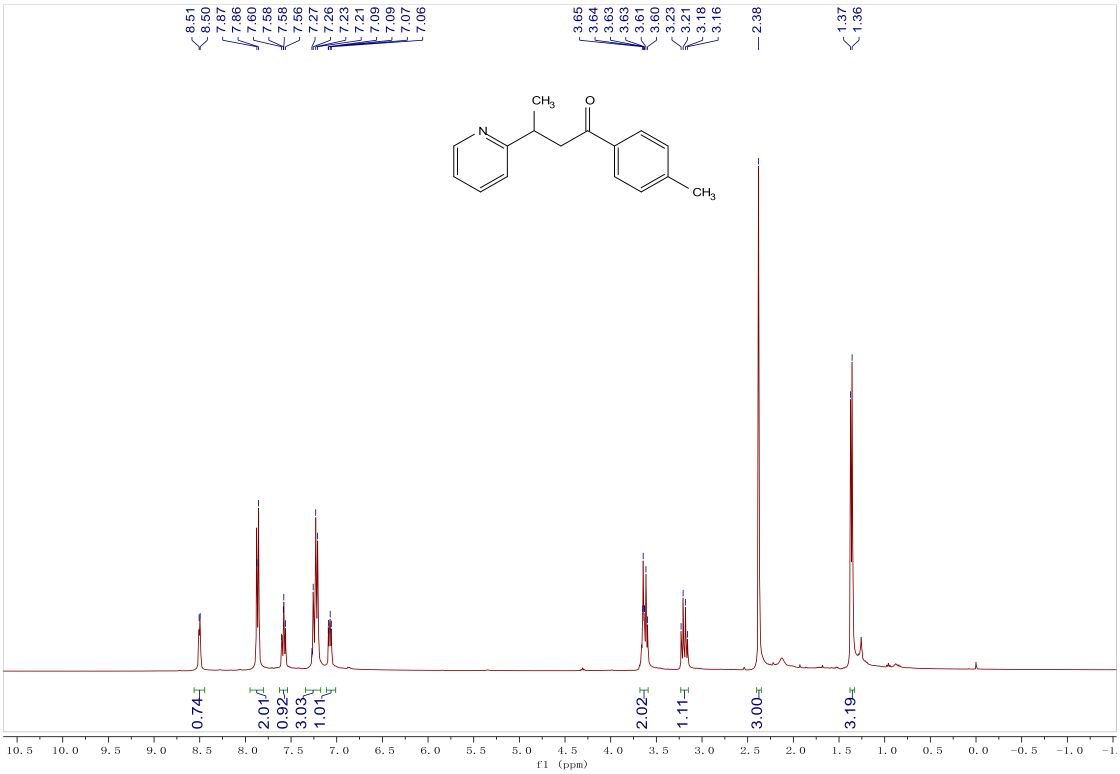

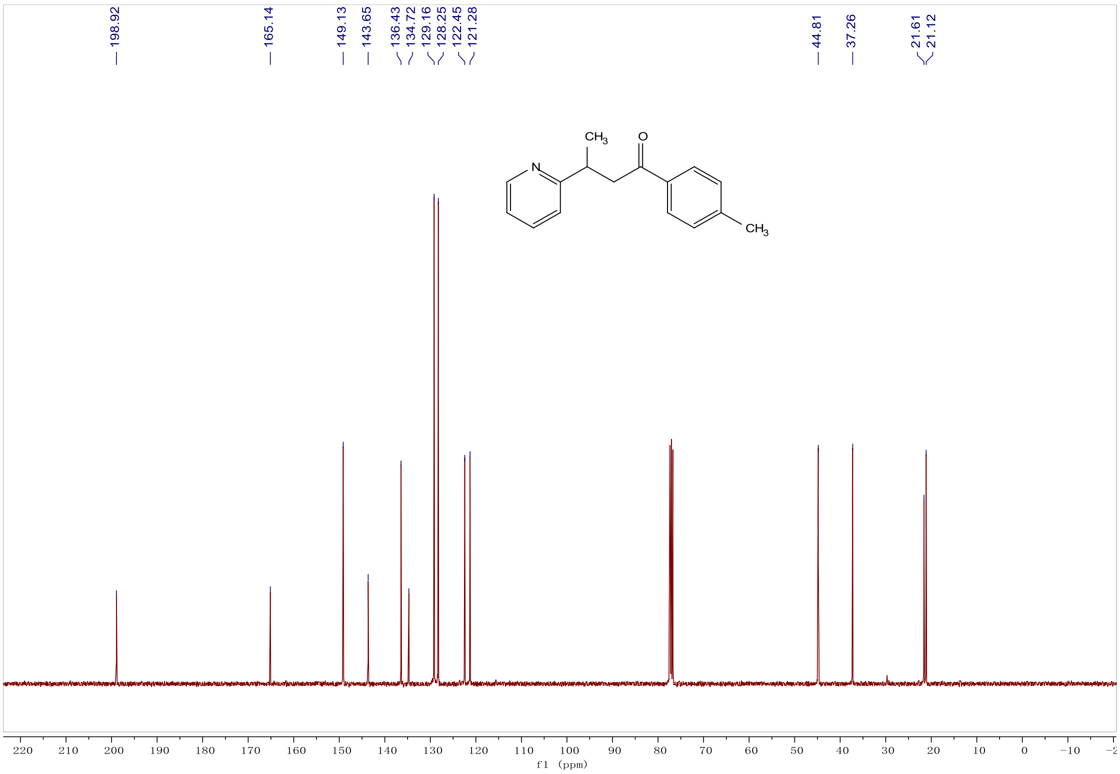

Supplementary Figure 53. ^{1}H and ^{13}C NMR spectra for compound **3mm**

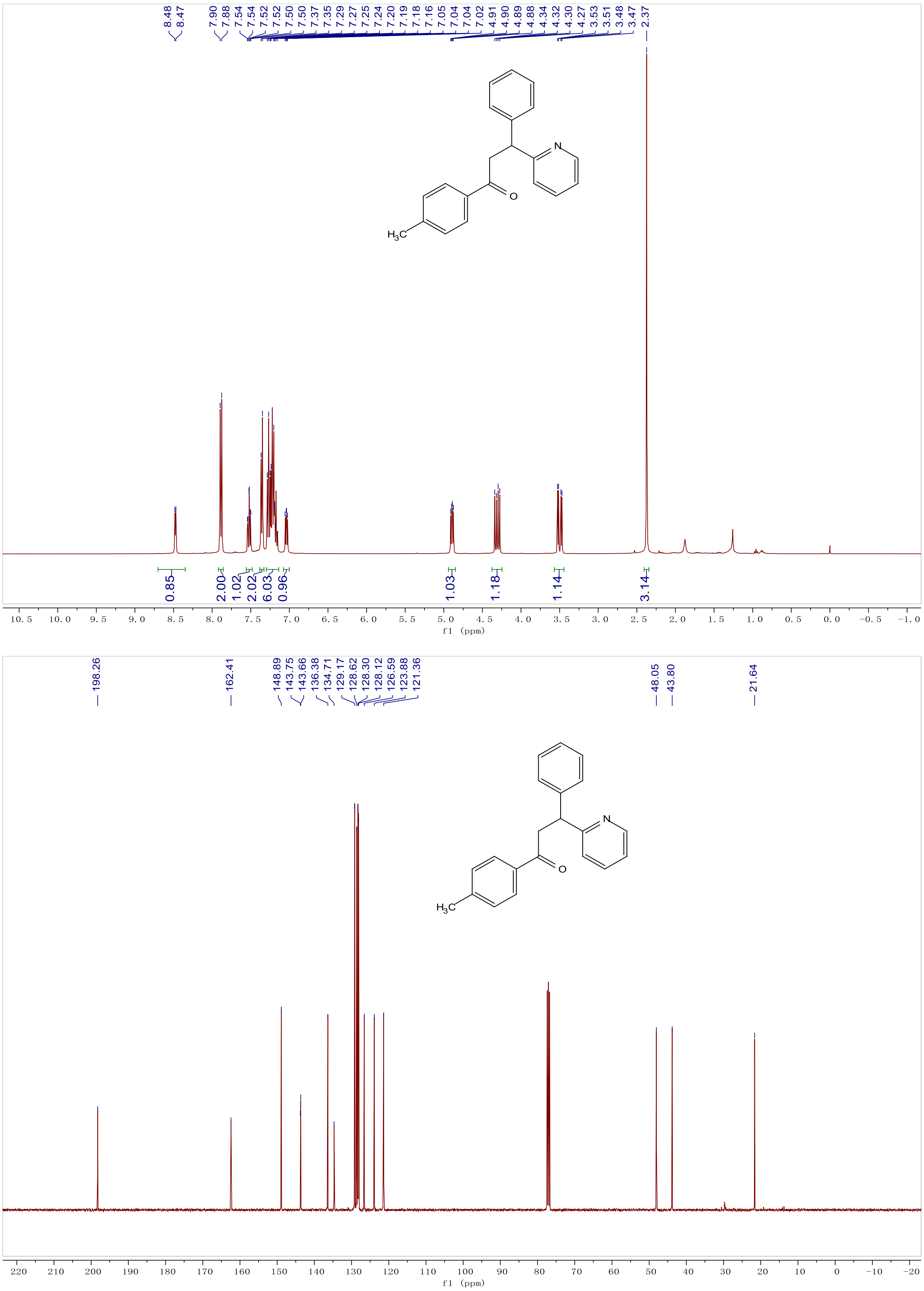

Supplementary Figure 54. ^{1}H and ^{13} C NMR spectra for compound **3nn**

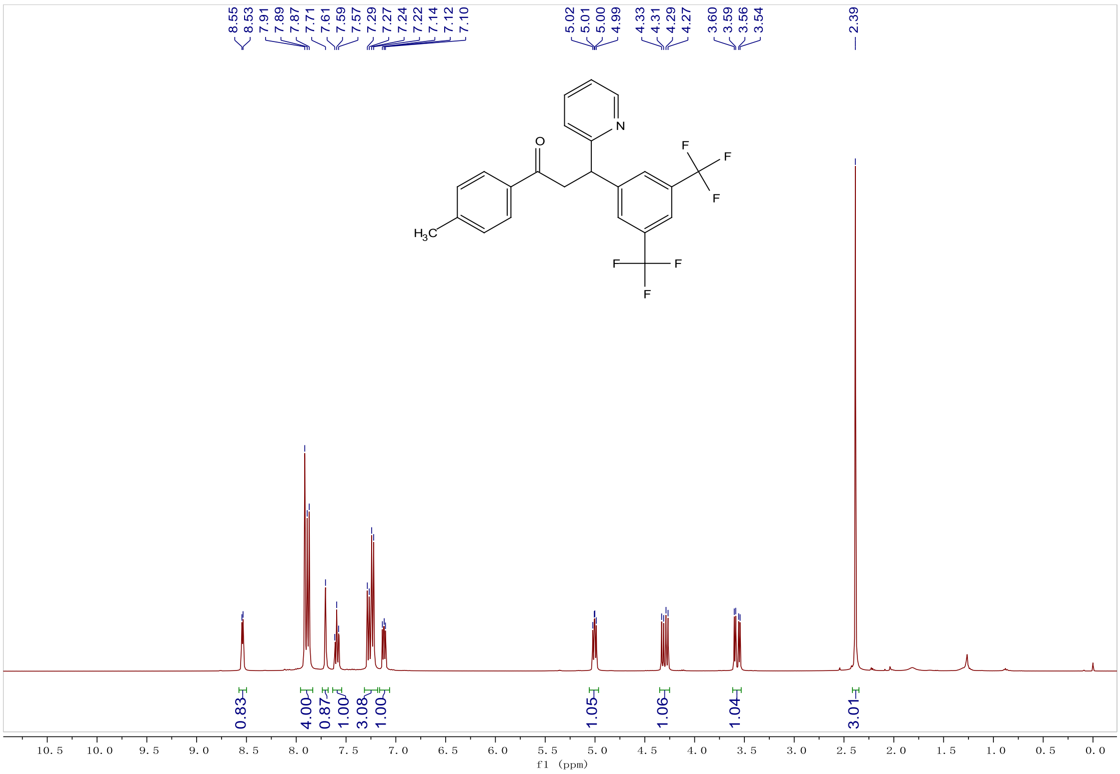

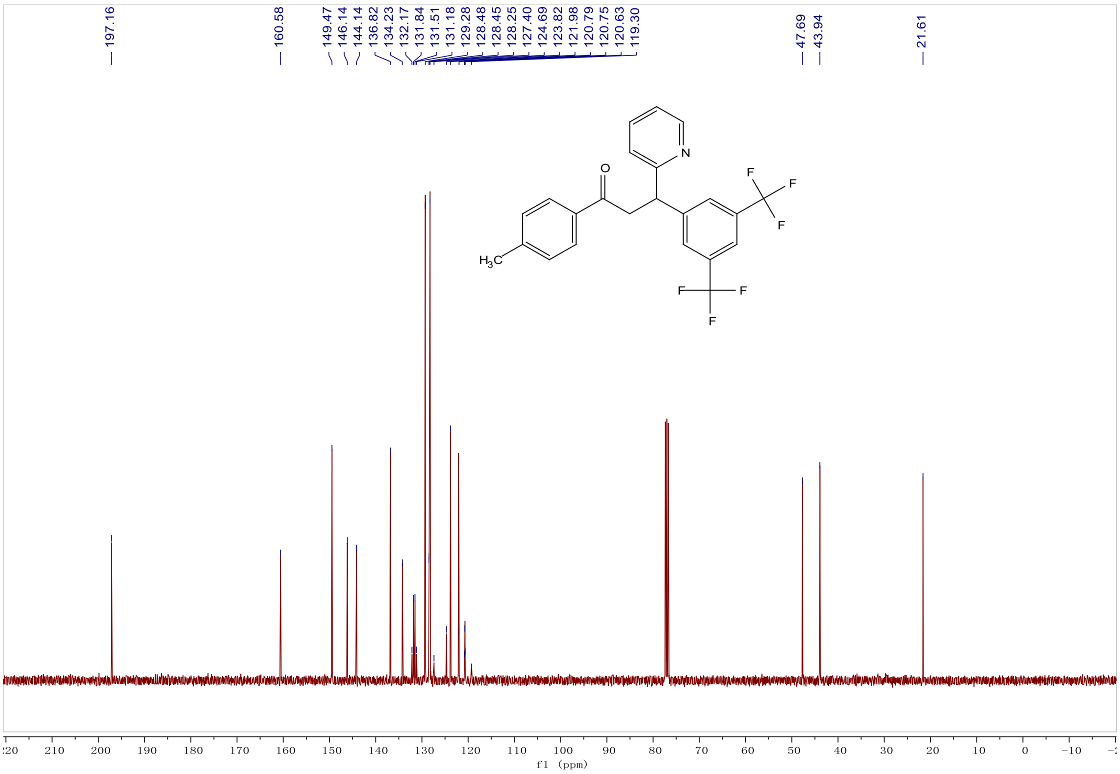

Supplementary Figure 55. ^{1}H and ^{13} C NMR spectra for compound **3oo**

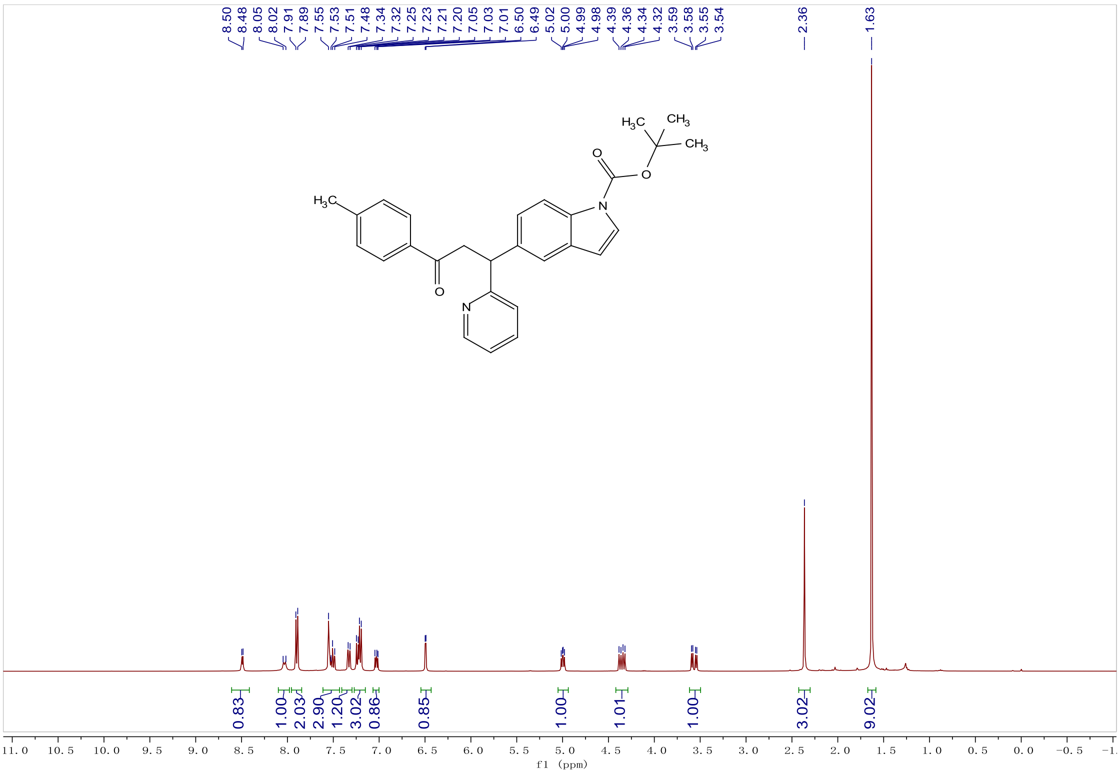

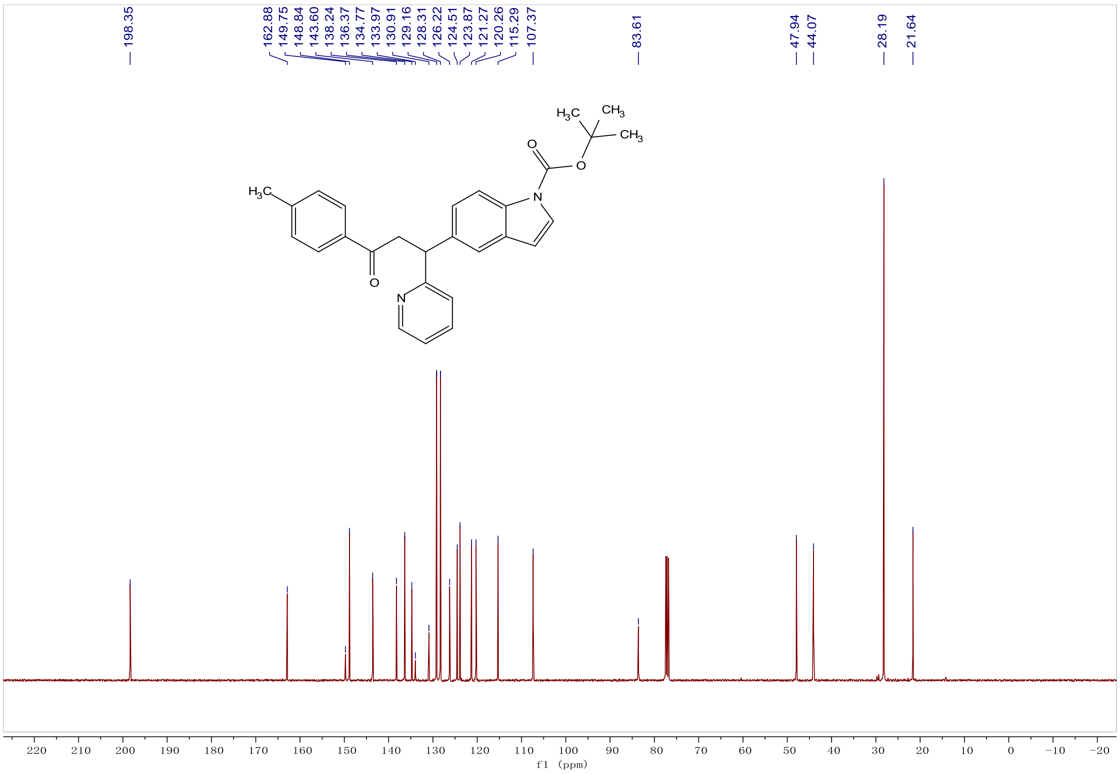

Supplementary Figure 56. ^{1}H and ^{13} C NMR spectra for compound **3pp**

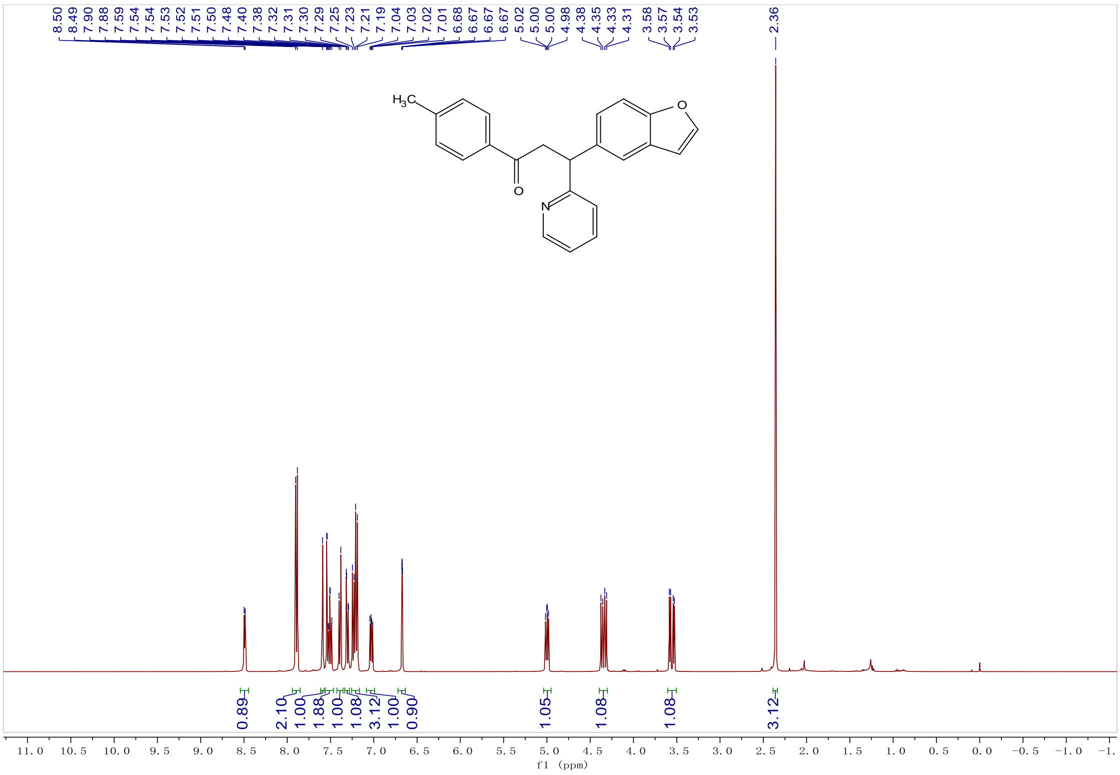

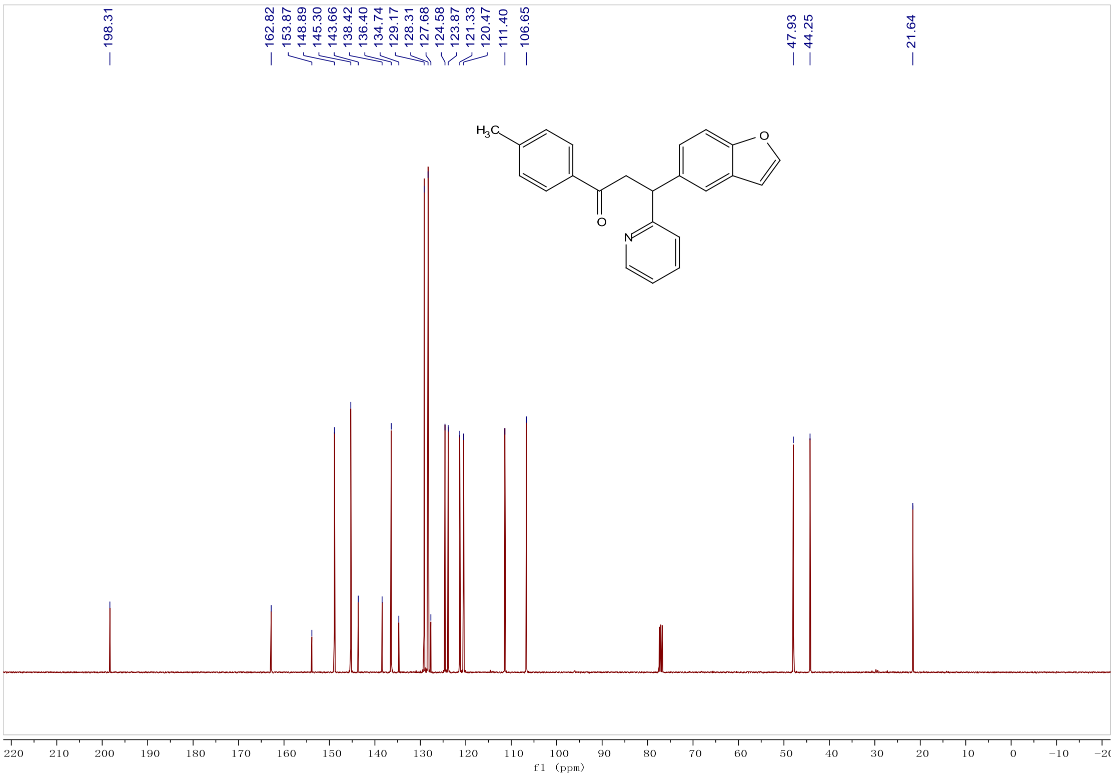

Supplementary Figure 57. ^{1}H and ^{13} C NMR spectra for compound **3qq**

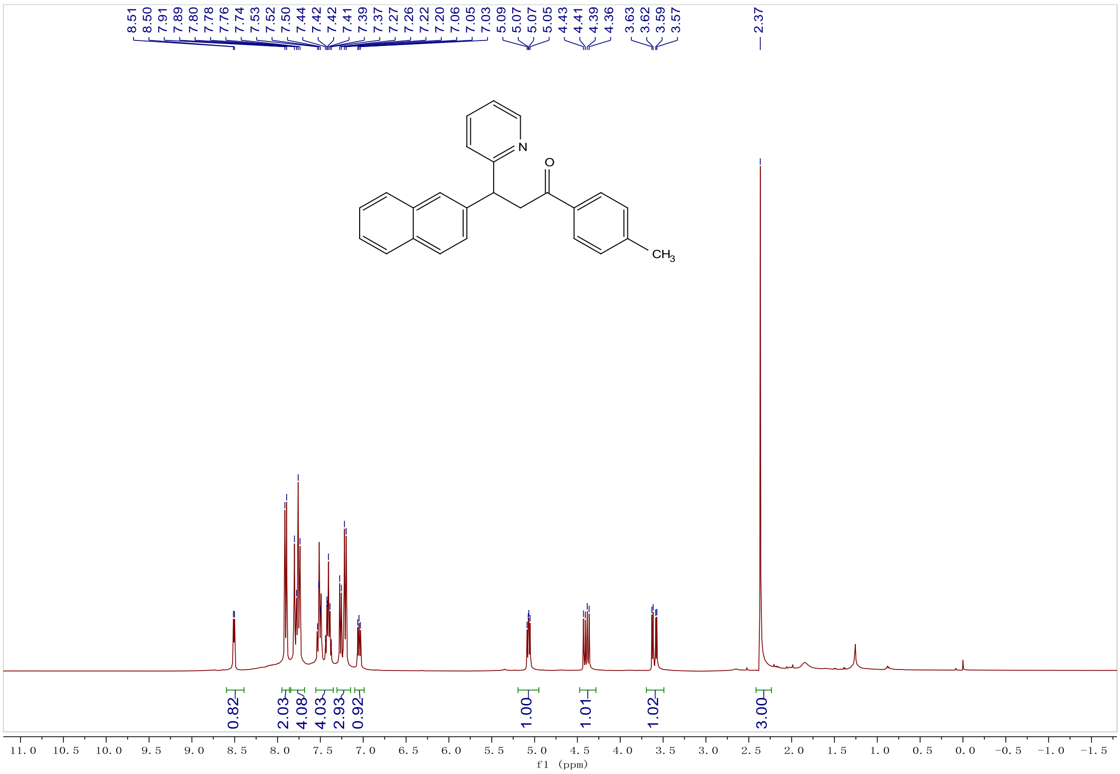

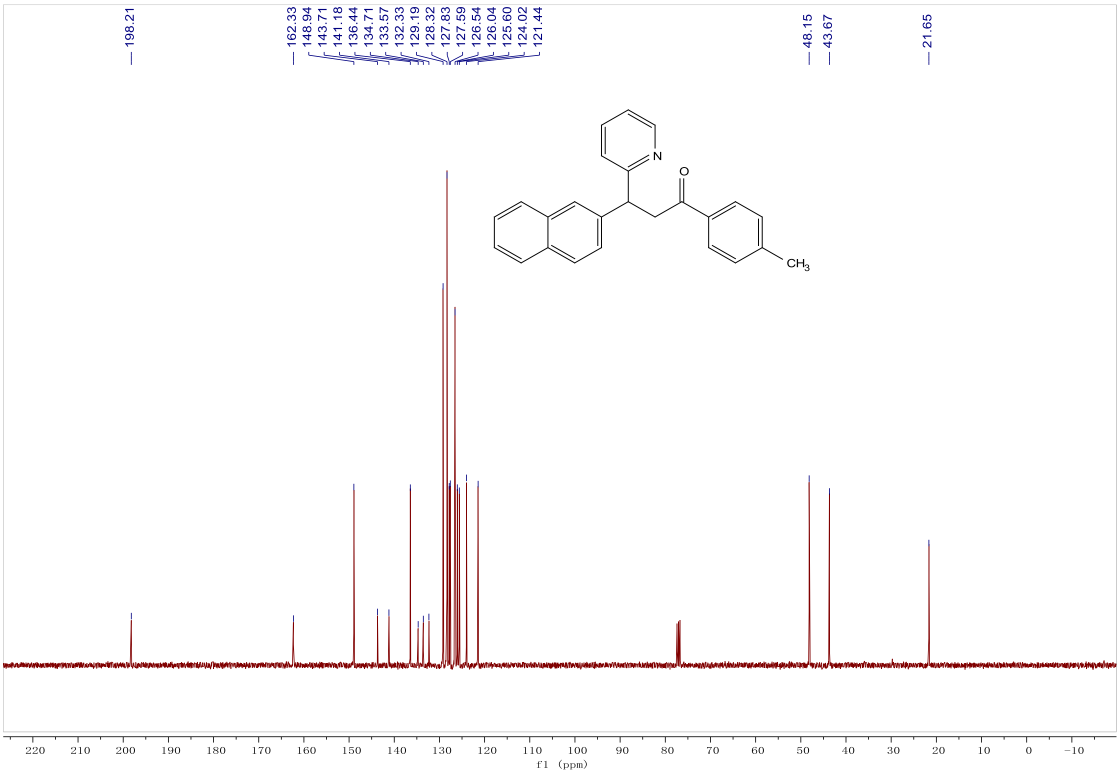

Supplementary Figure 58. ^{1}H and ^{13}C NMR spectra for compound **3rr**

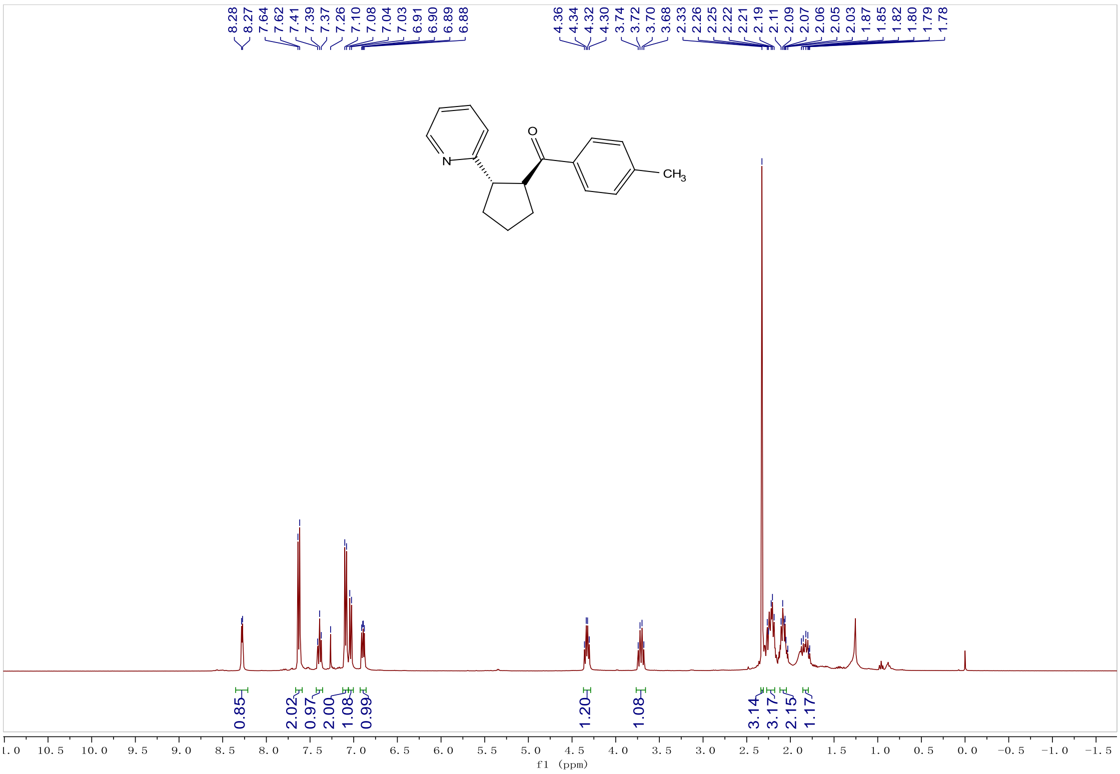
8.28
8.27
7.64
7.62
7.41
7.39
7.37
7.26
7.10
7.08
7.04
7.03
6.91
6.90
6.89
6.88
4.36
4.34
4.32
4.30
3.74
3.72
3.70
3.68
2.33
2.26
2.25
2.22
2.21
2.19
2.11
2.09
2.07
2.06
2.05
2.03
1.87
1.85
1.82
1.80
1.79
1.78
O
N
CH3
0.85
2.02
0.97
2.00
1.08
0.99
1.20
1.08
3.14
3.17
2.15
1.17
f1 (ppm)

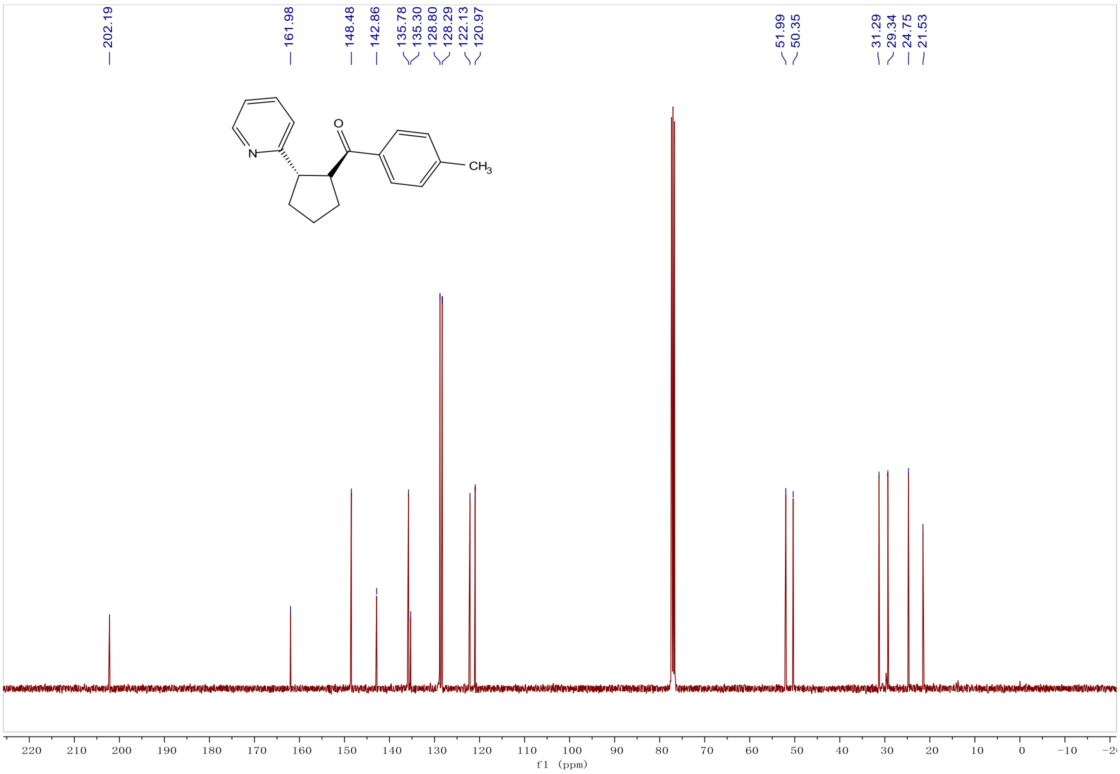
202.19
161.98
148.48
142.86
135.78
135.30
128.80
128.29
122.13
120.97
51.99
50.35
31.29
29.34
24.75
21.53
O
N
CH3
f1 (ppm)

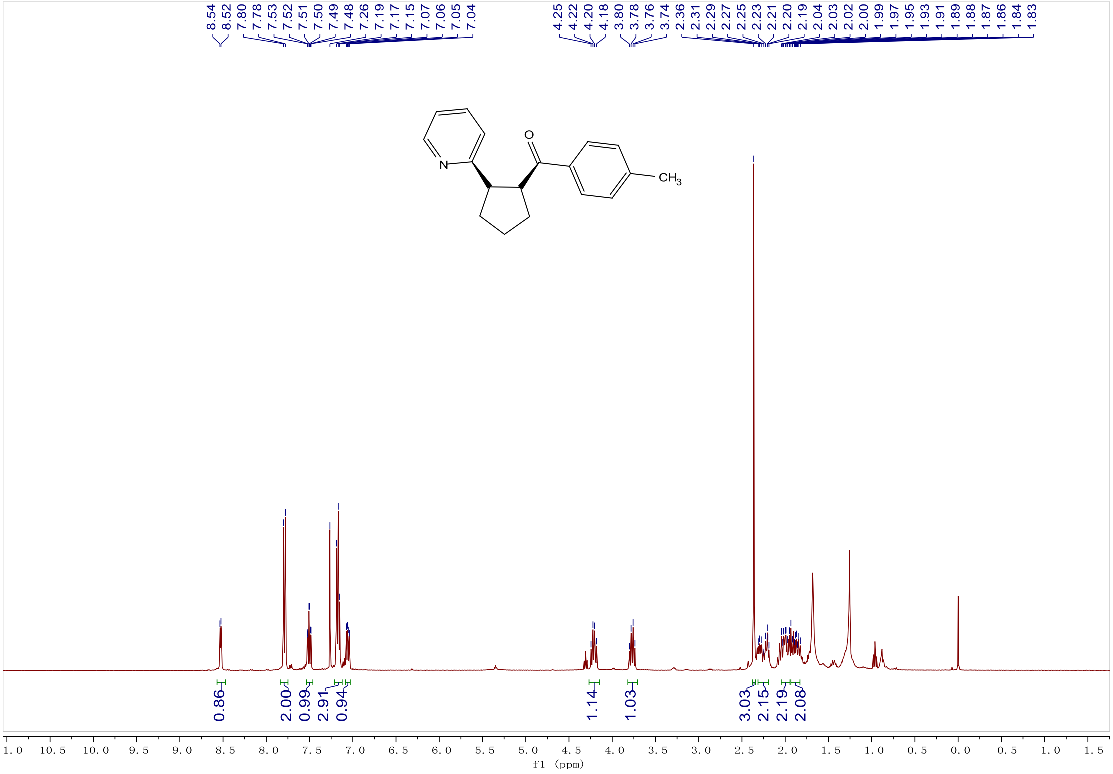

Supplementary Figure 59. ^{1}H and ^{13}C NMR spectra for compound **3ss**

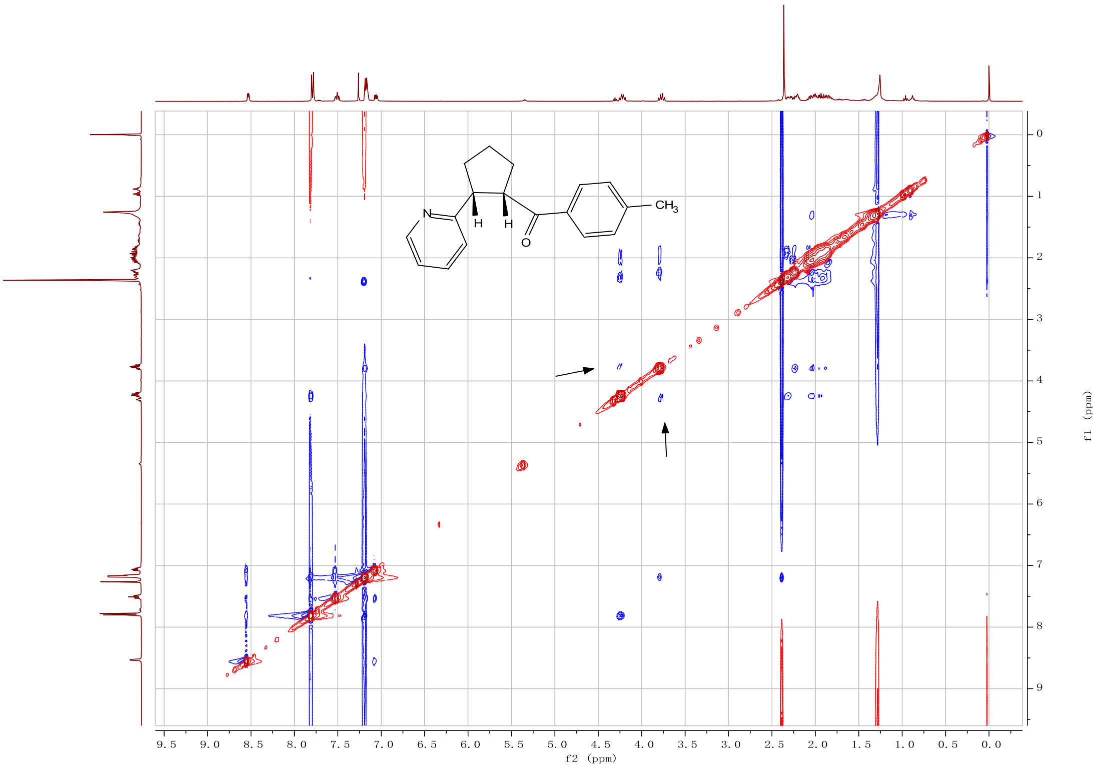

Supplementary Figure 60. NOESY spectrum of **3ss**

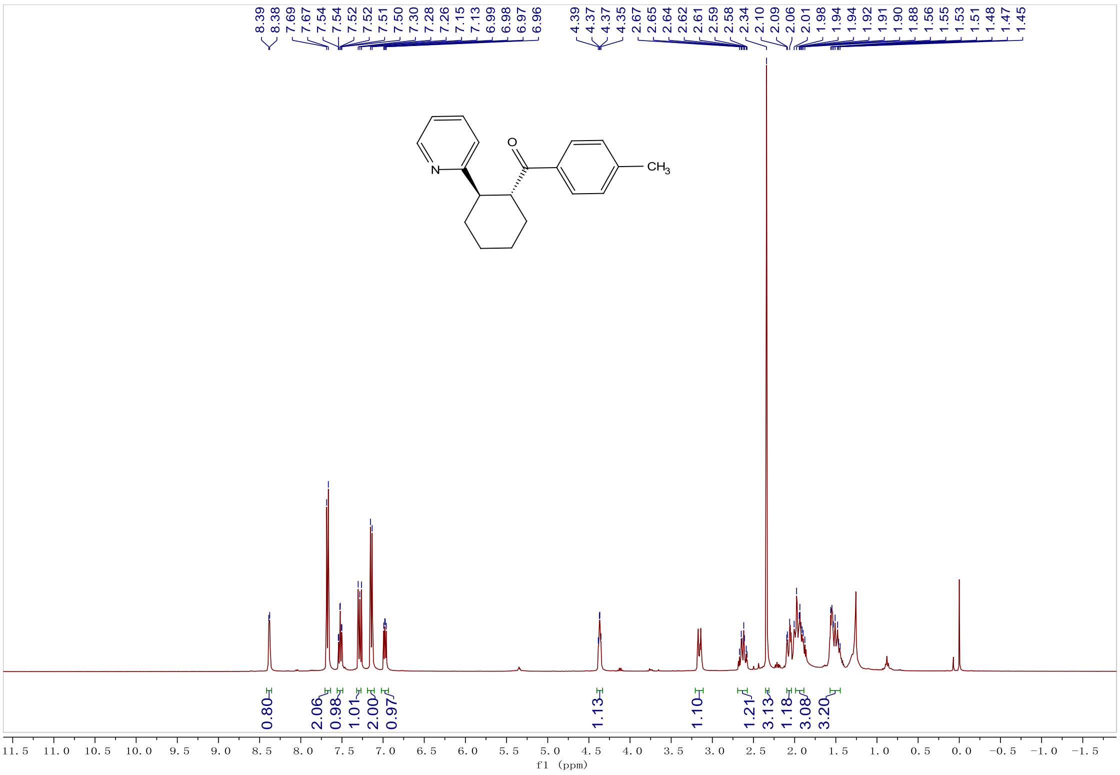
8.39
8.38
7.69
7.67
7.54
7.54
7.52
7.52
7.51
7.50
7.30
7.28
7.26
7.15
7.13
6.99
6.98
6.97
6.96
4.39
4.37
4.37
4.35
2.67
2.65
2.64
2.62
2.61
2.59
2.58
2.34
2.10
2.09
2.06
2.01
1.98
1.94
1.94
1.92
1.91
1.90
1.88
1.56
1.55
1.53
1.51
1.48
1.47
1.45
CH3
O
N
0.80
2.06
0.98
1.01
2.00
0.97
1.13
1.10
1.21
3.13
1.18
3.08
3.20
f1 (ppm)

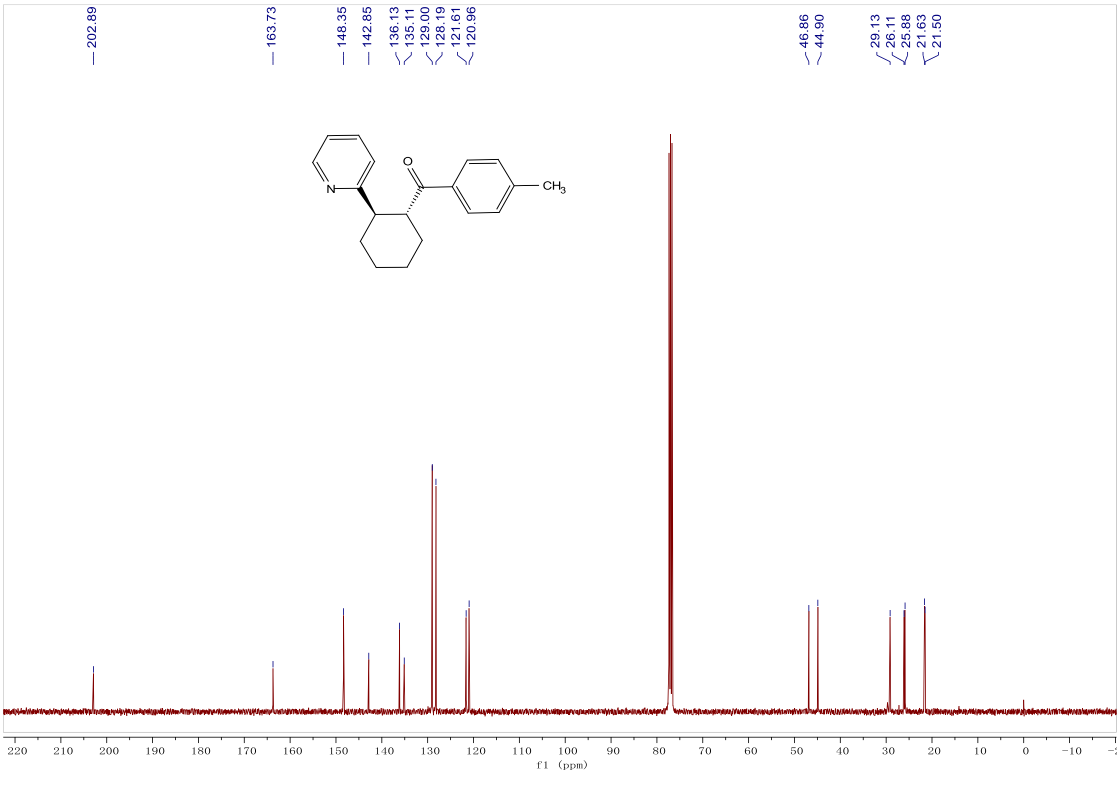
202.89
163.73
148.35
142.85
136.13
135.11
129.00
128.19
121.61
120.96
46.86
44.90
29.13
26.11
25.88
21.63
21.50
CH3
O
N
f1 (ppm)

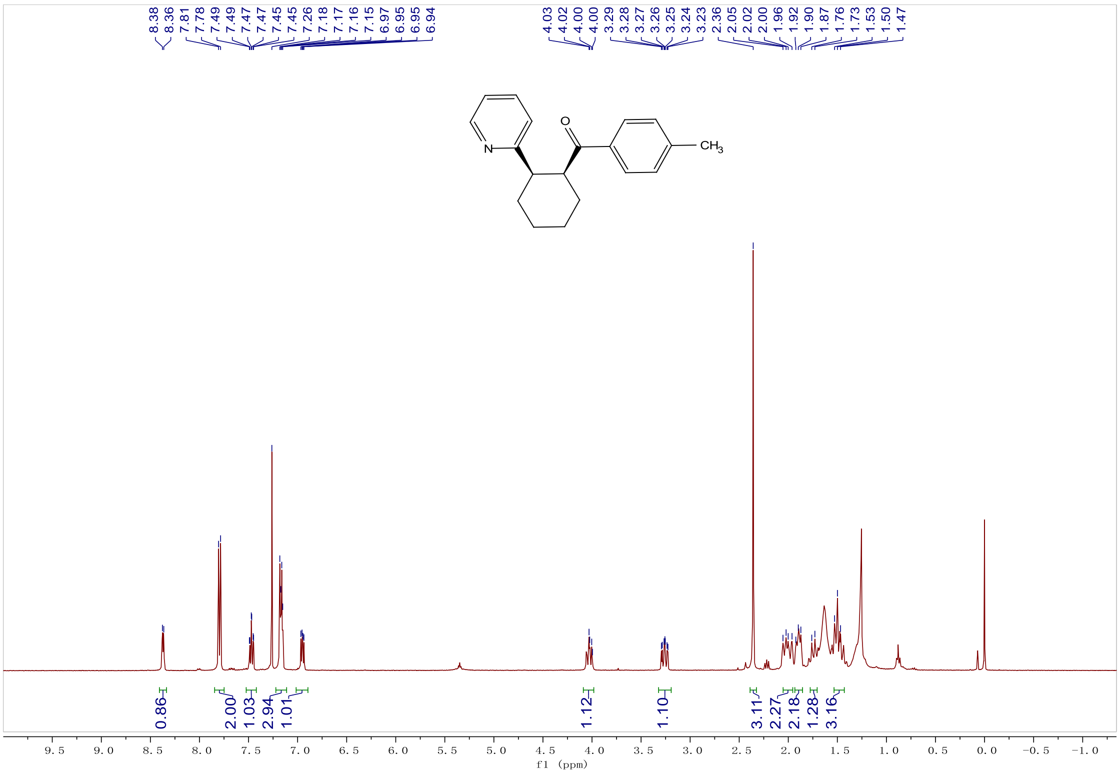

Supplementary Figure 61. ^{1}H and ^{13} C NMR spectra for compound **3tt**

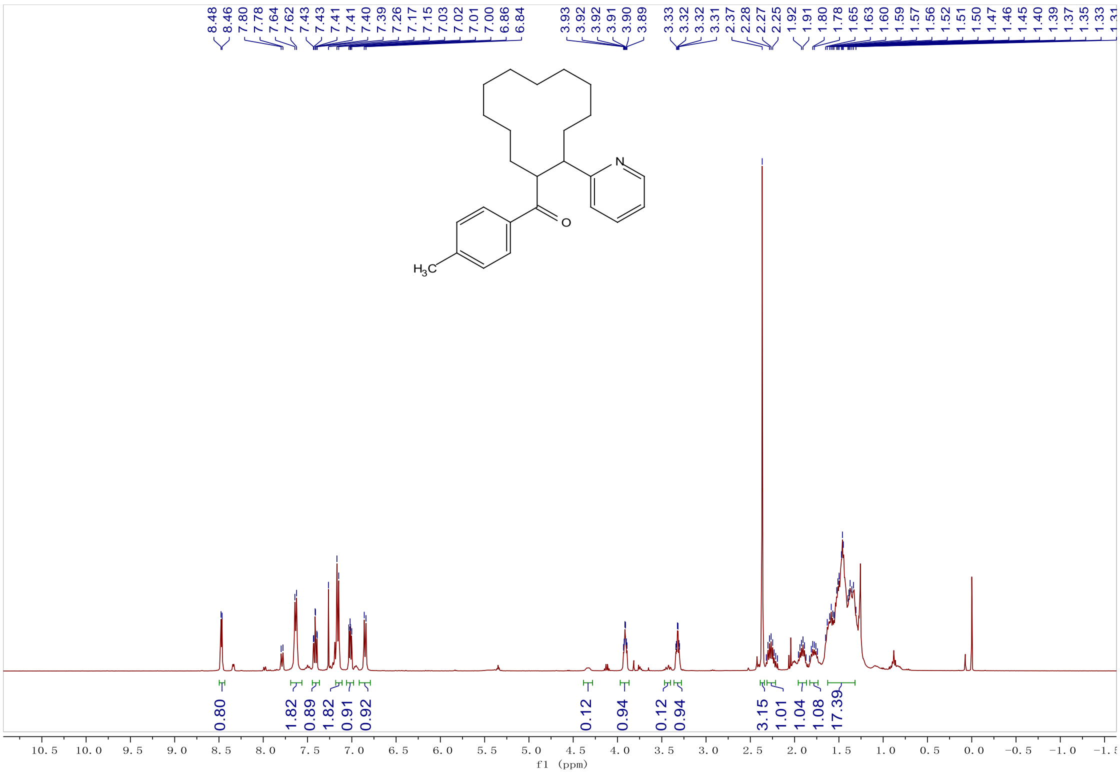

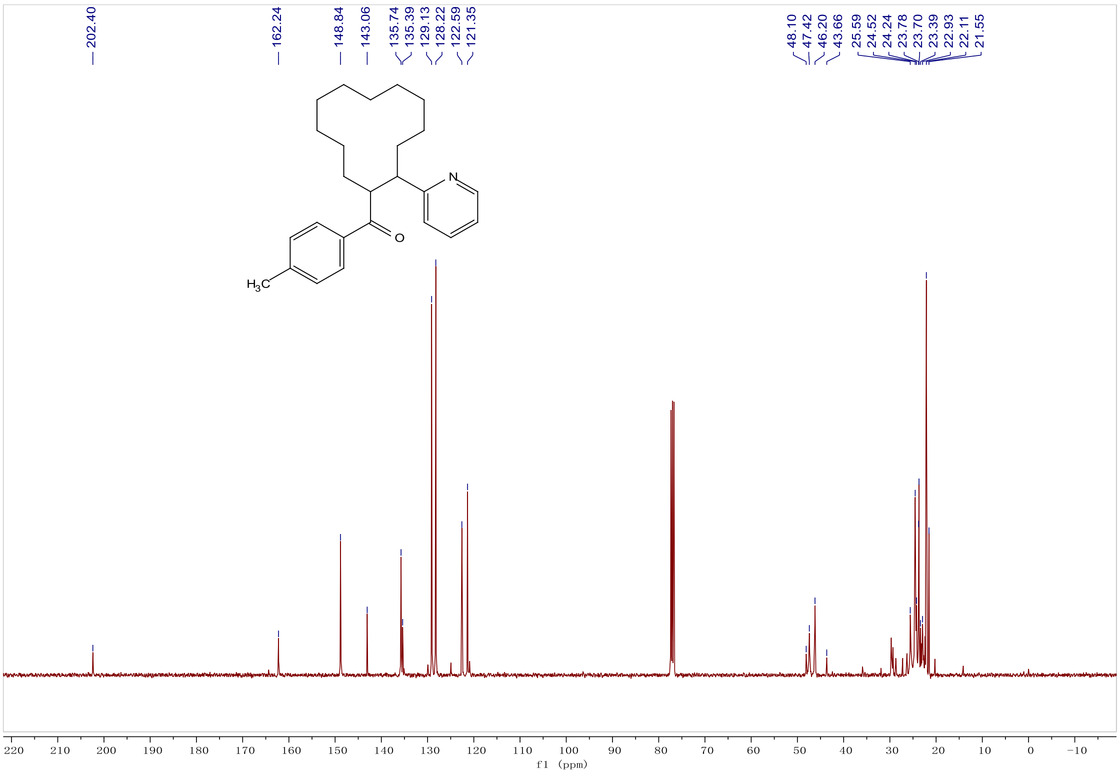

Supplementary Figure 62. ^{1}H and ^{13}C NMR spectra for compound **3uu**

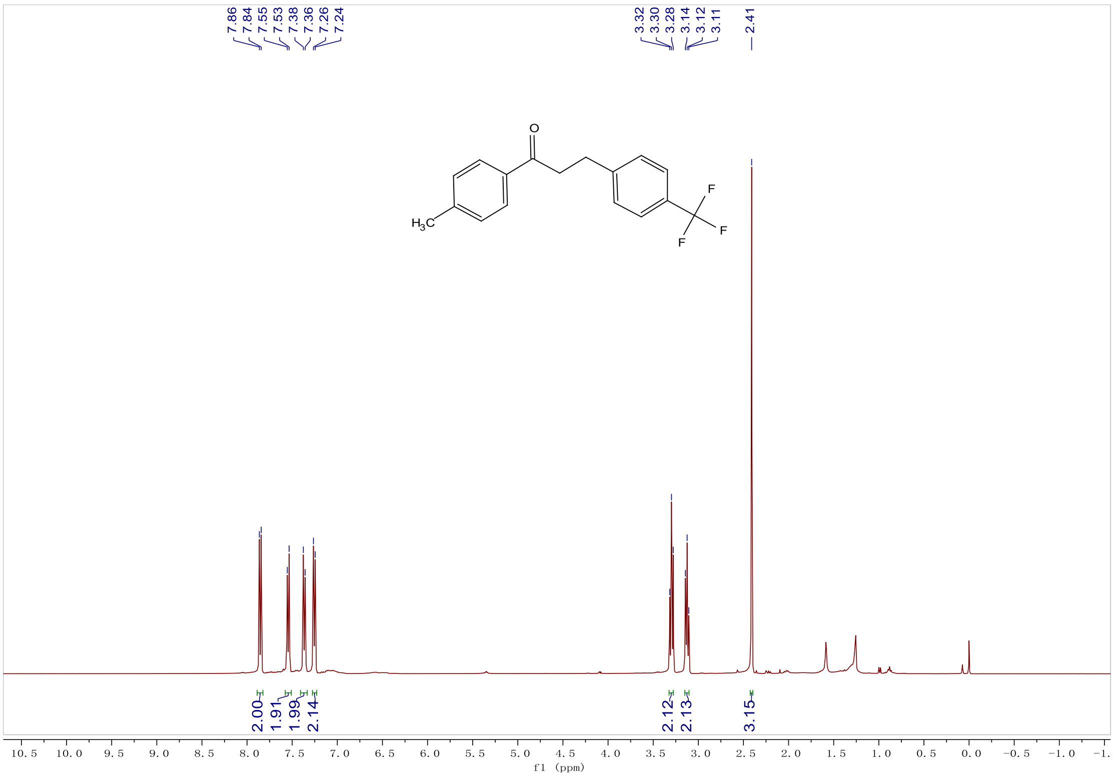

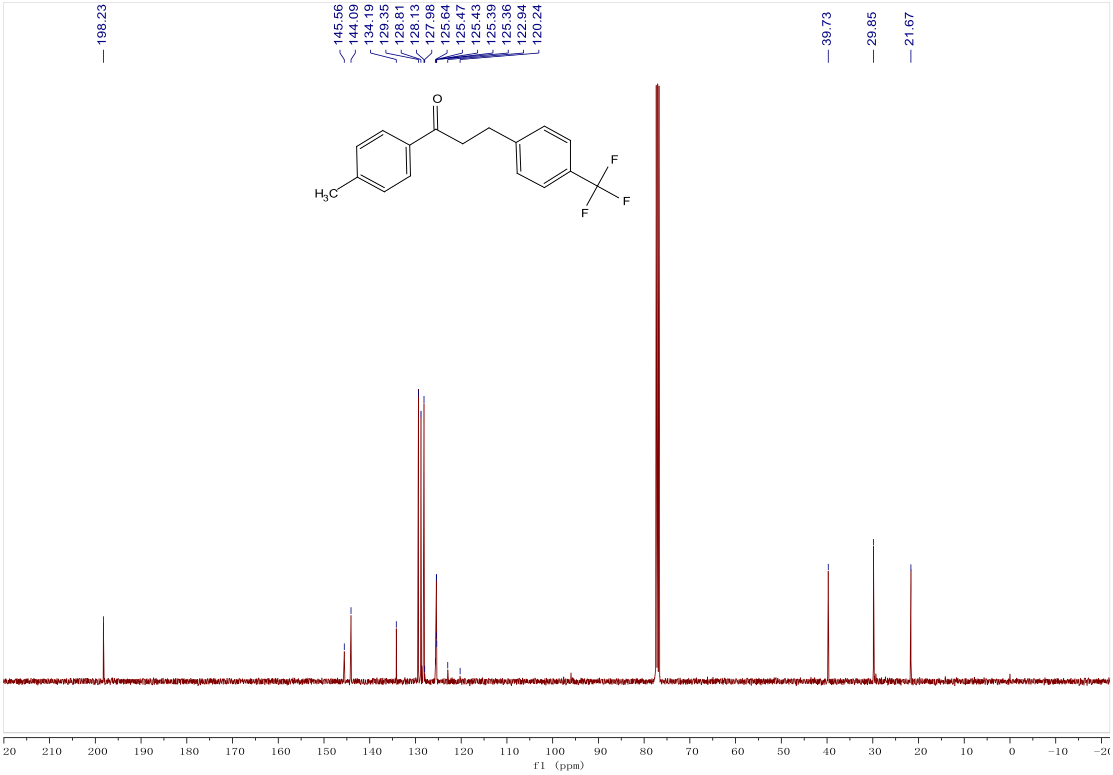

Supplementary Figure 63. ^{1}H and ^{13}C NMR spectra for compound **3vv**

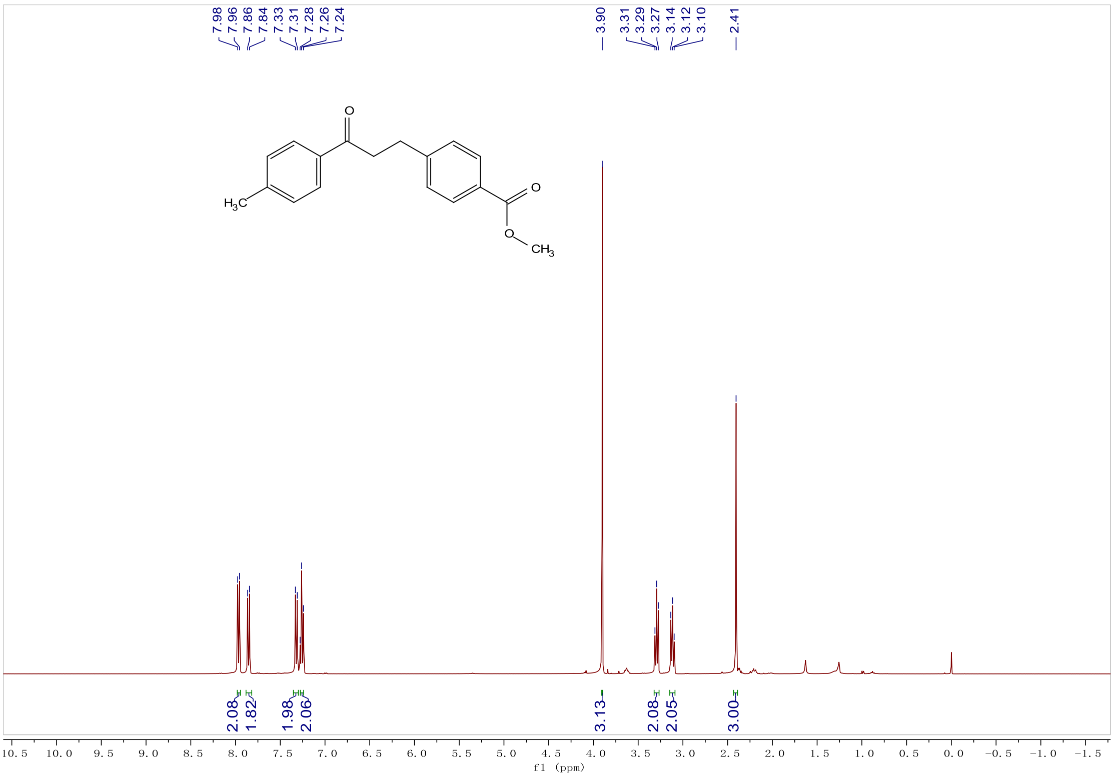

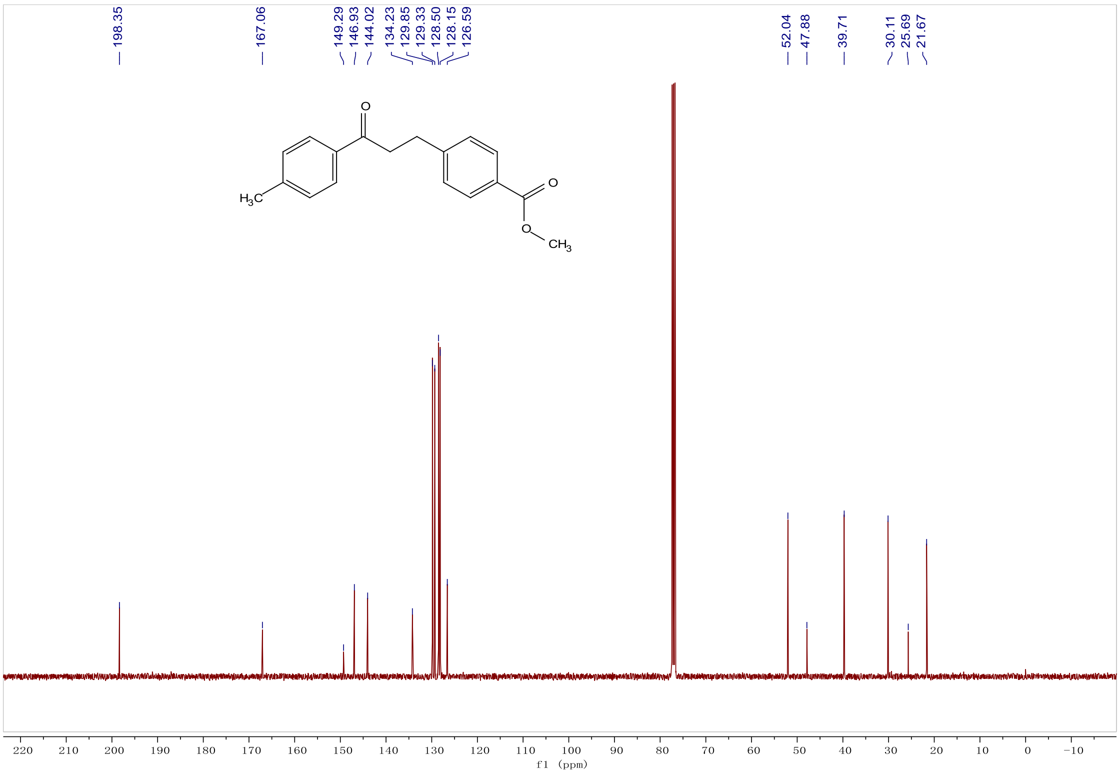

Supplementary Figure 64. ^{1}H and ^{13}C NMR spectra for compound **3ww**

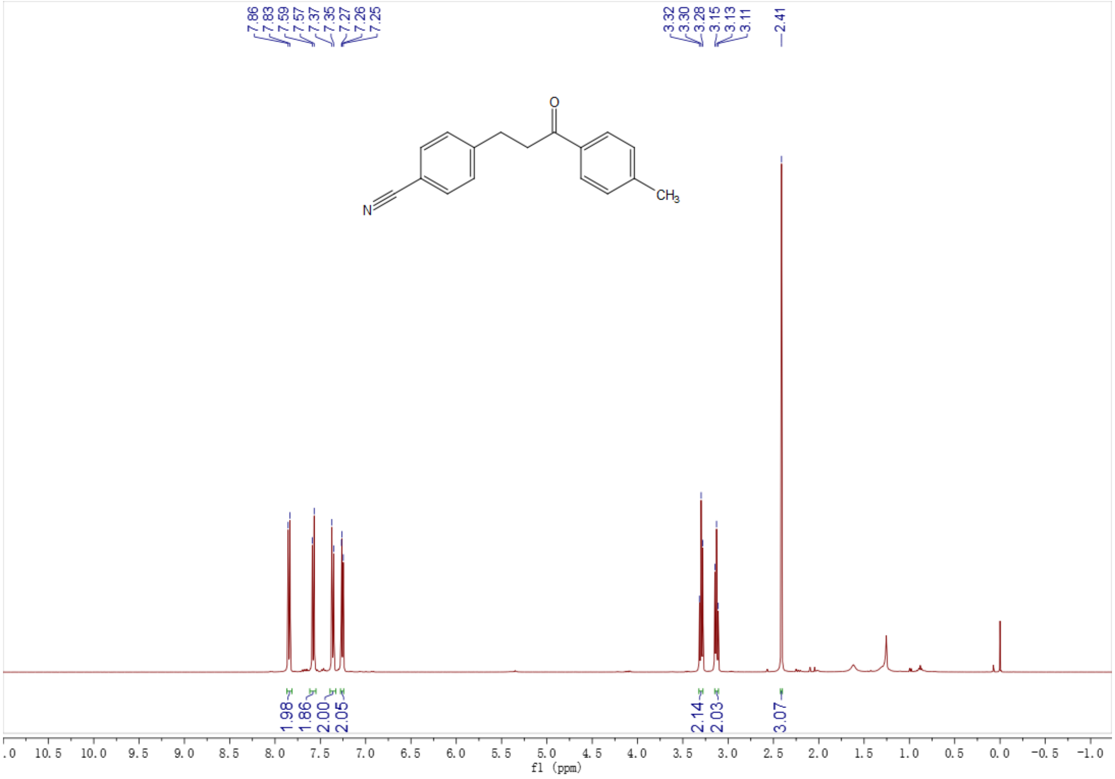

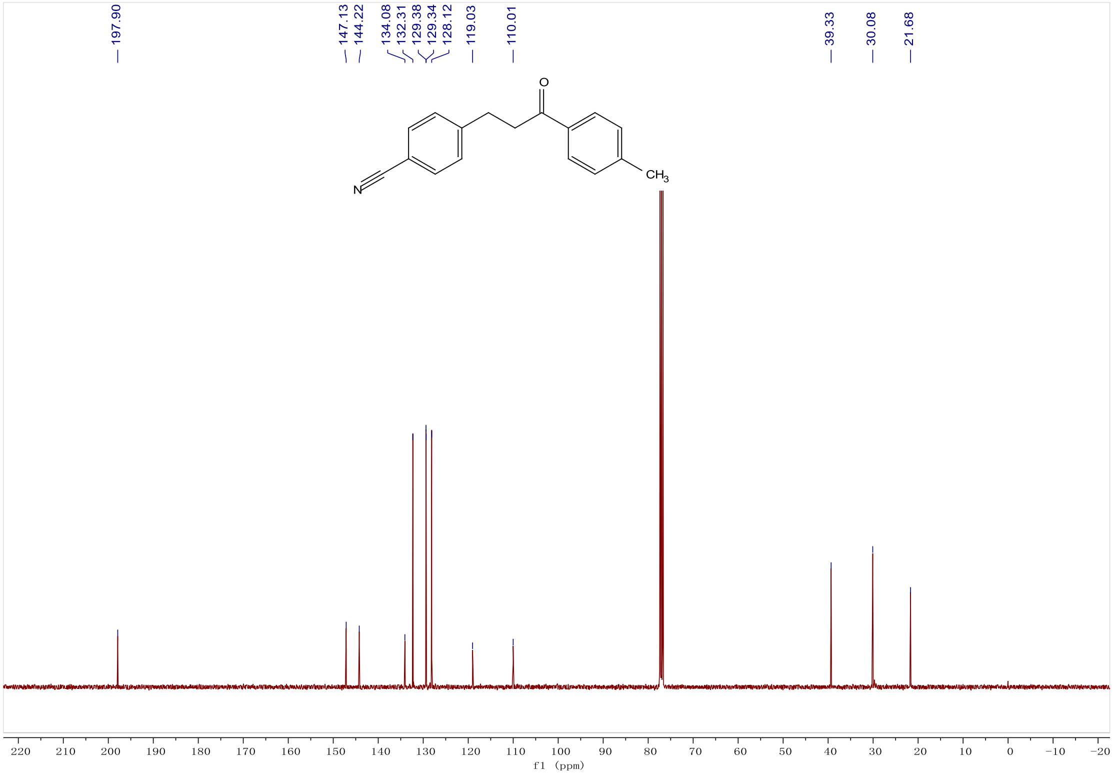

Supplementary Figure 65. ^{1}H and ^{13}C NMR spectra for compound **3xx**

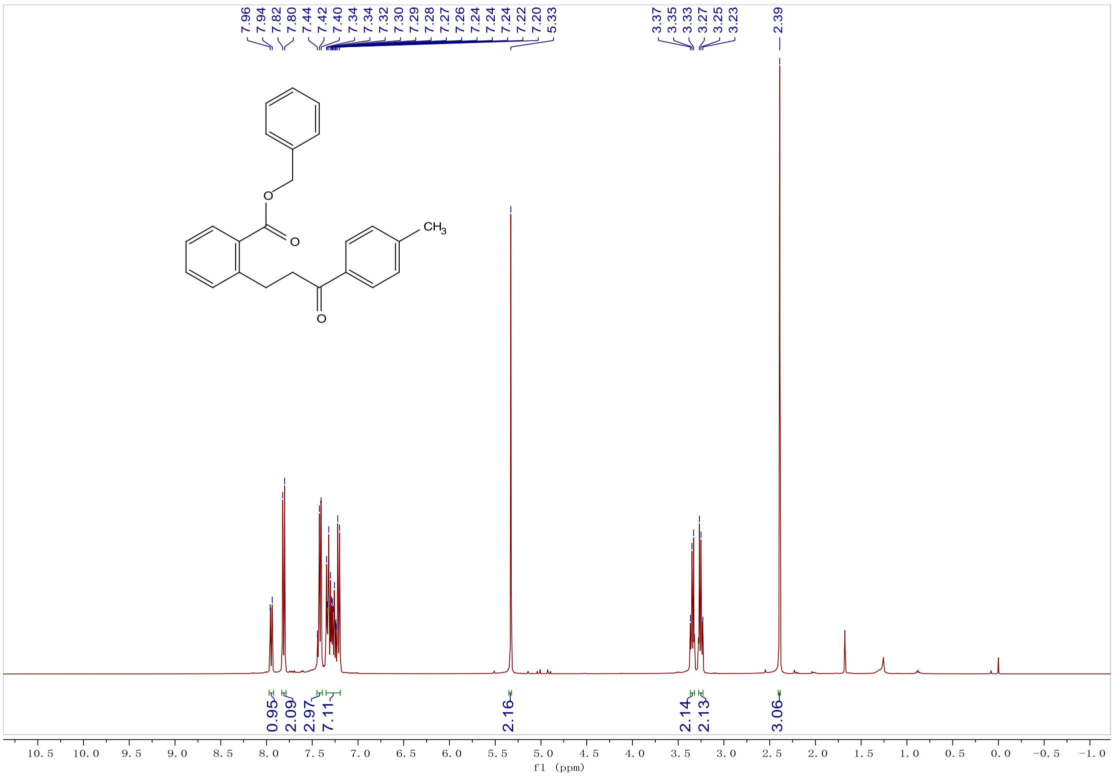

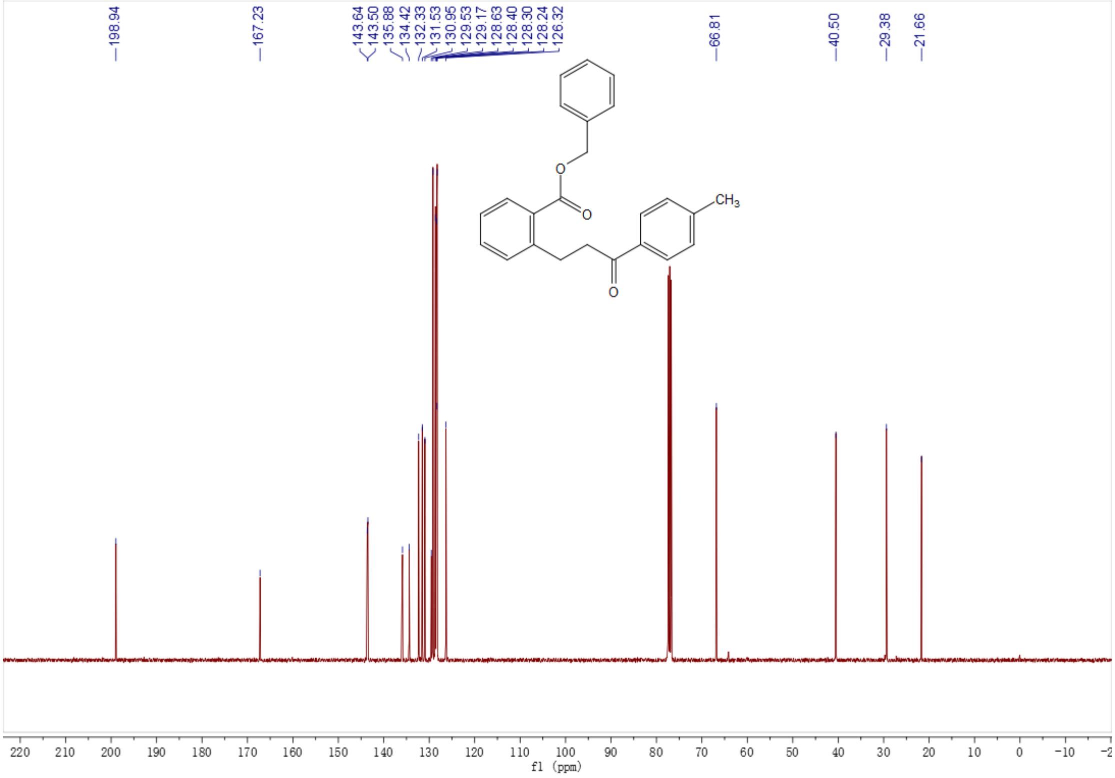

Supplementary Figure 66. ^{1}H and ^{13}C NMR spectra for compound **3yy**

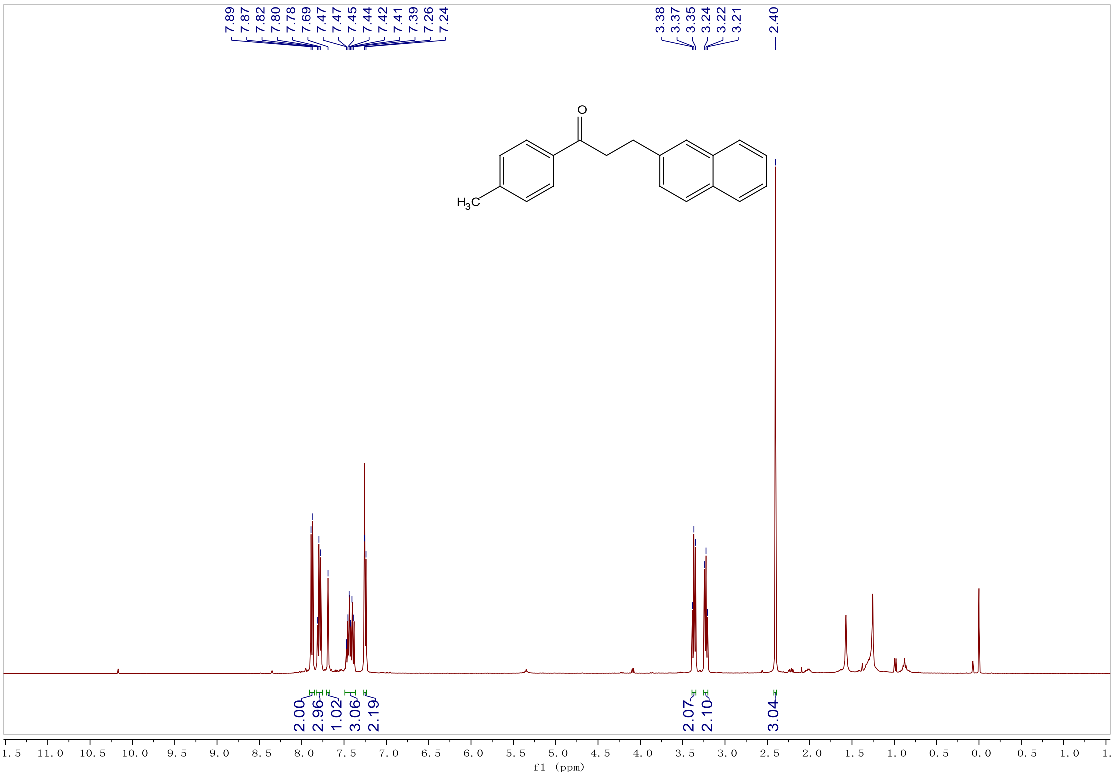

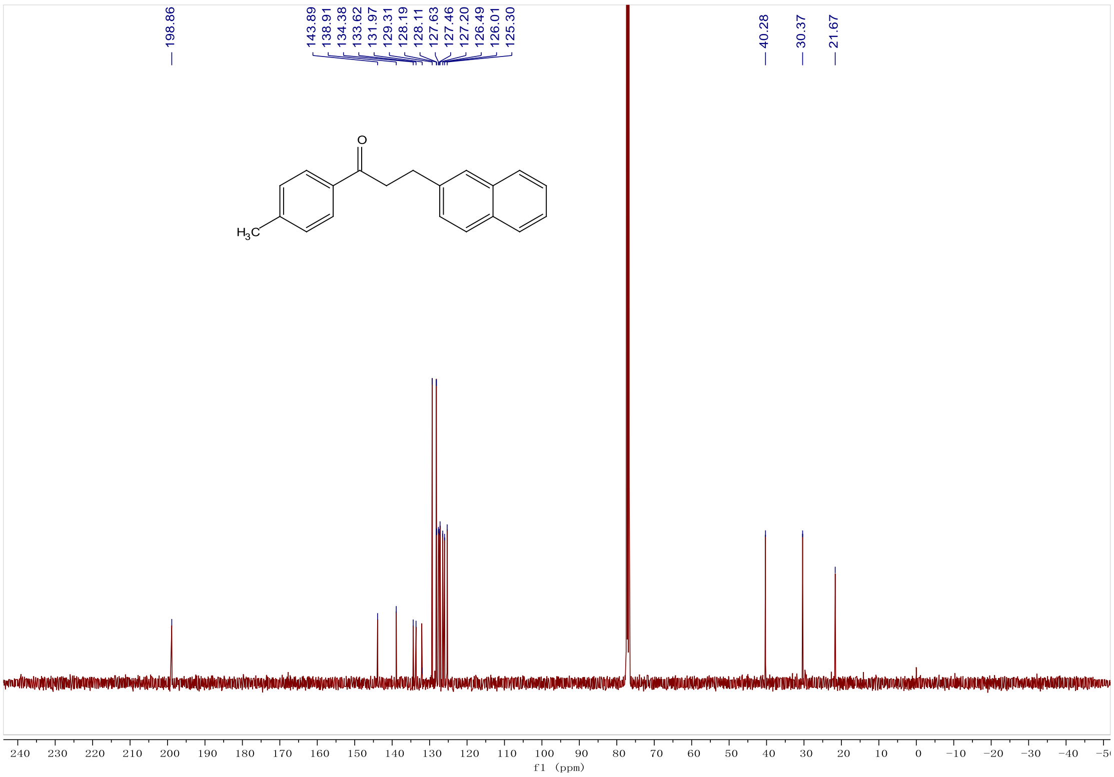

Supplementary Figure 67. ^{1}H and ^{13} C NMR spectra for compound **3zz**

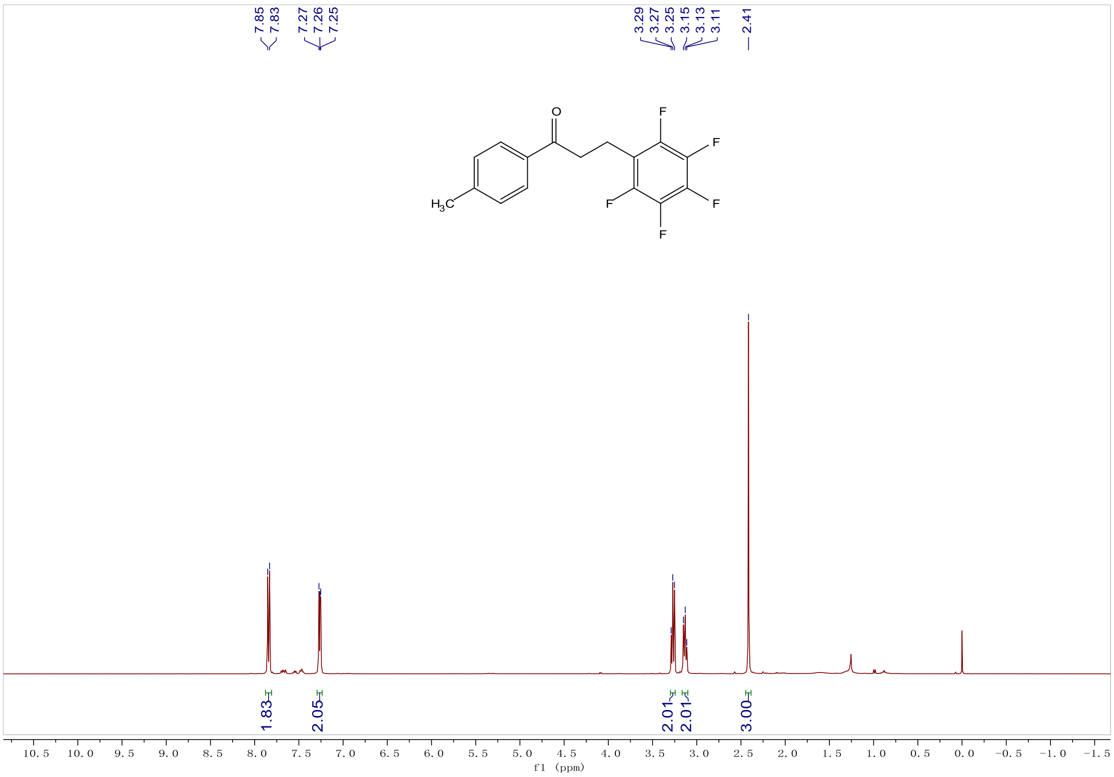

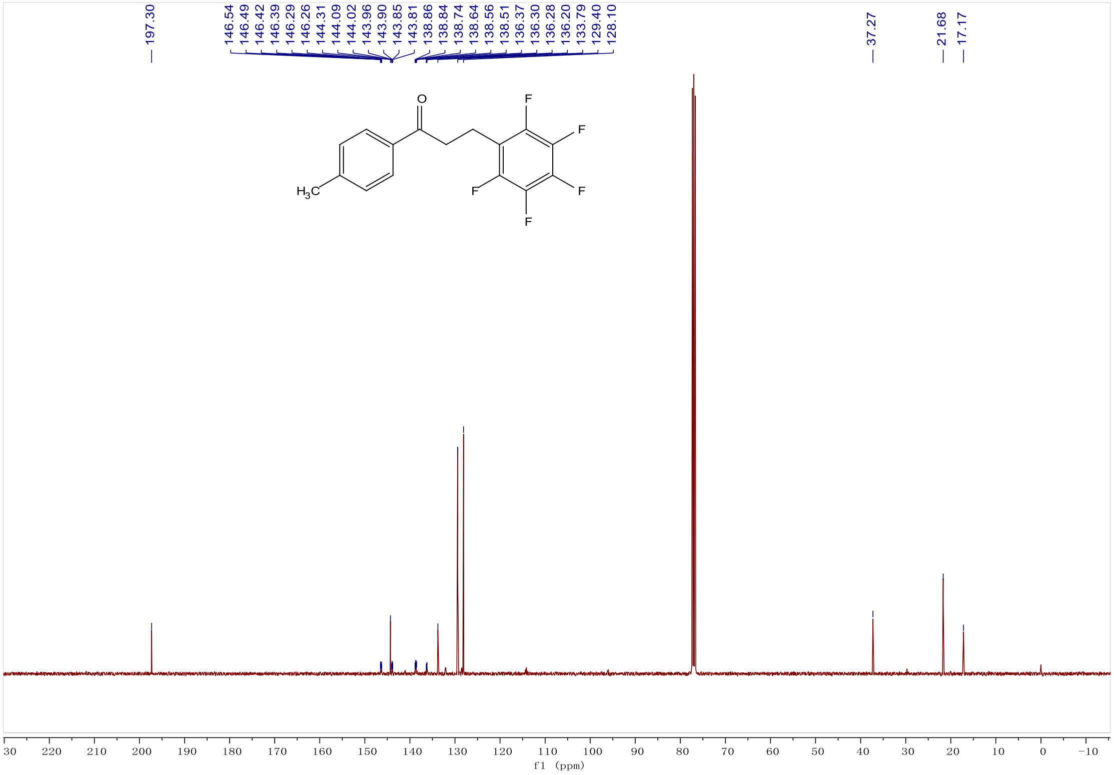

Supplementary Figure 68. ^{1}H and ^{13} C NMR spectra for compound **3aA**

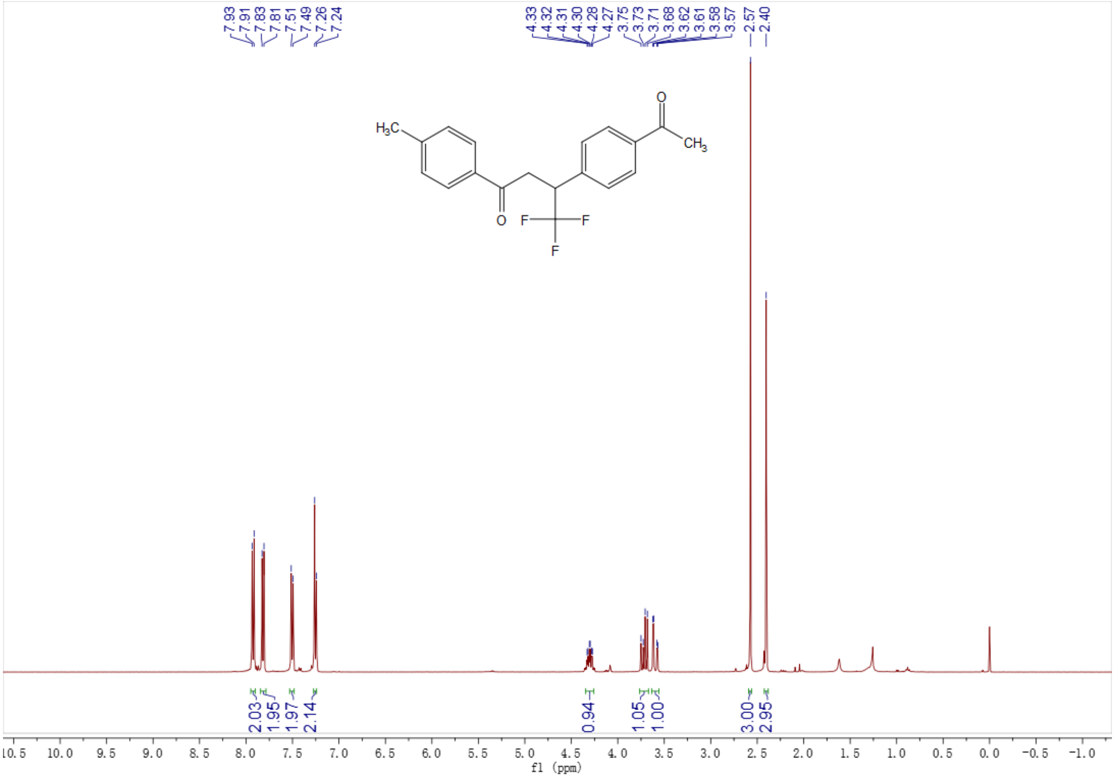

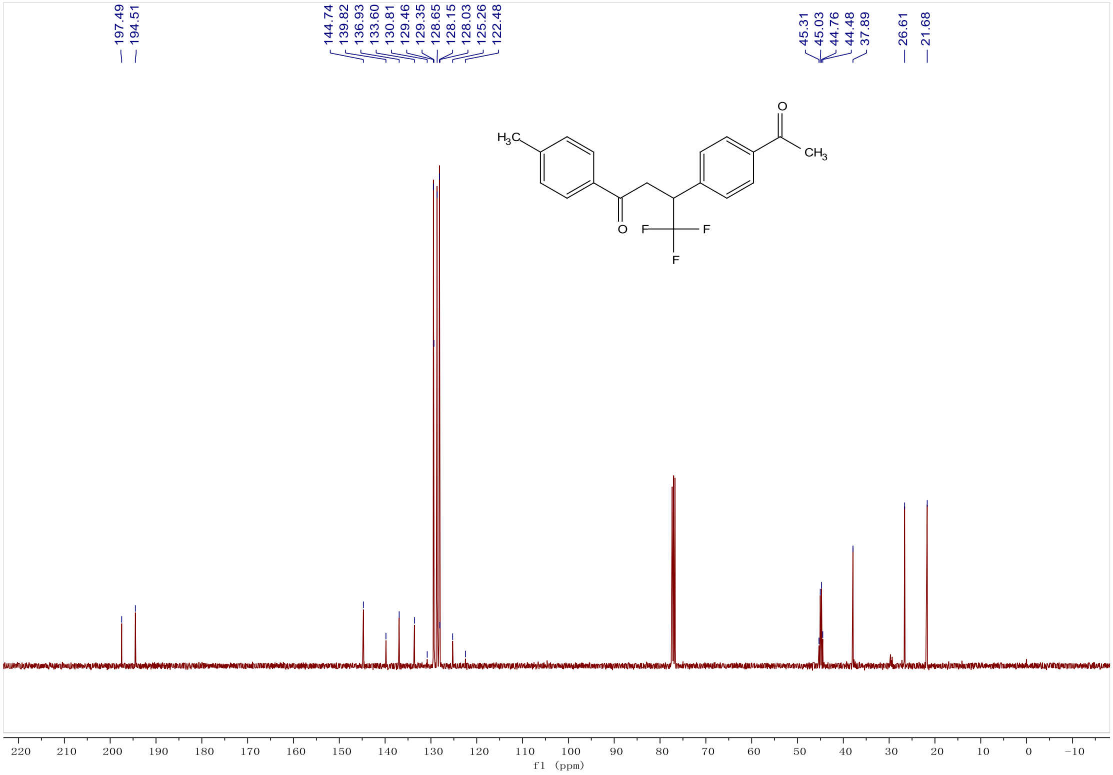

Supplementary Figure 69. ^{1}H and ^{13} C NMR spectra for compound **3bB**

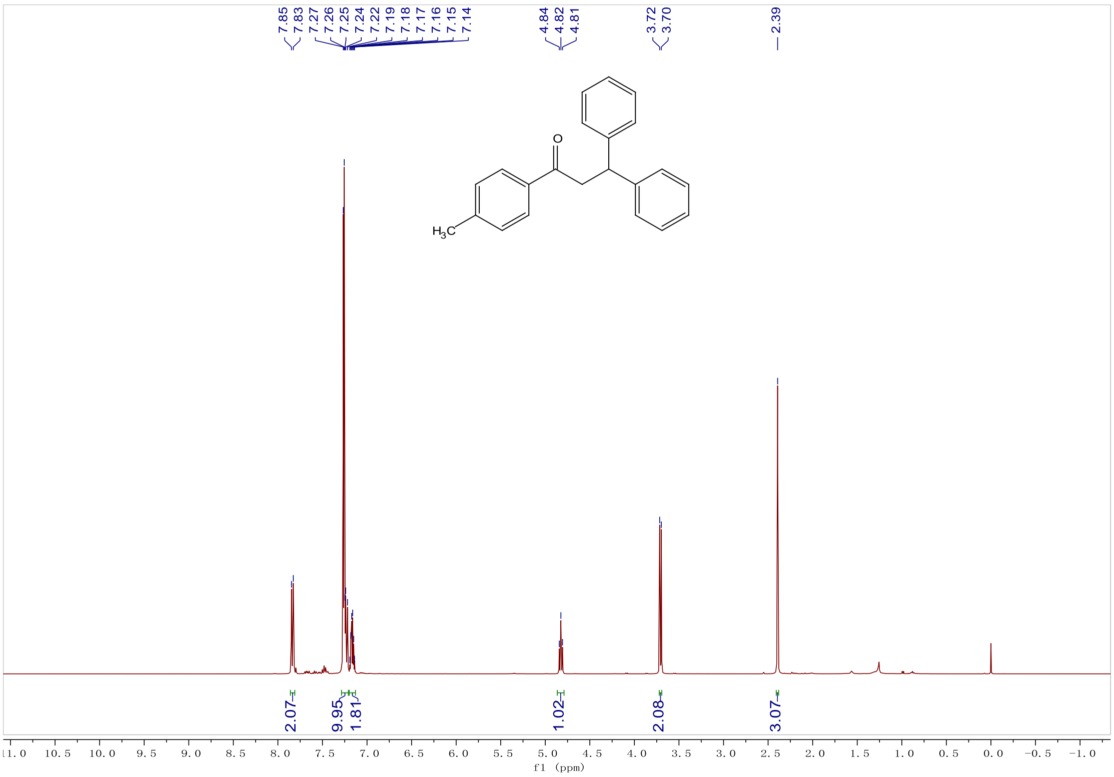

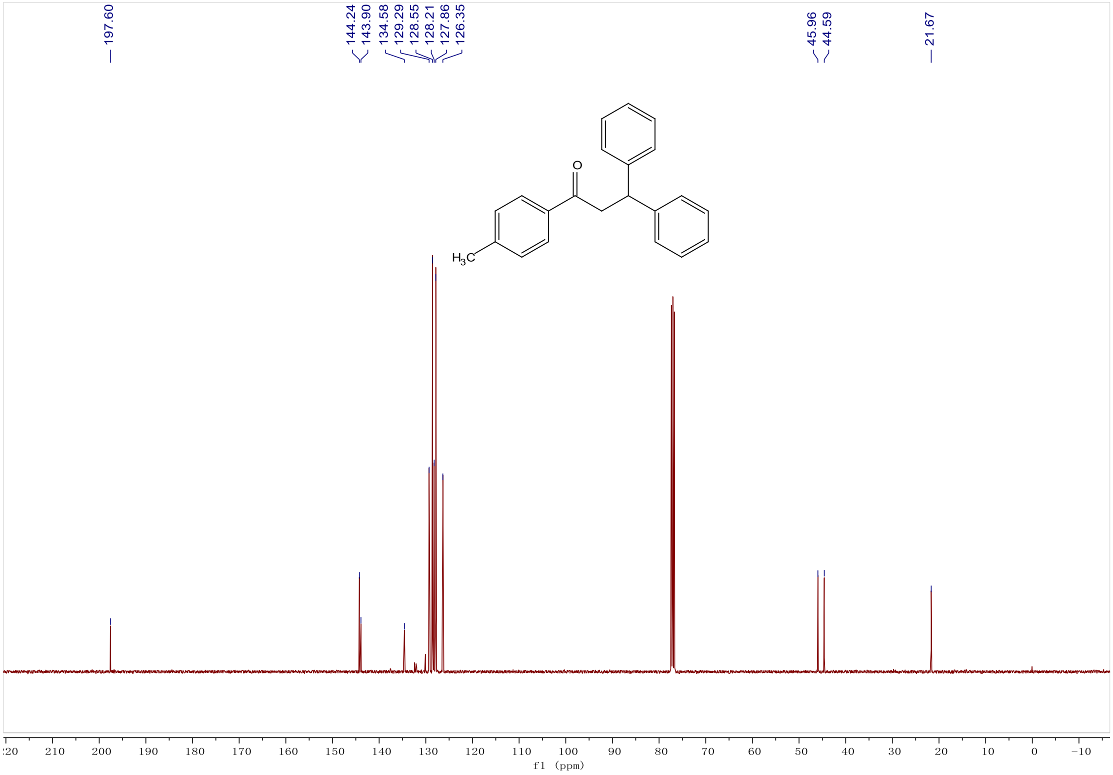

Supplementary Figure 70. ^{1}H and ^{13}C NMR spectra for compound **3cC**

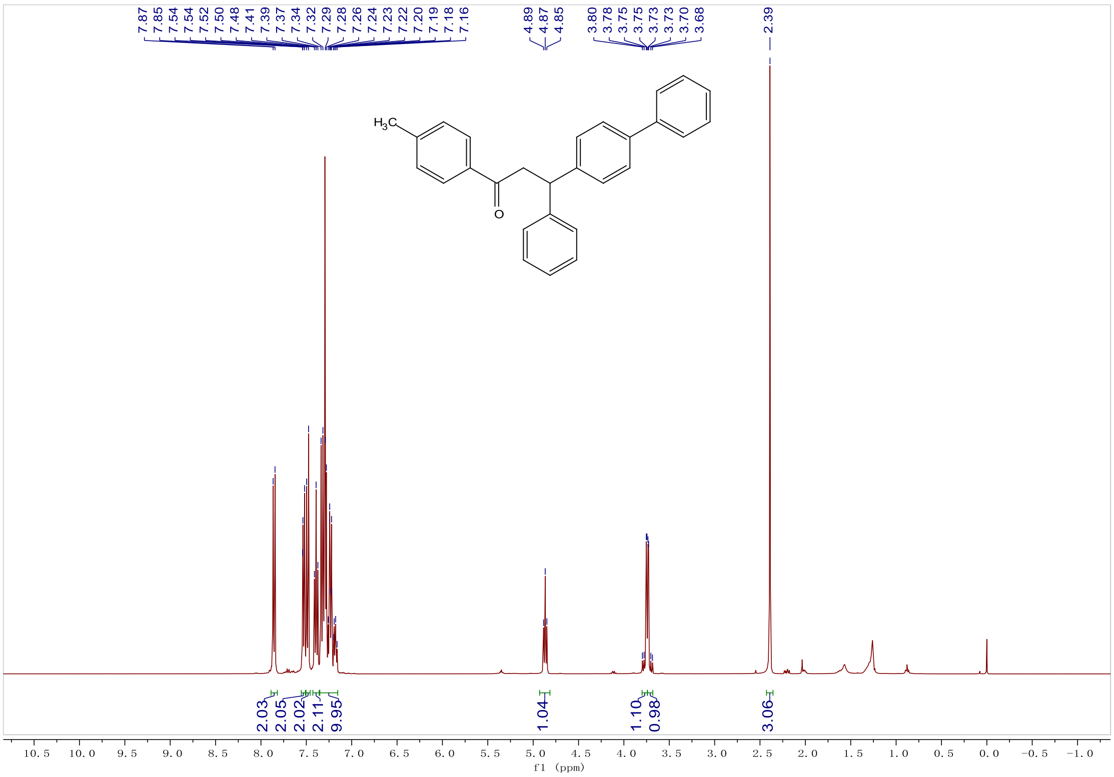

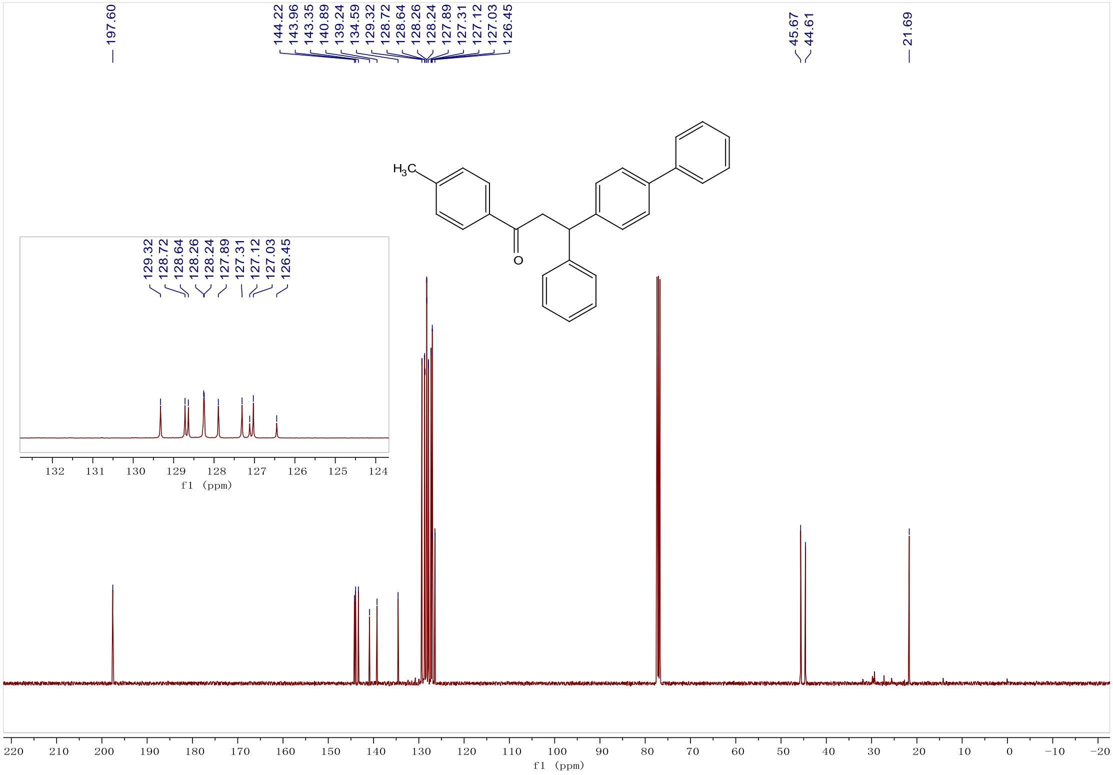

Supplementary Figure 71. ^{1}H and ^{13} C NMR spectra for compound **3dD**

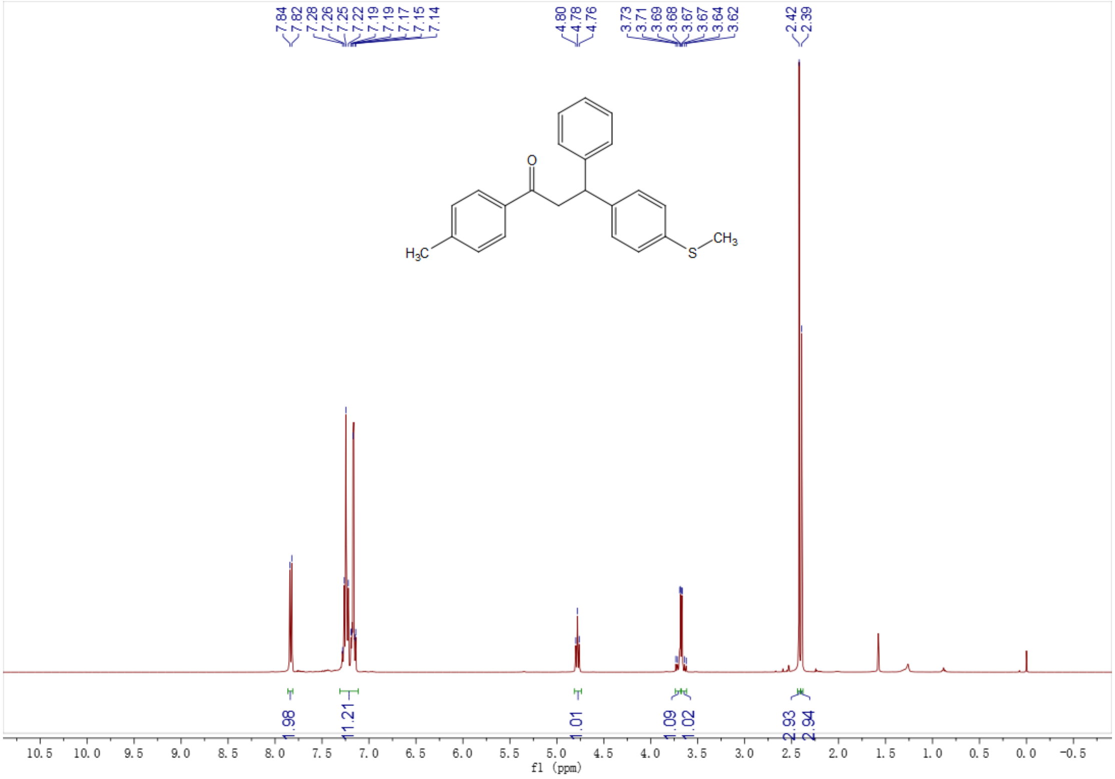

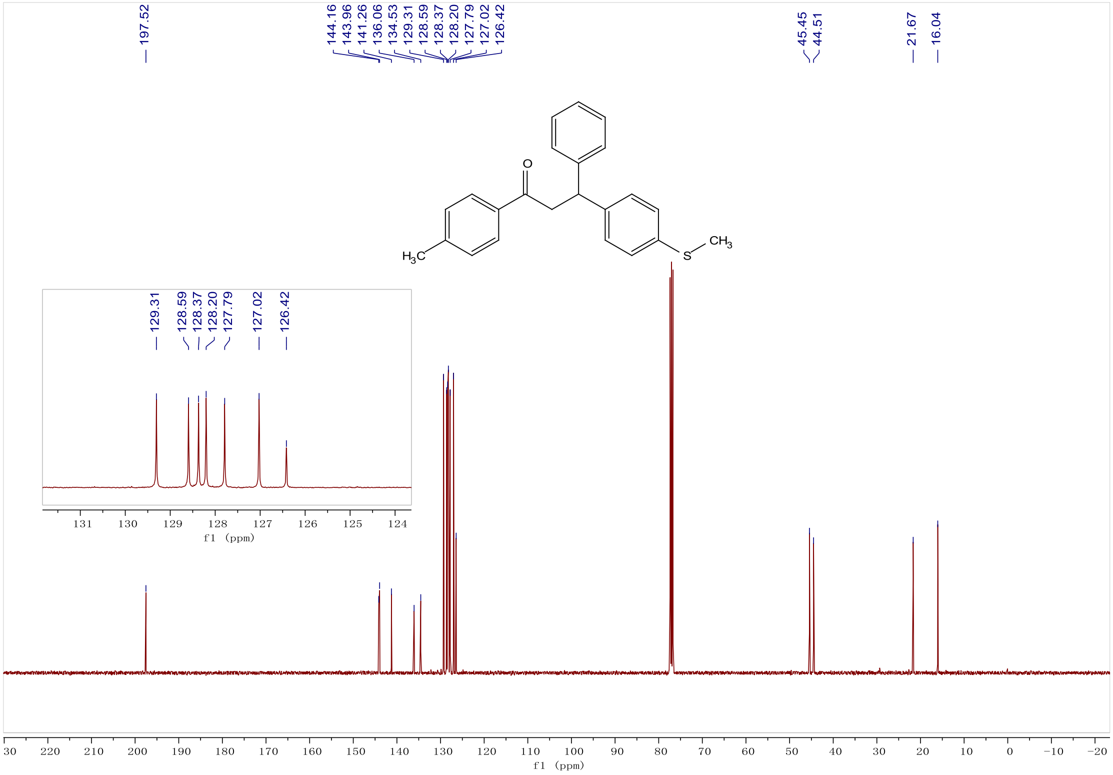

Supplementary Figure 72. ^{1}H and ^{13}C NMR spectra for compound **3eE**

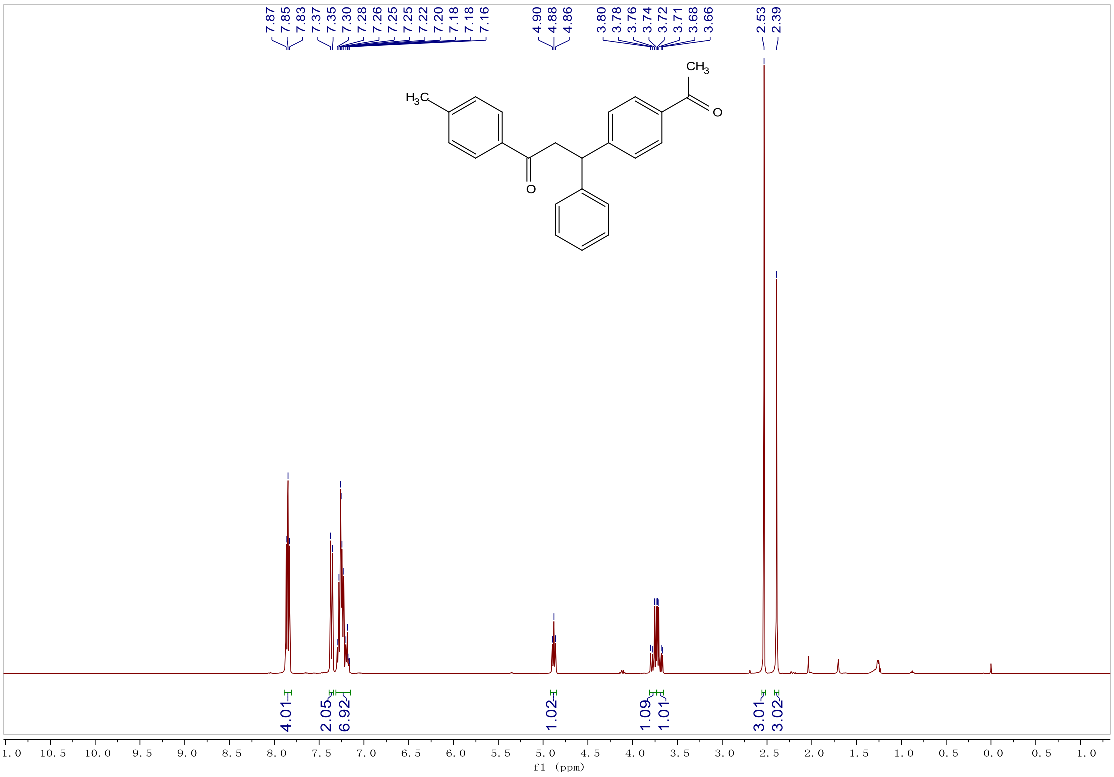

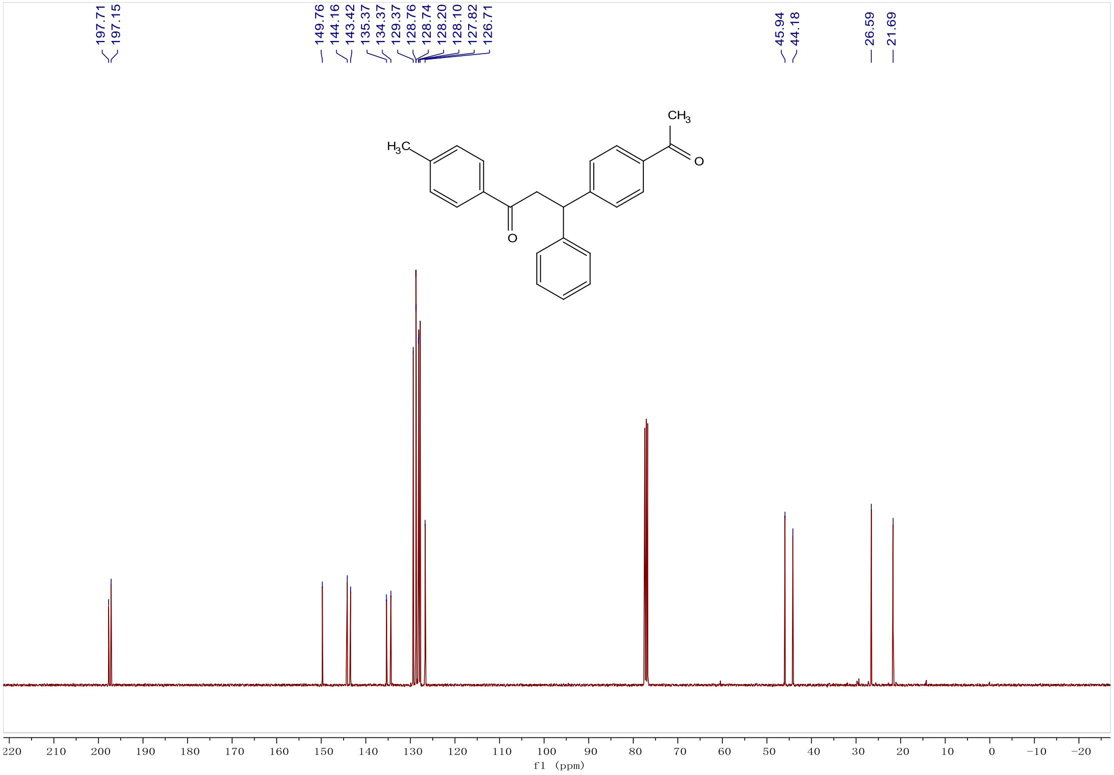

Supplementary Figure 73. ^{1}H and ^{13}C NMR spectra for compound **3fF**

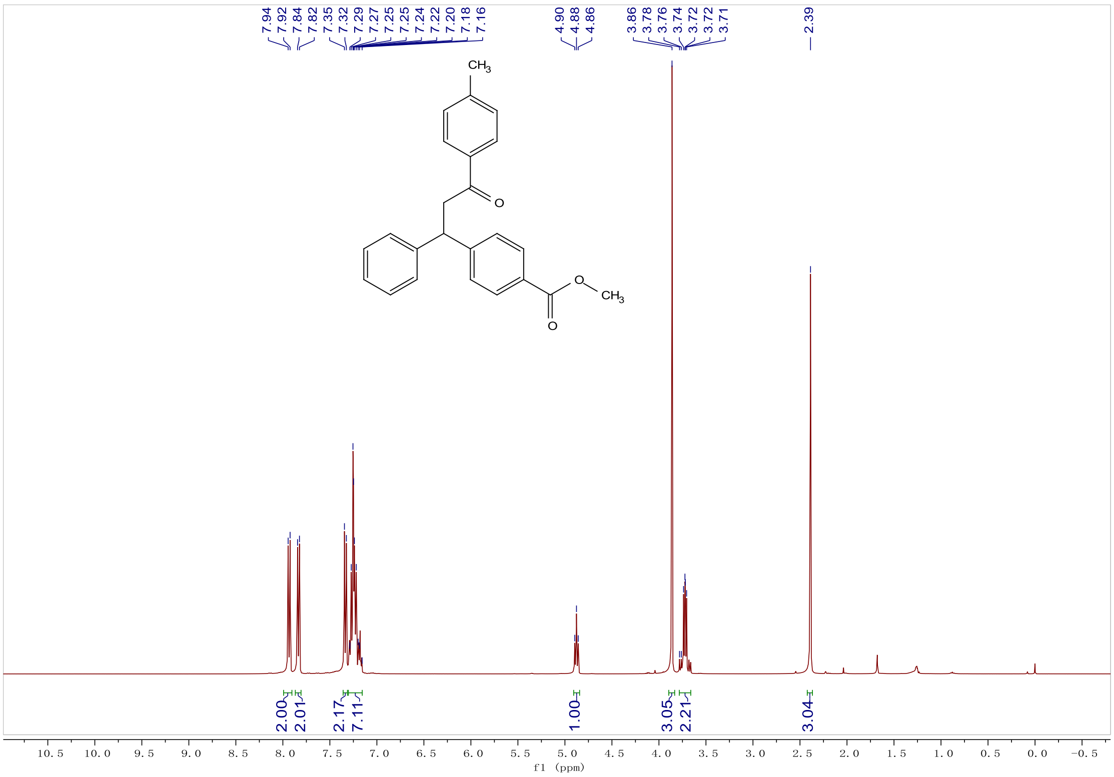

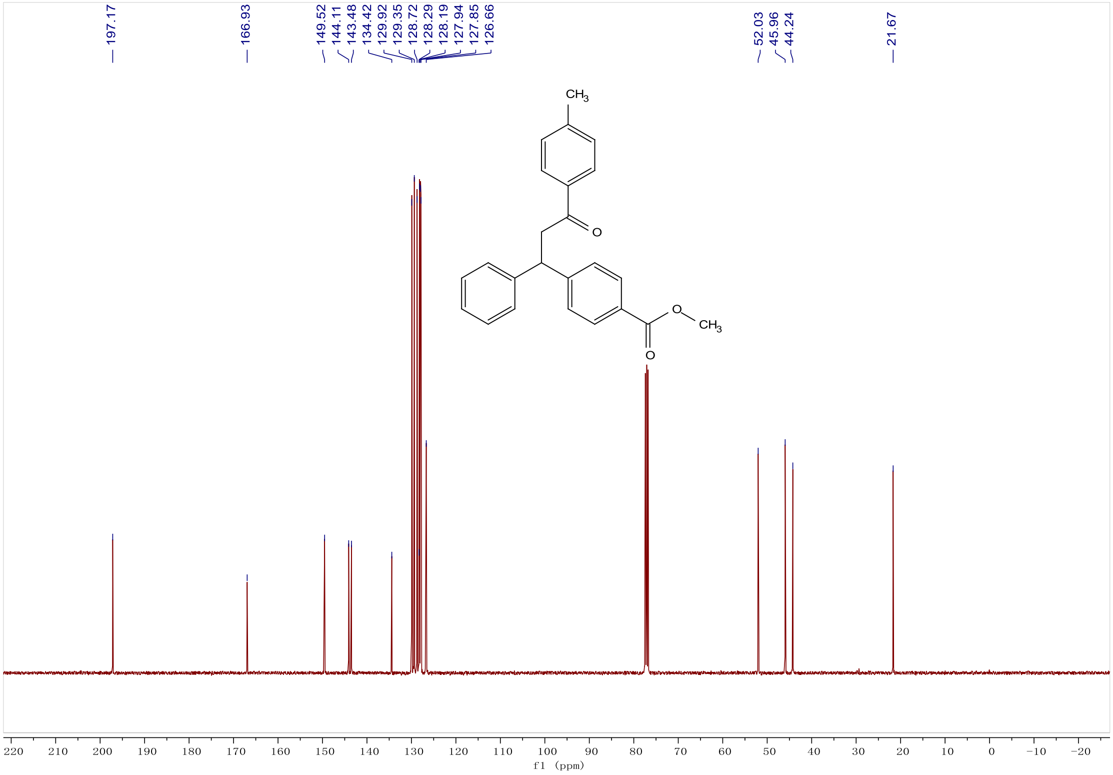

Supplementary Figure 74. ^{1}H and ^{13} C NMR spectra for compound **3gG**

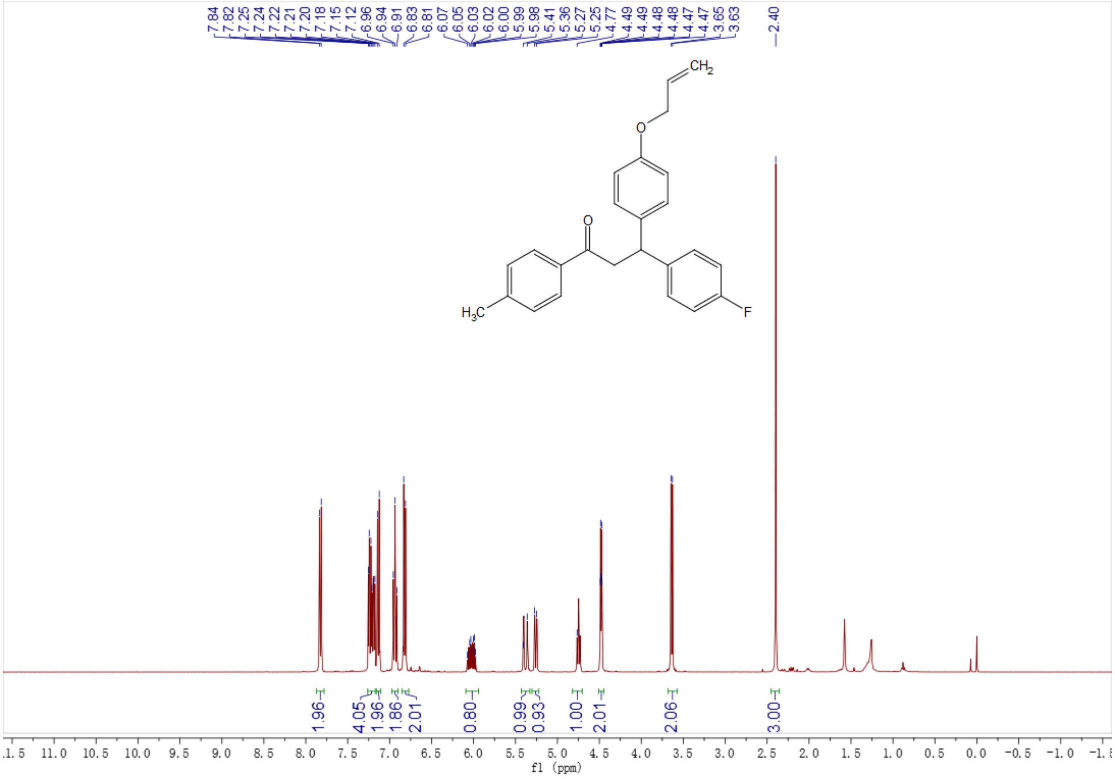

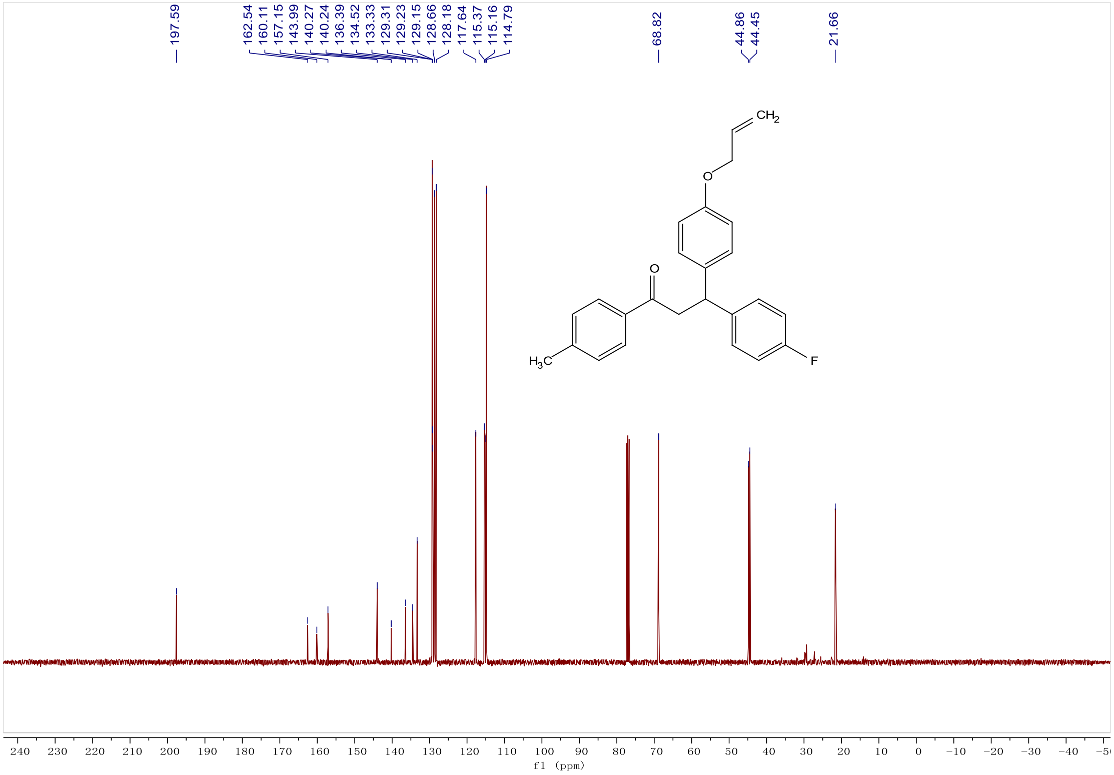

Supplementary Figure 75. ^{1}H and ^{13}C NMR spectra for compound **3hH**

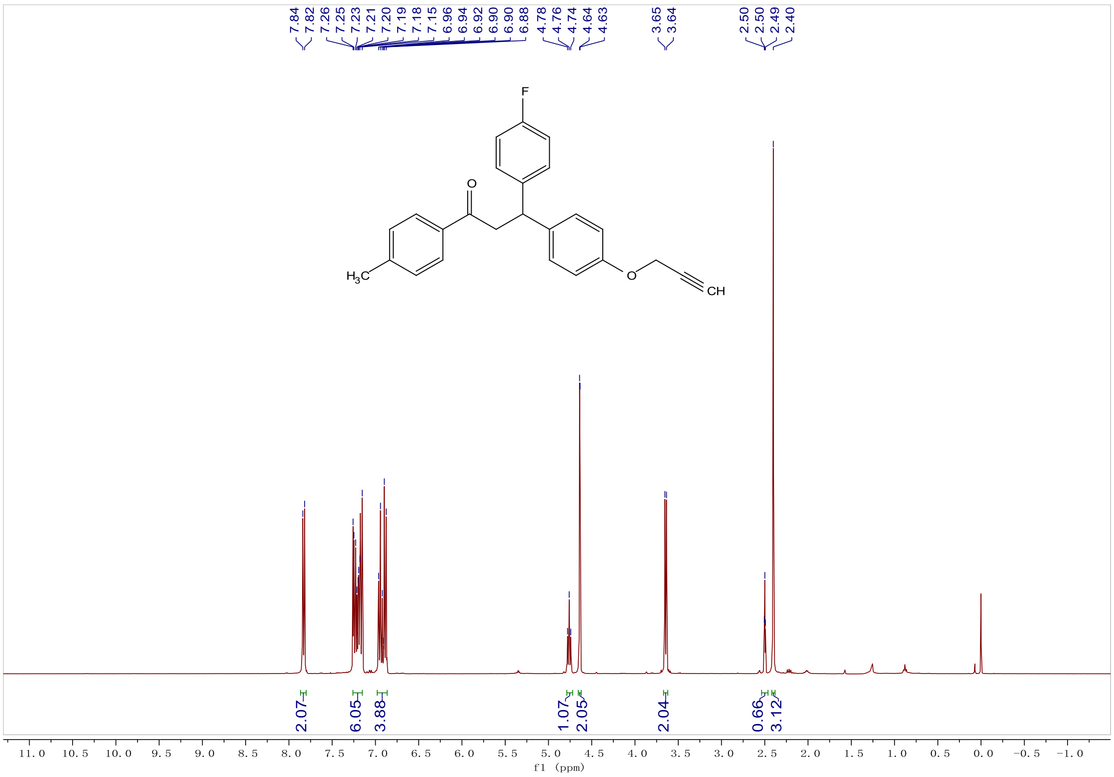

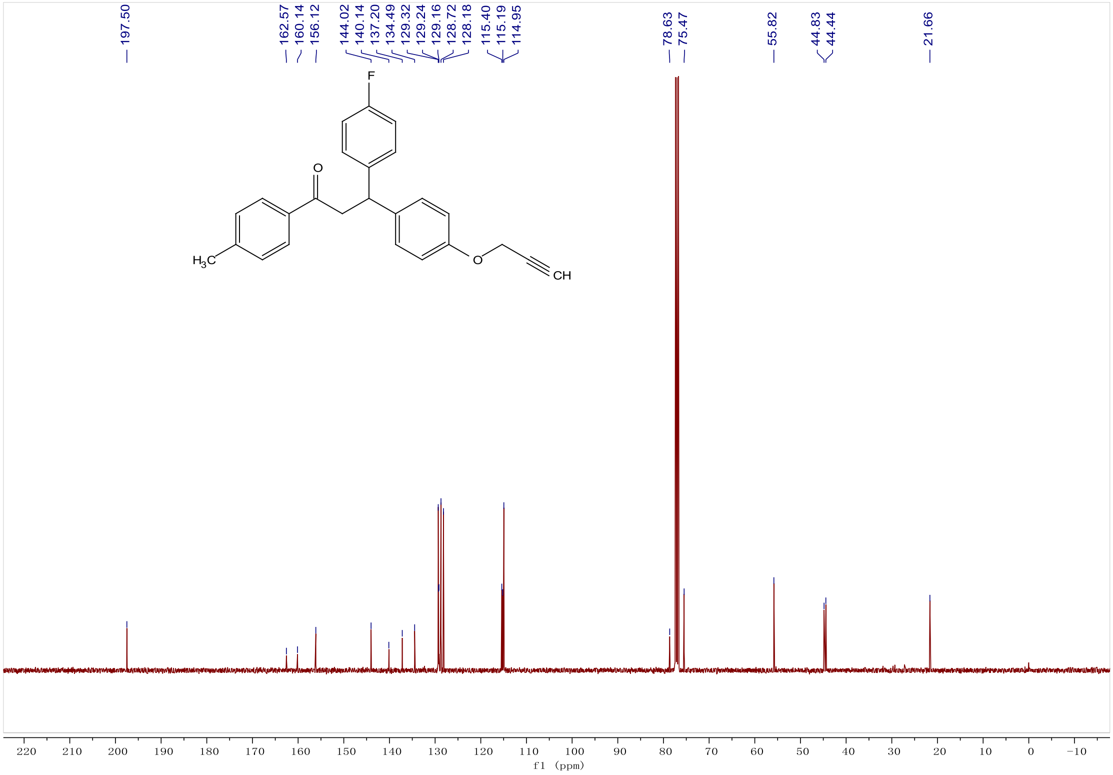

Supplementary Figure 76. ^{1}H and ^{13} C NMR spectra for compound **3iI**

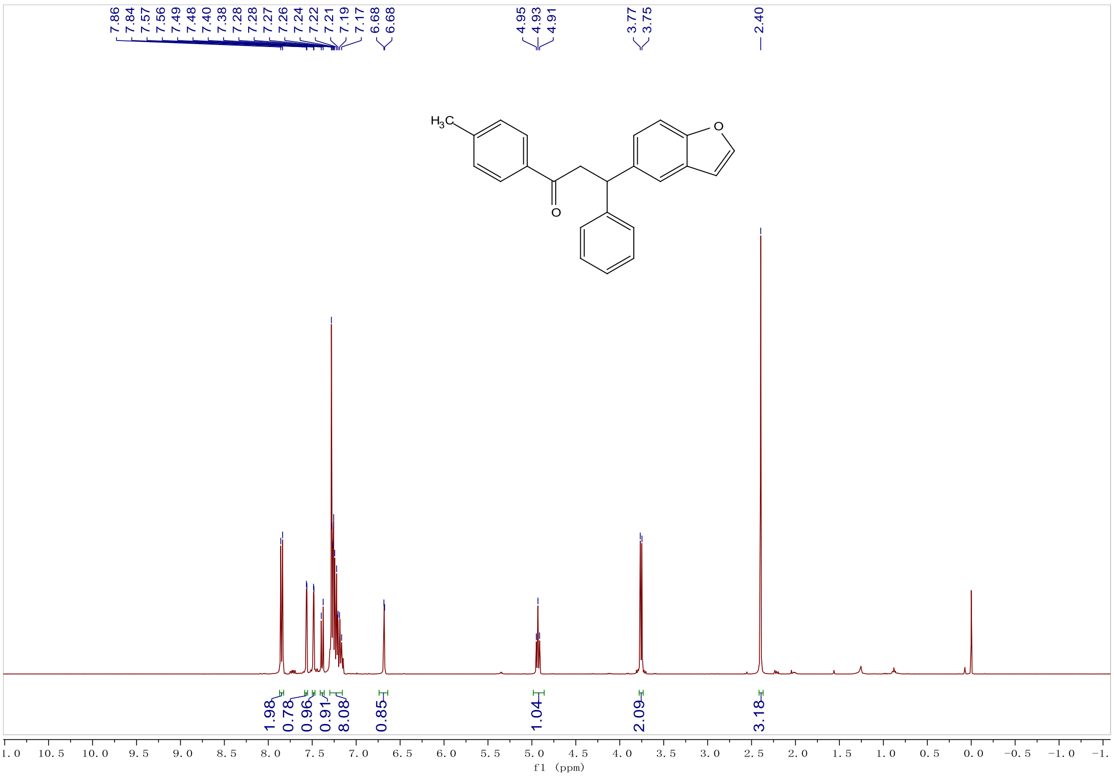

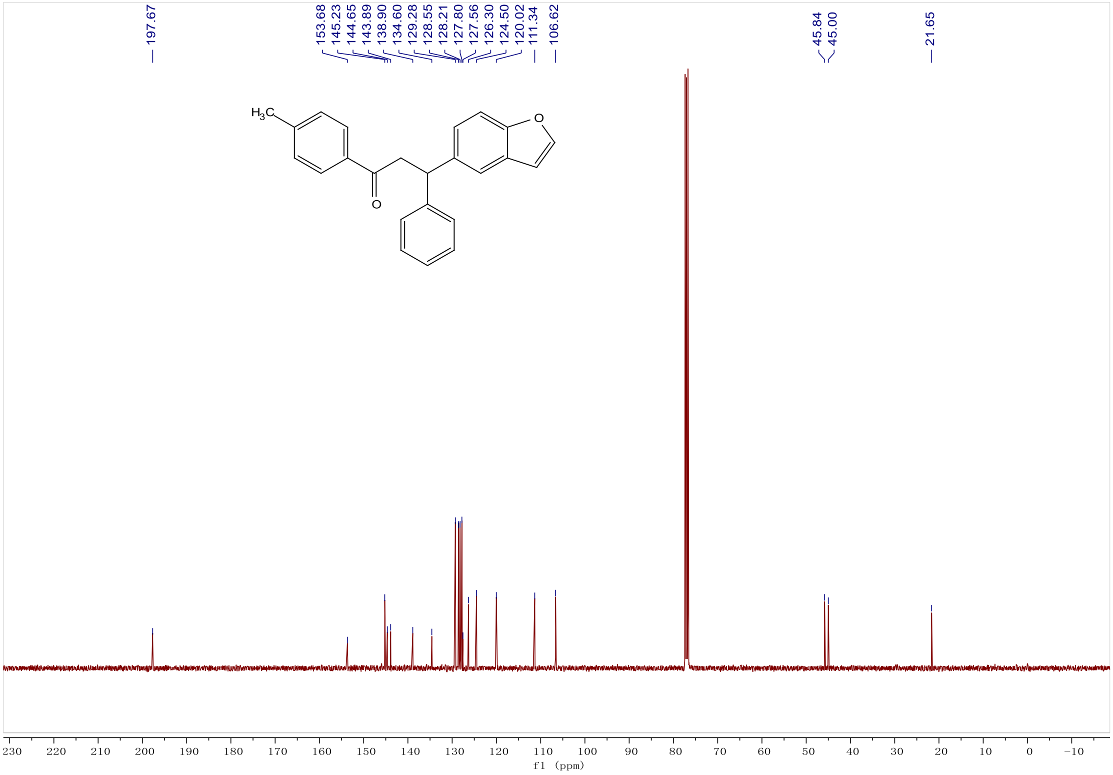

Supplementary Figure 77. ^{1}H and ^{13} C NMR spectra for compound **3jJ**

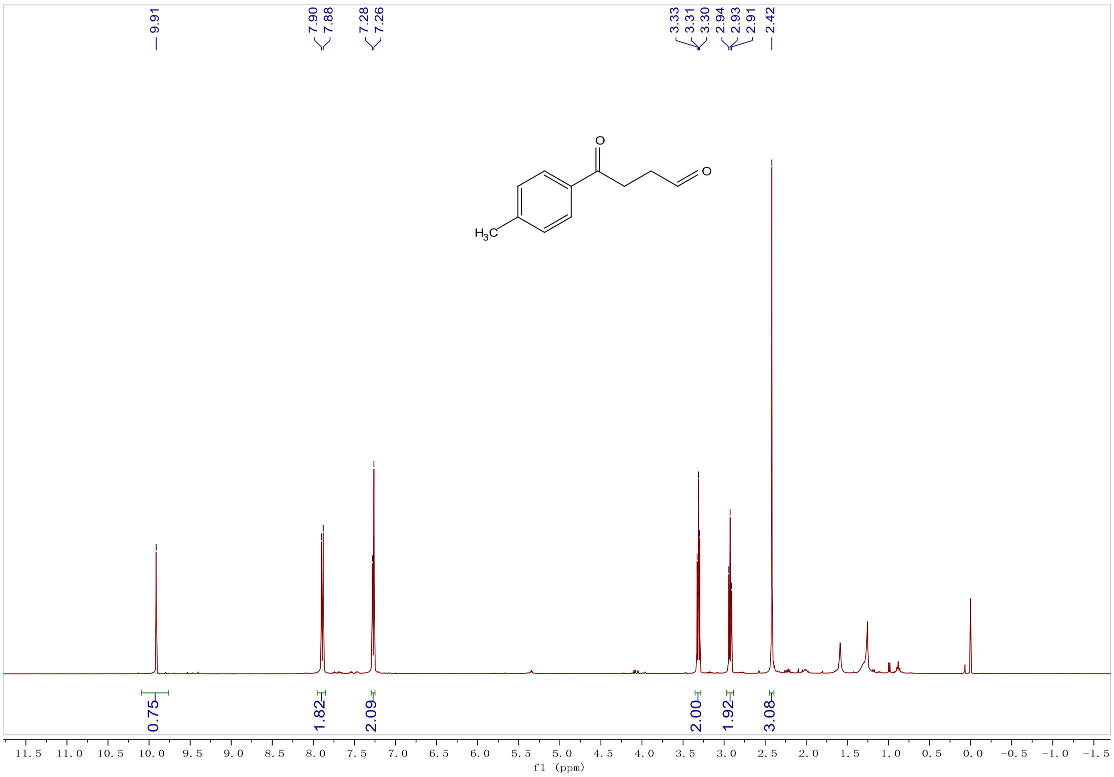

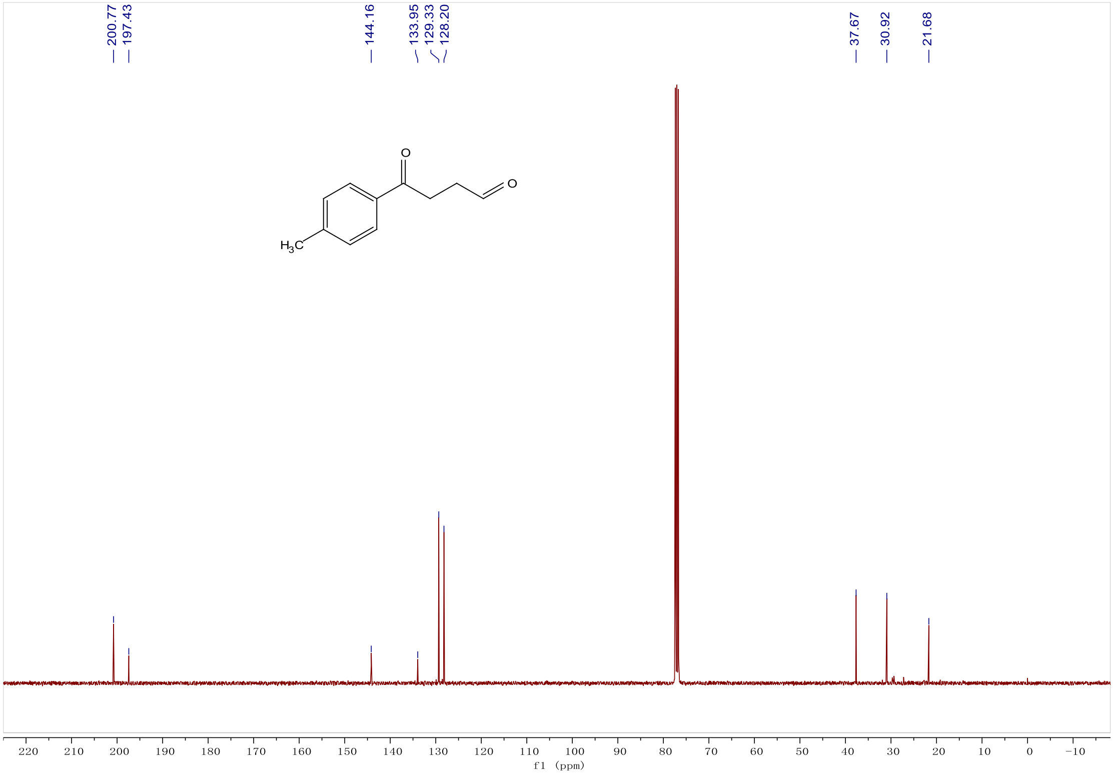

Supplementary Figure 78. ^{1}H and ^{13}C NMR spectra for compound **3kK**

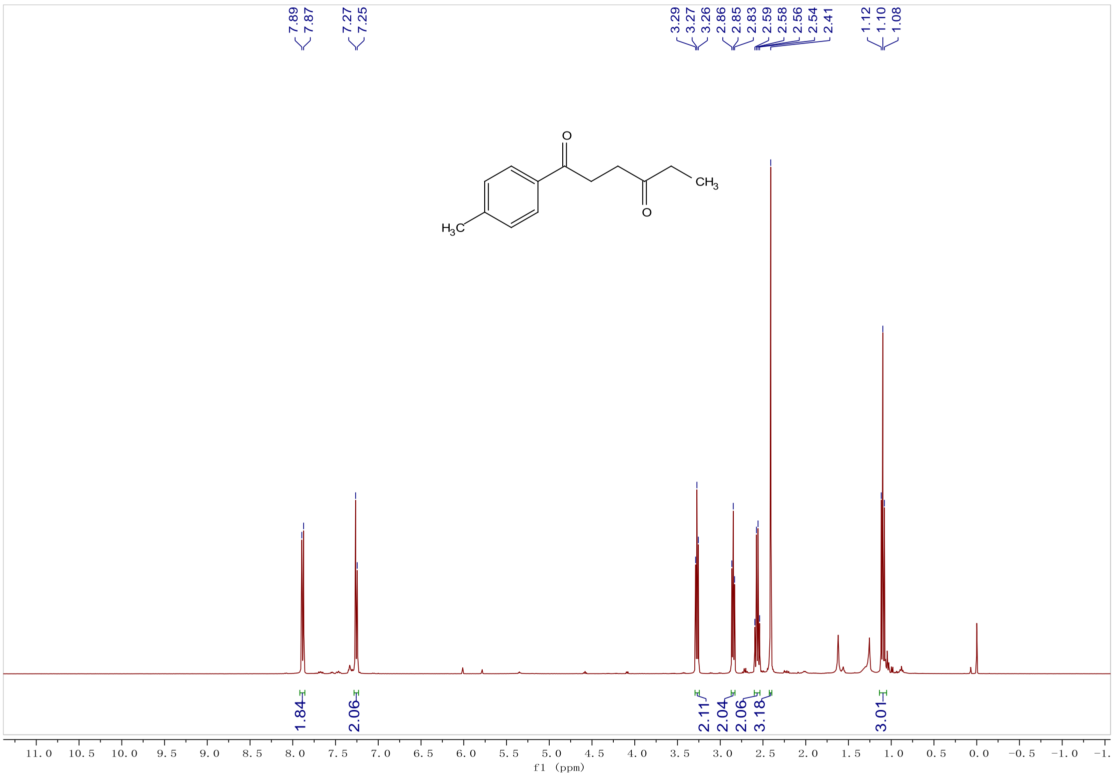

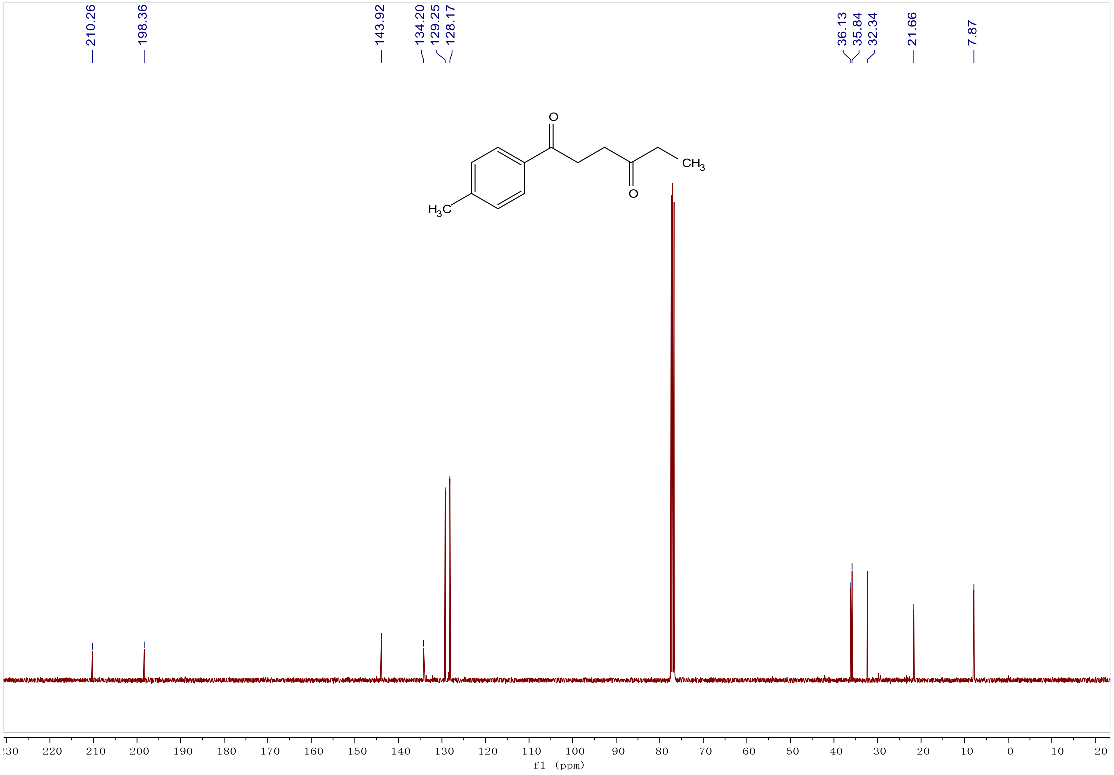

Supplementary Figure 79. ^{1}H and ^{13}C NMR spectra for compound **3lL**

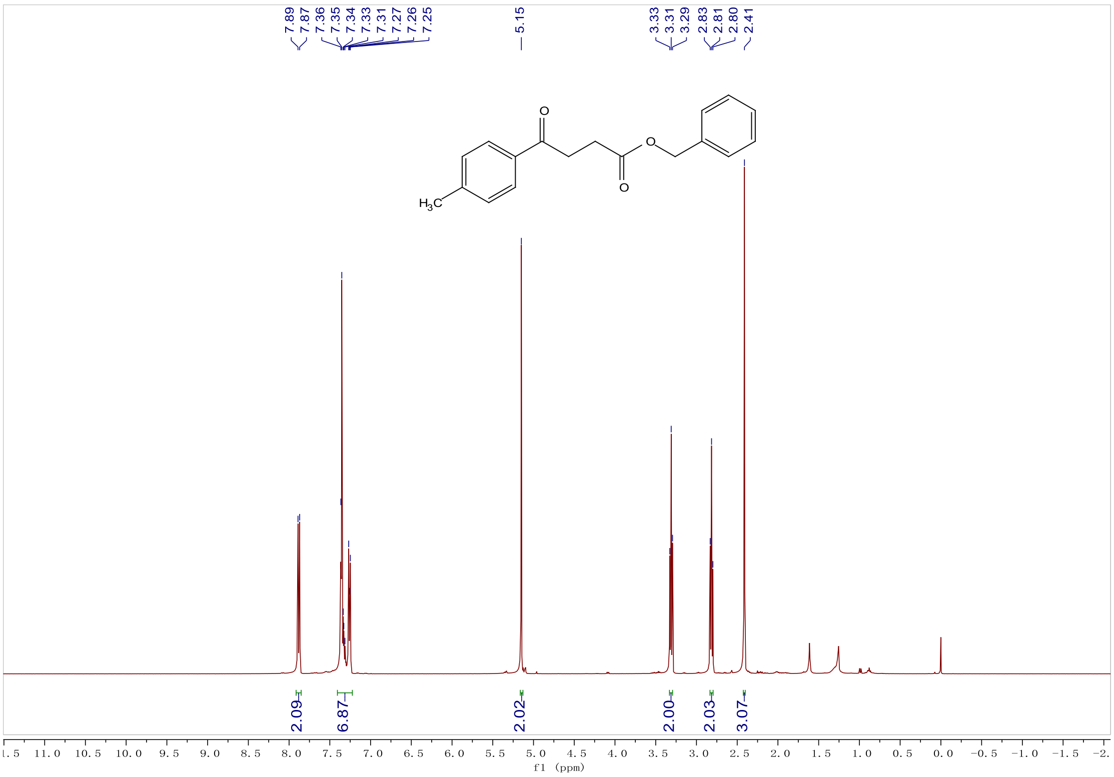

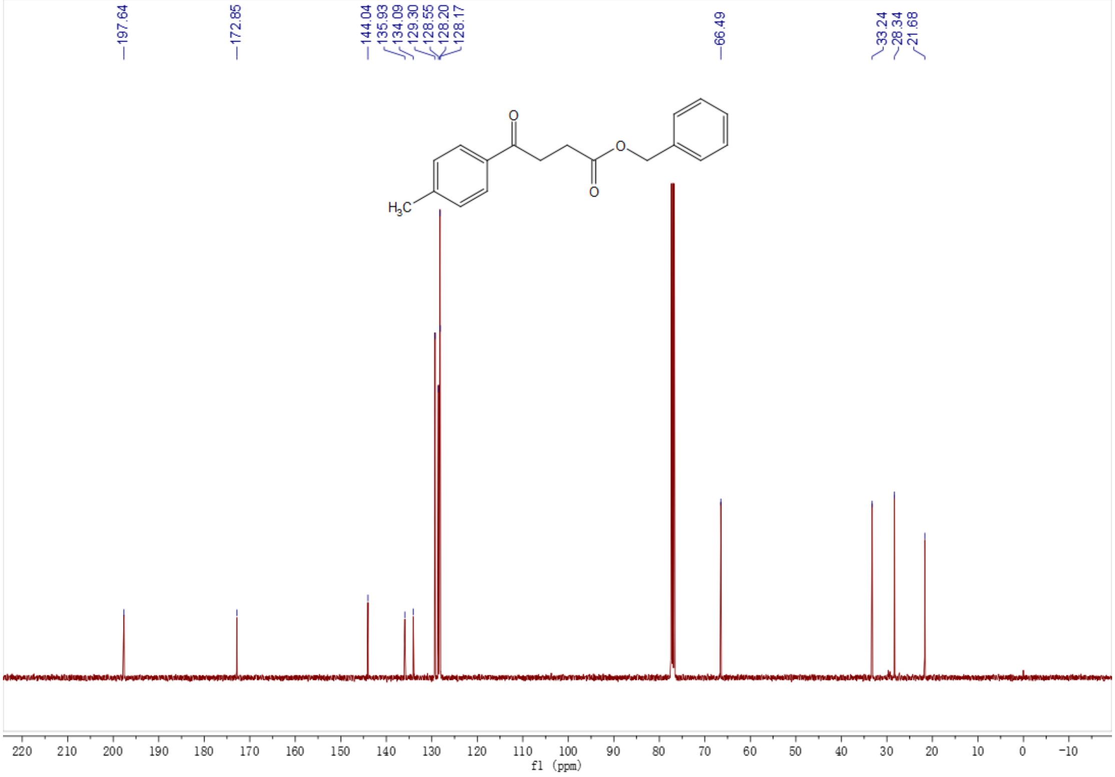

Supplementary Figure 80. ^{1}H and ^{13}C NMR spectra for compound **3mM**

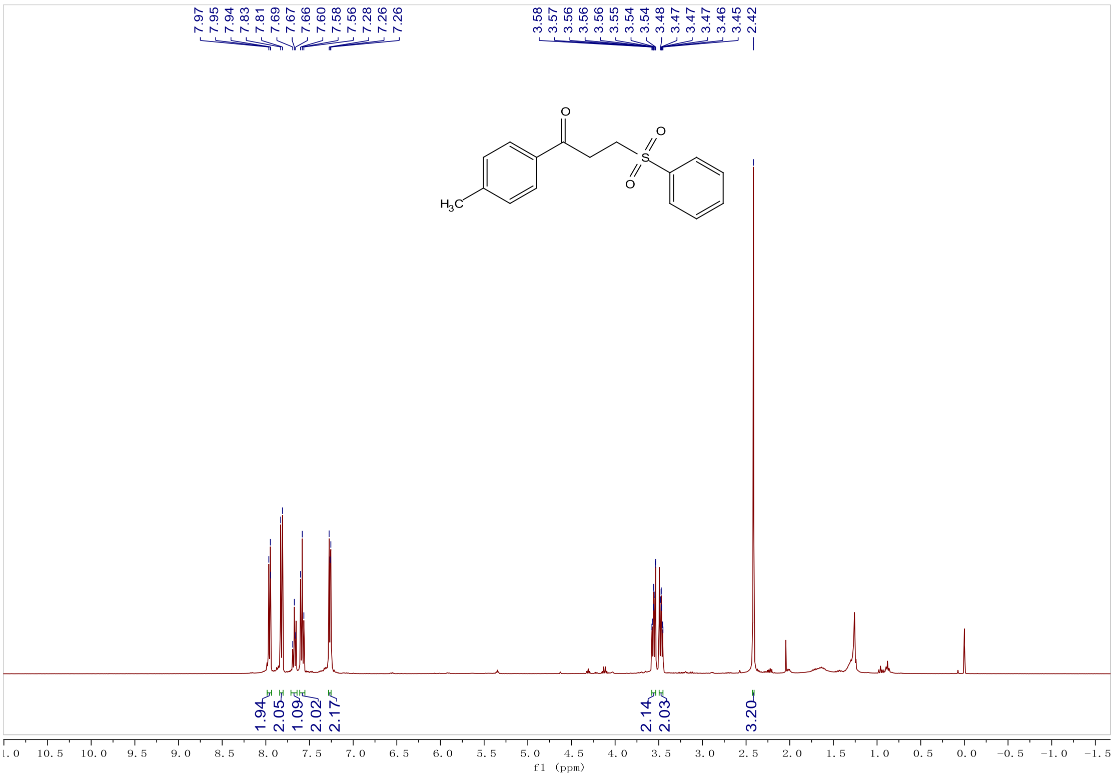

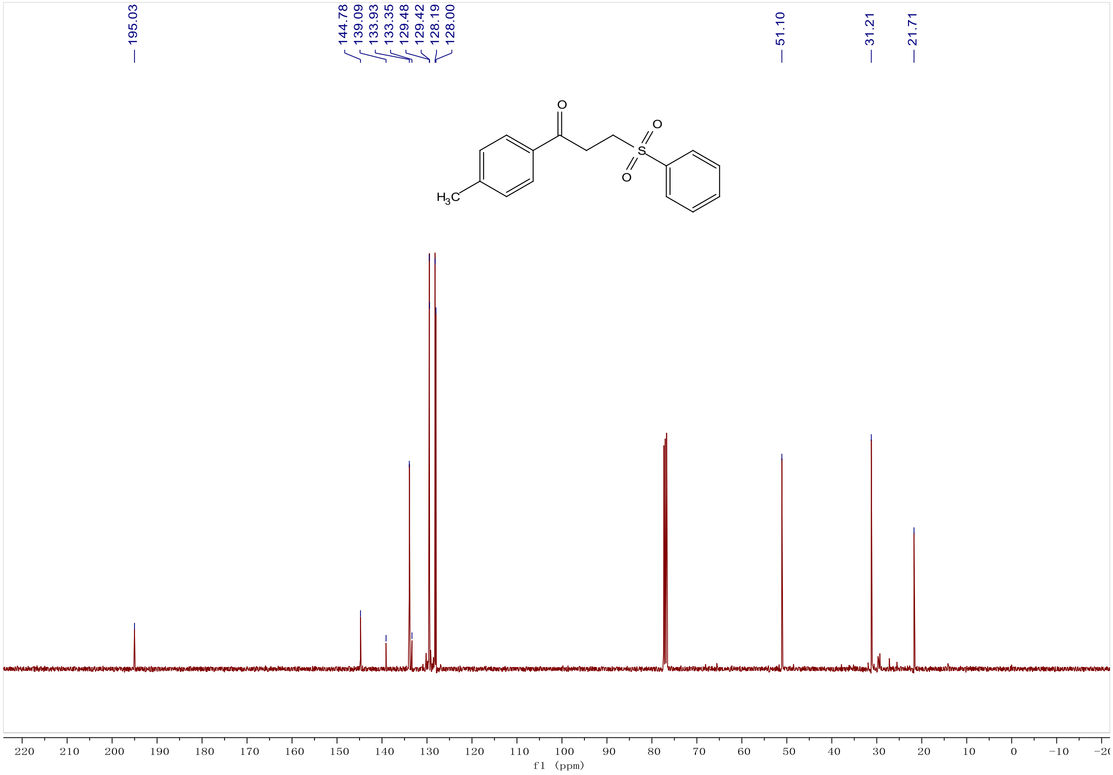

Supplementary Figure 81. ^{1}H and ^{13} C NMR spectra for compound **3nN**

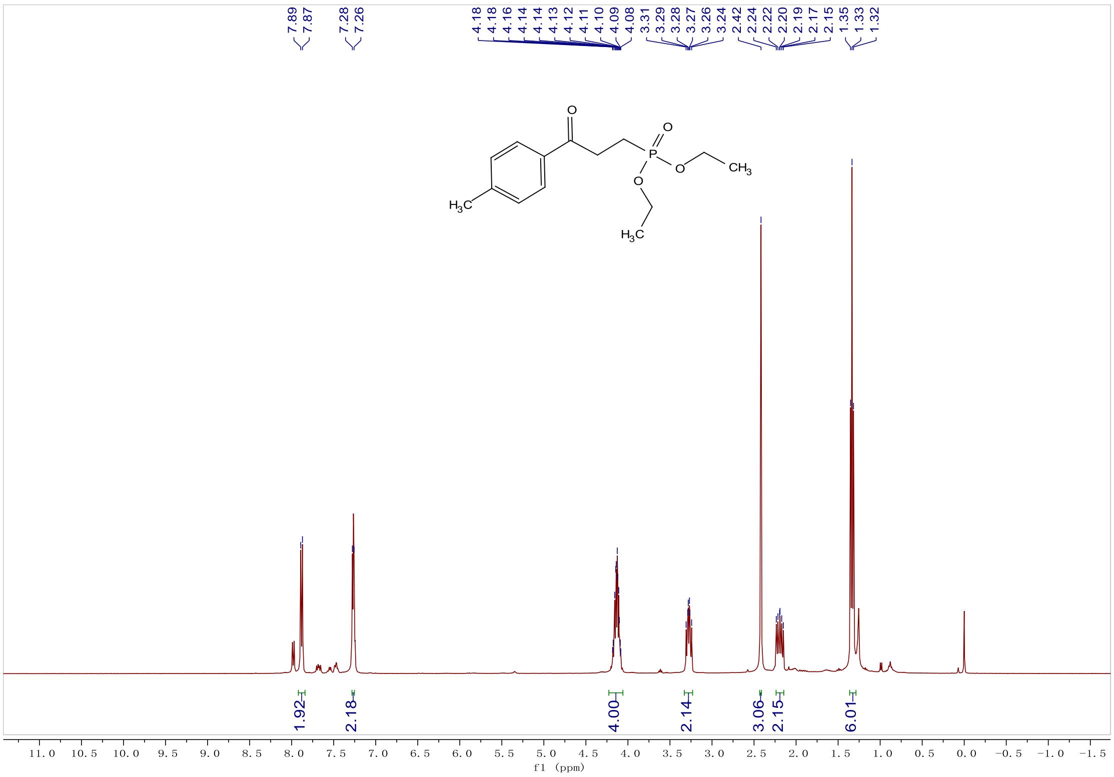

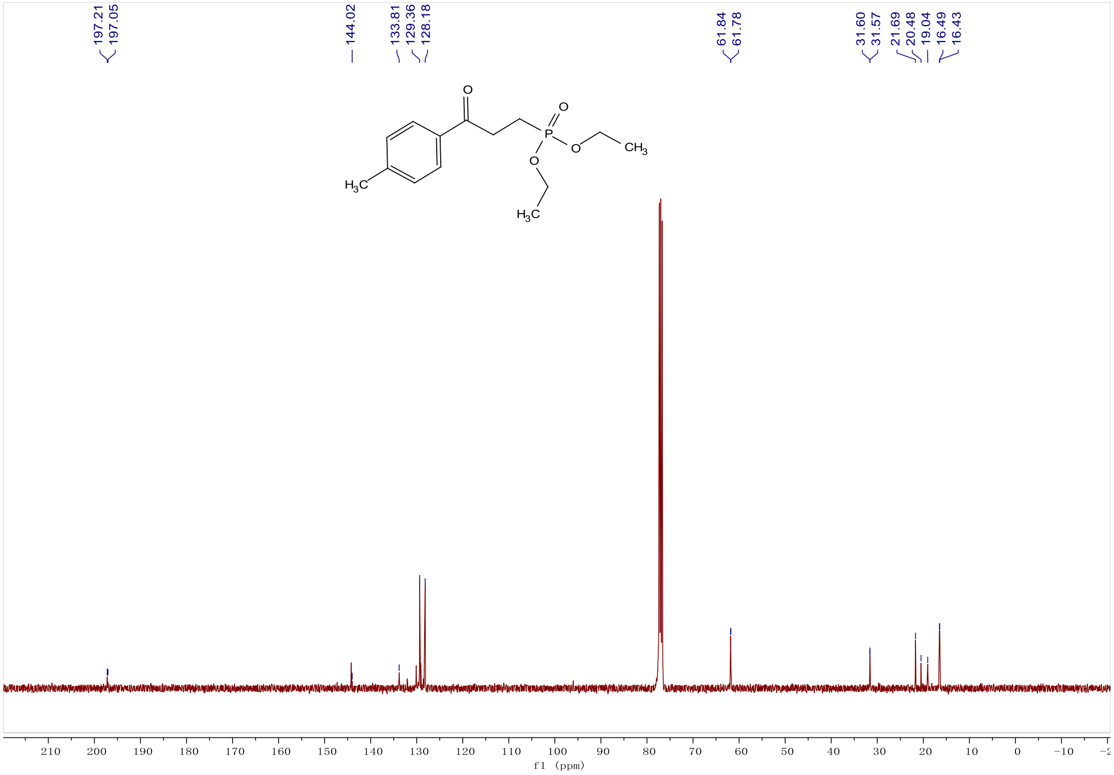

Supplementary Figure 82. ^{1}H and ^{13} C NMR spectra for compound **3oO**

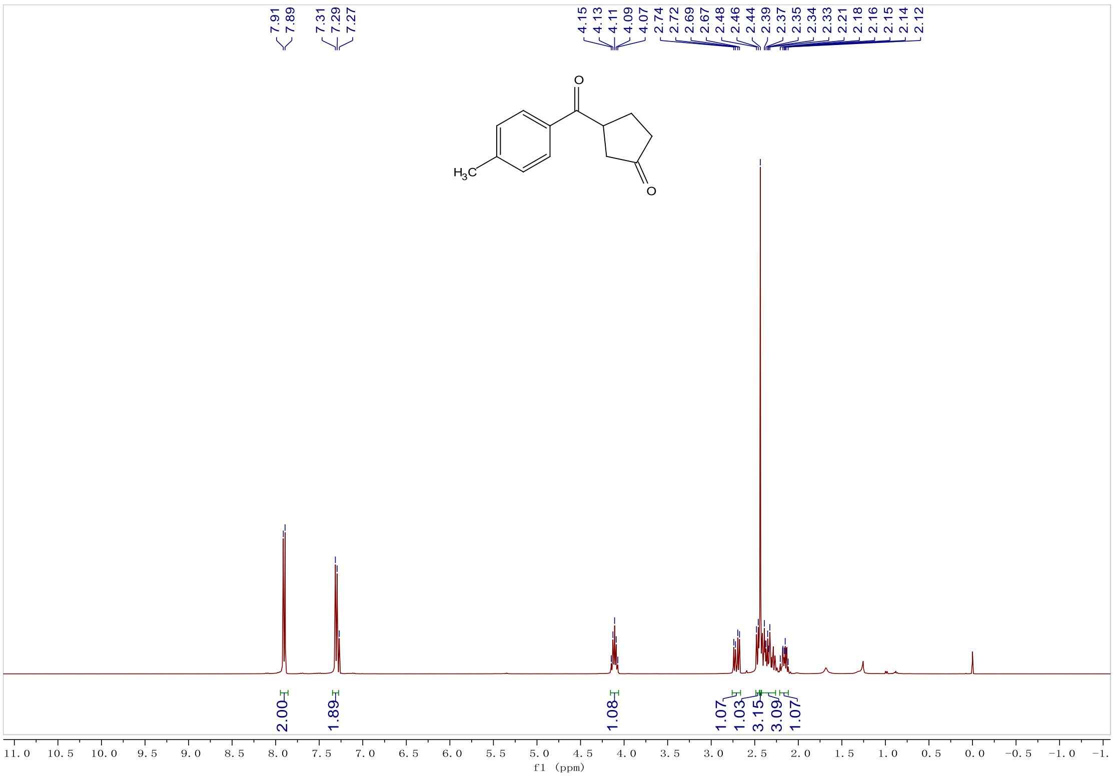

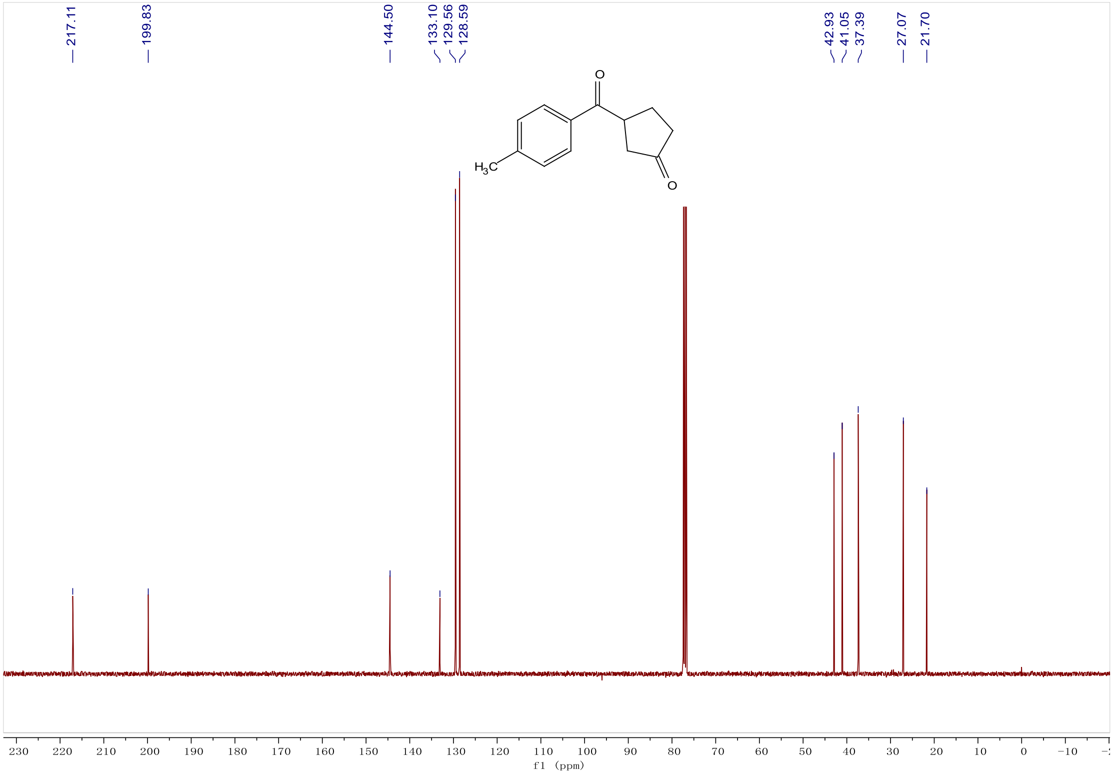

Supplementary Figure 83. ^{1}H and ^{13}C NMR spectra for compound **3pP**

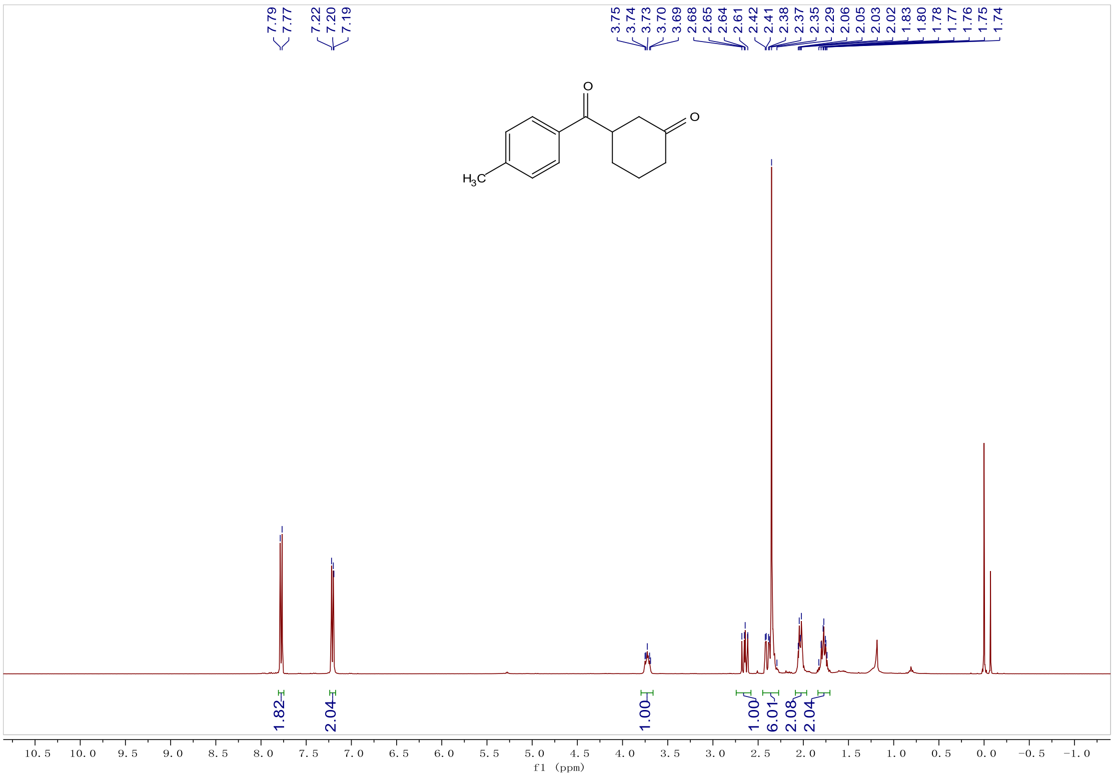

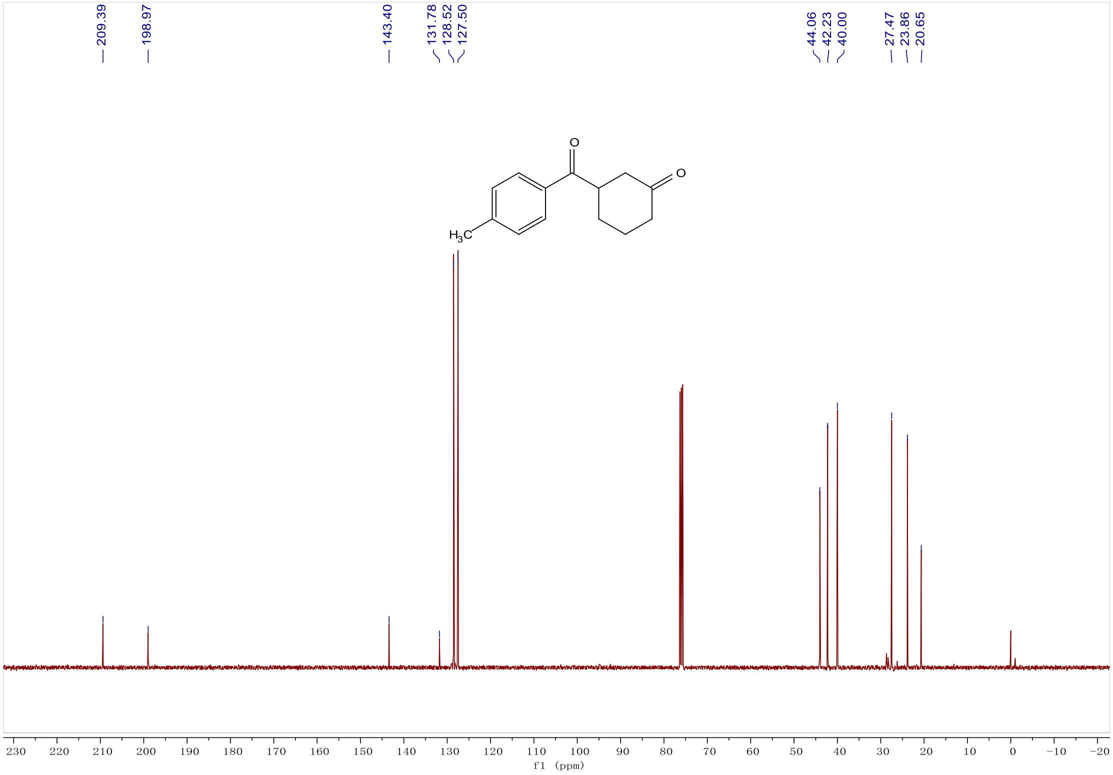

Supplementary Figure 84. ^{1}H and ^{13} C NMR spectra for compound **3qQ**

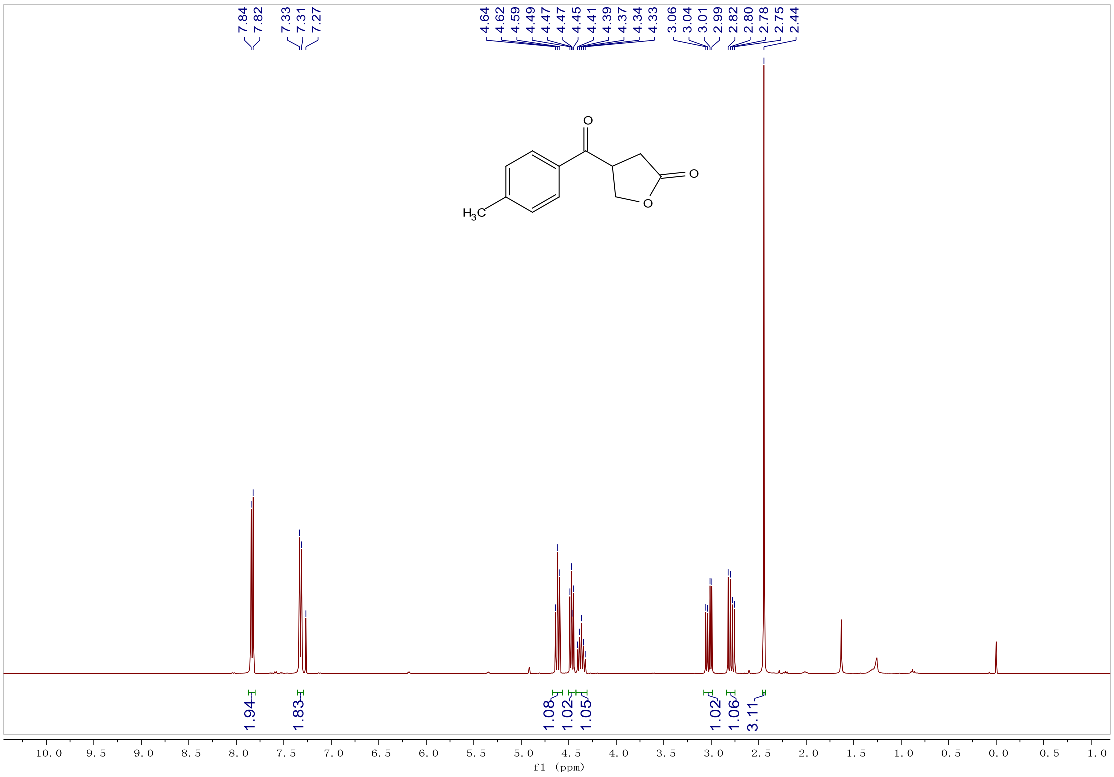

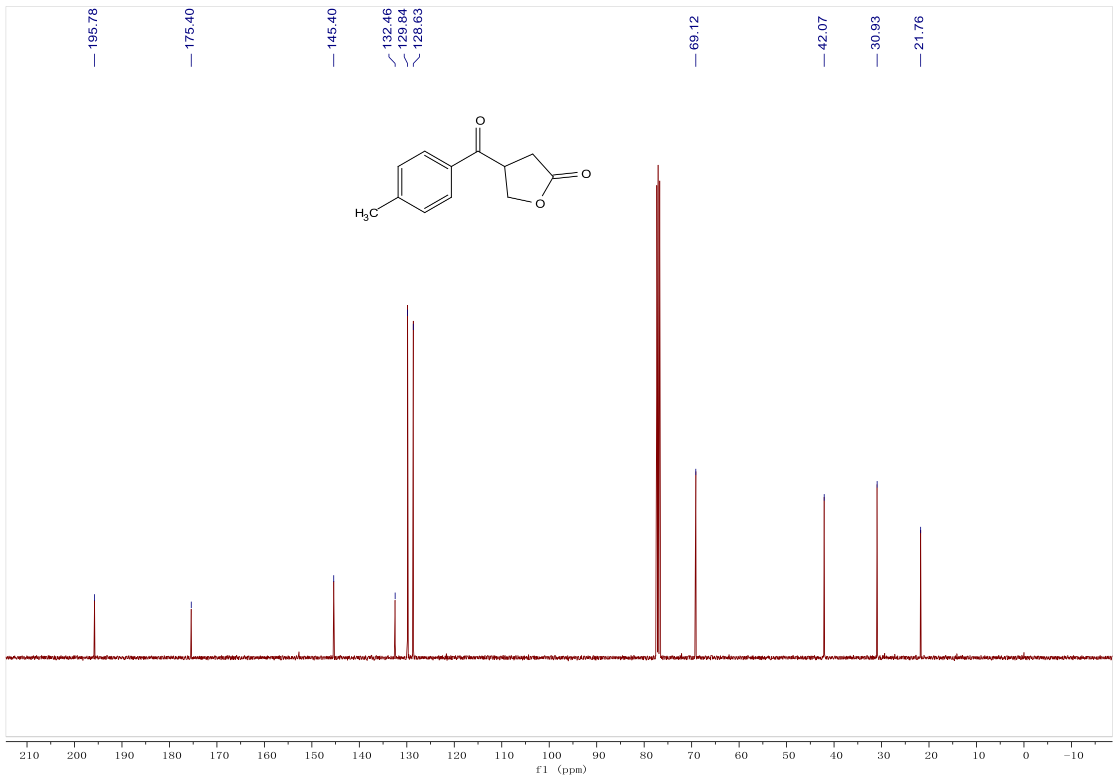

Supplementary Figure 85. ^{1}H and ^{13}C NMR spectra for compound **3rR**

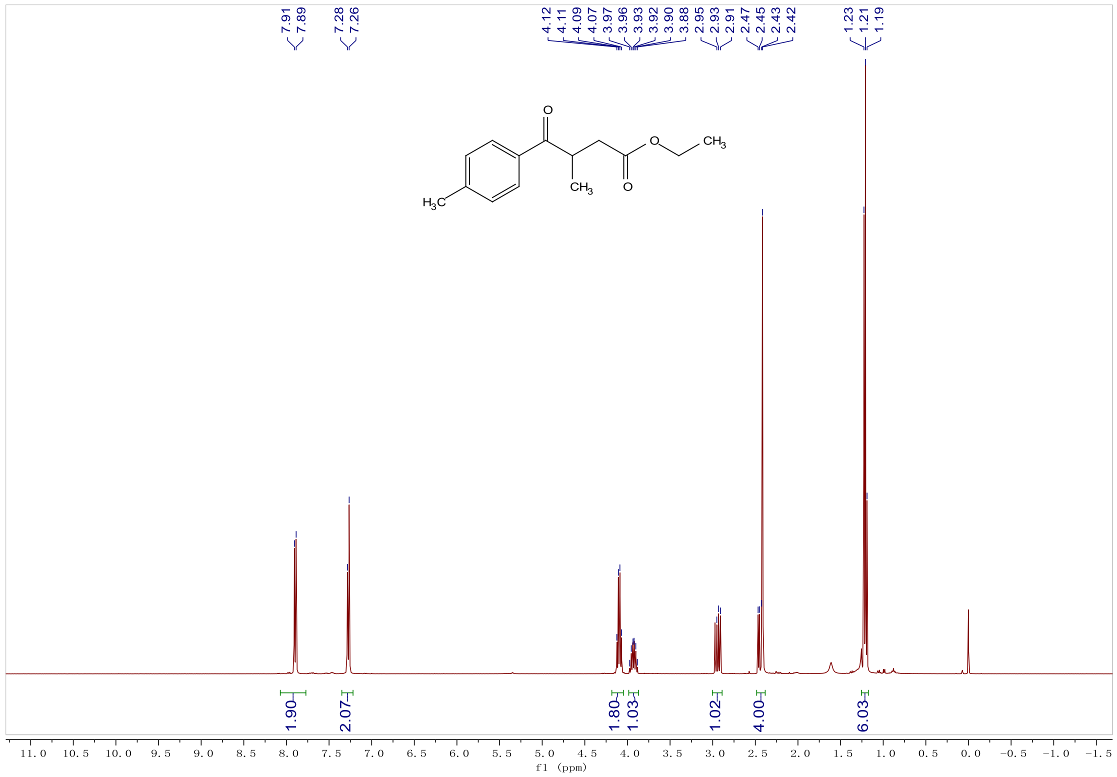

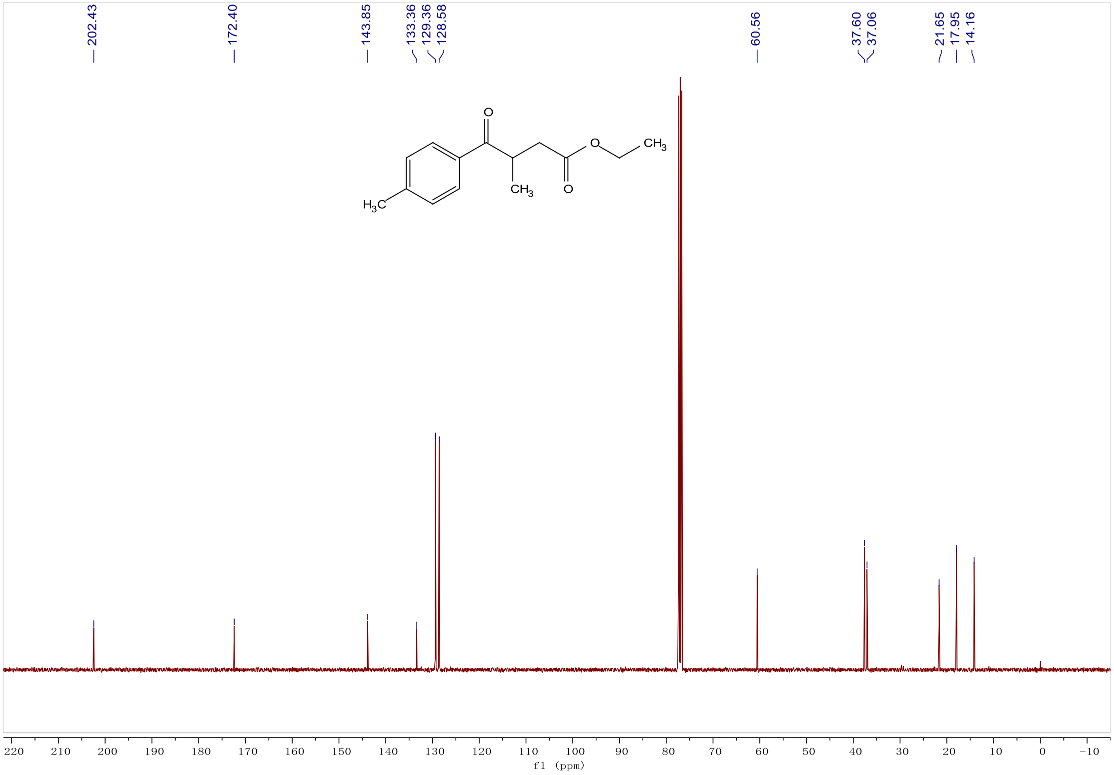

Supplementary Figure 86. ^{1}H and ^{13}C NMR spectra for compound **3sS**

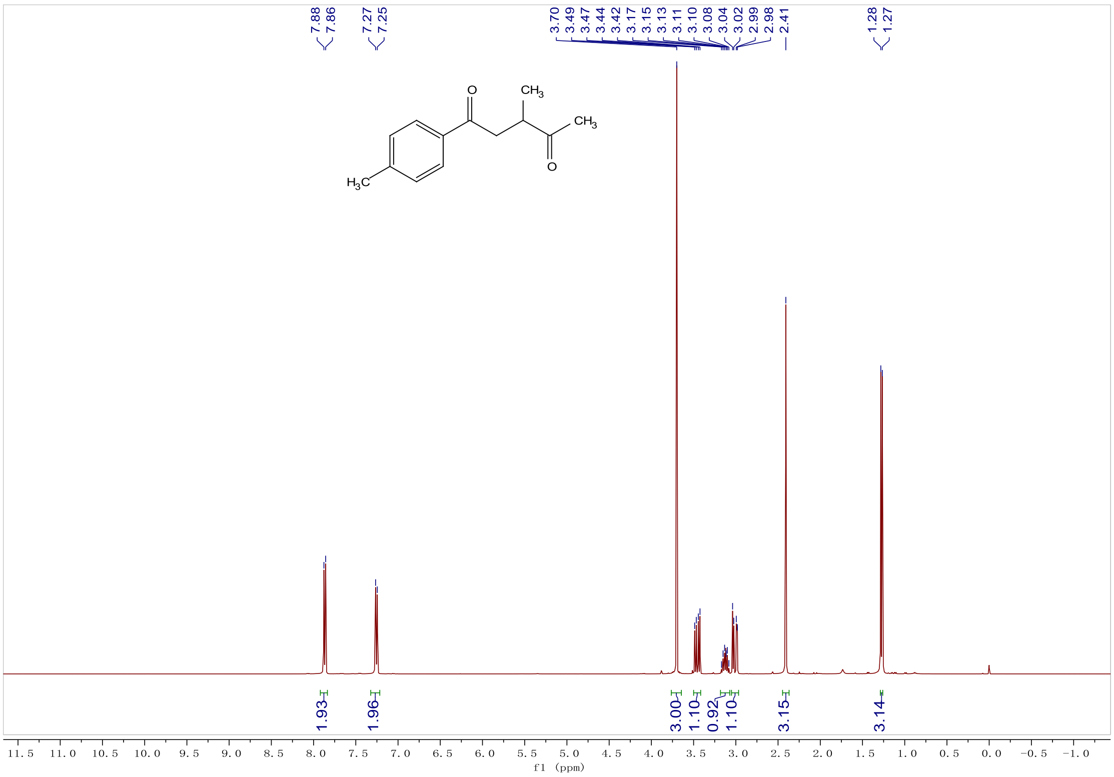

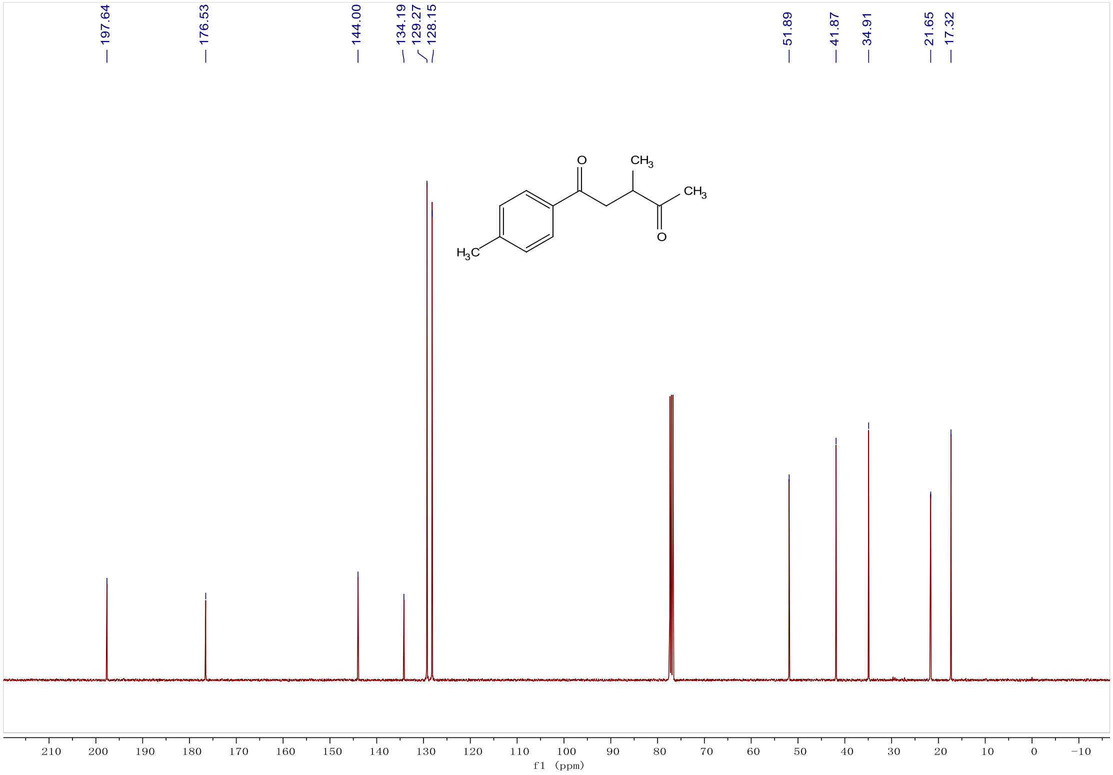

Supplementary Figure 87. ^{1}H and ^{13} C NMR spectra for compound **3tT**

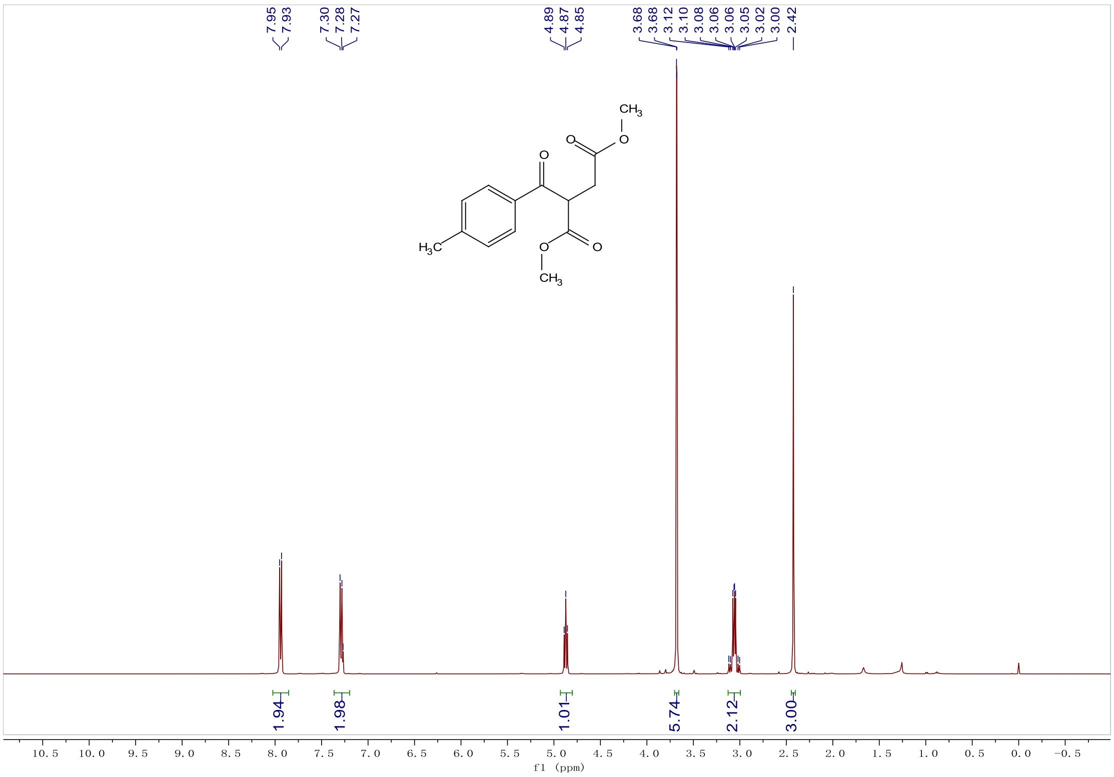

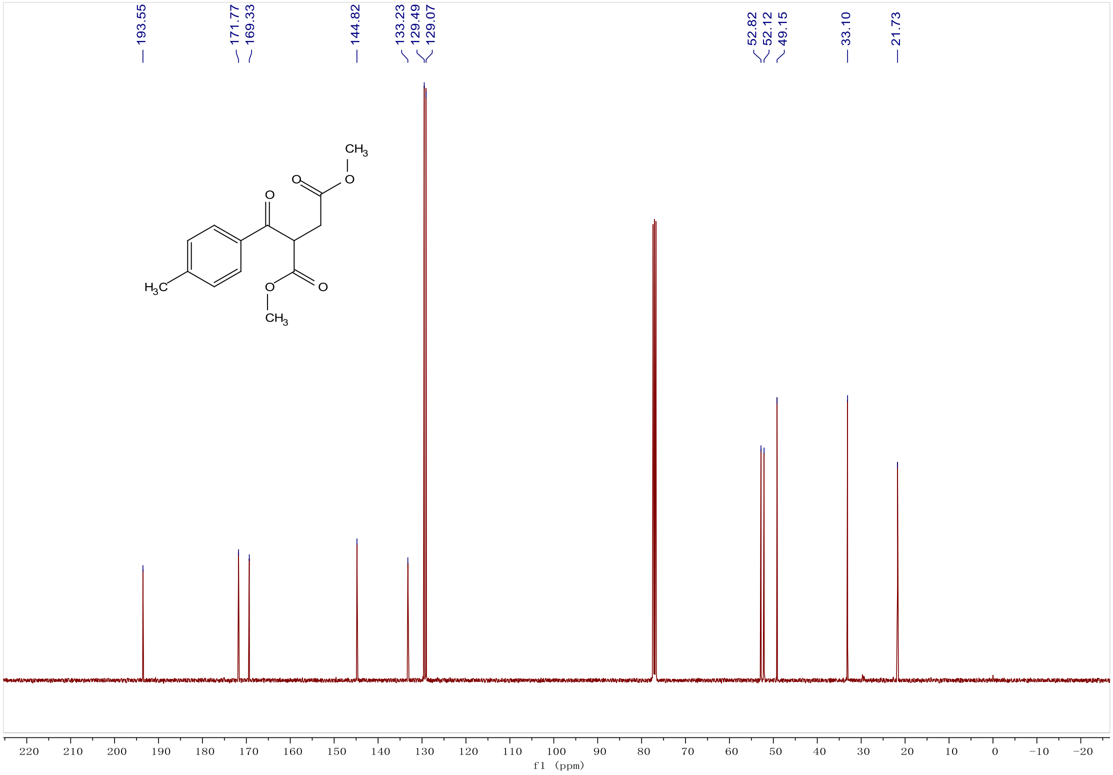

Supplementary Figure 88. ^{1}H and ^{13}C NMR spectra for compound **3uU**

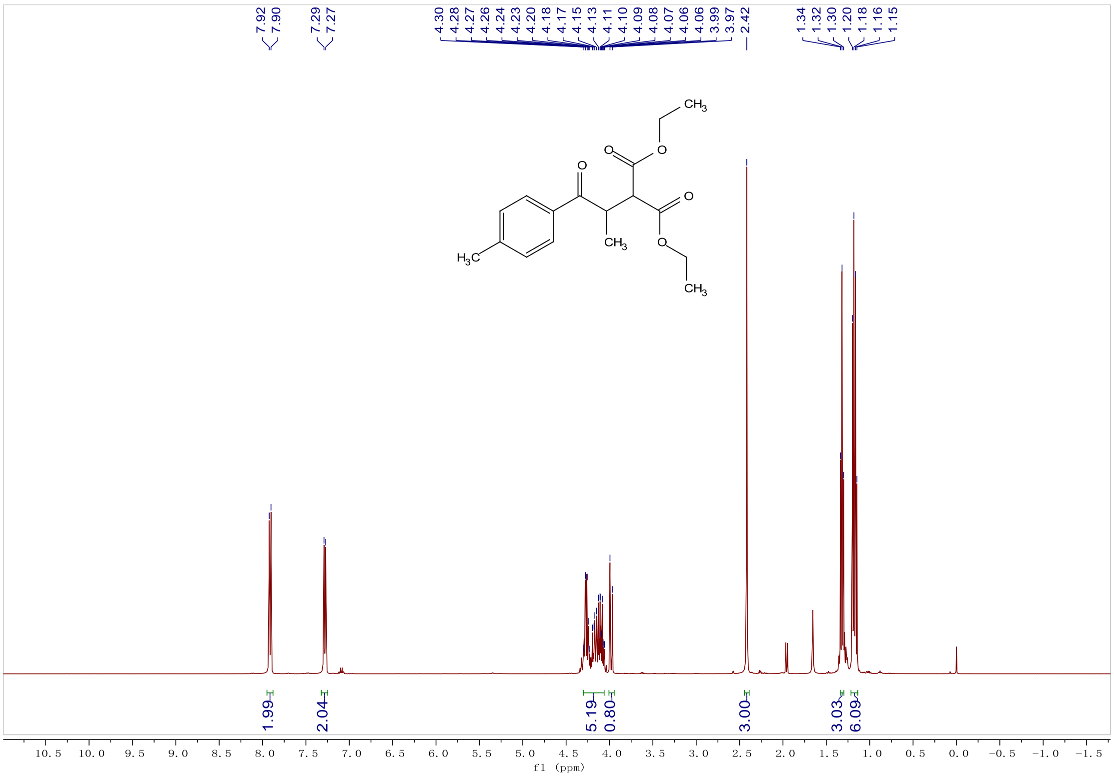

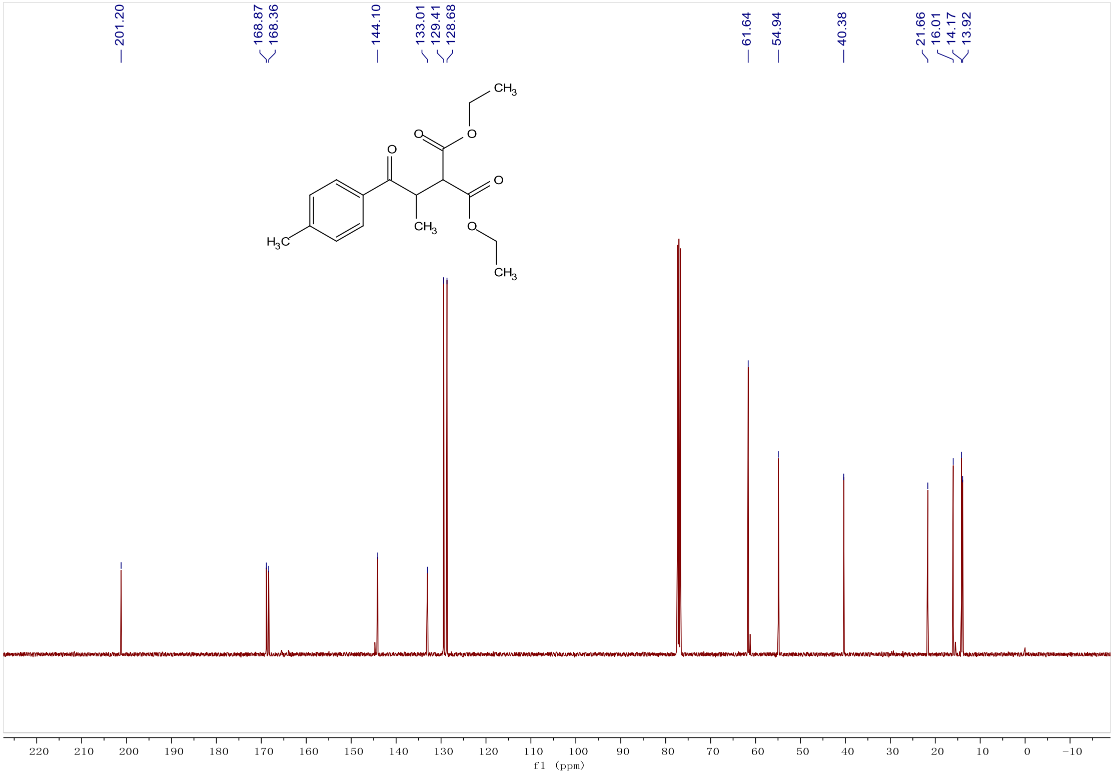

Supplementary Figure 89. ^{1}H and ^{13}C NMR spectra for compound **3vV**

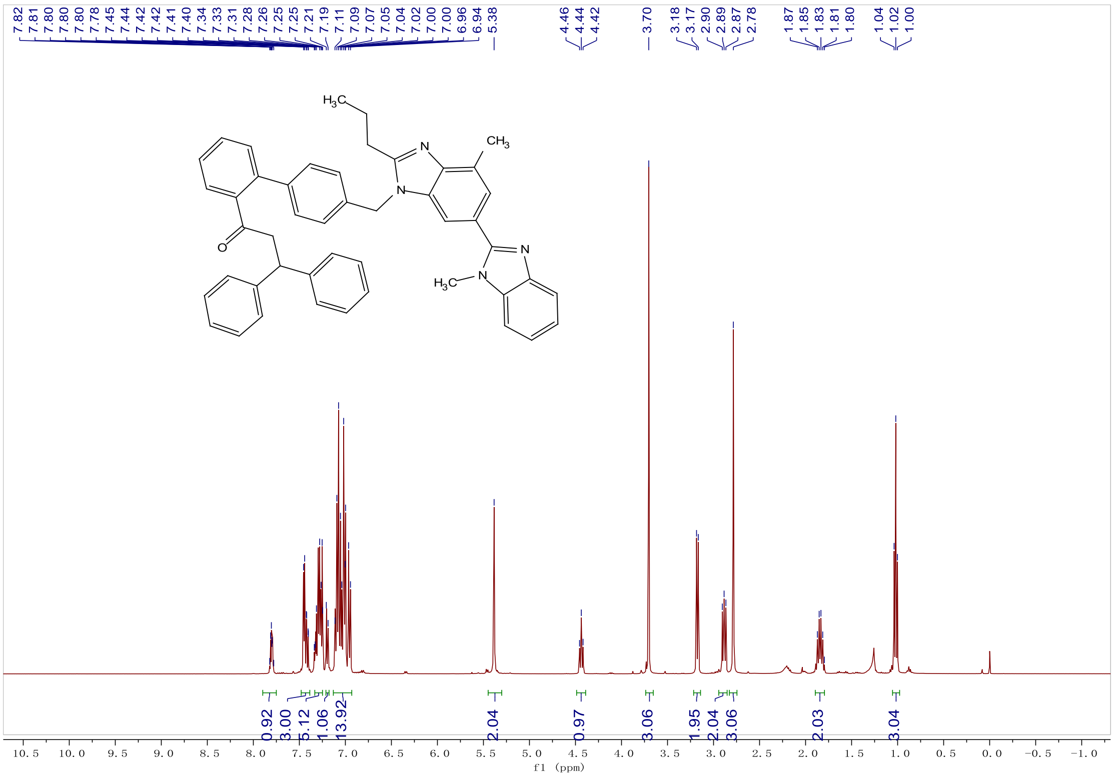

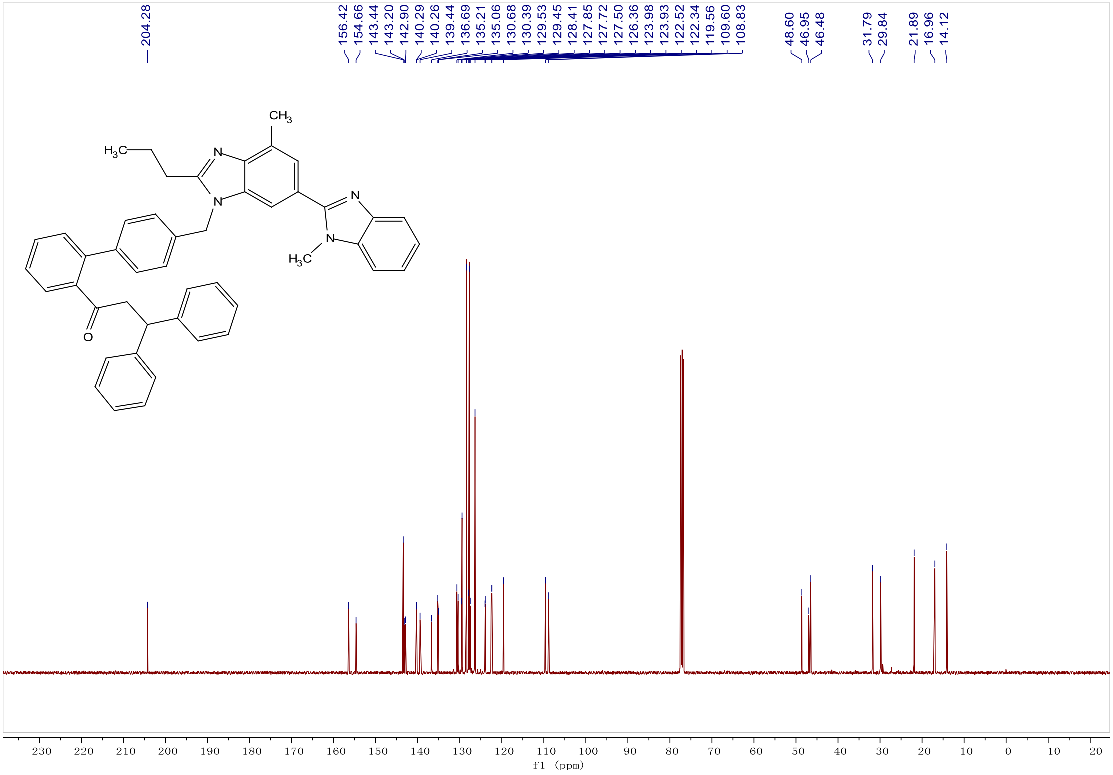

Supplementary Figure 90. ^{1}H and ^{13} C NMR spectra for compound Telmisartan **4**

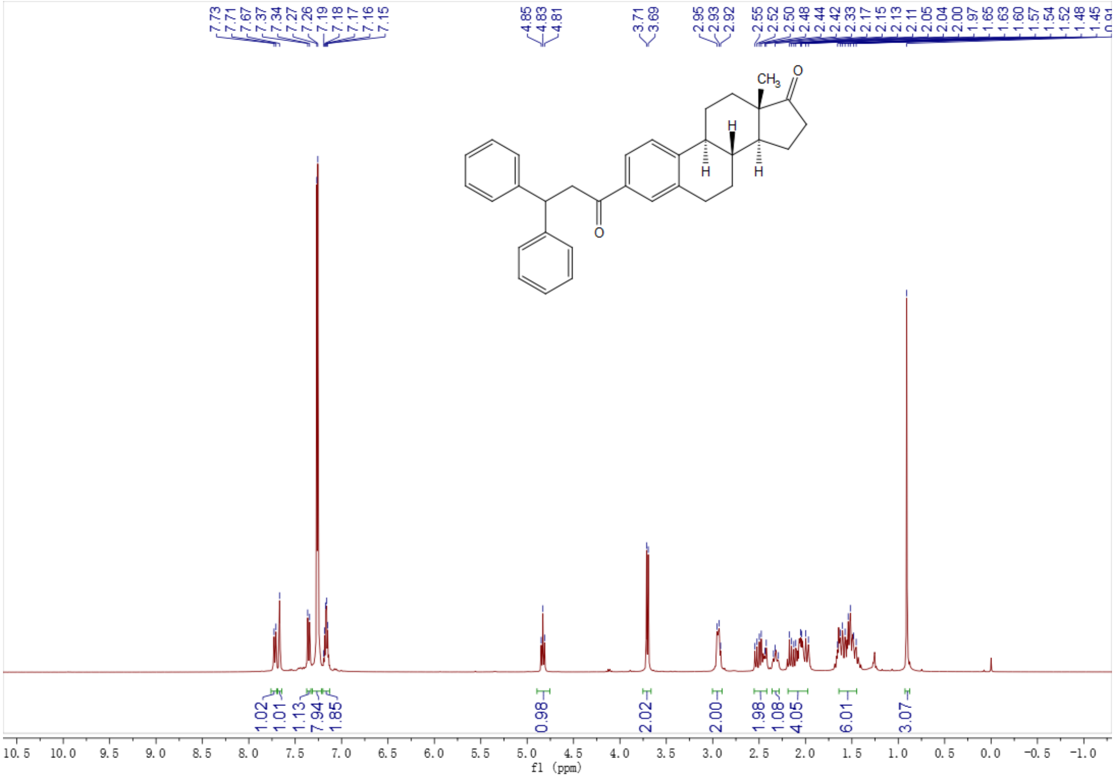

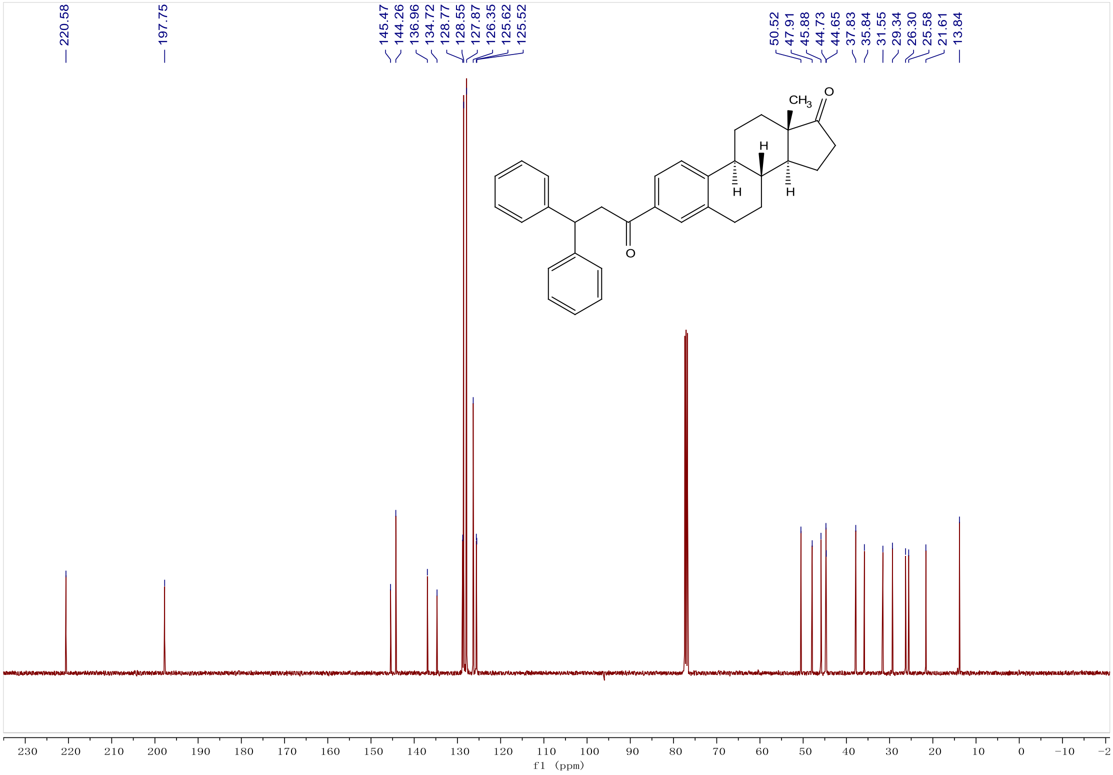

Supplementary Figure 91. ^{1}H and ^{13} C NMR spectra for compound Hiestrone **5**

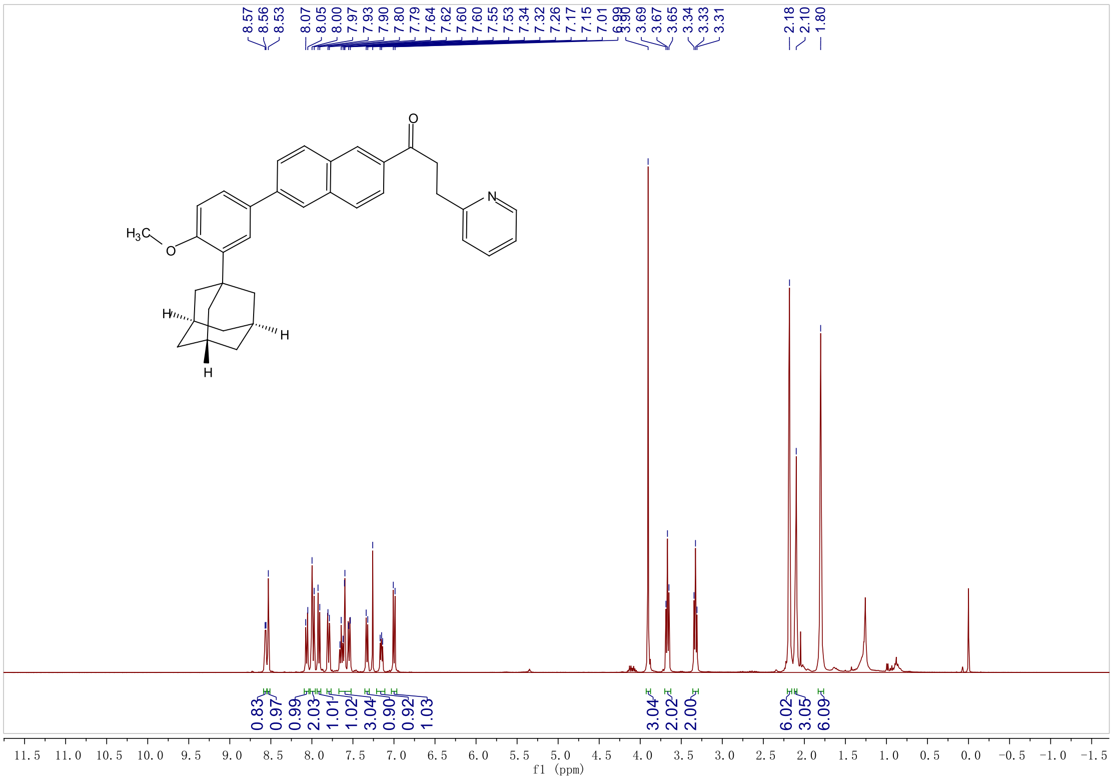

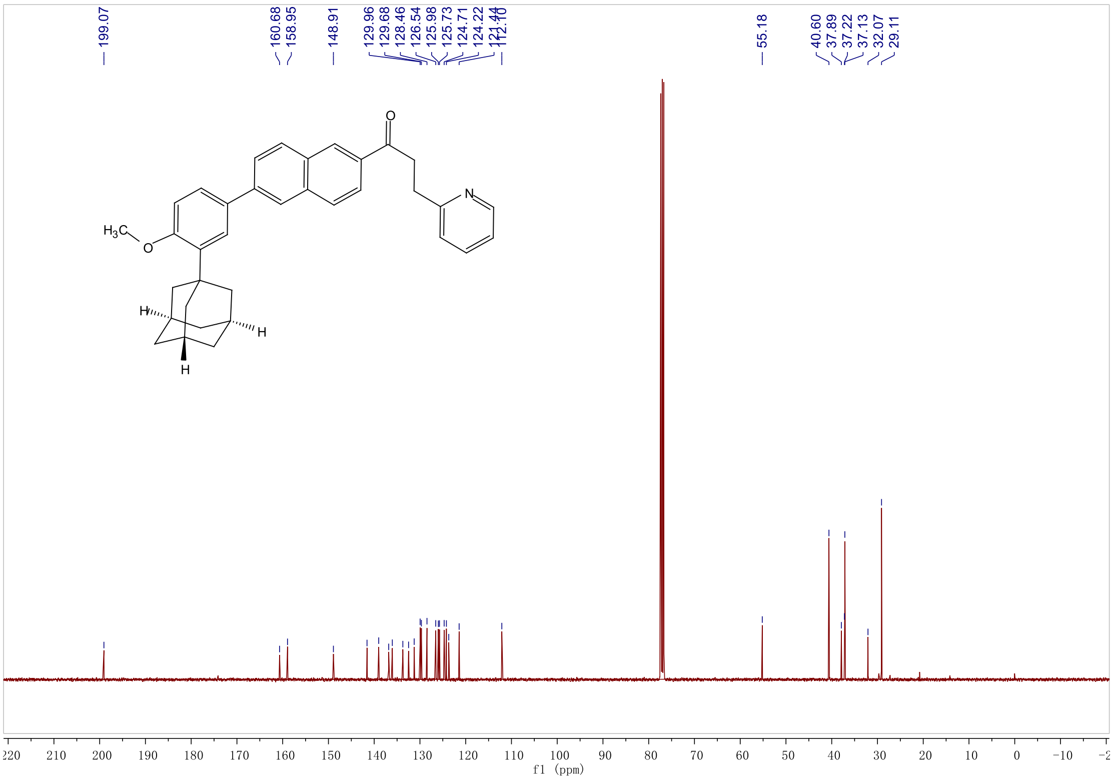

Supplementary Figure 92. ^{1}H and ^{13} C NMR spectra for compound Adapalene **6**

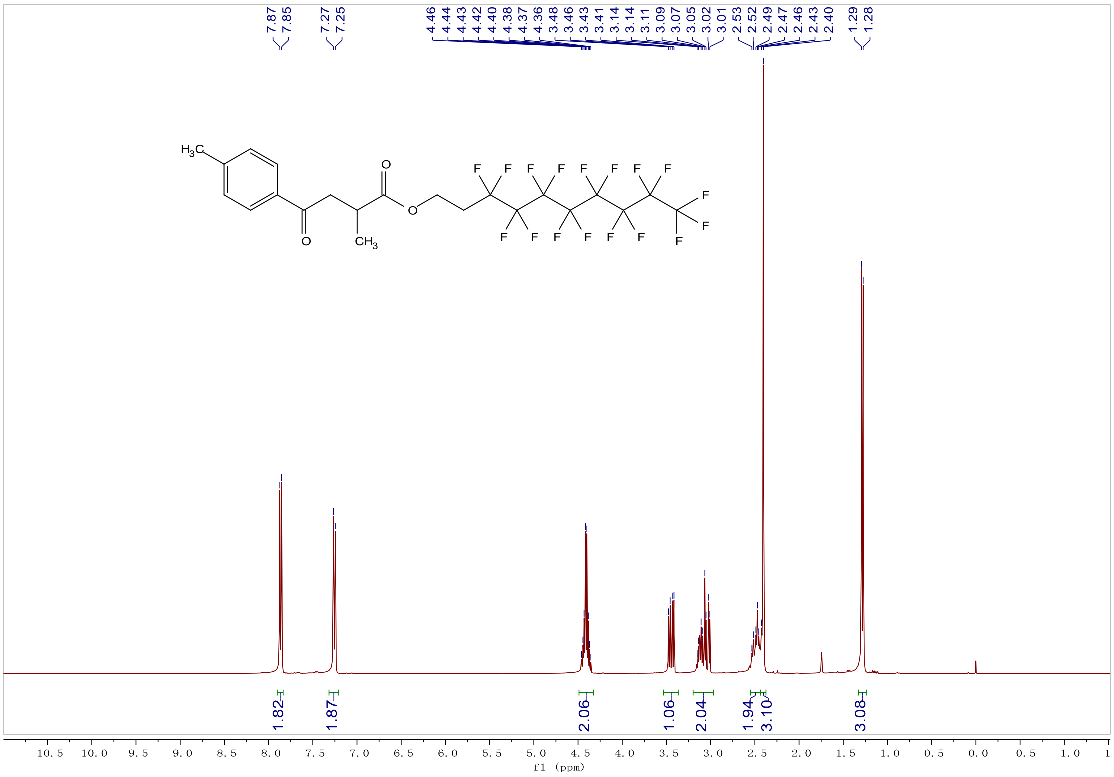

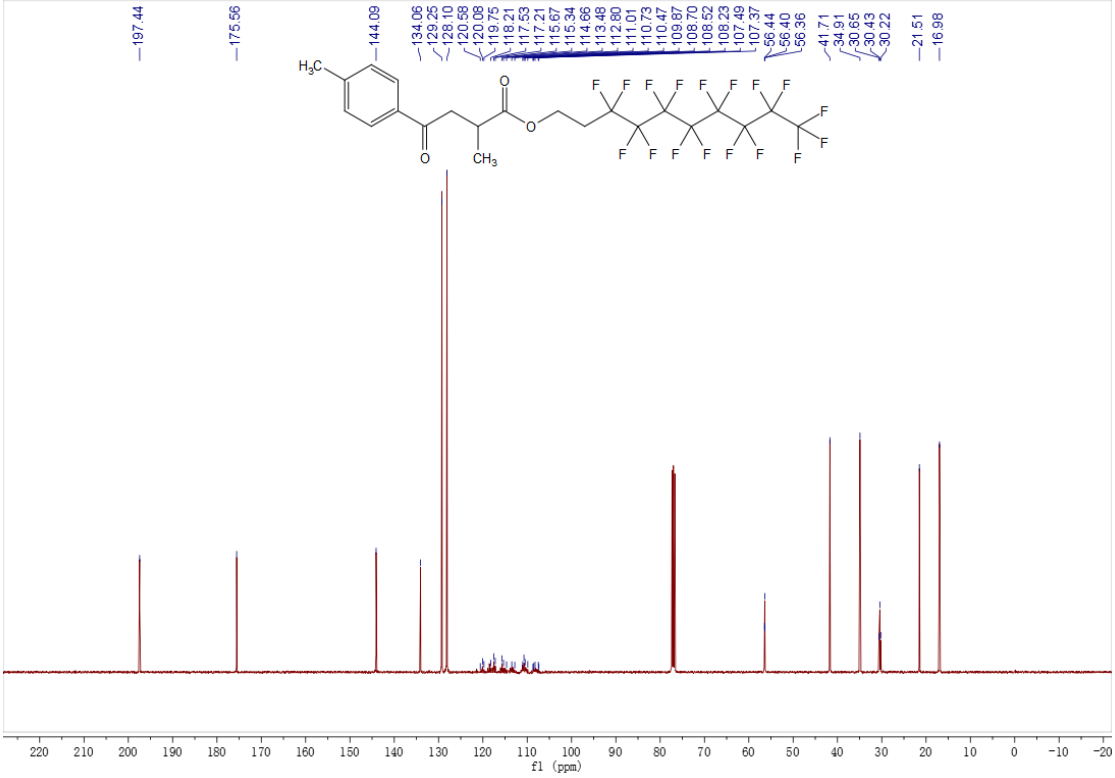

Supplementary Figure 93. ^{1}H and ^{13}C NMR spectra for compound HDFDMA **7**

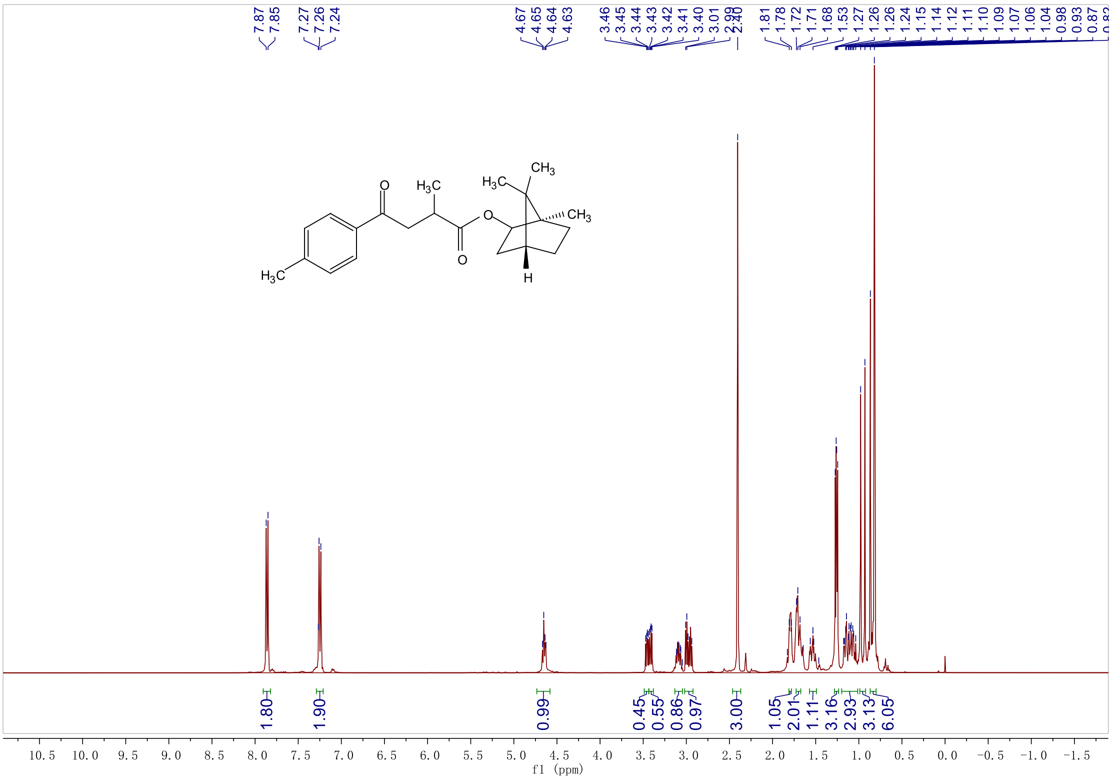

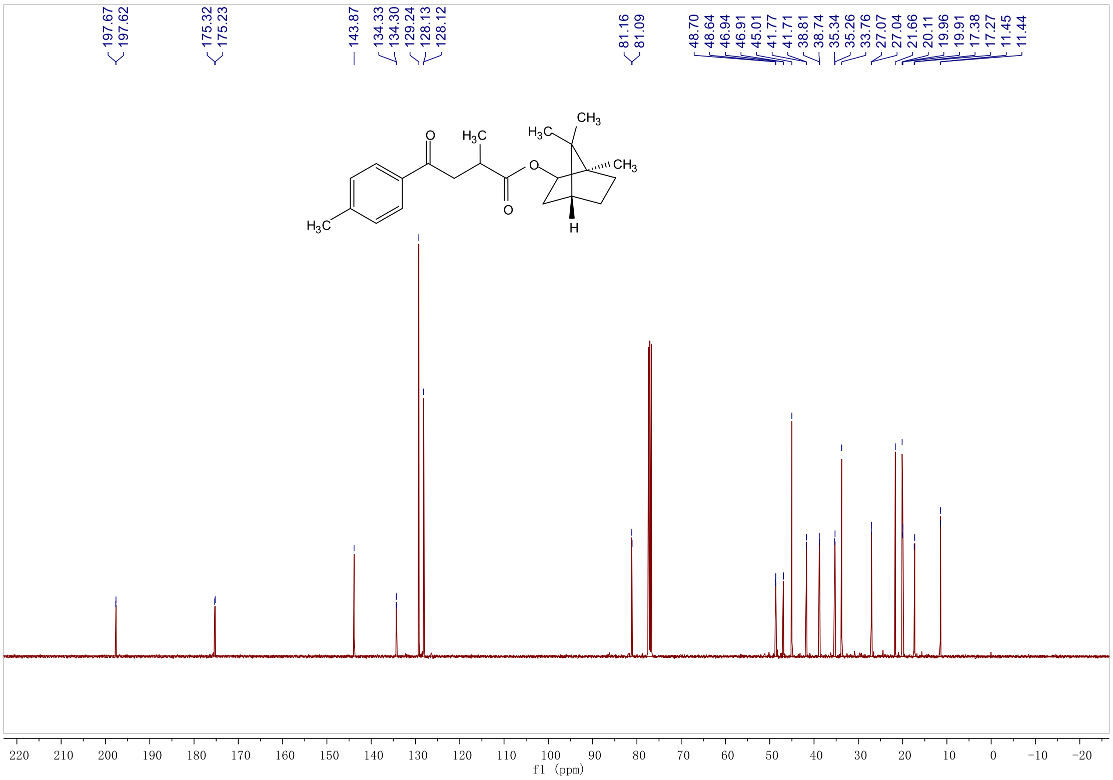

Supplementary Figure 94. ^{1}H and ^{13}C NMR spectra for compound Acryester IBX **8**

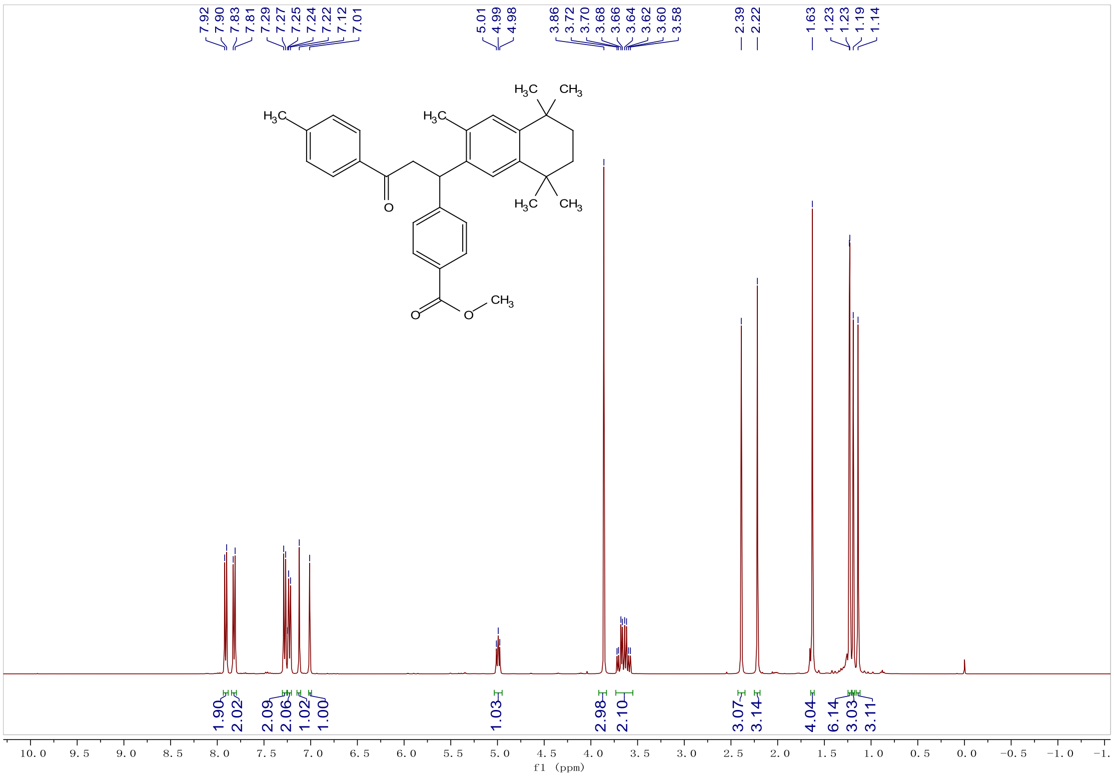

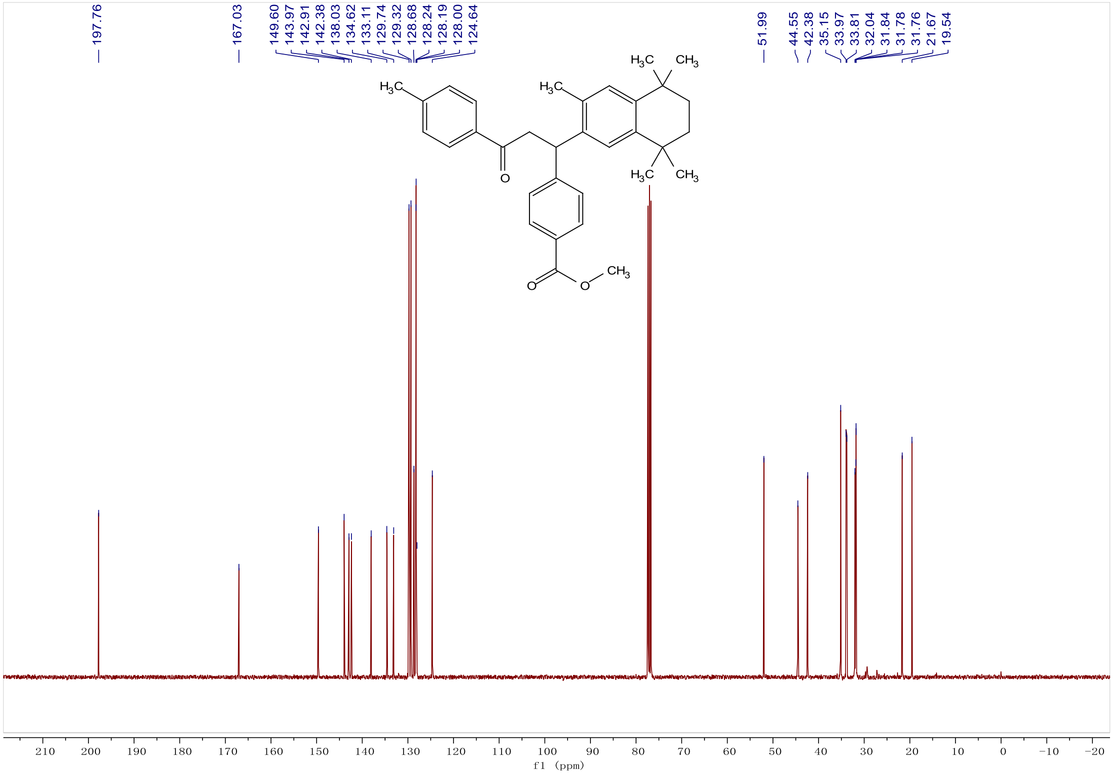

Supplementary Figure 95. ^{1}H and ^{13} C NMR spectra for compound Bexarotene **9**

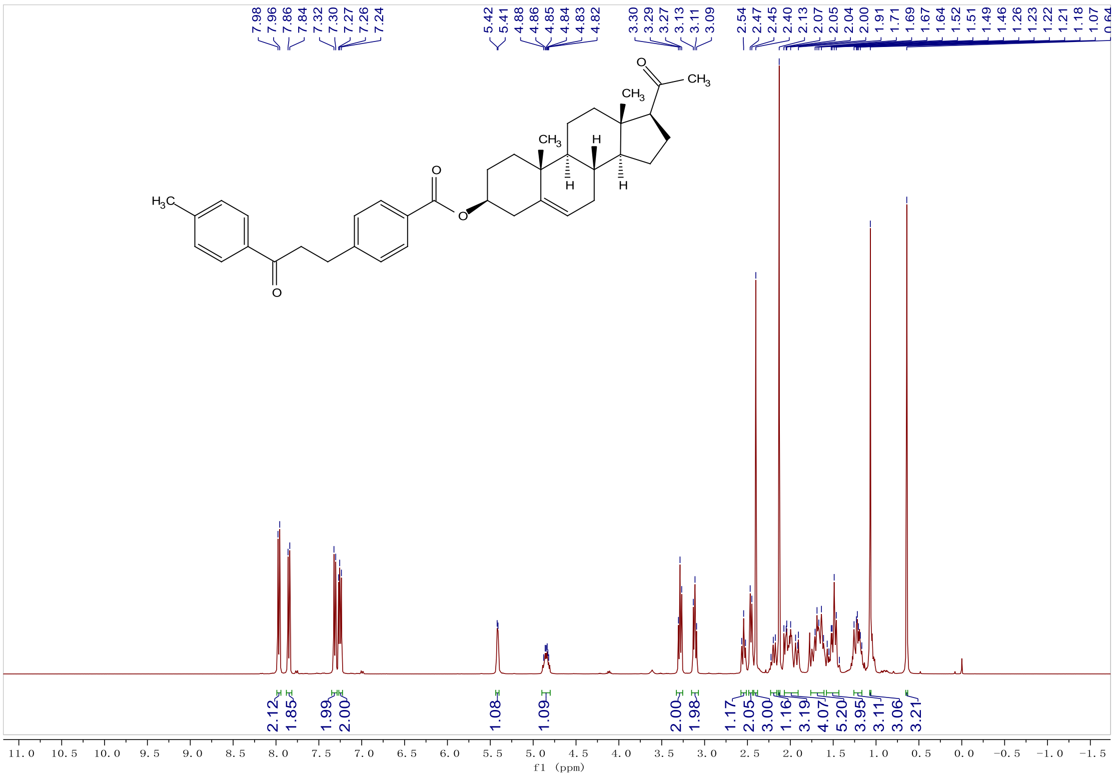

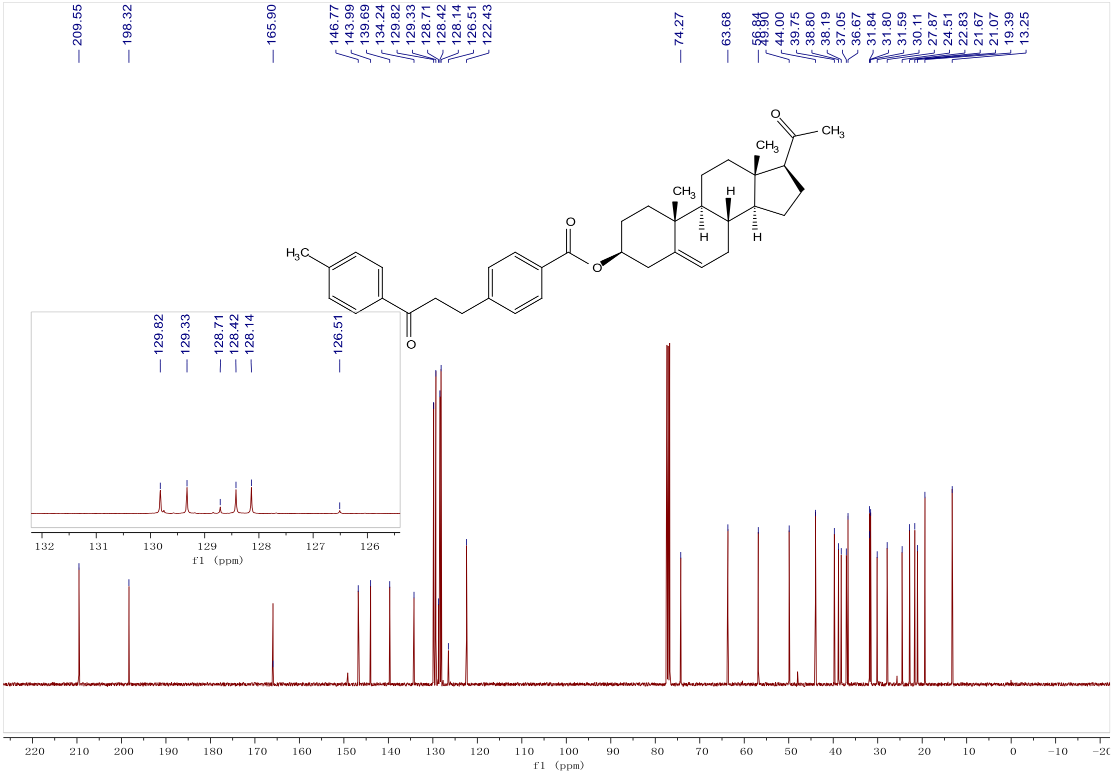

Supplementary Figure 96. ^{1}H and ^{13}C NMR spectra for compound Pregnenolone **10**

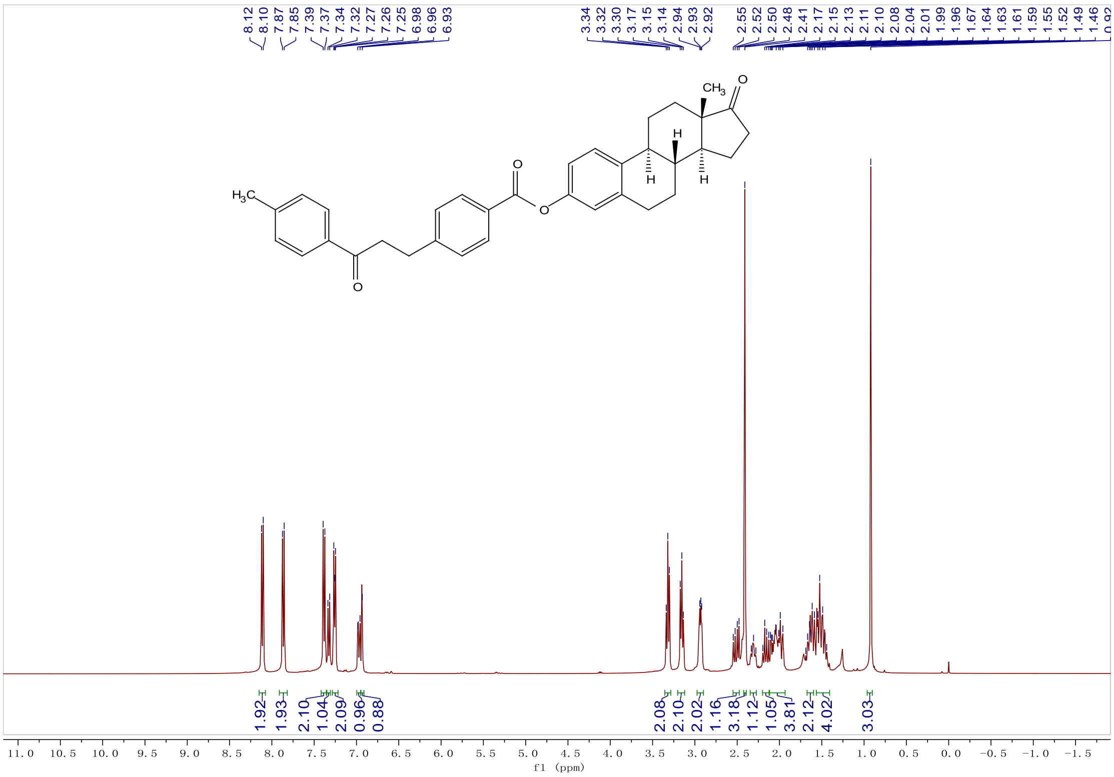

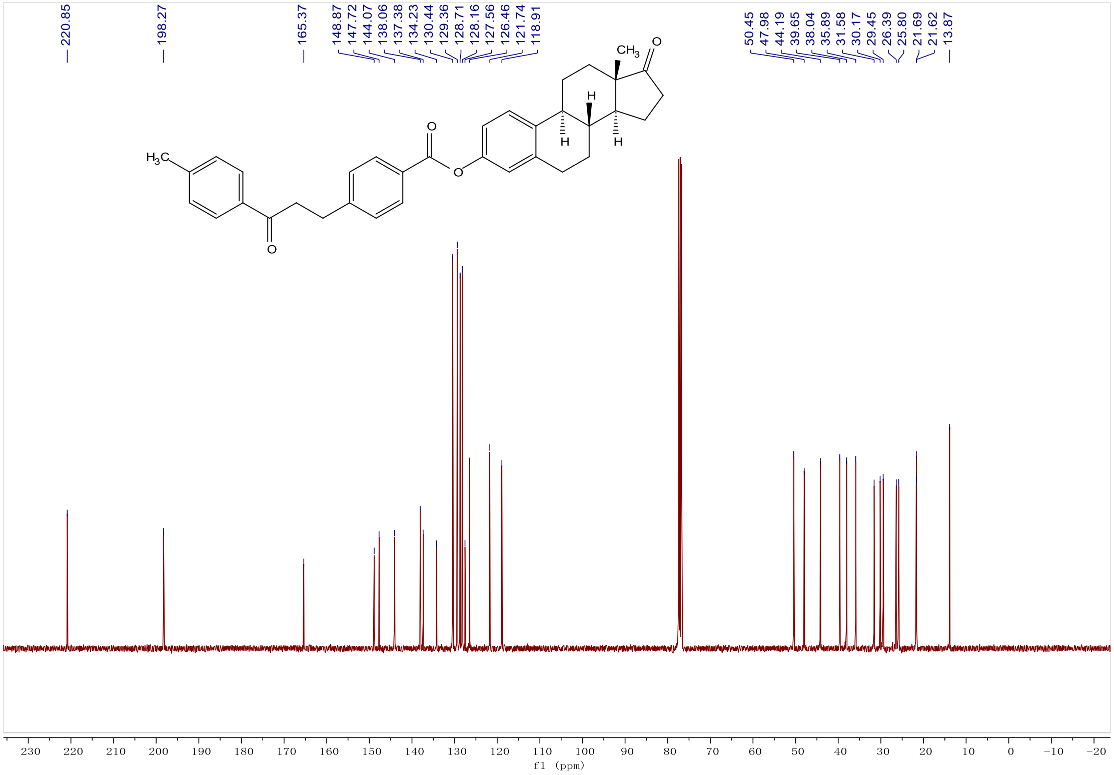

Supplementary Figure 97. ^{1}H and ^{13} C NMR spectra for compound Esterone **11**

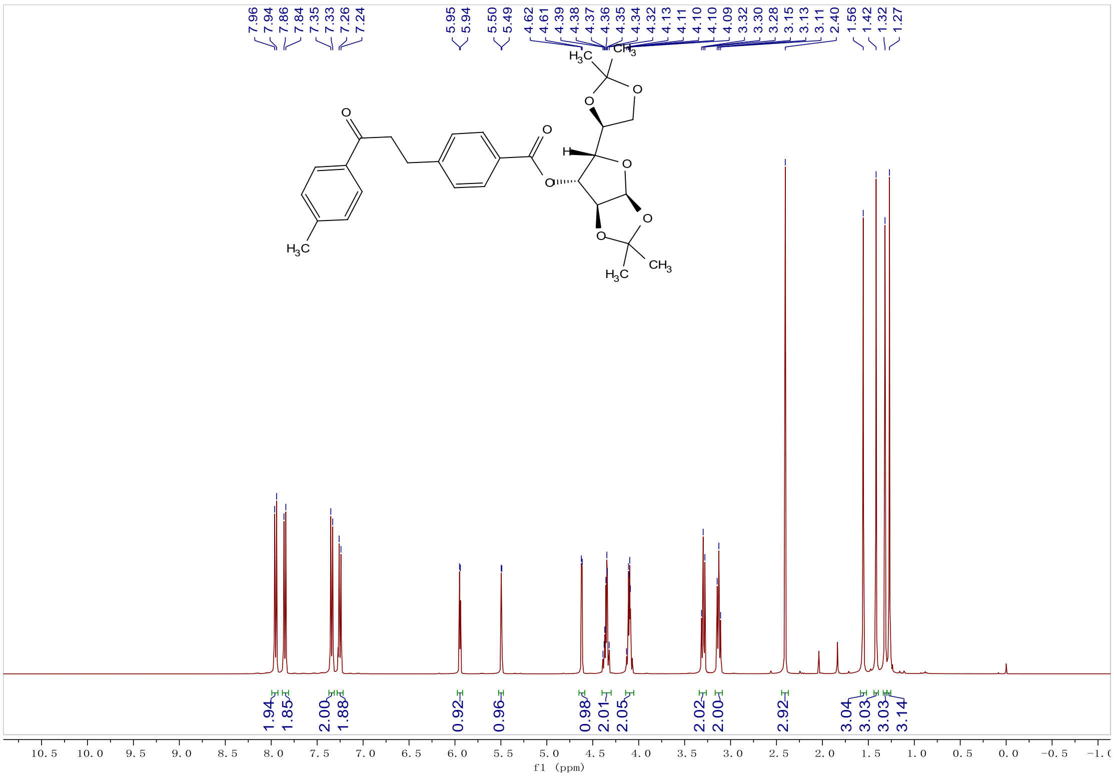

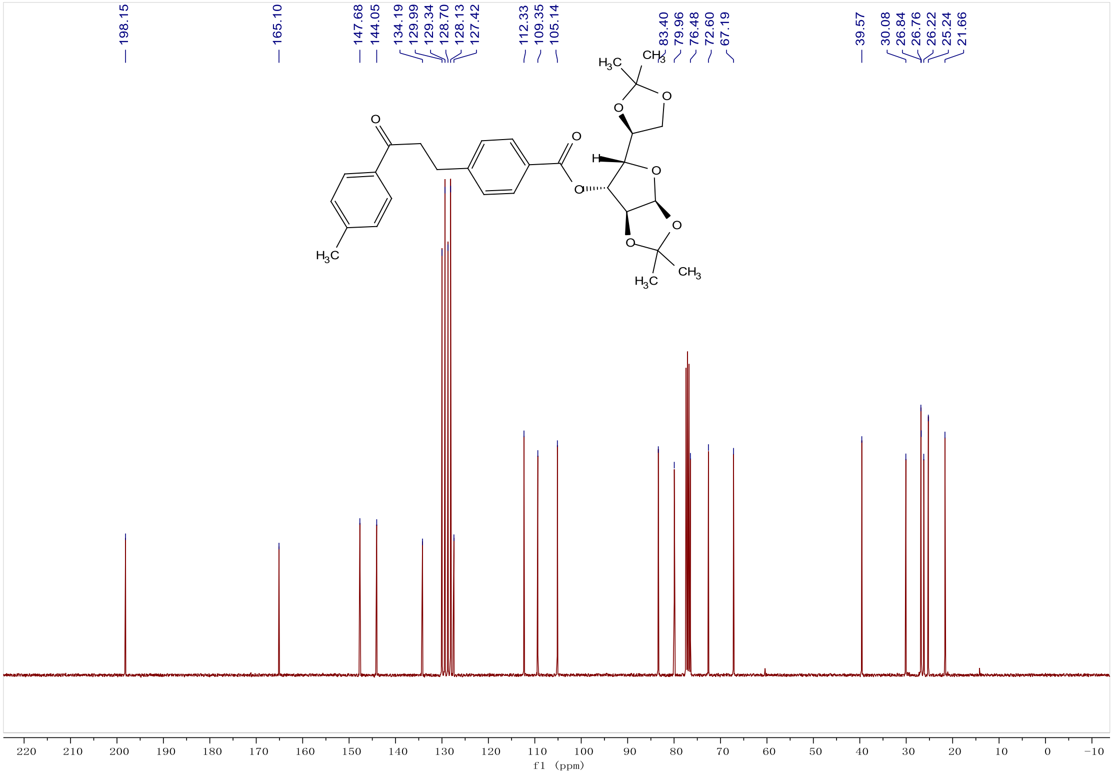

Supplementary Figure 98. ^{1}H and ^{13}C NMR spectra for compound **12**

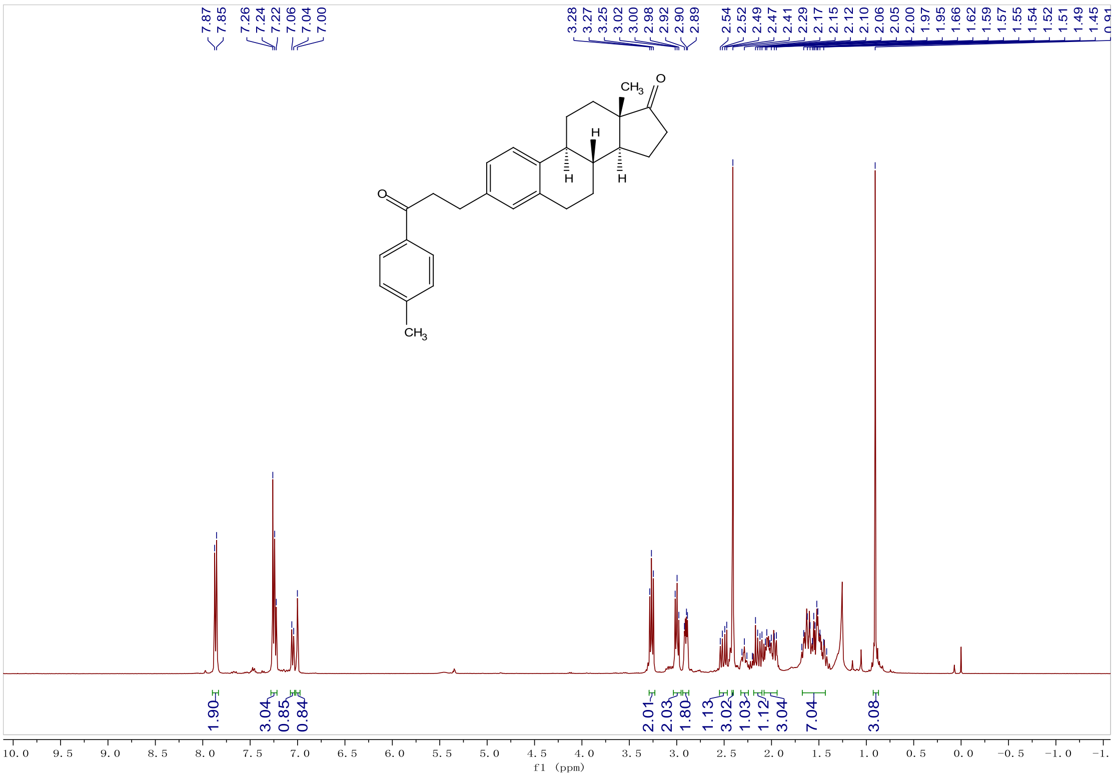

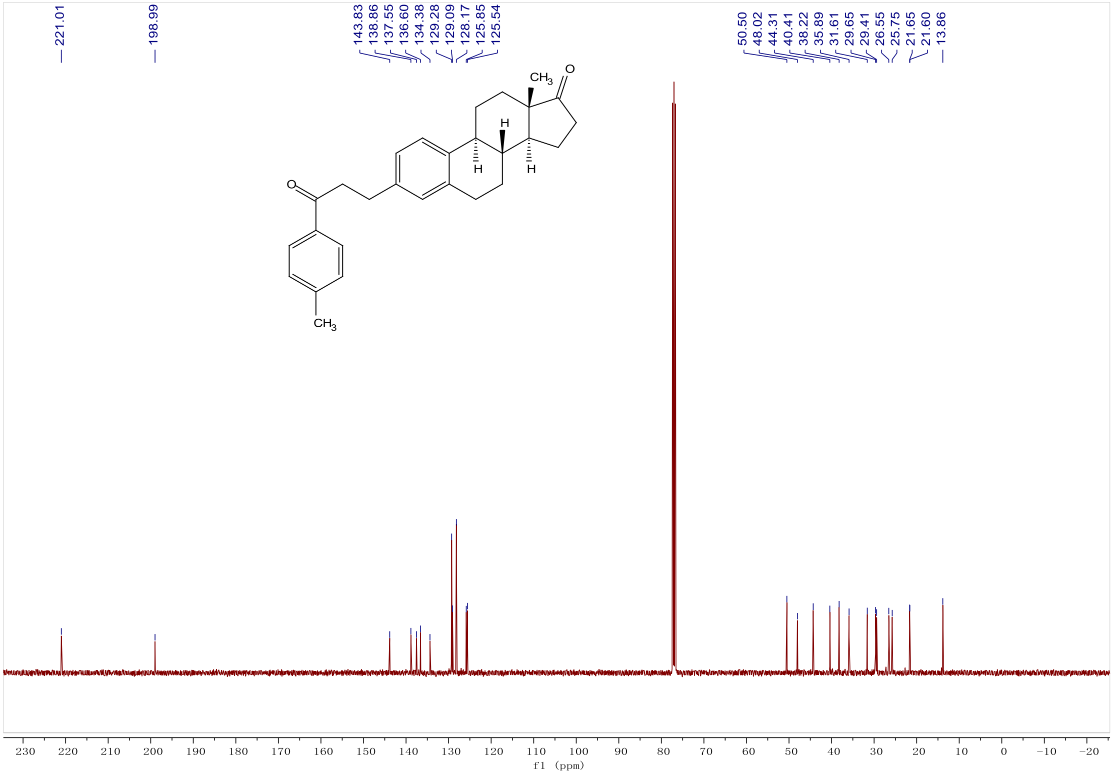

Supplementary Figure 99. ^{1}H and ^{13} C NMR spectra for compound Esterone **13**

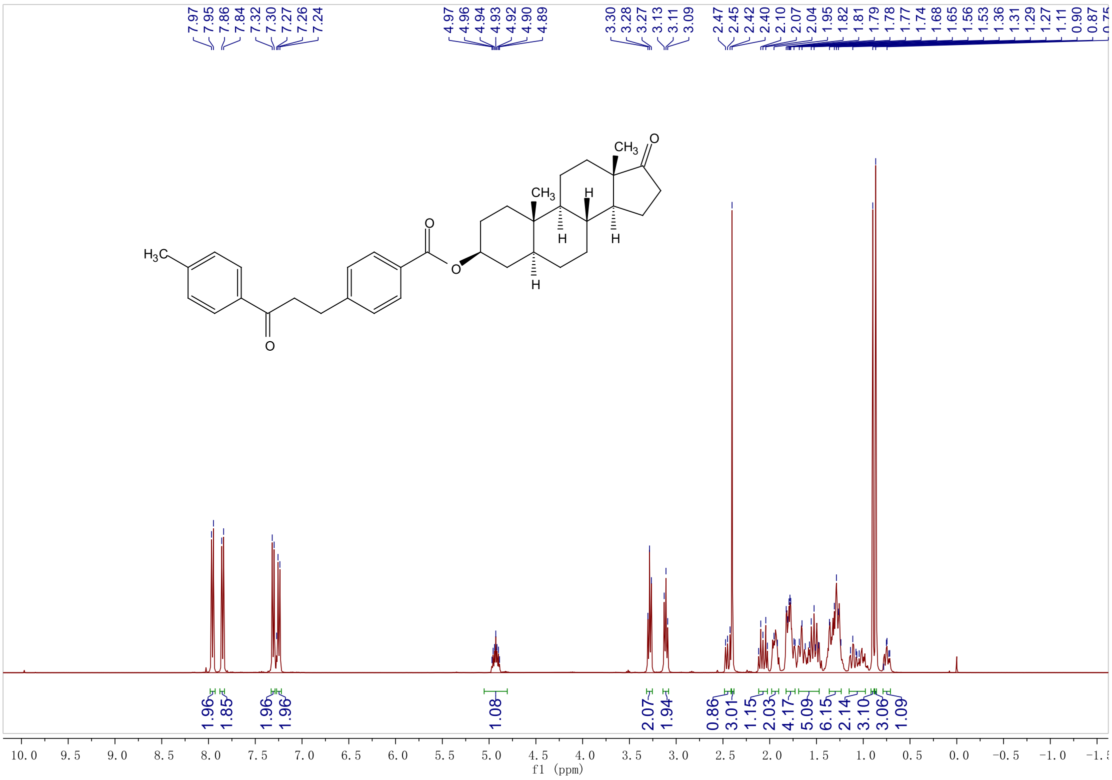

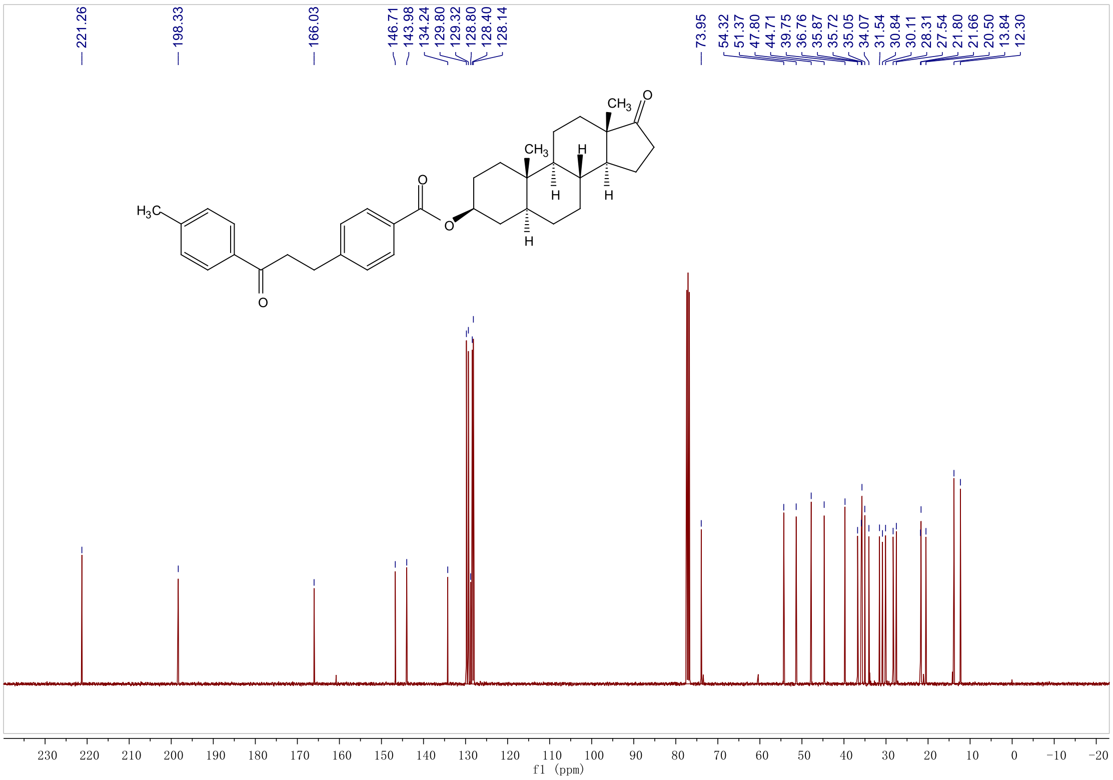

Supplementary Figure 100. ^{1}H and ^{13}C NMR spectra for compound **14**

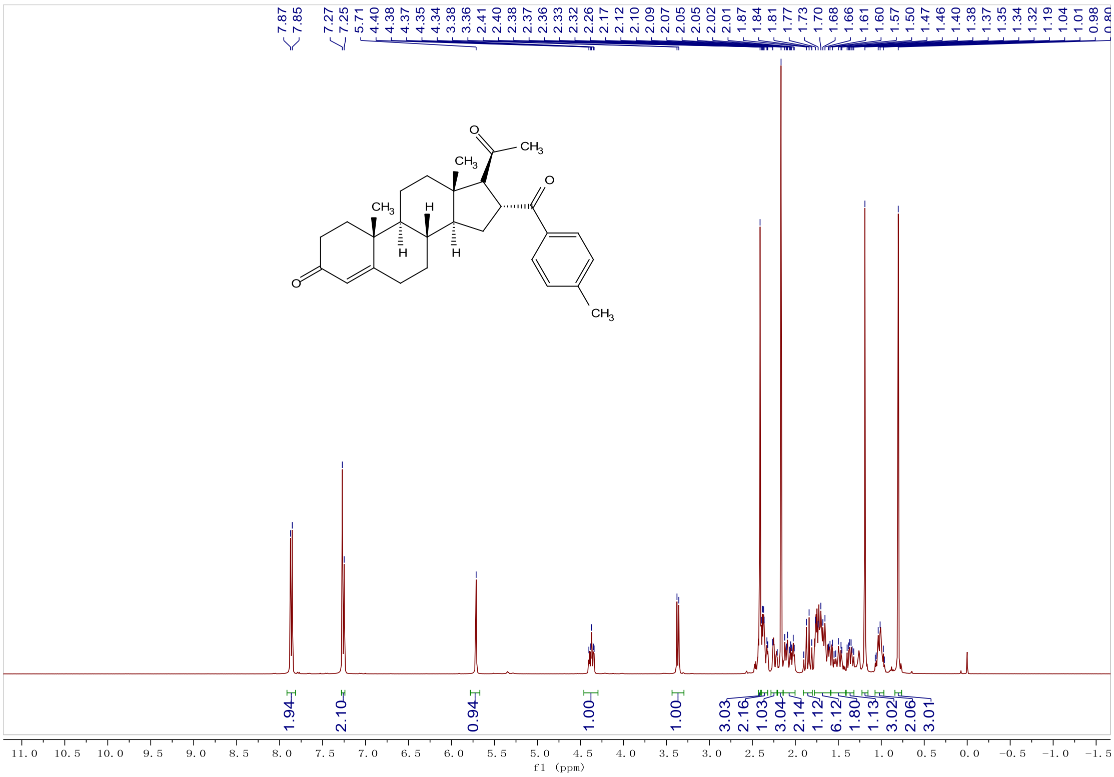

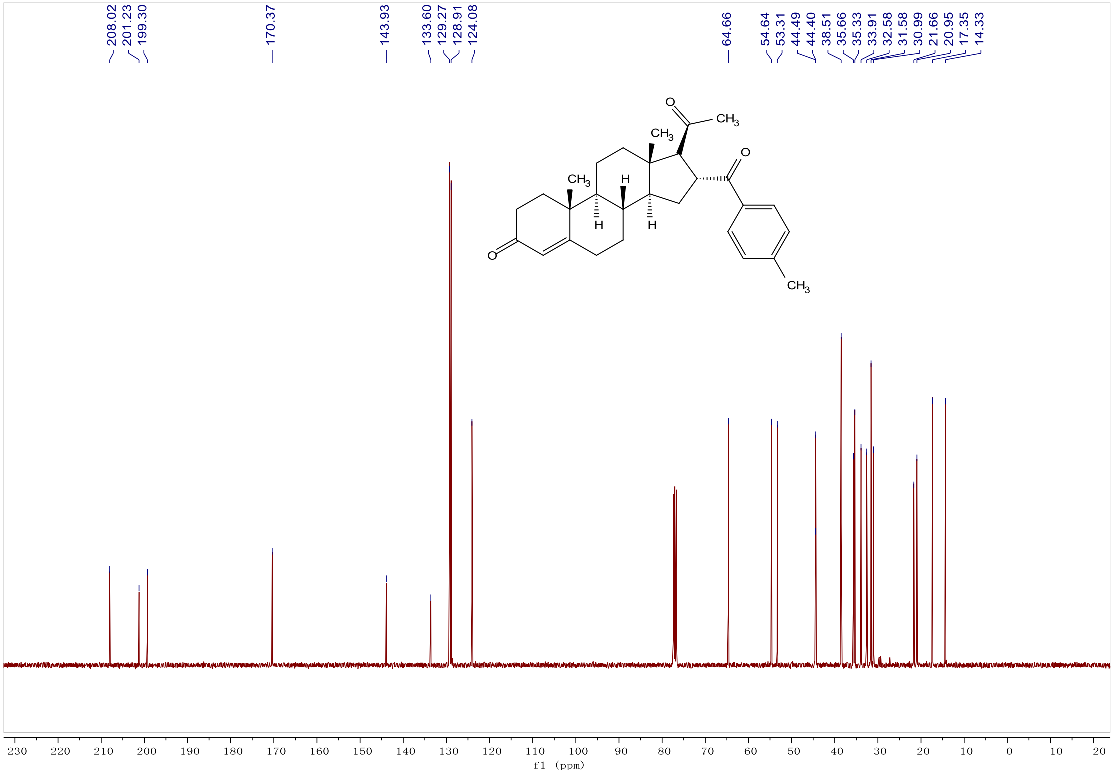

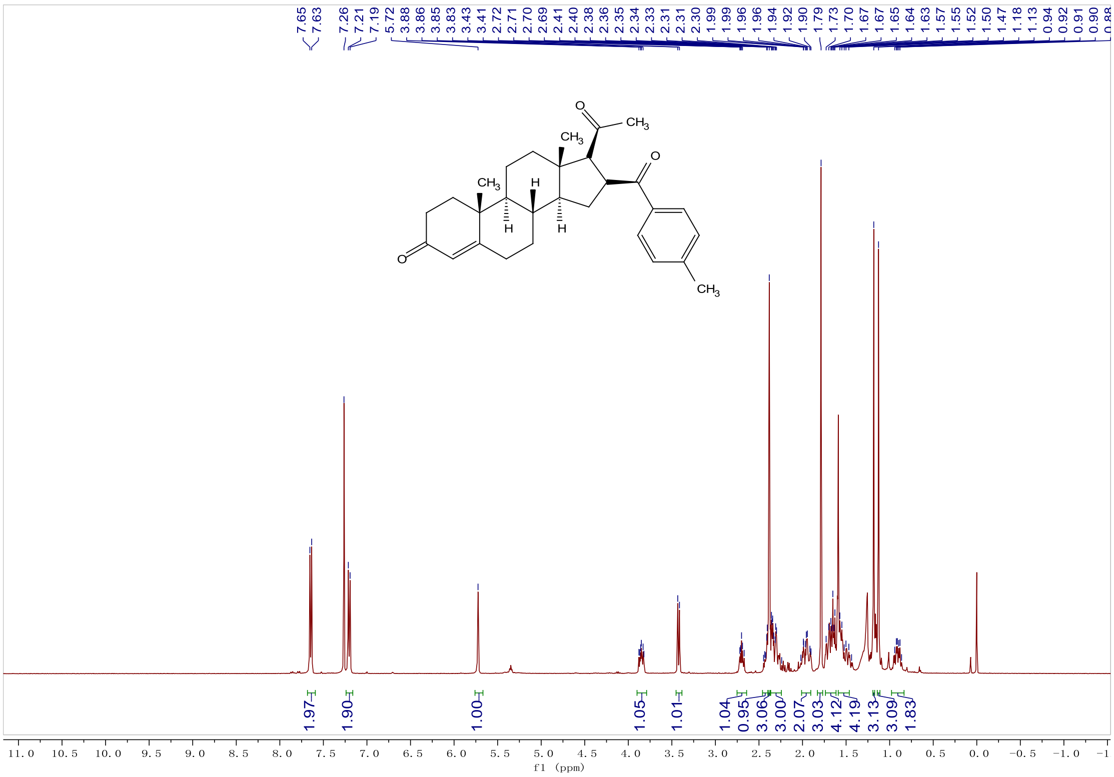

Supplementary Figure 101. ^{1}H and ^{13}C NMR spectra for compound **15**

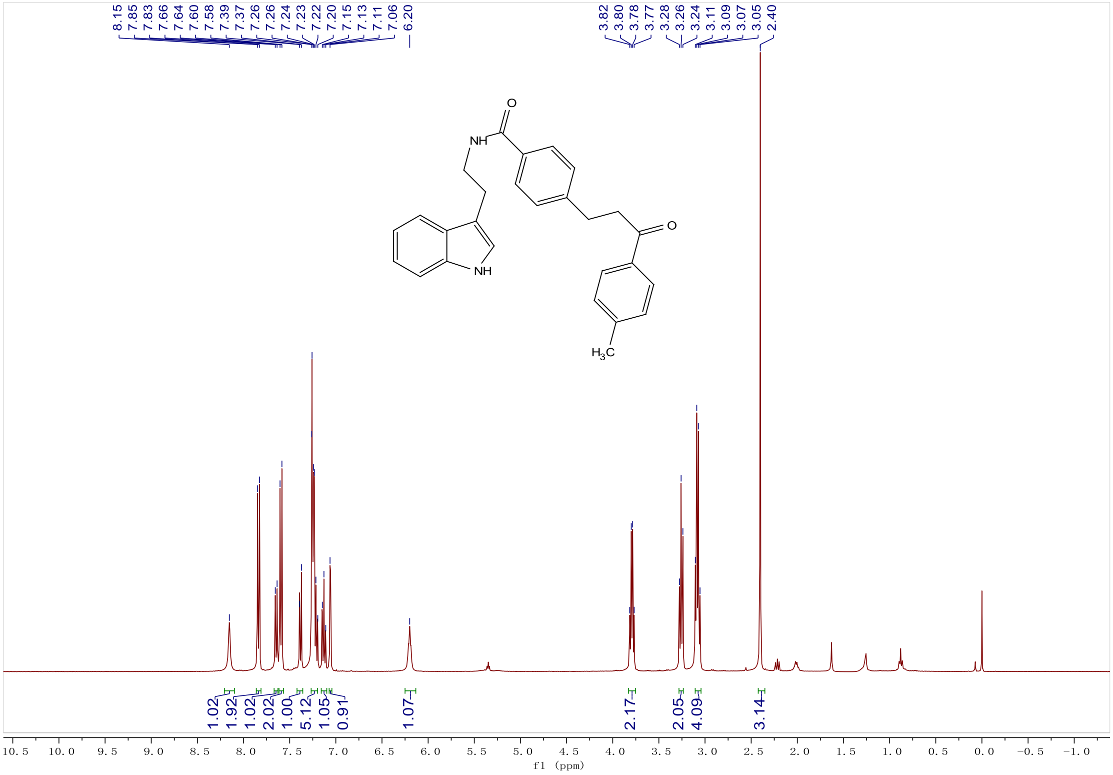

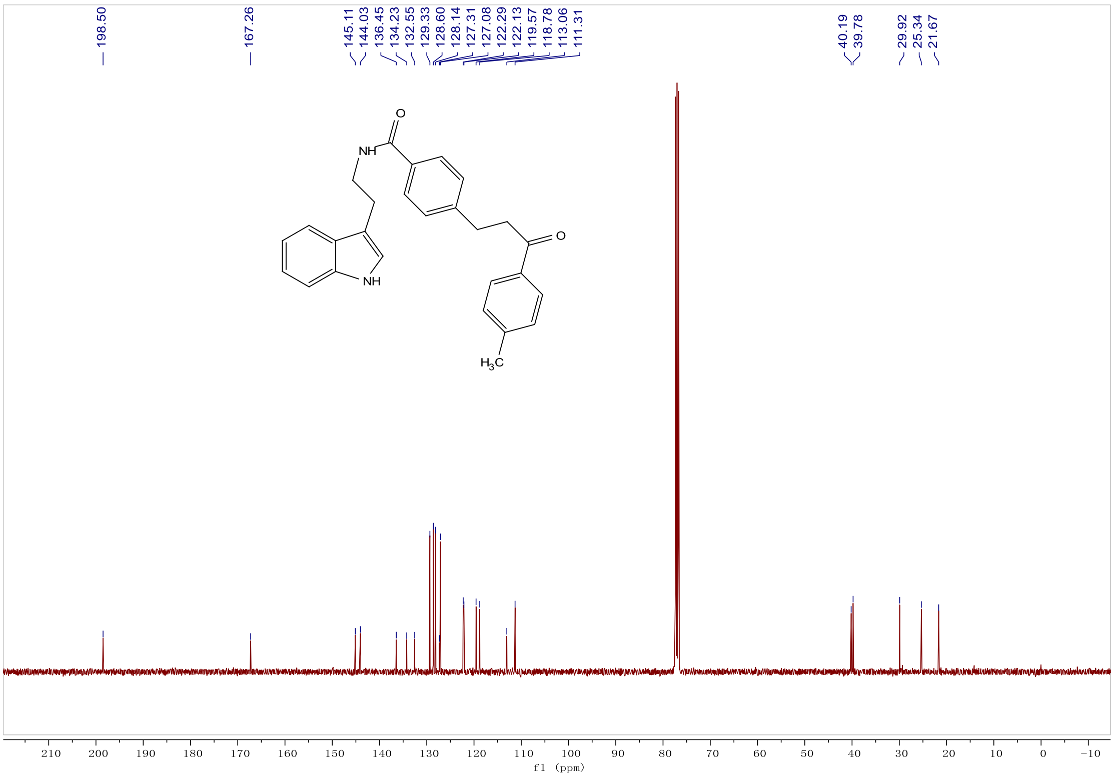

Supplementary Figure 102. ^{1}H and ^{13}C NMR spectra for compound **16**

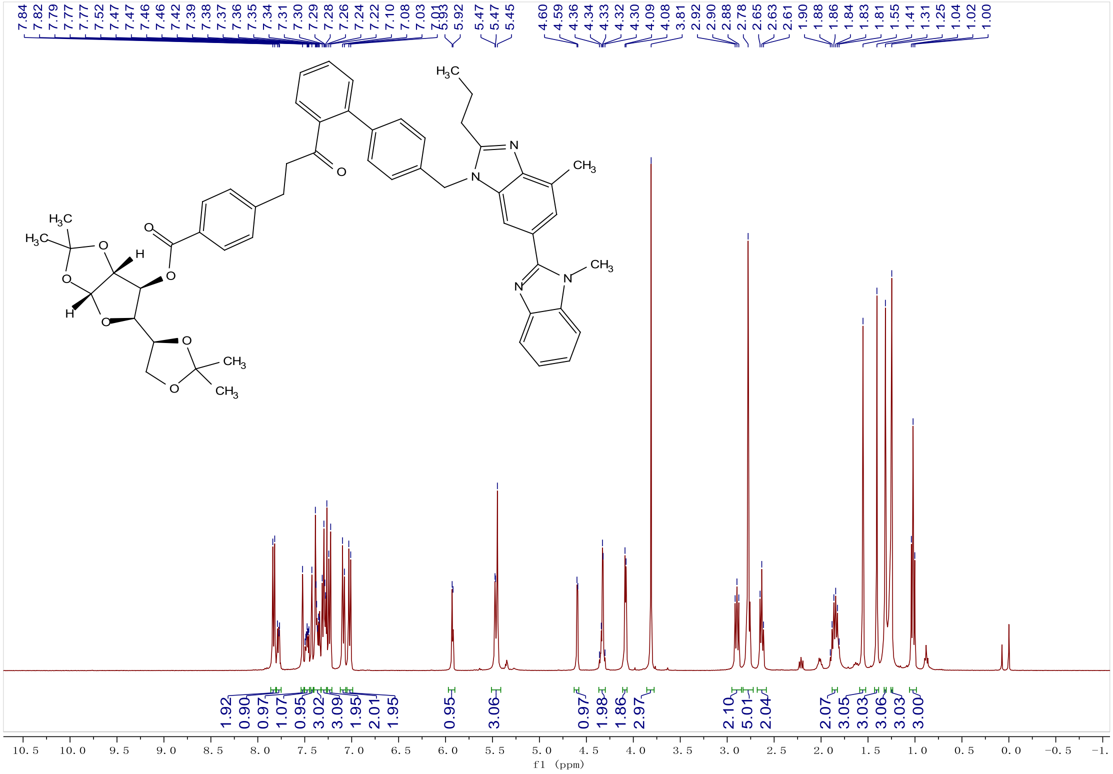

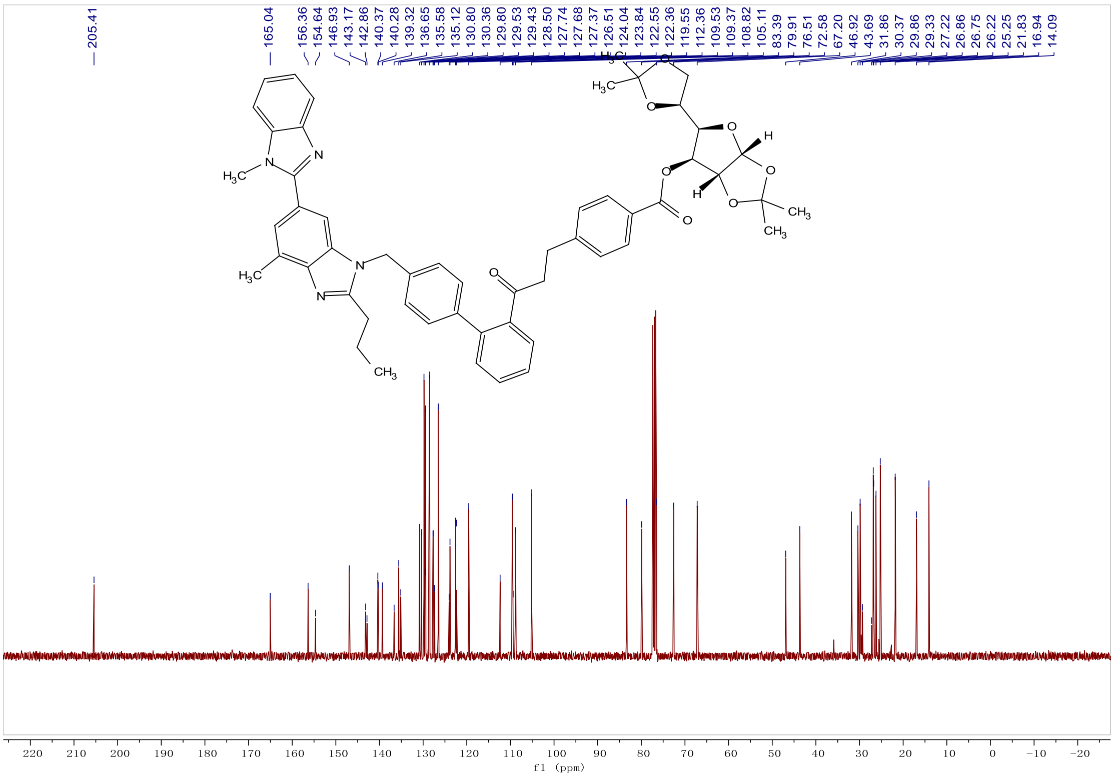

Supplementary Figure 103. ^{1}H and ^{13}C NMR spectra for compound **17**

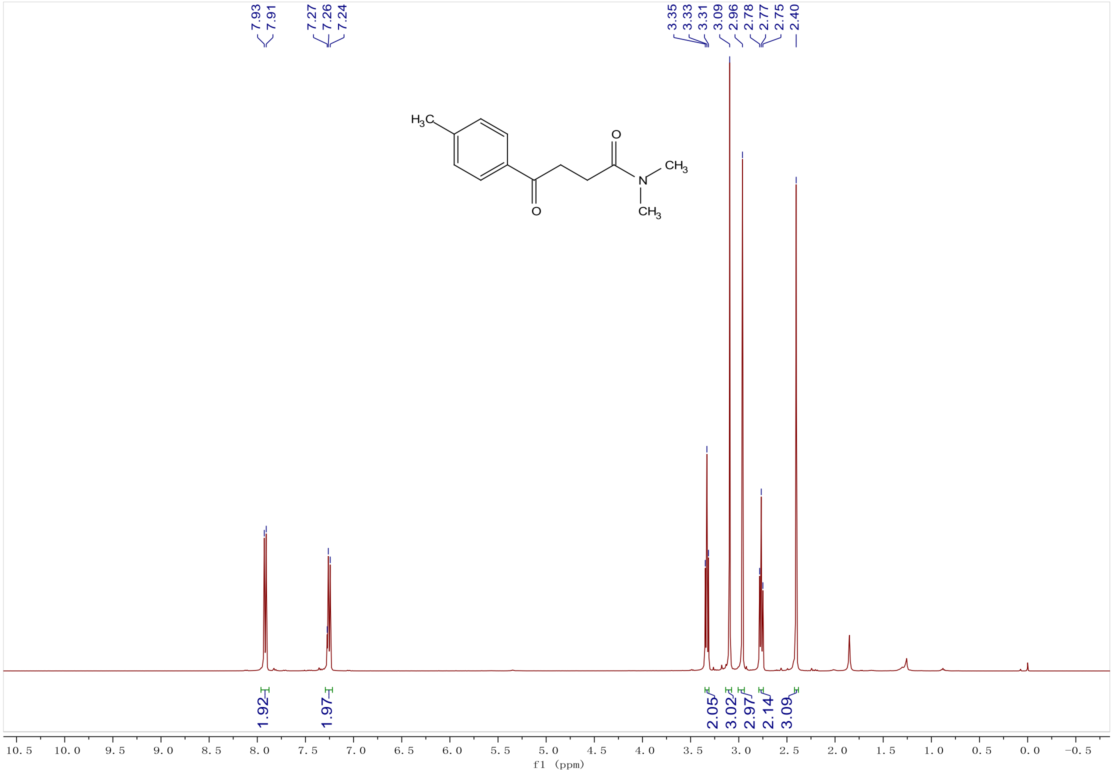

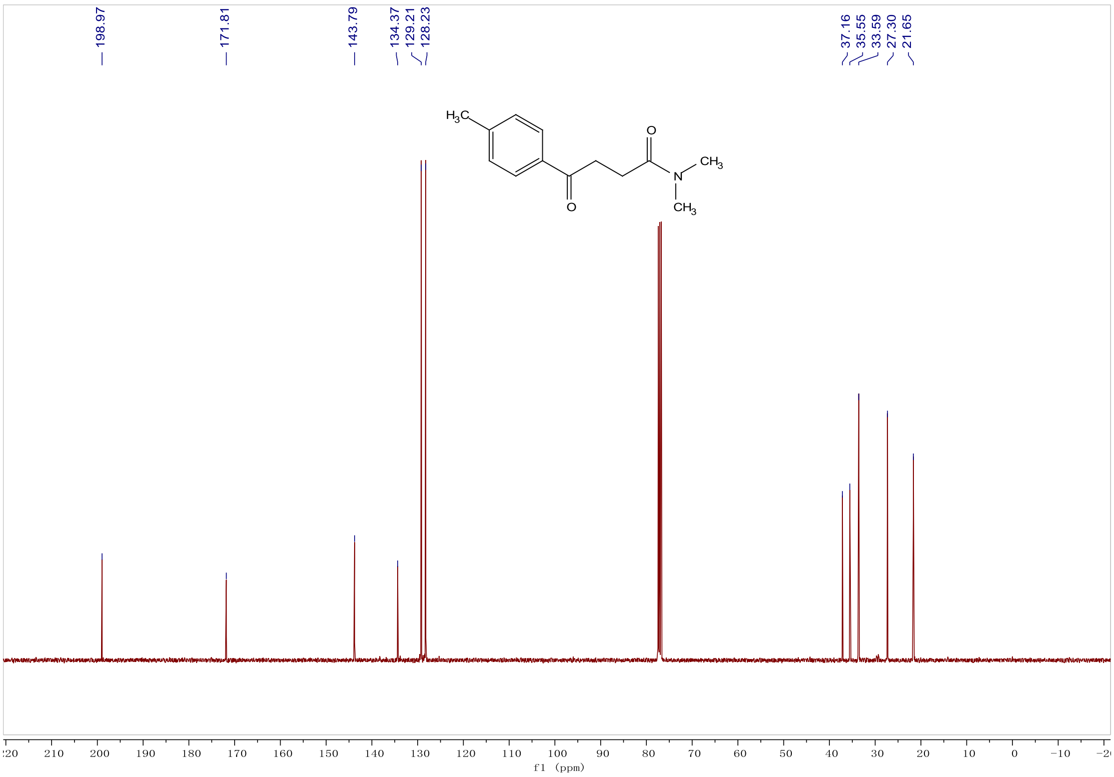

Supplementary Figure 104. ^{1}H and ^{13}C NMR spectra for compound **19**

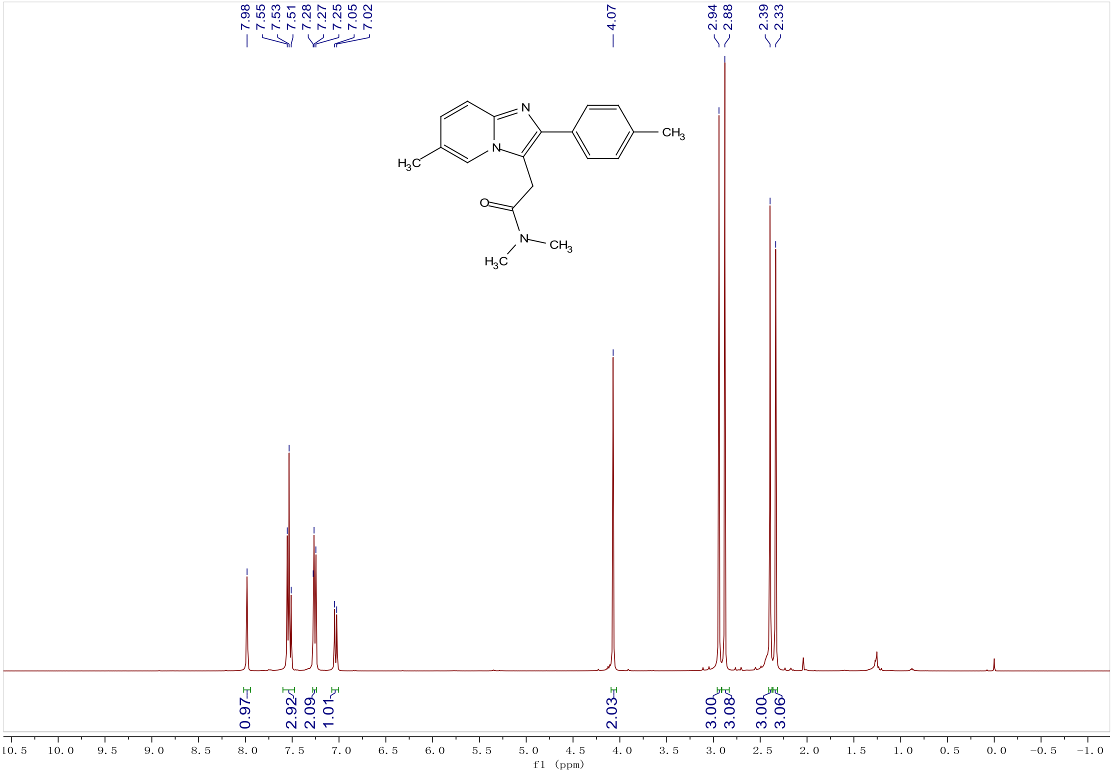

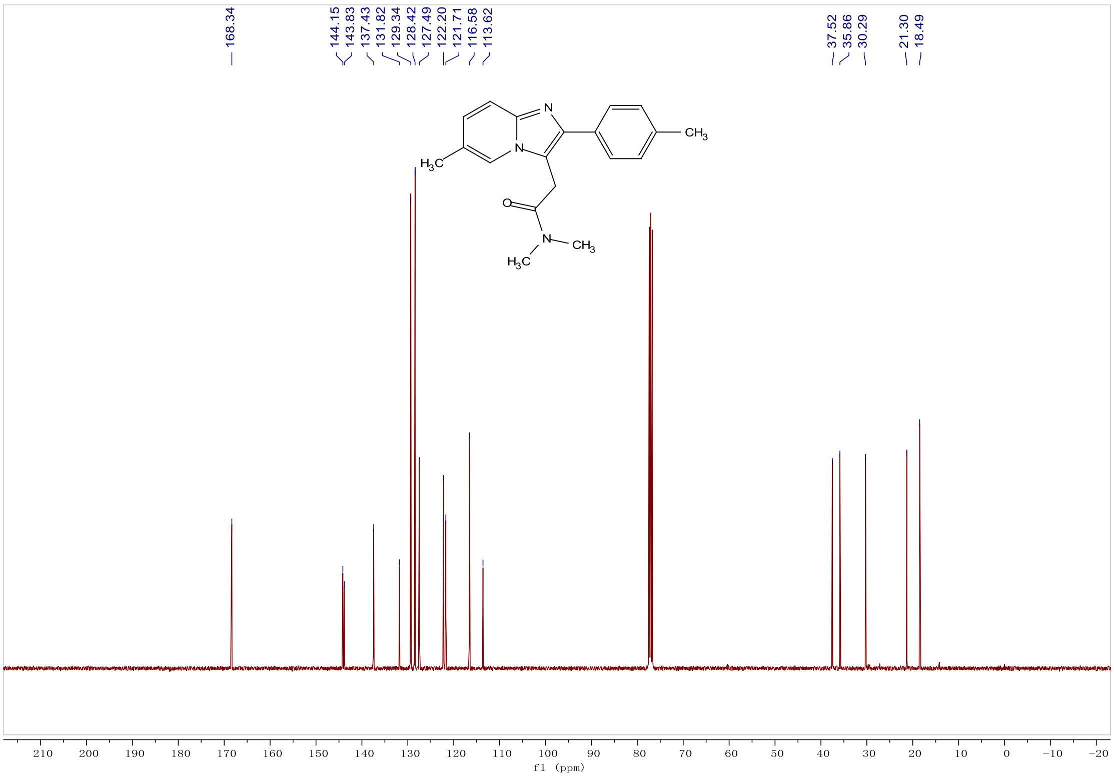

Supplementary Figure 105. ^{1}H and ^{13} C NMR spectra for compound Zolpidem

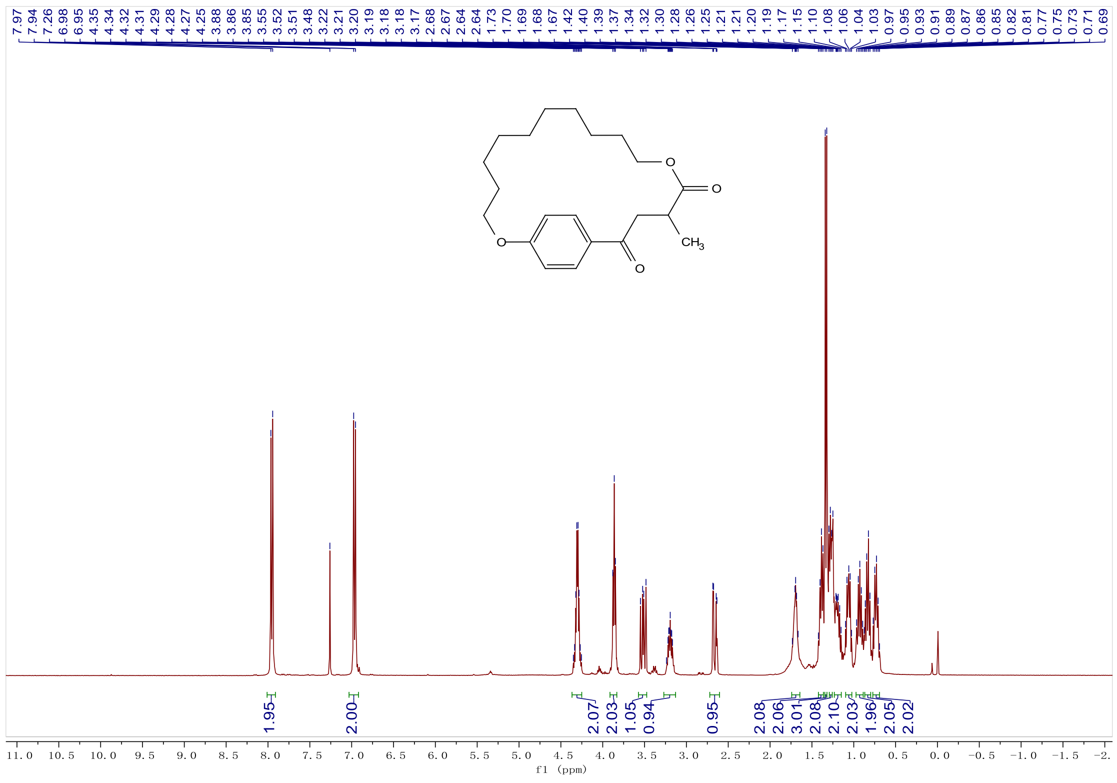

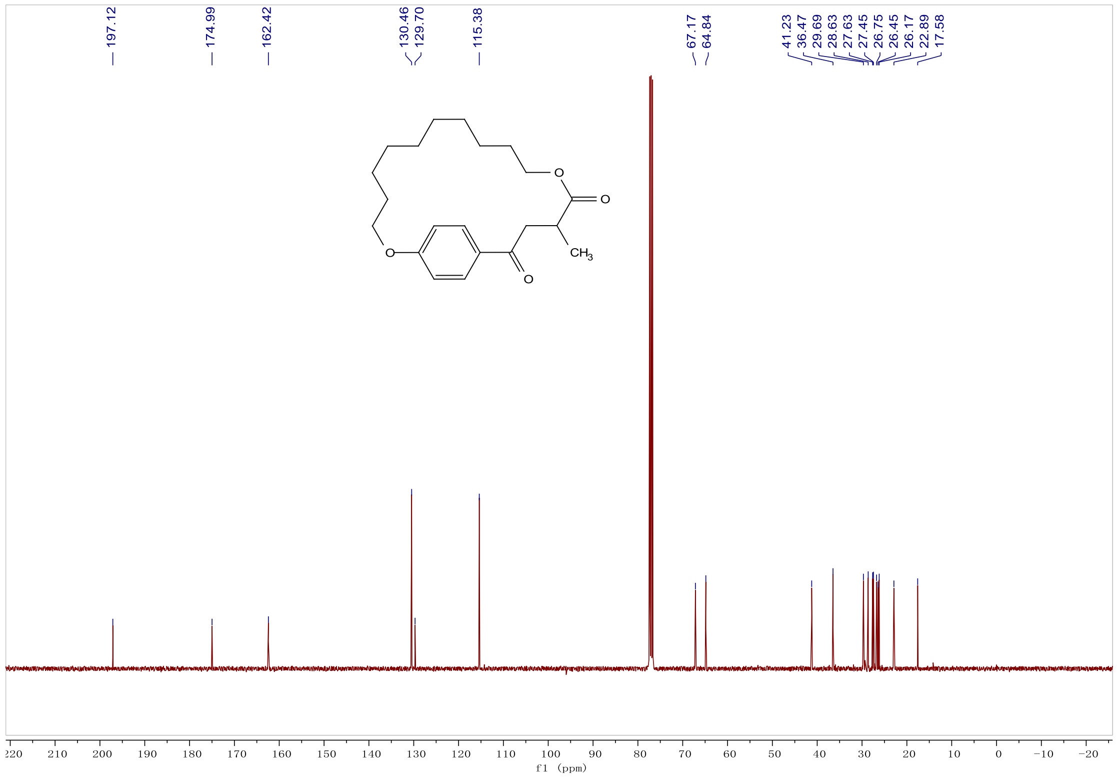

Supplementary Figure 106. ^{1}H and ^{13} C NMR spectra for compound **21a**

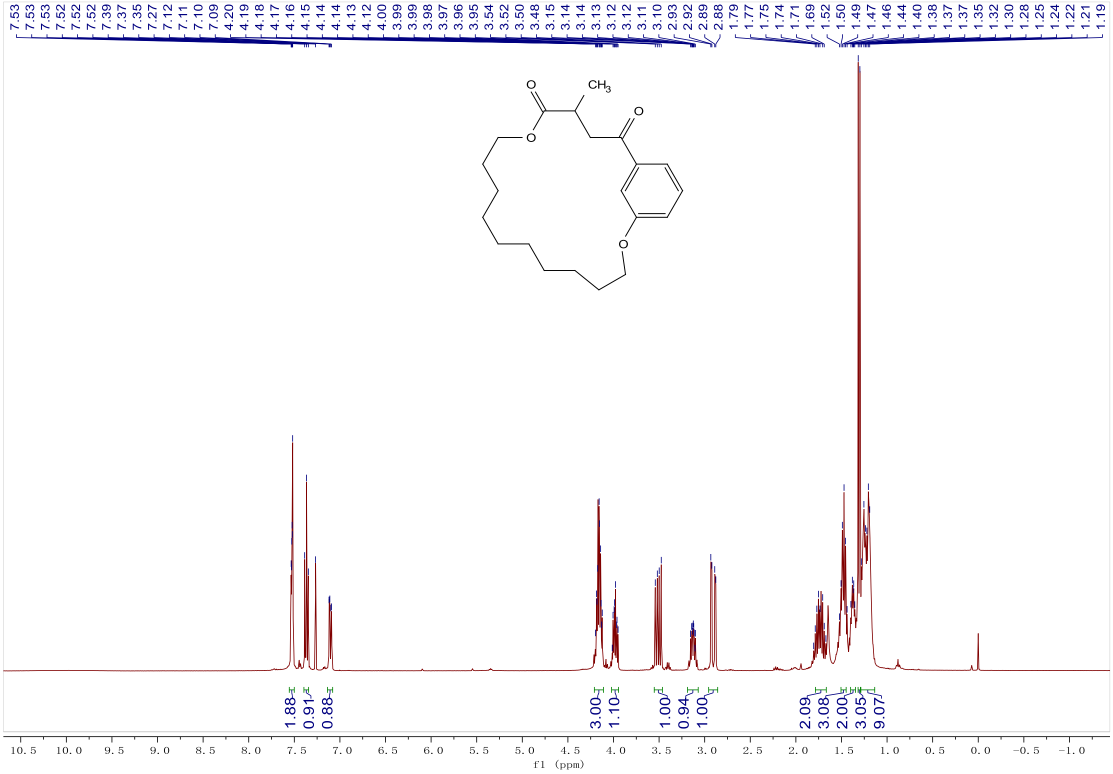

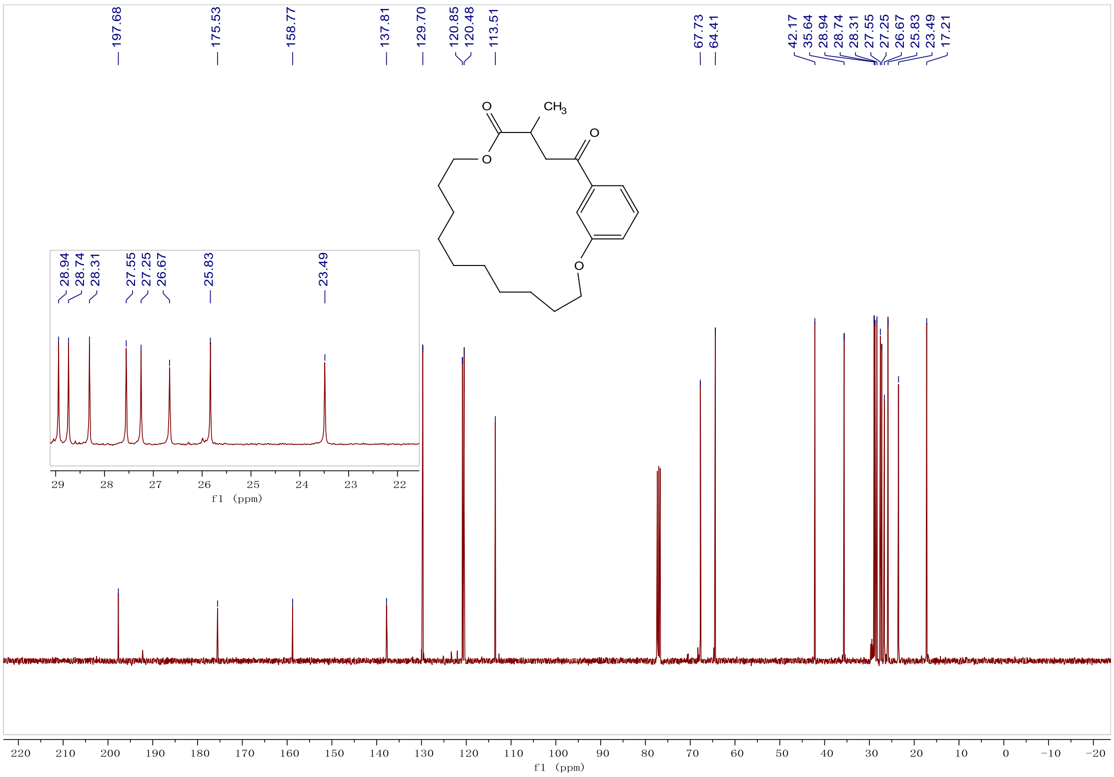

Supplementary Figure 107. ^{1}H and ^{13}C NMR spectra for compound **21b**

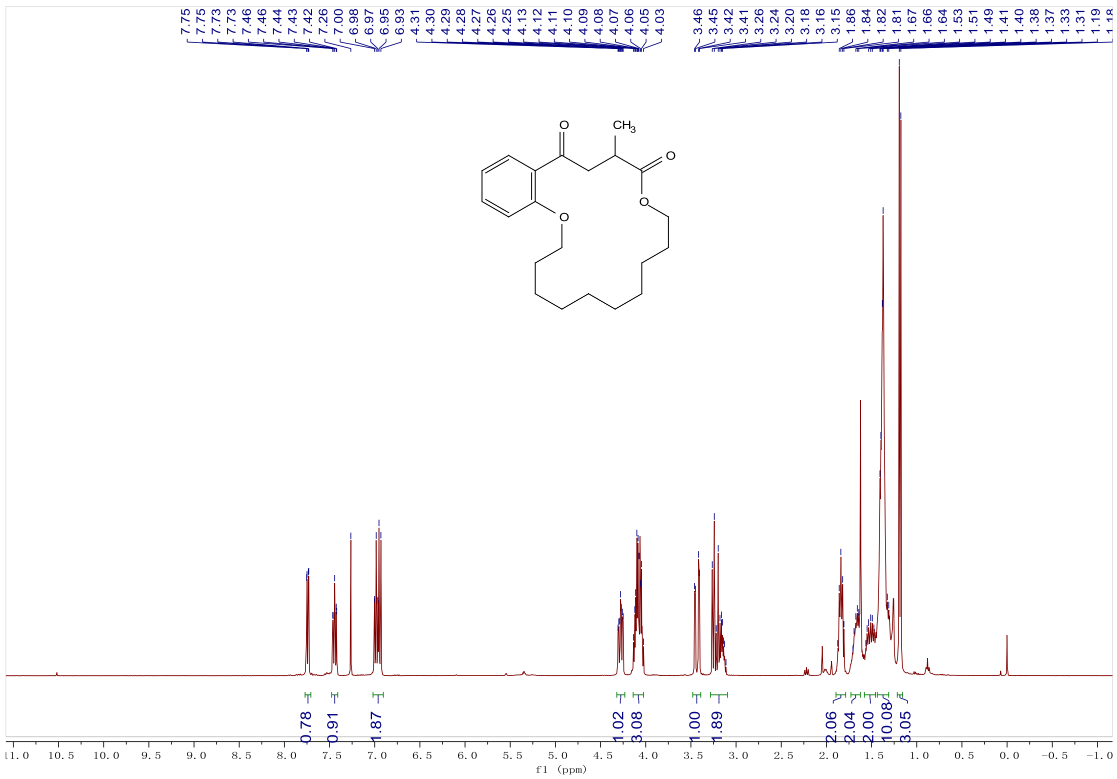

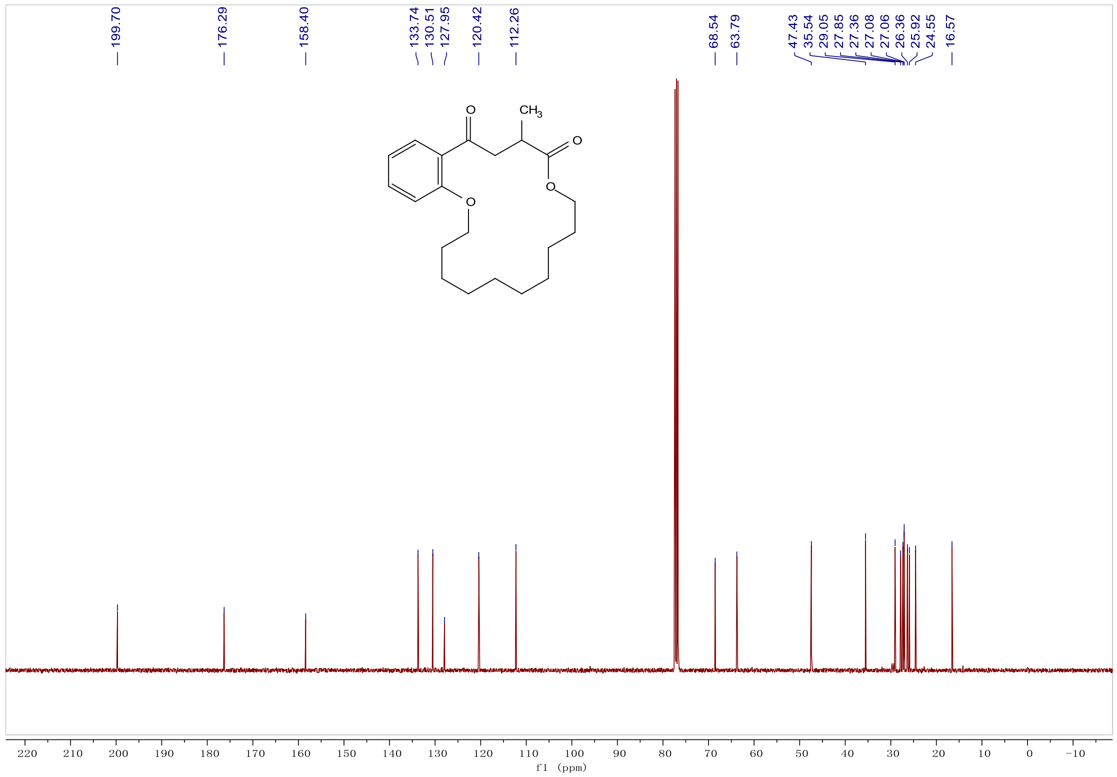

Supplementary Figure 108. ^{1}H and ^{13} C NMR spectra for compound **21c**

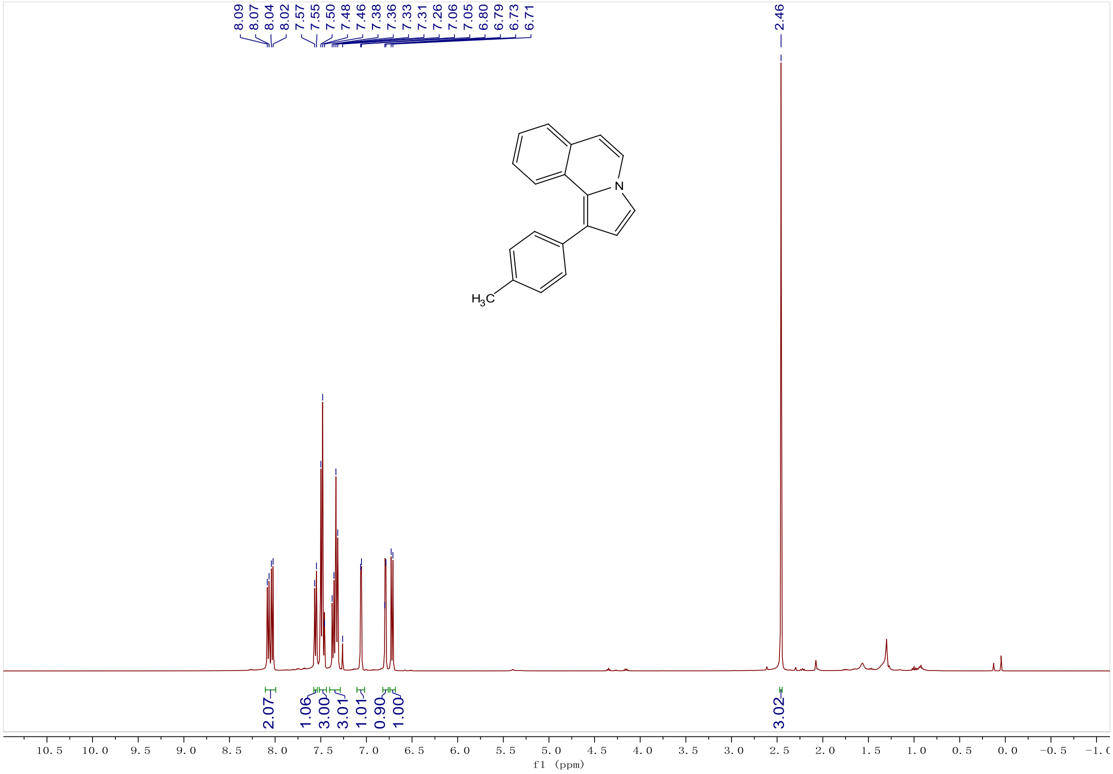

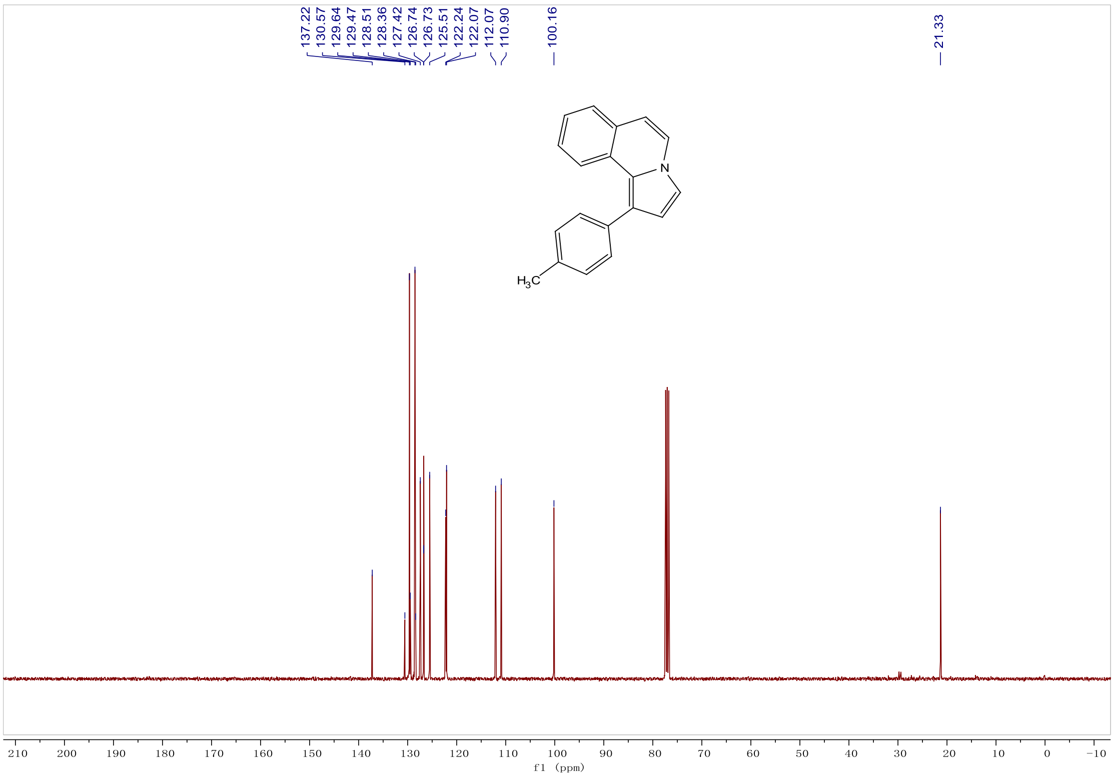

Supplementary Figure 109. ^{1}H and ^{13}C NMR spectra for compound **22**

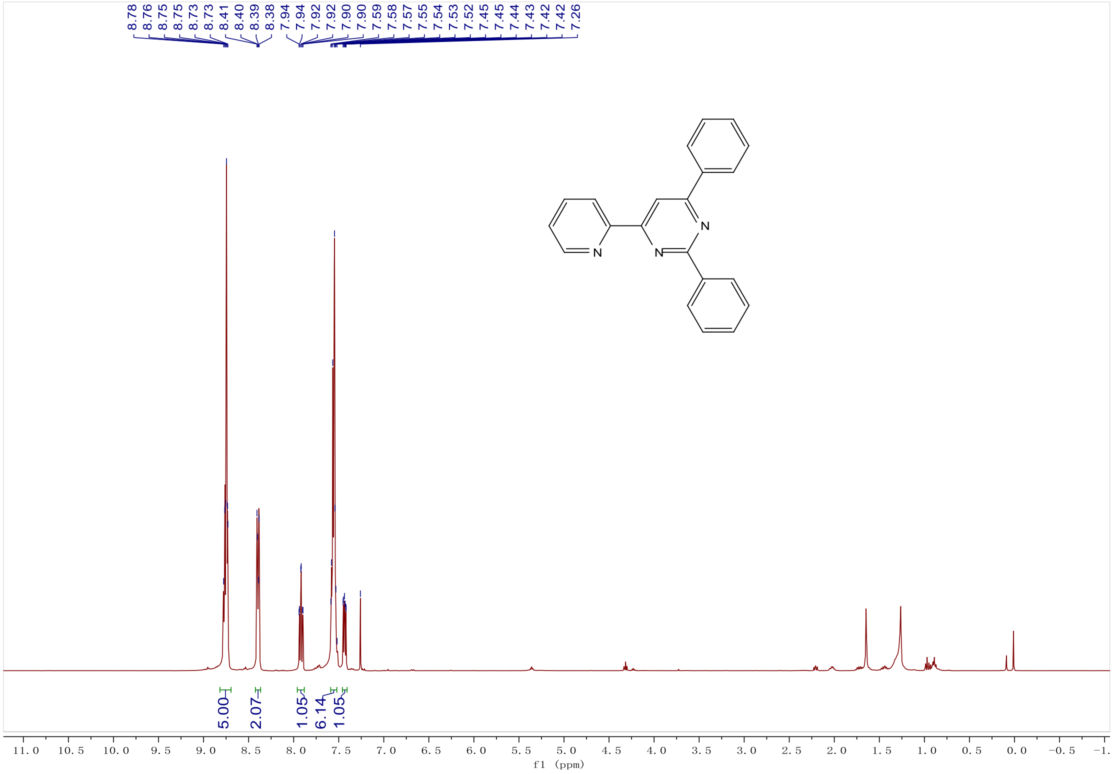

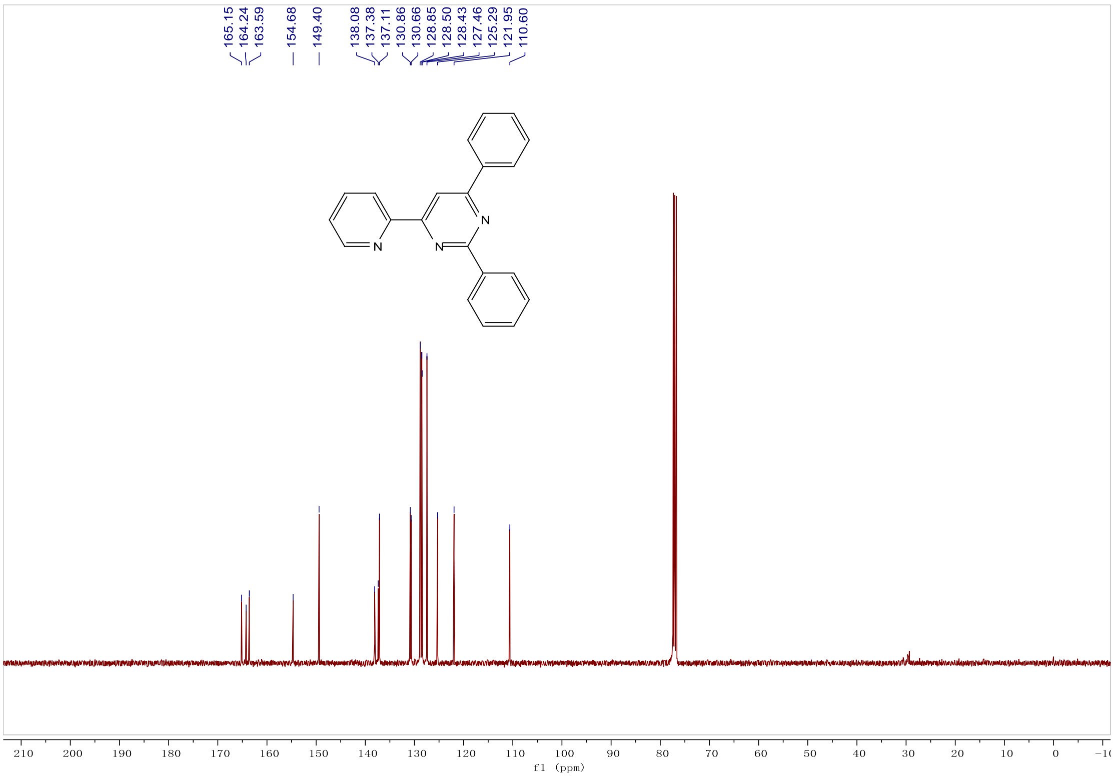

Supplementary Figure 110. ^{1}H and ^{13}C NMR spectra for compound **24**

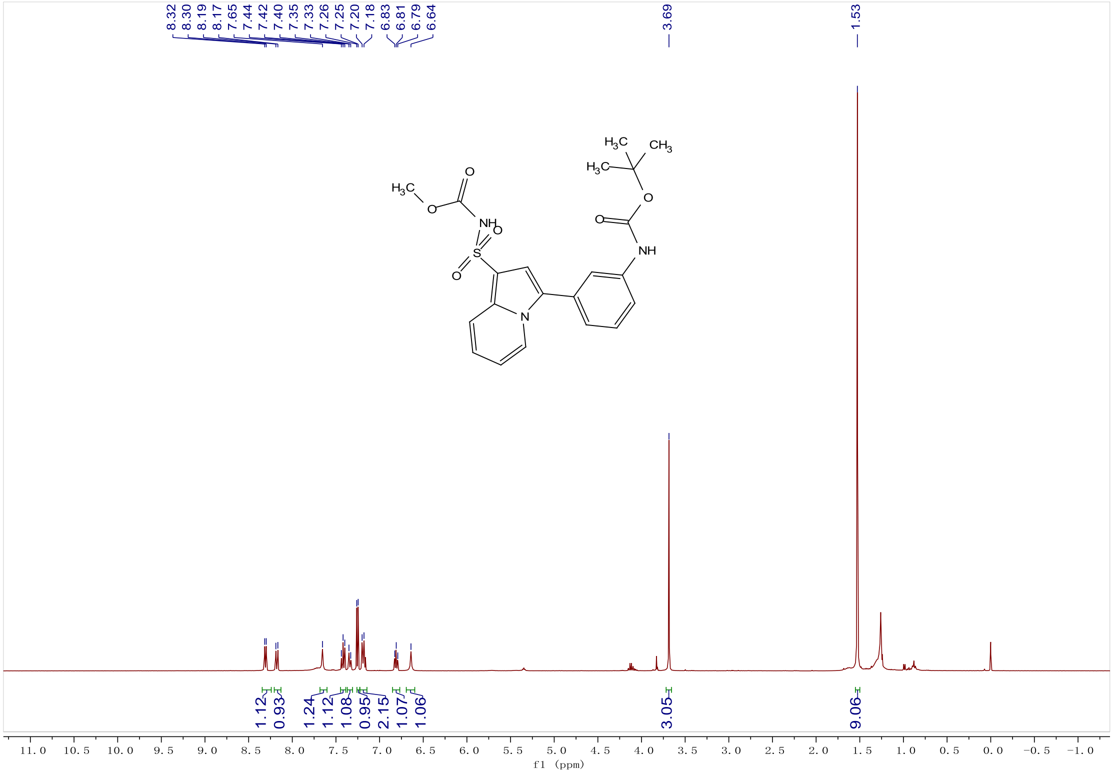

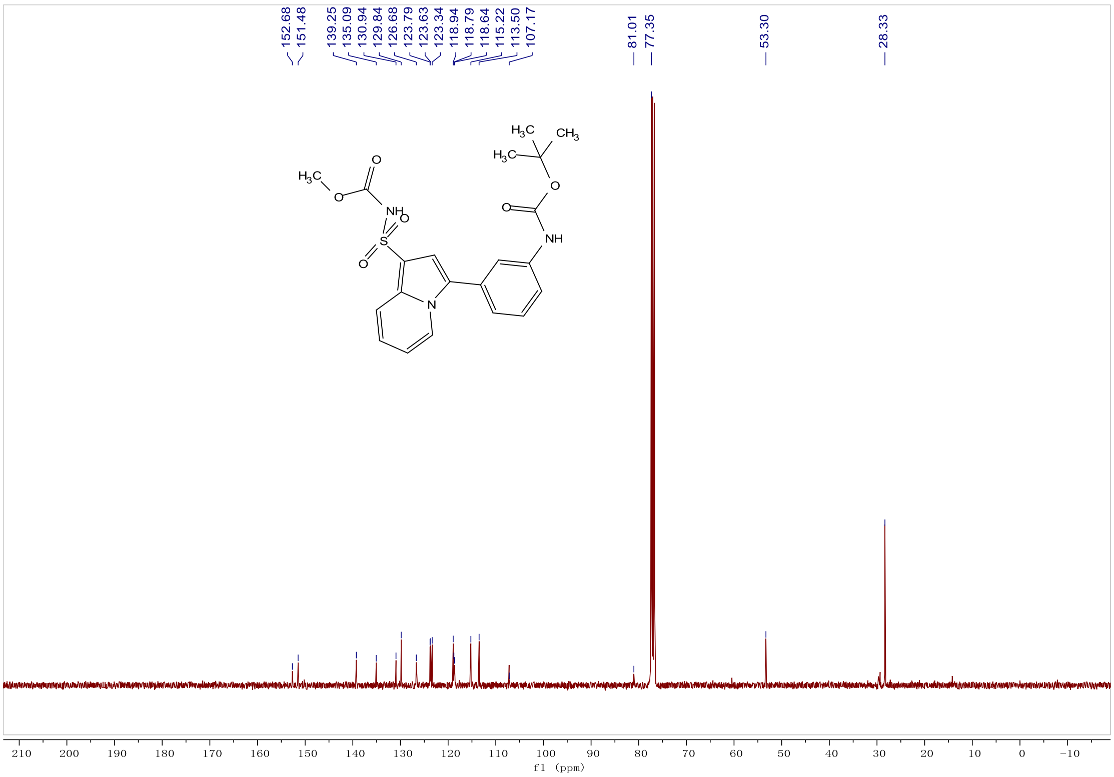

Supplementary Figure 111. ^{1}H and ^{13} C NMR spectra for compound **26**

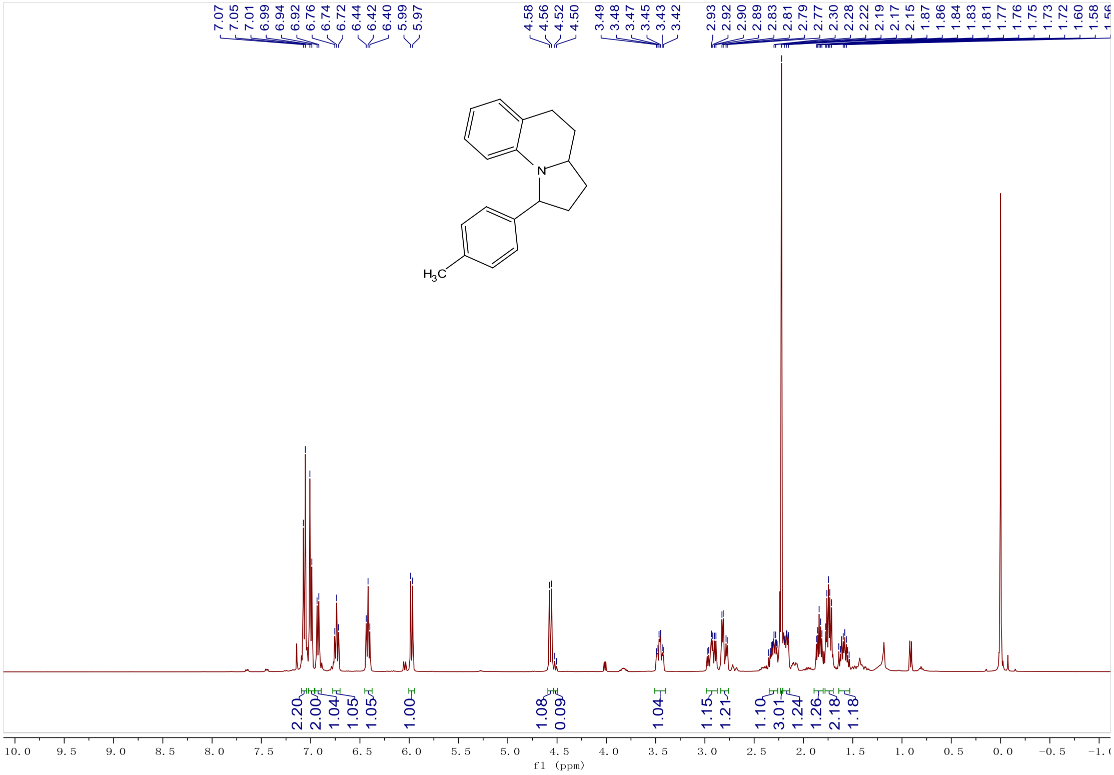

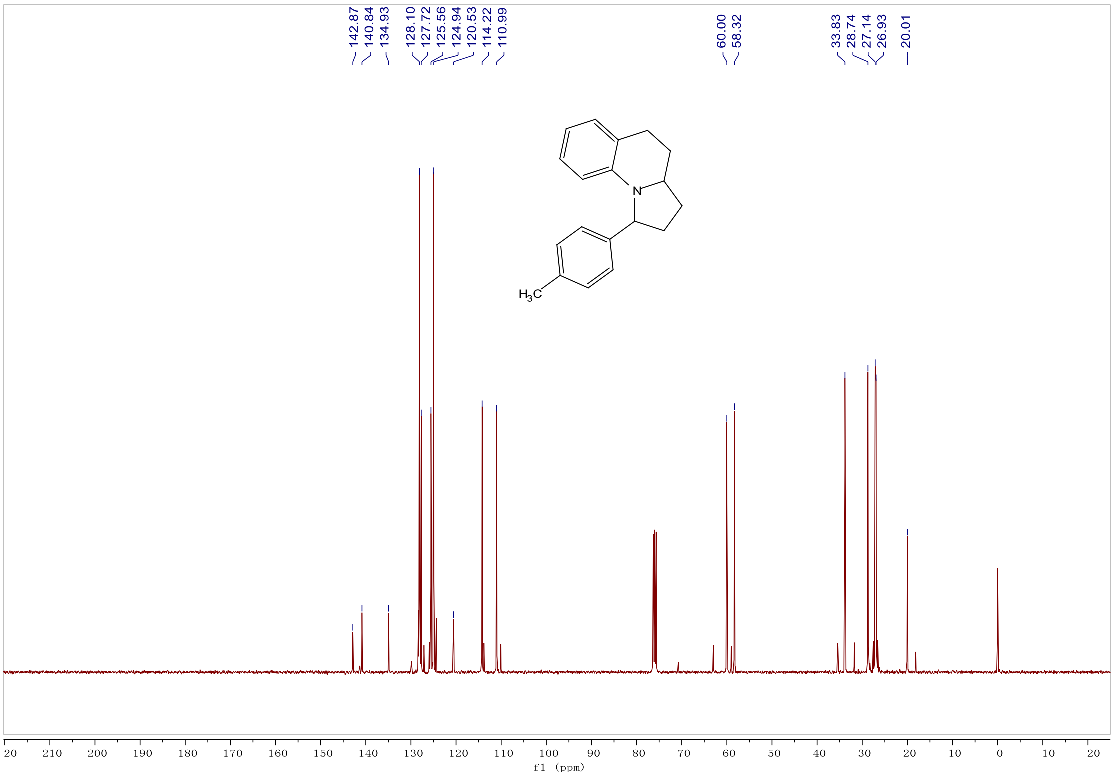

Supplementary Figure 112. ^{1}H and ^{13}C NMR spectra for compound **28**

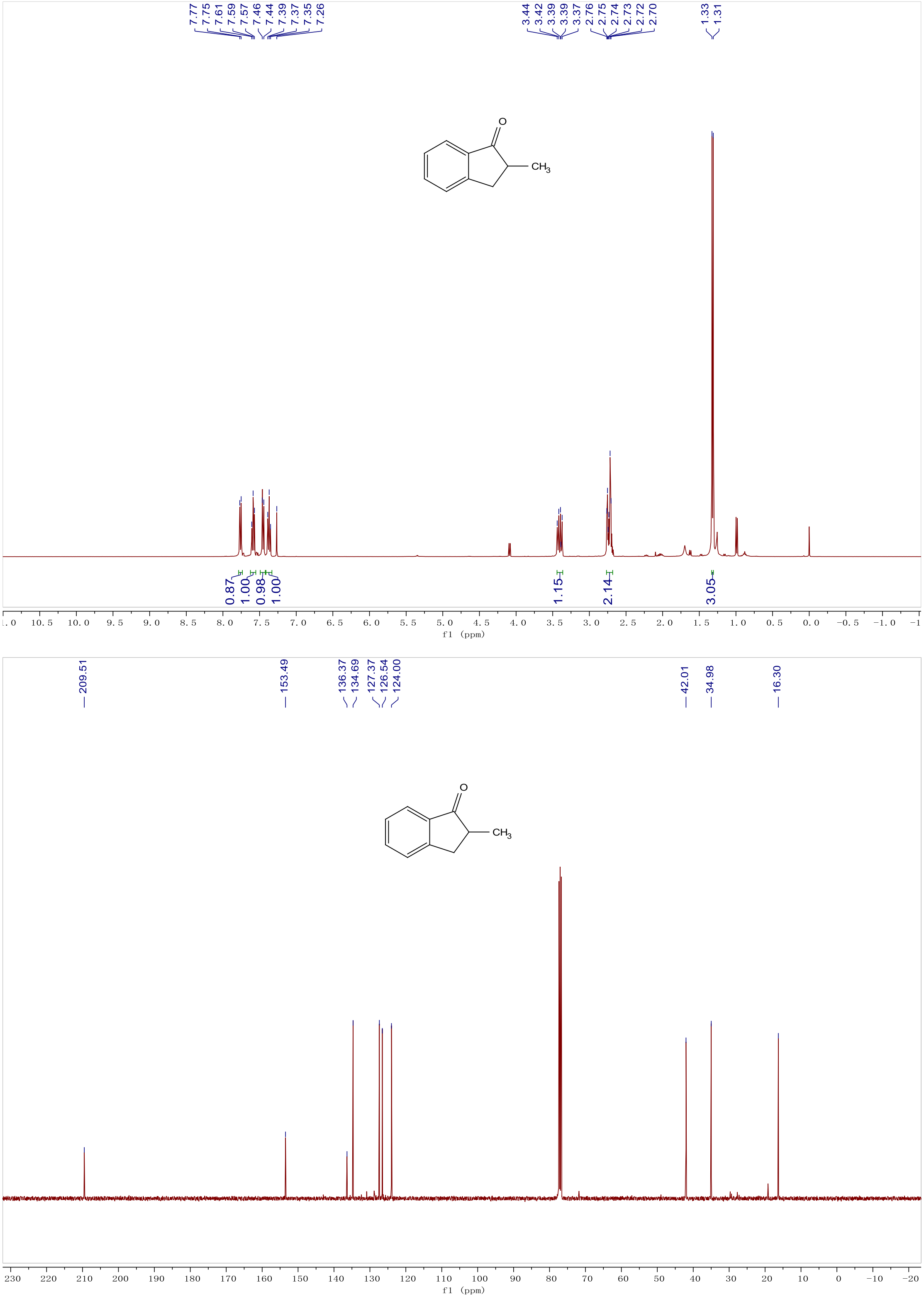

Supplementary Figure 113. ^{1}H and ^{13} C NMR spectra for compound **30**

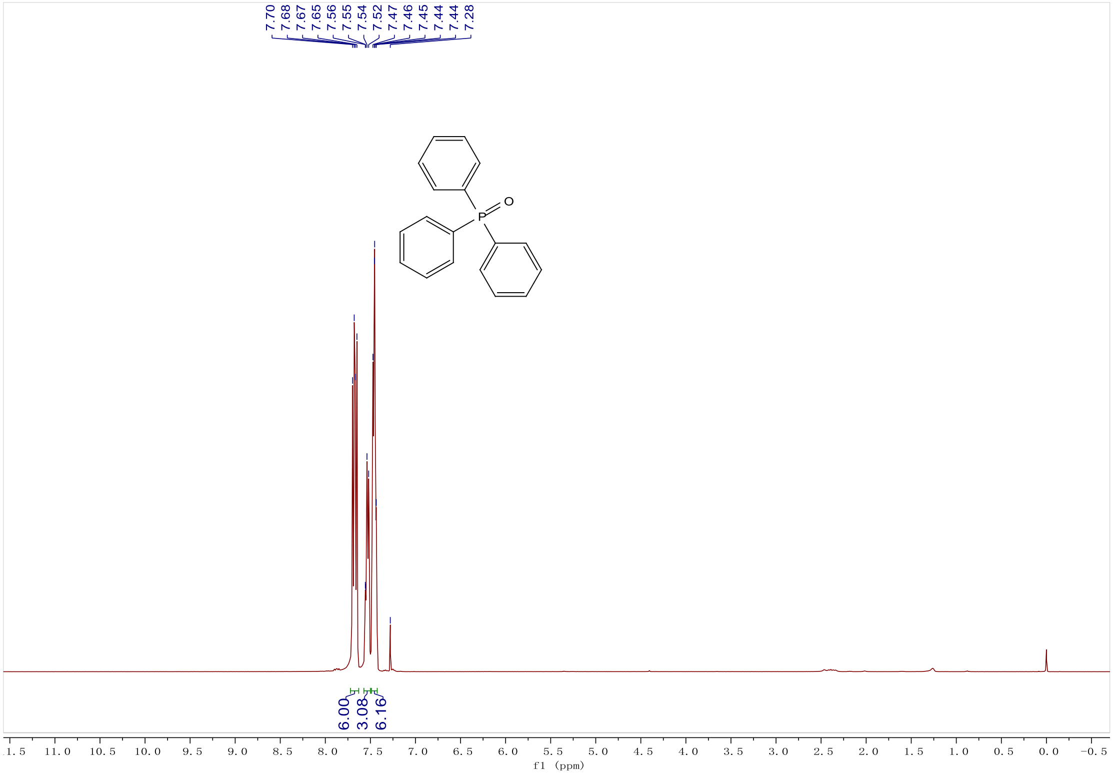

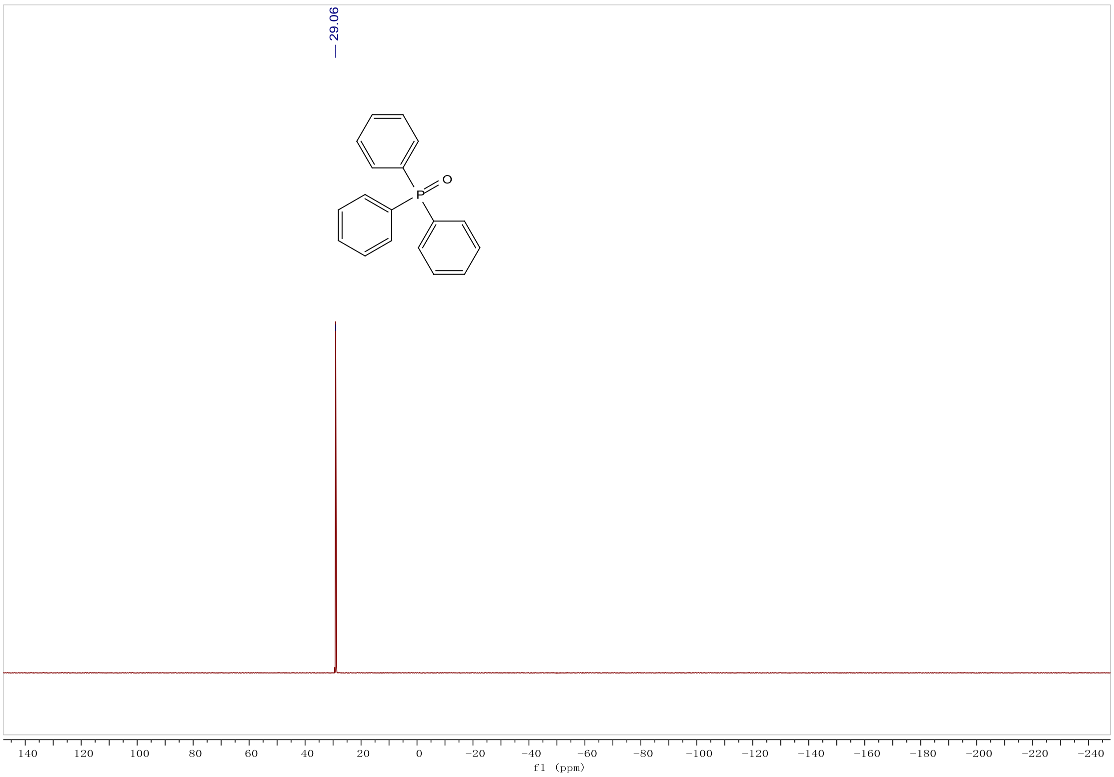

Supplementary Figure 114. ^{1}H and ^{31}P NMR spectra for triphenylphosphine oxide

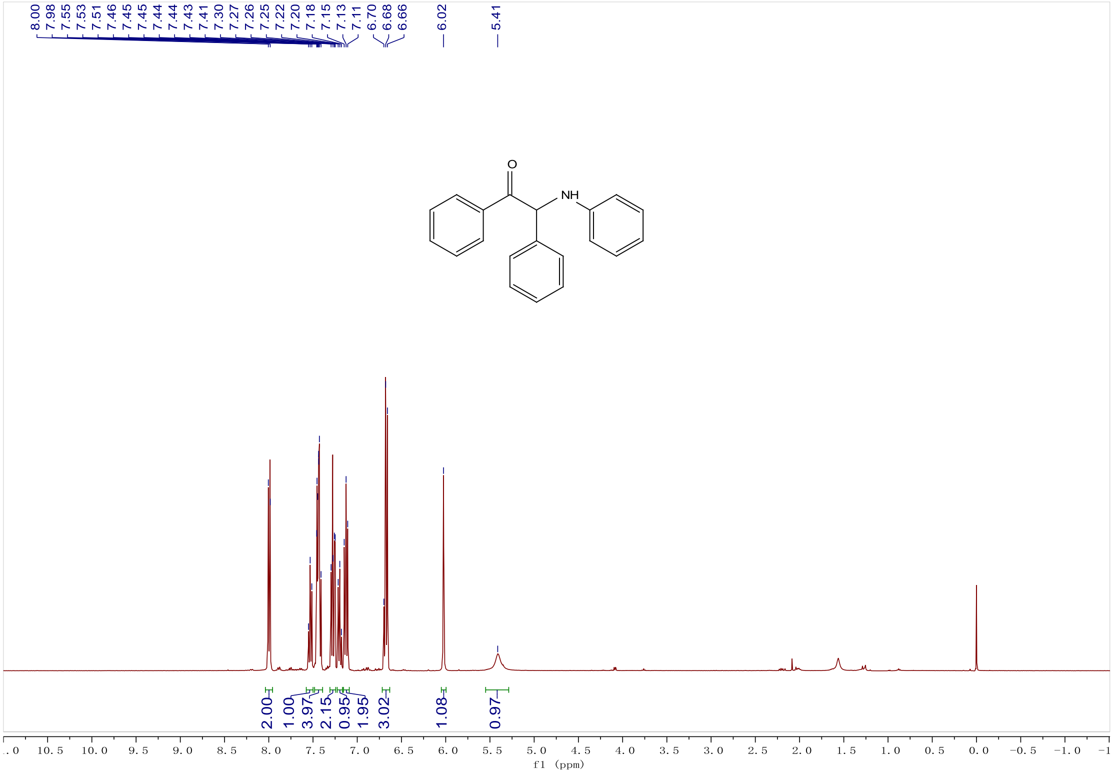

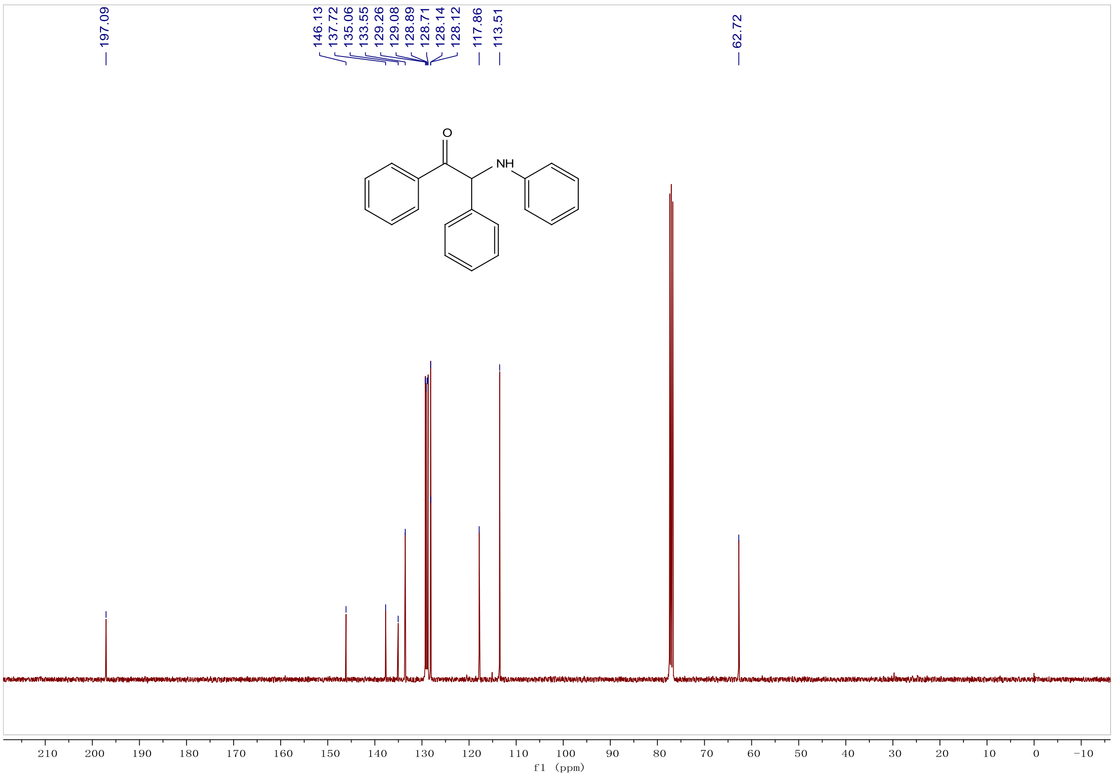

Supplementary Figure 115. ^{1}H and ^{13} C NMR spectra for compound **38a**

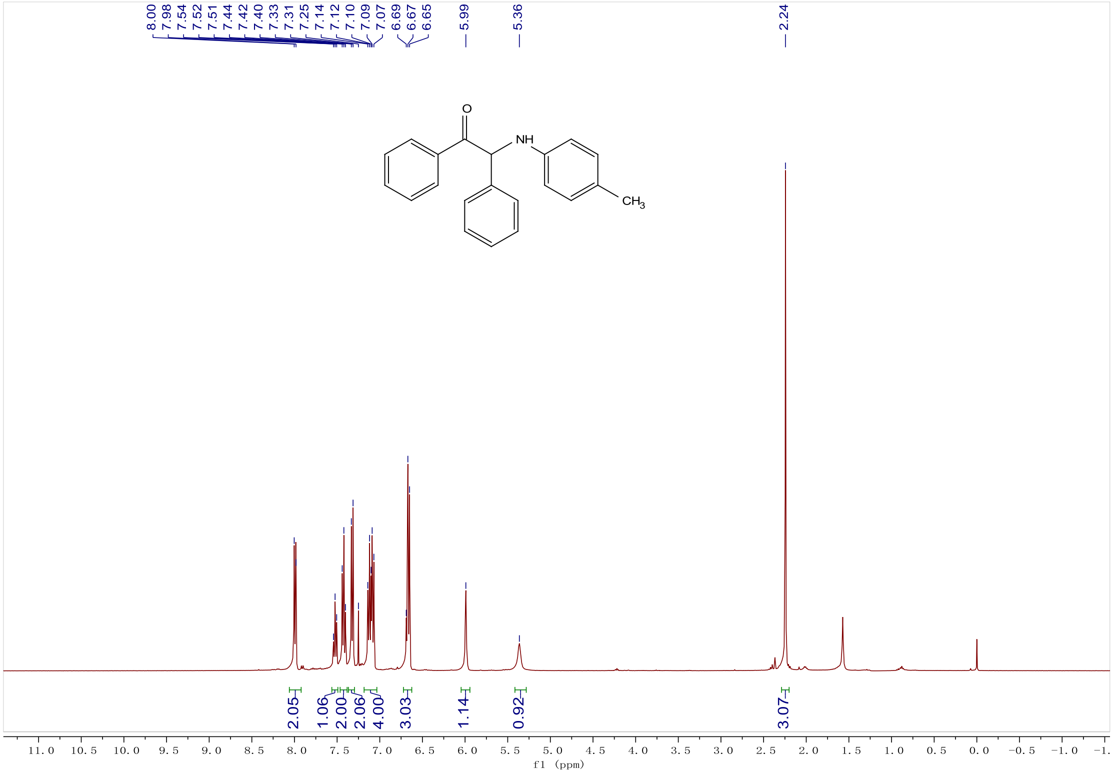

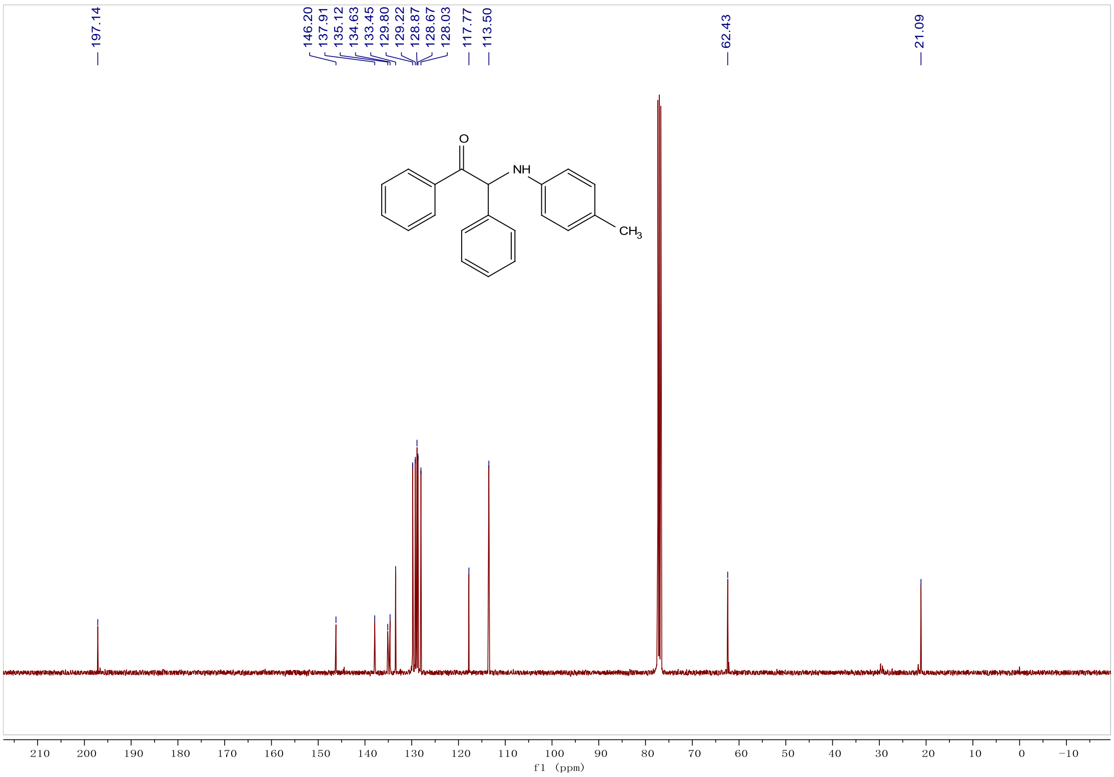

Supplementary Figure 116. ^{1}H and ^{13}C NMR spectra for compound **38b**

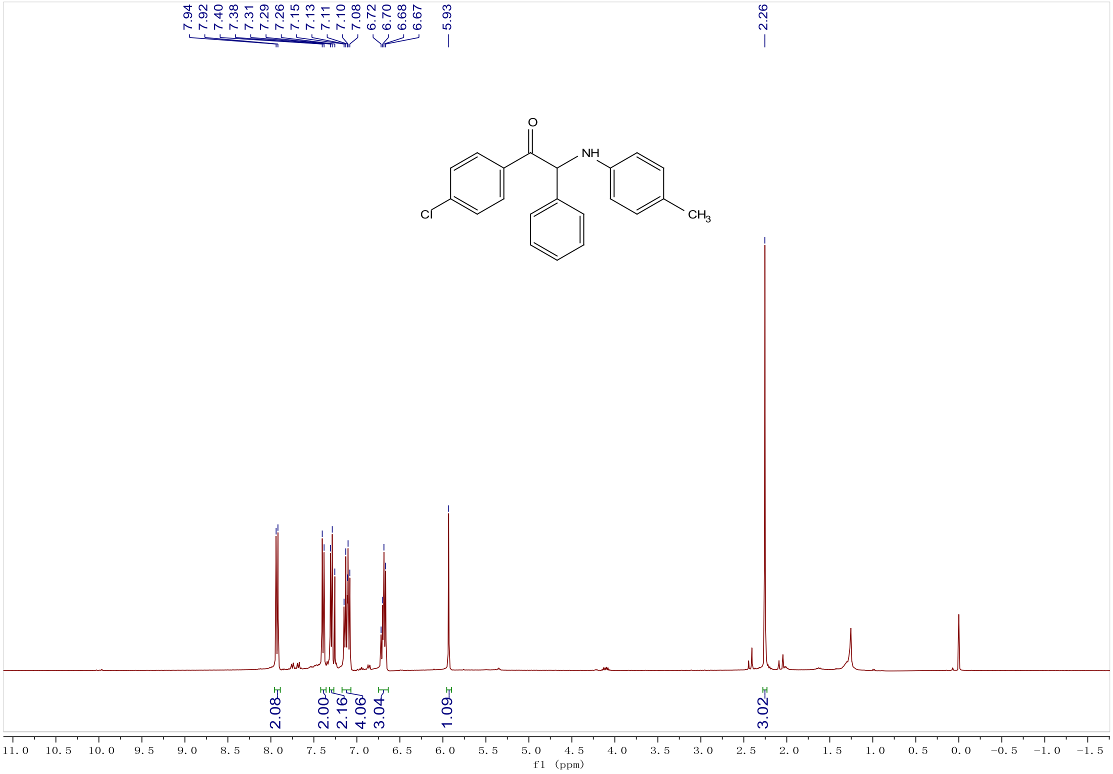

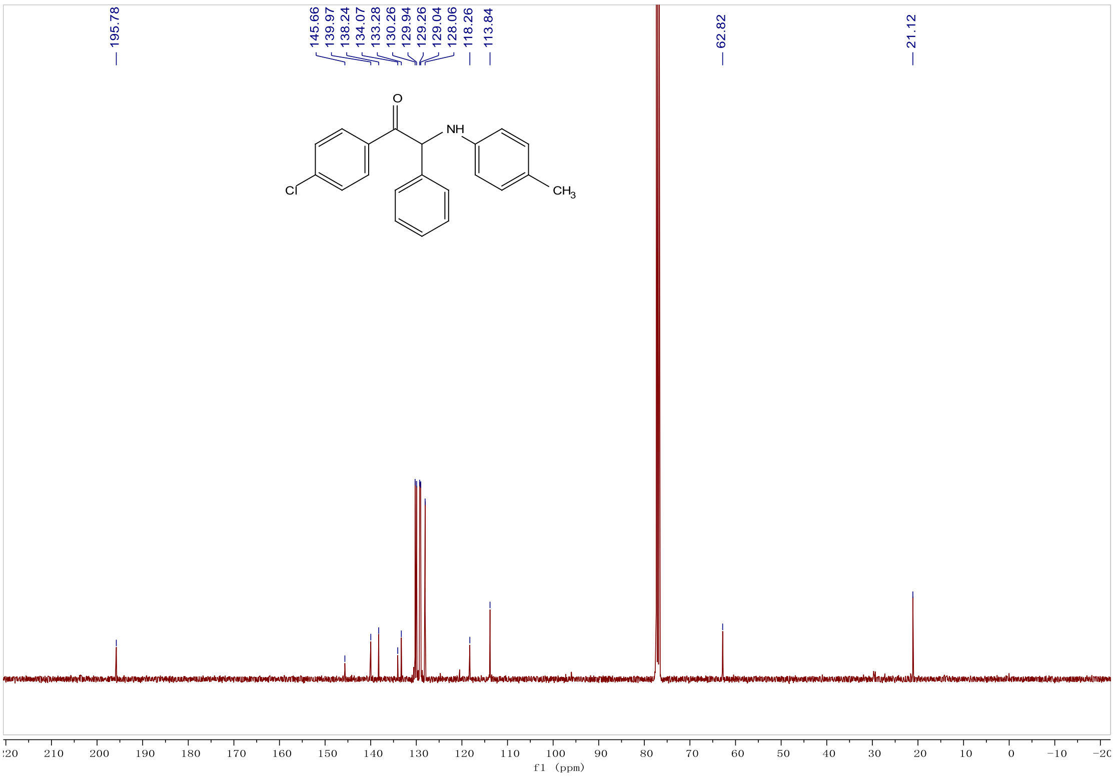

Supplementary Figure 117. ^{1}H and ^{13} C NMR spectra for compound **38c**

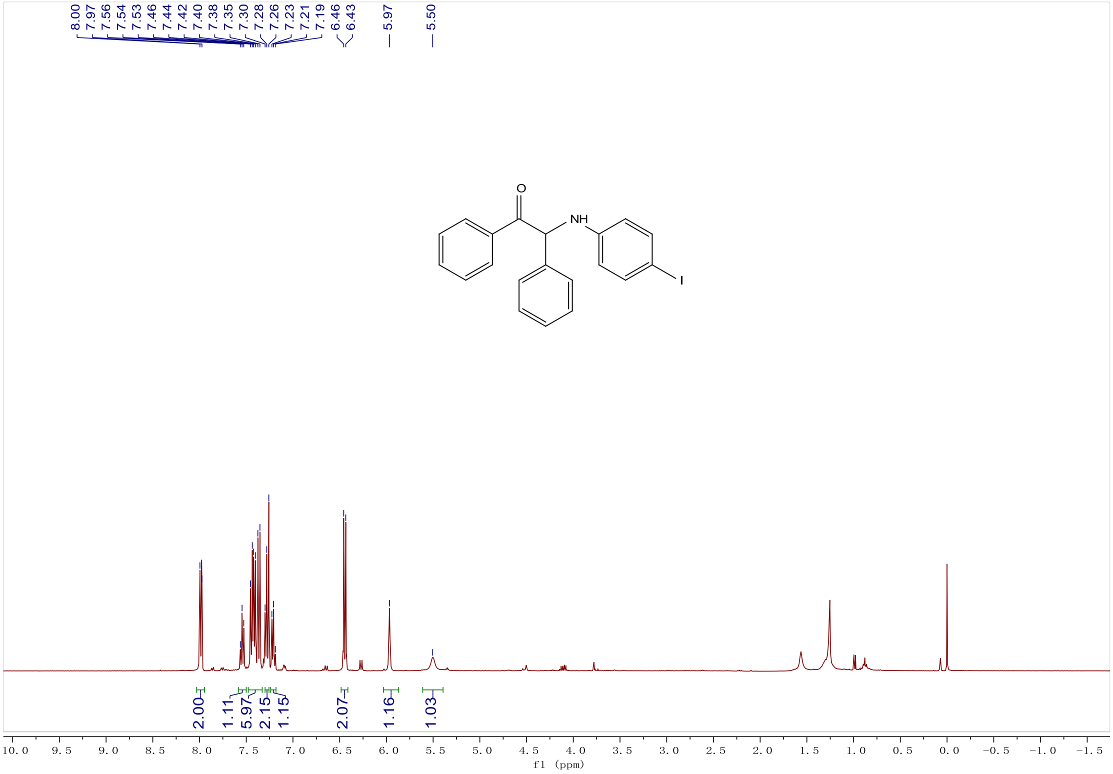

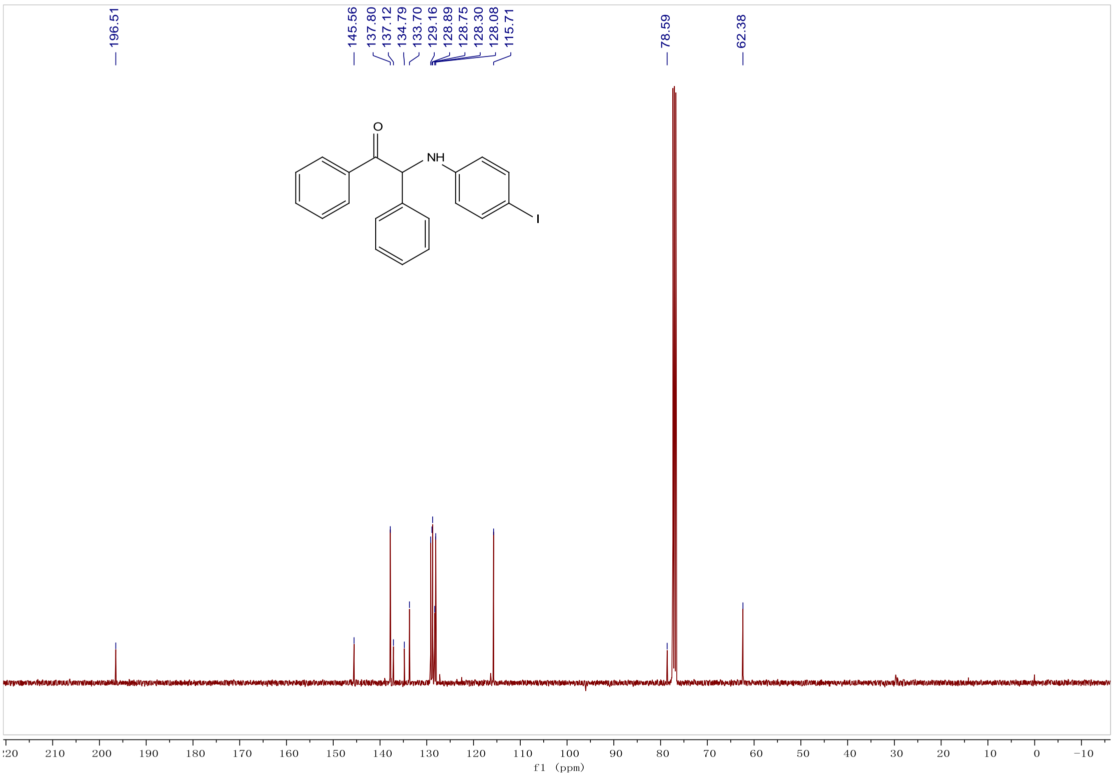

Supplementary Figure 118. ^{1}H and ^{13} C NMR spectra for compound **38d**

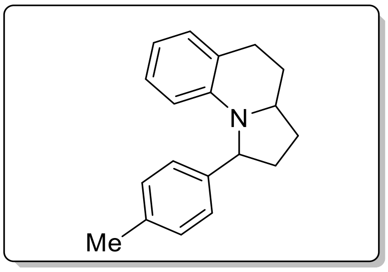

1-(*p*-tolyl)-1,2,3,3a,4,5-hexahydropyrrolo[1,2-*a*]quinoline

<Chromatogram>

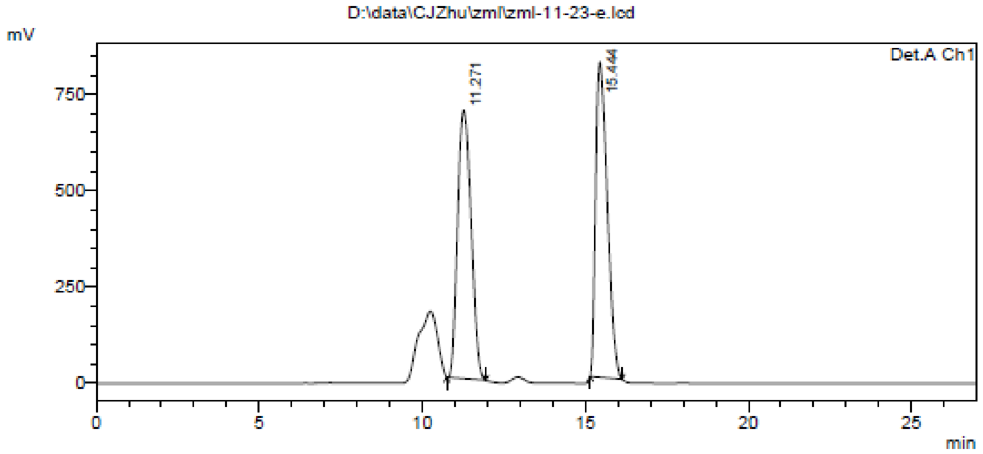

Peak#	Ret. Time	Area	Height	Area %	Height %
1	11.271	20140906	697337	49.742	45.975
2	15.444	20349545	819444	50.258	54.025
Total		40490450	1516781	100.000	100.000

<Chromatogram>

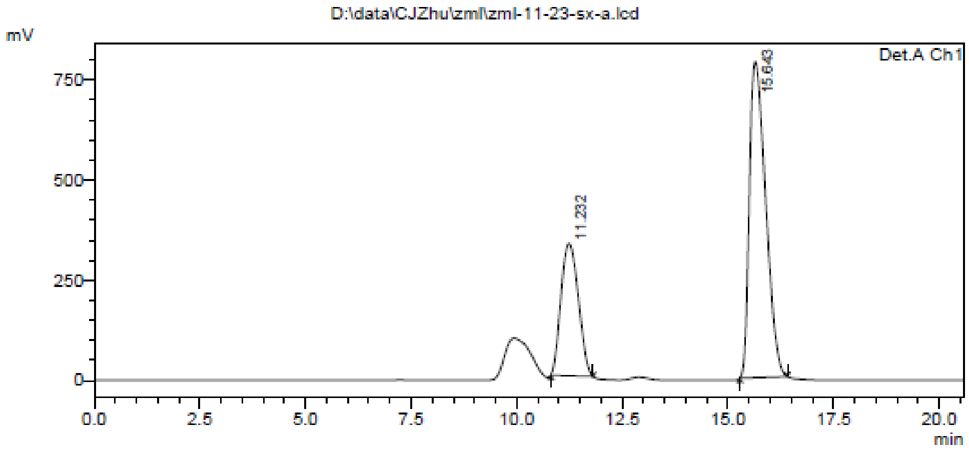

Peak#	Ret. Time	Area	Height	Area %	Height %
1	11.232	9476207	330812	30.939	29.546
2	15.643	21152815	788825	69.061	70.454
Total		30629021	1119637	100.000	100.000

Supplementary Figure 119 .HPLC spectra for **28**

Supplementary References

1. Boekelheide, V. & Godfrey, J. C. Syntheses of 7, 8-Benzopyrrocoline Derivatives. A Novel Reaction of Reissert Compounds. *J. Am. Chem. Soc.* **75**, 3679-3685 (1953).

2. Zhan, J.-L., Wu, M.-W., Chen, F. & Han, B. Cu-Catalyzed [3 + 3] Annulation for the Synthesis of Pyrimidines via *β*-C(sp3)–H Functionalization of Saturated Ketones. *J. Org. Chem.* **81**, 11994-12000 (2016).

3. Li, J. J. et al. A Synthesis of N-Bridged 5,6-Bicylic Pyridines via A Mild Cyclodehydration Using the Burgess Reagent and Discovery of a Novel Carbamylsulfonylation Reaction. *Org. Lett.* **10**, 2897-2900 (2008).

4. Rueping, M. & Hubener, L. Enantioselective Synthesis of Quinolizidines and Indolizidines via a Catalytic Asymmetric Hydrogenation Cascade. *Synlett* 1243-1246 (2011).

5. Davis, T. A., Hyster, T. K. & Rovis, T. Rhodium (III)-Catalyzed Intramolecular Hydroarylation, Amidoarylation, and Heck-Type Reaction: Three Distinct Pathways Determined by an Amide Directing Group. *Angew.Chem. Int. Ed.*, **52**, 14181-14185 (2013).

6. Luis, S. V., Ferrer, P. & Burguete, M. I. Generation of Benzyne in the Thermal Decomposition of 2-Carboxyphenyl *P*-Toluenesulfonate. *J. Org. Chem.* **55**, 3808-3812 (1990).

7. Feng, C. & Loh, T.-P. Rhodium(III)-Catalyzed Cross-Coupling of Alkenylboronic Acids and N-Pivaloyloxylamides. *Org. Lett.* **16**, 3444-3447 (2014).

8. Hada, K. et al. Angiogenesis Inhibitors Identified by Cell-Based High-throughput Screening: Synthesis, Structure–Activity Relationships and Biological Evaluation of 3-[(E)-styryl]benzamides that Specifically Inhibit Endothelial Cell Proliferation. *Bioorg. Med. Chem.* **20**, 1442-1460 (2012).

9. Hewitt, J. F. M., Williams, L., Aggarwal, P., Smith, C. D. & France, D. J. Palladium-Catalyzed Heteroallylation of Unactivated Alkenes – Synthesis of Citalopram. *Chem. Sci.* **4,** 3538-3543 (2013).

10. Peng, Y. et al. Iron-Catalyzed Anti-Markovnikov Hydroamination of Vinylpyridines. *Asian J. Org. Chem.* **6**, 694-697 (2017).

11. Saxena, A., Choi, B. & Lam, H. W. Enantioselective Copper-Catalyzed Reductive Coupling of Alkenylazaarenes with Ketones. *J. Am. Chem. Soc.* **134**, 8428-8431 (2012).

12. Lee, K. N., Lei, Z. & Ngai, M.-Y. *β*-Selective Reductive Coupling of Alkenylpyridines with Aldehydes and Imines via Synergistic Lewis Acid/Photoredox Catalysis. *J. Am. Chem. Soc.* **139**, 5003-5006 (2017).

13. Zhou, B., Hu, Y. & Wang, C. Manganese-Catalyzed Direct Nucleophilic C(sp2) H Addition to Aldehydes and Nitriles. *Angew. Chem. Int. Ed.* **54**, 13659-13663 (2015).

14. Walkowiak, J., del Campo, T. M., Ameduri, B. & Gouverneur, V. Syntheses of Mono-, Di-, and Trifluorinated Styrenic Monomers. *Synthesis* **11**, 1883-1890 (2010).

15. Kawashima, S., Aikawa, K., & Mikami, K. Rhodium-Catalyzed Hydrocarboxylation of Olefins with Carbon Dioxide. *Eur. J. Org. Chem.* **19**, 3166-3170 (2016)
